# Asymmetric Total Synthesis of 4,9,10-Trihydroxyguaia-11(13)en-12,6-olide and Discovery of Its Anticancer Activity against Atypical Teratoid Rhabdoid Tumor

**DOI:** 10.1021/acscentsci.5c00332

**Published:** 2025-06-03

**Authors:** Hyejin Lee, Hongjun Jang, Hwan Myung, Angela Rivera, Anna F. Averette, Joseph Heitman, Jiyong Park, Deukjoon Kim, Hyoungsu Kim, Jiyong Hong

**Affiliations:** † Department of Chemistry, 3065 Duke University, Durham, North Carolina 27708, United States; ‡ College of Pharmacy and Research Institute of Pharmaceutical Science and Technology (RIPST), 34919 Ajou University, Suwon 16499, Republic of Korea; § Department of Chemistry, Korea Advanced Institute of Science and Technology (KAIST), Daejeon 34141, Republic of Korea; ∥ Center for Catalytic Hydrocarbon Functionalizations, 364806 Institute for Basic Science (IBS), Daejeon 34141, Republic of Korea; ⊥ Department of Molecular Genetics and Microbiology, 12277 Duke University School of Medicine, Durham, North Carolina 27710, United States; # Department of Pharmacology and Cancer Biology, 12277 Duke University School of Medicine, Durham, North Carolina 27710, United States; ∇ College of Pharmacy, Seoul National University, Seoul 08826, Republic of Korea

## Abstract

The guaianolide family of sesquiterpene lactones is known for its distinctive structural features and diverse biological activities. 4,9,10-Trihydroxyguaia-11(13)­en-12,6-olide, with an underdetermined absolute stereochemistry (**1** or *ent*-**1**), is a newly identified 6,12-guaianolide isolated from the genus . Motivated by the potential biological activity of the natural product, we pursue its stereoselective synthesis. Starting from (*R*)-limonene, an asymmetric total synthesis of 4α,9α,10α-trihydroxyguaia-11(13)­en-12,6α-olide (**1**) is accomplished in 20 steps with an overall yield of 4%, utilizing key transformations such as stereoselective reductive epoxide opening and additions of methyl lithiopropiolate and allyl cuprate. Most significantly, preliminary biological testing uncovers new anticancer activity of **1** against rare and aggressive childhood atypical teratoid rhabdoid tumor (ATRT) and other cancer cell lines. We anticipate that our synthetic strategy will enable the development of chemical probes and derivatives derived from **1** for mechanism of action studies and anticancer drug discovery.

## Graphical abstract

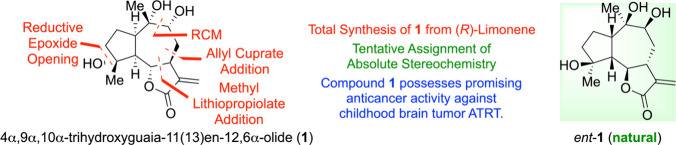

## Introduction

Evolution has generated a multitude of natural products, each with a unique chemical diversity that ensures effective interaction with biological macromolecules. This structural diversity positions natural products as a prime source for innovative drug discovery.^1–4^ Notable examples of FDA-approved drugs derived from natural products include antibiotics such as penicillin and erythromycin, the anticancer agent paclitaxel, and digoxin for treating heart failure. These examples underscore the critical contribution of natural products to therapeutic development. Additionally, natural products have carved a niche in chemical biology, functioning as modulators of biomolecular activity.^3^ Agents like brefeldin A, forskolin, cyclosporine A, and rapamycin have become indispensable in unraveling the intricacies of cellular machinery and signal transduction pathways.

In this regard, guaianolide family sesquiterpenes are an appealing class of natural products that have attracted considerable attention due to their diverse pharmacological properties.^5–7^ They belong to a class of sesquiterpene lactones and possess unique 6,12-, 8,12-, and *seco*-guaianolide skeletons (Figure 1). They exhibit a range of important biological activities such as anti-inflammatory, anticancer, and antimicrobial activity. Consequently, the molecular diversity and biological activity of guaianolide sesquiterpenes have garnered significant interest from several synthetic research groups.^5,8–16^

**Figure 1:**
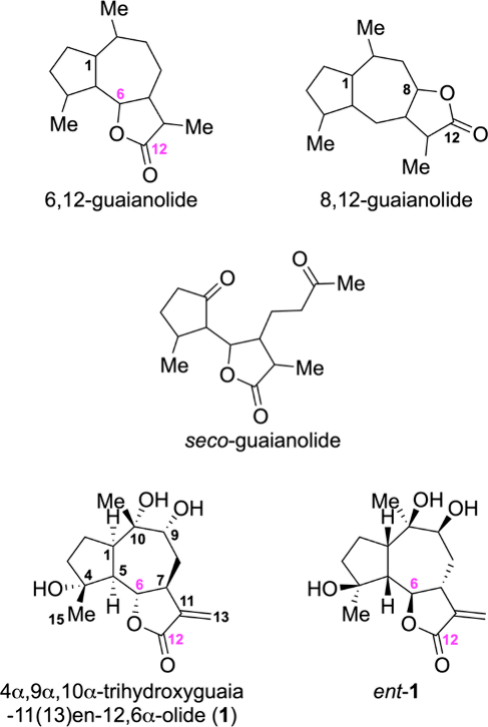
Representative skeletons of guaianolide sesquiterpenes and the two possible enantiomeric structures for 4,9,10-trihydroxyguaia-11(13)­en-12,6-olide (**1** and *ent*-**1**).

In 2020, Perveen, Taglialatela-Scafati, and co-workers isolated a sesquiterpene lactone with a 6,12-guaianolide skeleton from the genus , notable for its unique structural features and potential therapeutic applications.^17^ The natural product demonstrated potent antifungal activity against (MIC 0.21 μg/mL) and (MIC 0.25 μg/mL). Additionally, it exhibited modest antibacterial activity against both Gram-positive and Gram-negative pathogenic bacteria, such as , , and , with minimal inhibitory concentration (MIC) ranging from 2.3–5.7 μg/mL. The structure and relative stereochemistry of the natural product was firmly established by extensive spectroscopic analysis, including 1D and 2D NMR techniques. However, since the absolute stereochemistry of the natural product remains undetermined, two possible enantiomeric structures—4α,9α,10α-trihydroxyguaia-11(13)­en-12,6α-olide (**1**) and its enantiomer *ent*-**1**—are presented in Figure 1.

In broad connection with our interest in the synthesis and study of mechanisms of action of biologically important natural products,^18–24^ we undertook a total synthesis of **1** to develop a robust and efficient synthetic route that would be easily amenable to the development of derivatives and chemical probes for biological and mechanistic studies. Since Perveen et al. did not report the absolute stereochemistry of the natural product, and two enantiomeric structures are possible (**1** and *ent*-**1**, Figure 1), we arbitrarily selected **1** as the target natural product. Here, we report the asymmetric total synthesis and determination of the absolute stereochemistry of **1**. Our synthesis began with (*R*)-limonene and employed a SmI_2_-mediated stereoselective reductive epoxide opening and stereoselective additions of methyl lithiopropiolate and allyl cuprate as key steps. We also discovered the new and promising anticancer activity of **1** against childhood atypical teratoid rhabdoid tumor (ATRT), a rare and aggressive form of cancer. We anticipate that our synthetic strategy will provide a solid foundation for future development of anticancer agents derived from **1**. Additionally, it will facilitate the preparation of mechanistic chemical probes, thereby deepening our understanding of the molecular interactions of **1** in biological systems.

## Results and Discussion

Figure 2 illustrates our synthetic approach to 4α,9α,10α-trihydroxyguaia-11(13)­en-12,6α-olide (**1**). Our retrosynthetic analysis for **1** enlisted a stereoselective reductive opening of an epoxide to introduce the C4-hydroxyl group of **1**. Additionally, the formation of the γ-lactone fragment in **1** was envisioned through a stereoselective addition of methyl lithiopropiolate followed by lactonization. We further envisioned that the synthesis of **1** could begin with either (*R*)-carvone (**5**) via a hydration route or (*R*)-limonene (**6**) via an epoxide opening route.

**Figure 2:**
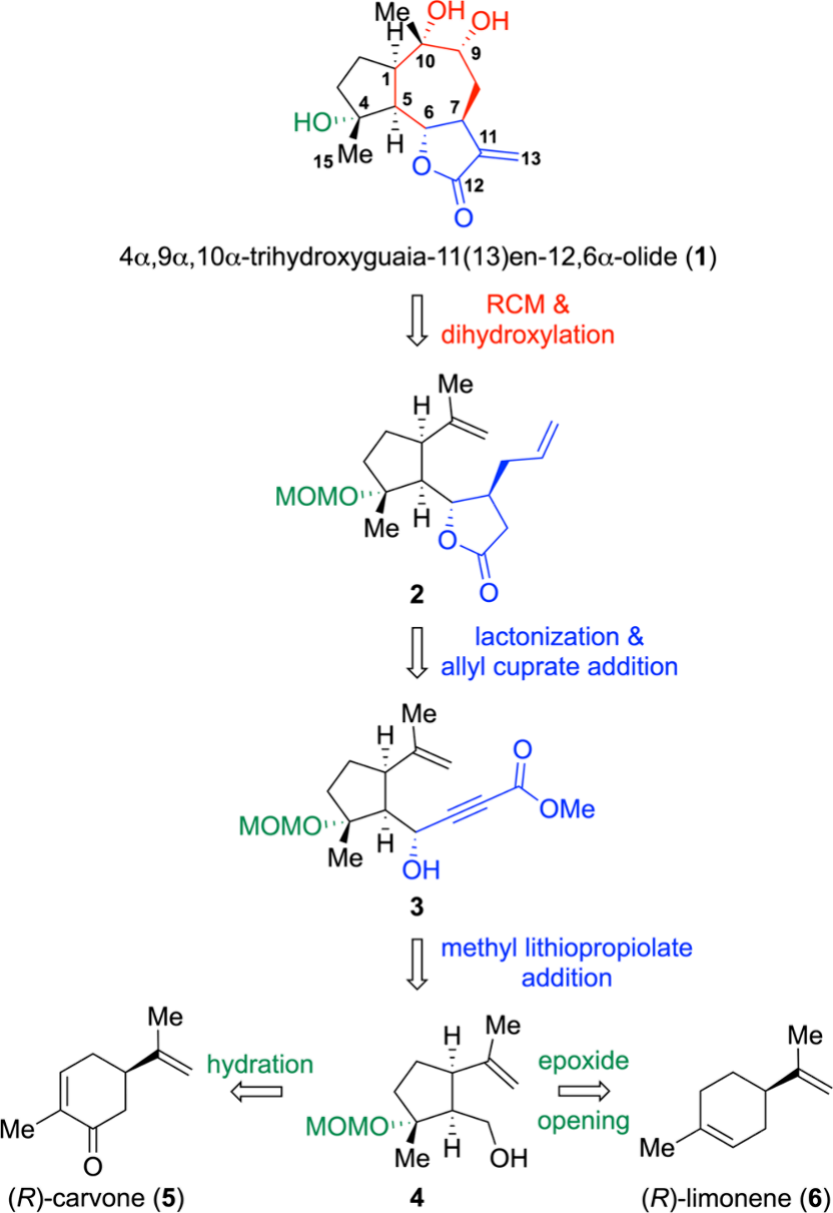
Retrosynthetic analysis of 4α,9α,10α-trihydroxyguaia-11(13)­en-12,6α-olide (**1**).

Our synthetic endeavor began with the preparation of the appropriately functionalized cyclopentylmethyl alcohol **4**. Historically, the syntheses of structurally similar cyclopentyl moieties have predominantly employed the Favorskii rearrangement starting from carvone.^9,10,13,14,25–29^ Therefore, we began with (*R*)-carvone (**5**) to stereoselectively synthesize compound **4** (Scheme 1). Starting from (*R*)-carvone, we followed the reported procedures^9,10^ and prepared tribromide **8**. The Favorskii rearrangement of **8** proceeded smoothly, yielding the intermediate cyclic imidate **9**, which was used directly in the subsequent step. Direct hydrolysis of **9** under acidic conditions afforded the known bicyclic lactone **10** in four steps from (*R*)-carvone (**5**).^9,10^ To install the C4-tertiary hydroxyl group, we employed the hydration reaction, which has been underutilized in the synthesis of guaianolides. The Mukaiyama hydration of **10** in the presence of Fe­(acac)_3_, methyl 4-nitrobenzenesulfonate^30^ proceeded smoothly to give a mixture of the desired (4*R*)-tertiary alcohol **11** and the diastereomeric (4*S*)-alcohol (4*R*:4*S* = 4:1). It is worth noting that among the reaction conditions we tested, a combination of Fe­(acac)_3_ and methyl 4-nitrobenzenesulfonate was most effective to give the highest diastereoselectivity presumably due to the steric demand of the nitroarene (see the Supporting Information for details). MOM protection, reductive lactone opening, and subsequent TBS protection gave tertiary alcohol **13**. When tertiary alcohol **13** was subjected to the Burgess’ dehydration condition,^31,32^ a mixture of the two alkenes was obtained (**14**:**15** = 6.9:1) (see the Supporting Information for details). Final TBS deprotection of **14** completed the 10-step synthesis of the appropriately functionalized cyclopentylmethyl alcohol **4** starting from (*R*)-carvone (**5**) with an overall yield of 12%. However, the hydration route presented several challenges, including modest stereoselectivity in the hydration step and difficulties encountered in separating the desired olefin **14** from its regioisomer **15**. These limitations rendered the hydration route impractical, leading us to explore an alternative synthetic pathway to **4**.

**Scheme 1:**
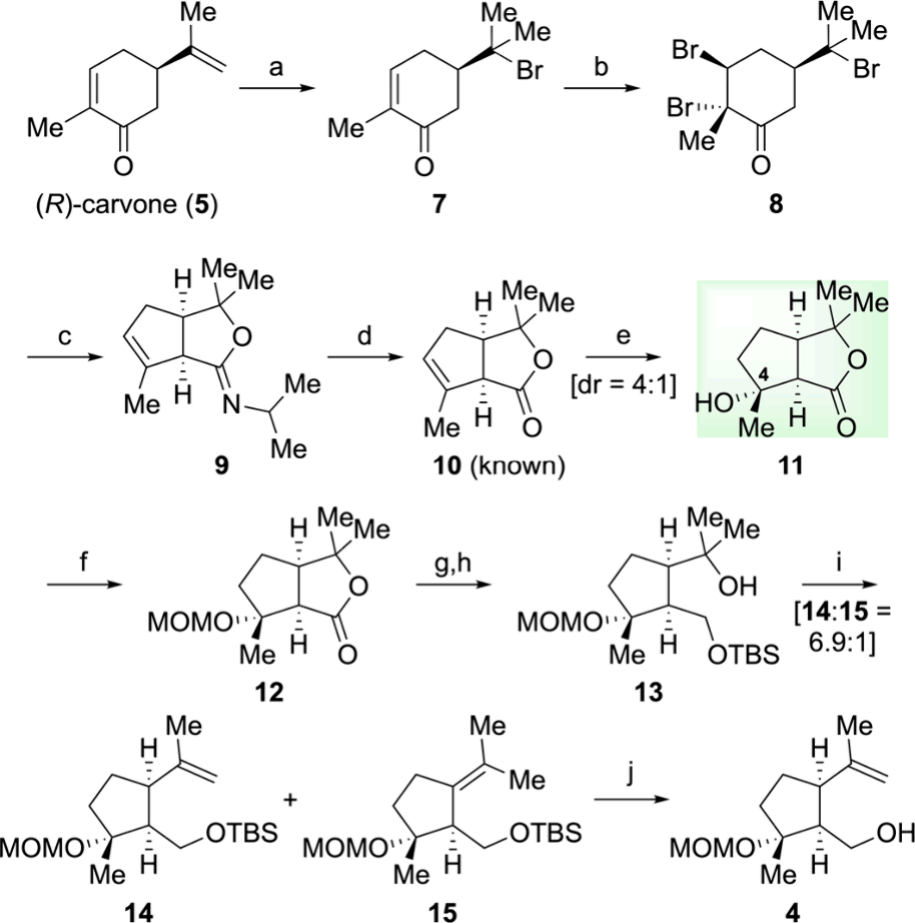
Synthesis of Cyclopentylmethyl Alcohol **4** from (*R*)-Carvone (**5**) via a Hydration Route^a^

a Reaction conditions: (a) HBr, HOAc, 0 °C, 1.5 h; (b) Br_2_, HOAc, 15 to 17 °C, 1.5 h; (c) *i*-PrNH_2_, Et_2_O, 0 to 25 °C, 20 h; (d) 10% HOAc (aq), THF, 50 °C, 5.5 h, 32% for four steps; (e) Fe­(acac)_3_, methyl 4-nitrobenzenesulfonate, PhSiH_3_, NaHCO_3_, MeOH/THF (2:1), 0 to 15 °C, 2 h, 69% for **11** (dr = 4:1); (f) MOMCl, DIPEA/CH_2_Cl_2_ (1:1), 0 to 25 °C, 20 h, 85%; (g) LiAlH_4_, THF, 0 °C, 3 h, 92%; (h) TBSCl, imidazole, CH_2_Cl_2_, 25 °C, 1 h, 94%; (i) Burgess reagent, NaHMDS, THF, 0 °C, 1 h, 84% (**14**:**15** = 6.9:1); (j) TBAF, THF, 0 to 25 °C, 2 d, 99%.

Motivated by the need for a more practical and stereoselective approach, we embarked on developing an alternative pathway starting from (*R*)-limonene (**6**) (Scheme 2). Epoxidation of **6** by *m-*CPBA,^33^ oxidative cleavage of the resultant epoxide by NaIO_4_,^34^ and subsequent aldol condensation^35^ gave the known α,β-unsaturated aldehyde **17**.^35^ The next phase involved the diastereoselective epoxidation of **17** using *t-*BuOOH/NaOH (69%, dr = 11:1), the Pinnick oxidation leading to the corresponding carboxylic acid, and the formation of the methyl ester, which proceeded seamlessly, culminating in the formation of α,β-epoxy ester **19**.

**Scheme 2:**
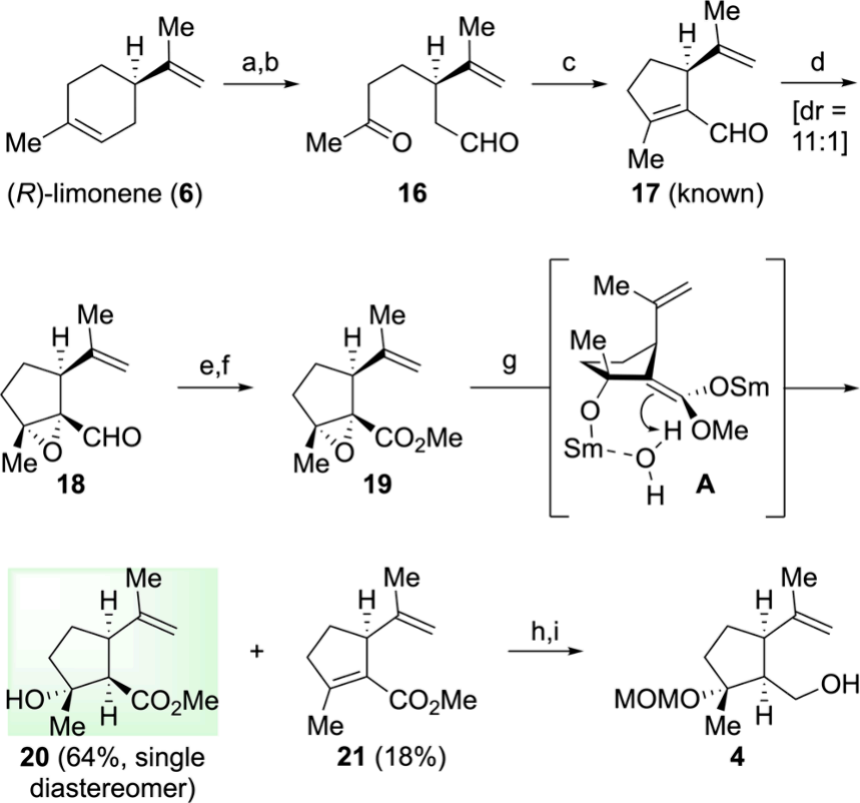
Synthesis of Cyclopentylmethyl Alcohol **4** from (*R*)-Limonene (**6**) via an Epoxide Opening Route^a^

a Reaction conditions: (a) *m-*CPBA, CH_2_Cl_2_, 0 °C, 1 h, 81%; (b) NaIO_4_, THF/H_2_O (2:1), 25 °C, 20 h, 73%; (c) piperidine, HOAc, benzene, reflux, 30 min, 96%; (d) *t-*BuOOH, NaOH, MeOH, 25 °C, 20 h, 69%, dr = 11:1; (e) 2-methyl-2-butene, NaH_2_PO_4_, NaClO_2_, *t-*BuOH/H_2_O (1:1), 25 °C, 4 h; (f) TMSCHN_2_, benzene/MeOH (1:1), 0 to 25 °C, 3 h, 65% for two steps; (g) SmI_2_, THF/H_2_O (9:1), –15 to –10 °C, 1 h, 64%, single diastereomer; (h) MOMCl, DIPEA/CH_2_Cl_2_ (1:1), 0 to 25 °C, 2 d, 85%; (i) LiAlH_4_, THF, 0 °C, 5.5 h, 66% (86% BRSM).

Next, we investigated the SmI_2_-mediated reductive opening^36,37^ of α,β-epoxy ester **19** to prepare β-hydroxy ester **20**. When compound **19** was treated with SmI_2_ in THF/H_2_O under the Paquette’s modified conditions,^37^ the reaction yielded the desired β-hydroxy ester **20** with excellent stereoselectivity (single diastereomer) and a good yield (64%) via a plausible samarium-chelated intermediate **A**, accompanied by the readily separable elimination product **21** (18%). Compound **21** could be recycled to **17** via a simple two-step process.^38^ It is noteworthy that the use of MeOH, as an alternative proton source to H_2_O, afforded the desired β-hydroxy ester **20** as a single isomer in <75% yield, but it was contaminated with inseparable impurities (see the Supporting Information for details). MOM protection and LiAlH_4_-reduction afforded the same cyclopentylmethyl **4**, which was derived by the established procedure commencing from (*R*)-carvone (Scheme 1). The synthesis of **4** from (*R*)-limonene was accomplished in 9 steps with an overall yield of 9% (12% BRSM). To the best of our knowledge, this may be the first example of the use of limonene as a building block in the synthesis of guaianolides.

In summary, we developed two independent routes to synthesize the appropriately functionalized cyclopentylmethyl alcohol **4**, both comparable in terms of step count and overall yield. However, we opted to proceed with the epoxide opening route starting from (*R*)-limonene (Scheme 2) because it proved to be more practical, given the limitations associated with the hydration route (Scheme 1).

With the key intermediate **4** in hand, we directed our efforts toward constructing the γ-lactone moiety of 4α,9α,10α-trihydroxyguaia-11(13)­en-12,6α-olide (**1**). The installation of the γ-lactone moiety began with the Dess–Martin oxidation of primary alcohol **4** to the corresponding aldehyde followed by subsequent stereoselective addition of methyl lithiopropiolate to afford (6*R*)-alcohol **3** with excellent yield and stereoselectivity (89% for two steps, single diastereomer) (Scheme 3). The excellent diastereoselectivity observed in the addition of methyl lithiopropiolate to the aldehyde can be rationalized by the chelation-controlled model (**B**) shown in Scheme 3. According to this model, nucleophilic addition from the *si* face avoids steric clashes with the axial β-methyl group, resulting in the formation of (6*R*)-alcohol **3**.^39^ The absolute configuration of C6 was established by the Mosher ester analysis^40–42^ (see the Supporting Information for details). The partial reduction of alkyne **3** to the corresponding *cis*-alkene by the Lindlar catalyst followed by an acid-catalyzed lactonization yielded the desired γ-lactone **22** in an excellent yield (91% for two steps). The implementation of an allyl cuprate addition further embellished the lactone, achieving a synthesis of the β-substituted lactone **2** as a single stereoisomer (86%). The 6,7-*trans* stereochemistry of lactone **2** was conclusively determined by the X-ray crystal structure of one of the subsequent intermediates (*vide infra*). The final stage of the synthesis, involving ring-closing metathesis, was successfully completed using the Grubbs second-generation catalyst, thereby finalizing the 6,12-guaianolide framework.

**Scheme 3:**
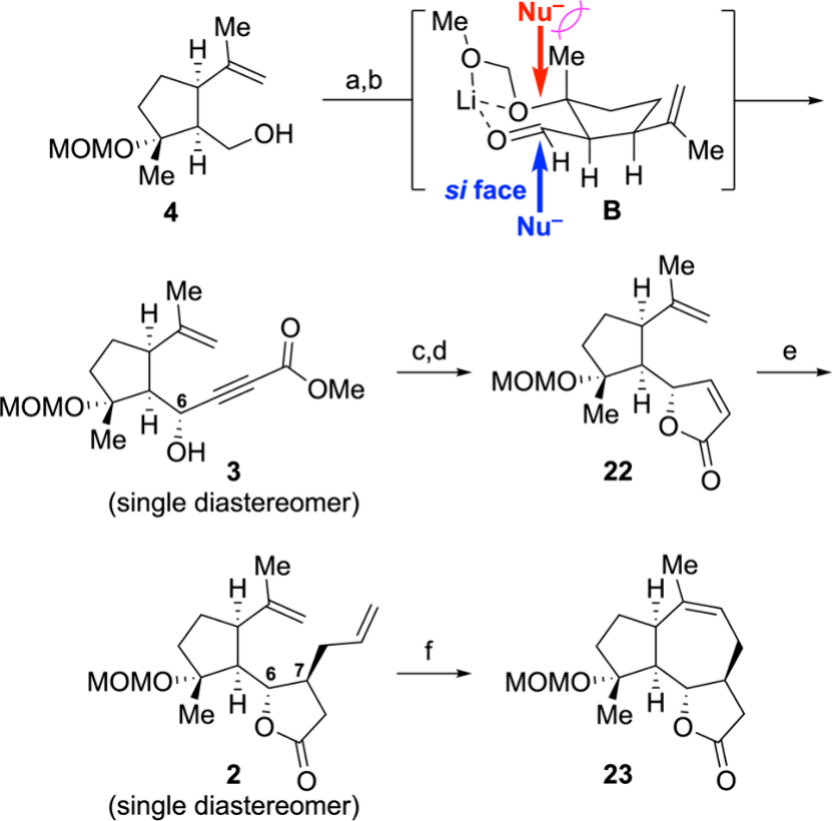
Synthesis of the 6,12-Guaianolide Core of **1** via an Addition of Methyl Lithiopropiolate and Allyl Cuprate Route^a^

a Reaction conditions: (a) Dess–Martin periodinane, CH_2_Cl_2_, 0 to 25 °C, 1 h; (b) methyl propiolate, LiHMDS, THF, –78 °C, 45 min, 89% for two steps, single diastereomer; (c) H_2_, Lindlar catalyst, quinoline, EtOAc, 25 °C, 8 h; (d) PPTS, toluene, 40 °C, 5 h, 91% for two steps; (e) CuBr·DMS, allylmagnesium bromide, TMSCl, THF, –78 °C, 30 min, 86%, single diastereomer; (f) Grubbs second-generation catalyst, CH_2_Cl_2_, reflux, 1 h, 99%.

In pursuit of a more direct approach to the formation of the 6,12-guaianolide framework, we explored the tandem hydroallylation/cyclization reaction for the synthesis of the β-allyl γ-lactone intermediate **24a** (Scheme 4). The tandem hydroallylation/cyclization reaction was originally devised by Yamamoto and co-workers for the efficient synthesis of β-allylbutenolides and proceeded with good regioselectivity and yield.^43^ In our initial investigation, we attempted the tandem reaction with epoxide **25** (see the Supporting Information for the preparation of **25**). The reaction proceeded smoothly, affording the desired β-allyl lactone product **26a** with excellent regioselectivity (β-allyl product:α-allyl product = 9.4:1). However, all attempts for regioselective epoxide opening to install the C4-tertiary hydroxyl group were unsuccessful, leading us to abandon the epoxide substrate. Next, we subjected the MOM-protected alcohol substrate **3** to the same tandem reaction conditions. Unfortunately, this substrate exhibited only moderate regioselectivity in the allyl addition step (β-allyl product:α-allyl product = 1.3:1), which was surprising and disappointing.

**Scheme 4:**
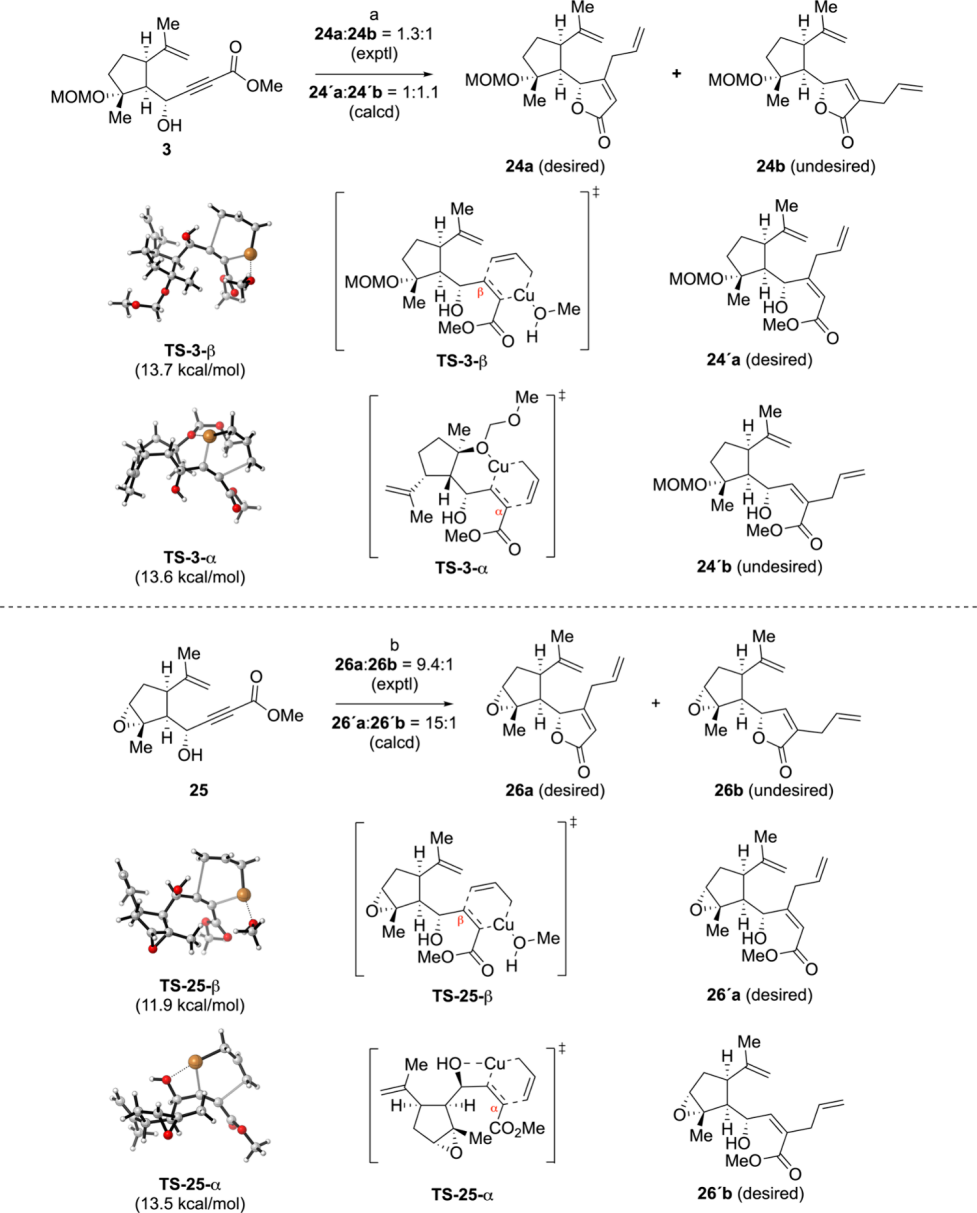
Synthesis of the β-Allyl γ-Lactone Intermediate **24a** via a Tandem Hydroallylation/Cyclization Route and DFT Calculation Results^a^

a Reaction conditions: (a) allylboronic acid pinacol ester, Cu­(OAc)_2_, MeOH, 25 °C, 3 h, 85% (**24a**:**24b** = 1.3:1); (b) allylboronic acid pinacol ester, Cu­(OAc)_2_, MeOH, 25 °C, 20 h, 83% (**26a**:**26b** = 9.4:1).

To understand the origin of the moderate regioselectivity observed in the tandem reaction of MOM-protected alcohol substrate **3**, we performed density functional theory (DFT) calculations (Scheme 4). We identified transition state (TS) structures leading to β- and α-allyl addition products of **3**, namely, **TS-3-β** and **TS-3-α**, respectively. In the TS of β-allylation (**TS-3-β**), a solvent molecule (MeOH) coordinates the metal center. Interestingly, in the TS of α-allylation (**TS-3-α**), the MOM-protected oxygen atom coordinates the copper center, constructing an intramolecular 6-membered metallacycle. The computed activation energies were 13.7 kcal/mol for **TS-3-β** and 13.6 kcal/mol for **TS-3-α**, which explains the observed regioselectivity [**24’a**:**24’b** = 1:1.1 (calcd) vs **24a**:**24b** = 1.3:1 (exptl)].

Geometric restriction present in epoxide substrate **25** explains the observed high regioselectivity. Here, the computed activation energy of β-allylation is 1.6 kcal/mol lower in energy than that of α-allylation: 11.9 kcal/mol for **TS-25-β** vs 13.5 kcal/mol for **TS-25-α**. This finding is in good agreement with the observed regioselectivity [**26’a**:**26’b** = 15:1 (calcd) vs **26a**:**26b** = 9.4:1 (exptl)]. Due to the geometric restriction of the epoxy oxygen, the oxygen of the unprotected hydroxyl group coordinates the Cu-center in the TS of α-allylation (**TS-25-α**), leading to the formation of a four-membered metallacycle. Consequently, a higher strain in **TS-25-α** impedes the formation of α-allylation product. We anticipate that these DFT calculation results will aid in the design of improved γ-hydroxybutynoate substrates for tandem hydroallylation/cyclization reactions.

The synthesis advanced with OsO_4_-dihydroxylation, which proceeded to give a single diastereomer in 91% yield, leading to mono-MOM protected triol **27** (Scheme 5). The Flack parameter obtained from X-ray crystallography (see the Supporting Information for details) confirmed the absolute configuration of C9 and C10 as *R* and *S*, respectively.^44^ Subsequent 1,2-acetonide protection of diol **27** was achieved using 2,2-dimethoxypropane, resulting in the formation of acetonide **28**. The introduction of the *exo*-methylene group was accomplished by treating **28** with Eschenmoser’s salt, followed by an oxidation/elimination process.^45^ The final step entailed a global deprotection using TFA, which furnished 4α,9α,10α-trihydroxyguaia-11(13)­en-12,6α-olide (**1**), concluding the synthetic sequence in 20 steps with an overall yield of 4%. The synthetic **1** was confirmed to be indistinguishable from the authentic natural product (see the Supporting Information for details), except for its optical rotation (synthetic **1**: [α]_*D*_^24^ –14.9°, *c* = 0.1 in MeOH; the optical rotation reported in ref 17: [α]_*D*_^25^ +72°, *c* = 0.1 in MeOH). The optical rotation sign of synthetic compound **1** was opposite to that of the natural product; however, the significant difference in magnitude complicated matters, making it difficult to definitively conclude that they are enantiomers. To date, only guaianolide sesquiterpenoids with a C7–Hα configuration have been reported in the literature.^6,46,47^ Should the tentative absolute stereochemical assignment of the natural 4,9,10-trihydroxyguaia-11(13)­en-12,6-olide isolated by Perveen et al. be confirmed, it could be among the first reported guaianolide sesquiterpenoids with a C7–Hβ configuration.

**Scheme 5:**
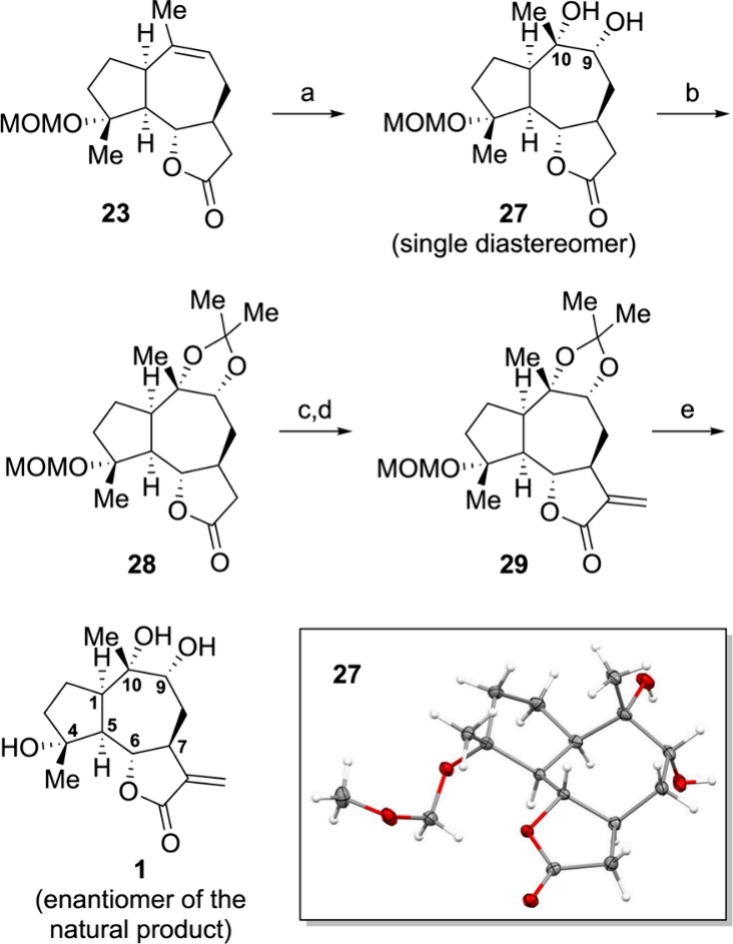
Completion of the Synthesis of 4α,9α,10α-Trihydroxyguaia-11(13)­en-12,6α-olide (**1**)­^a^

a Reaction conditions: (a) OsO_4_, NMO, acetone/H_2_O (1:1), 0 to 25 °C, 20 h, 91%, single diastereomer; (b) 2,2-dimethoxypropane, PPTS, CH_2_Cl_2_, 25 °C, 5 h, quant.; (c) Eschenmoser’s salt, LiHMDS, THF, –78 °C, 1.5 h; (d) *m-*CPBA, NaHCO_3_, CH_2_Cl_2_, 0 to 25 °C, 1 h, 67% for two steps; (e) TFA, MeOH, CH_2_Cl_2_, 0 °C, 2.5 h, quant.

To investigate the biological potential of 4α,9α,10α-trihydroxyguaia-11(13)­en-12,6α-olide (**1**), we assessed its antifungal activity against . Although the previous study reported significant antifungal activity for the natural product (0.21 ± 0.04 μg/mL),^17^ our evaluation of compound **1**, the enantiomer of the natural product, showed no notable antifungal activity even at a concentration of 50 μg/mL (see the Supporting Information for details). This result suggests that the absolute stereochemistry of the natural product might be crucial for its antifungal activity.

The α-*exo*-methylene γ-lactone moiety of **1** is a prominent structural motif in biologically important natural products,^48–50^ known for its role in anticancer natural products, which prompted us to assess its anticancer capabilities. We utilized the Profiling Relative Inhibition Simultaneously in Mixtures (PRISM) technology, a high-throughput screening method that employs molecular barcoding to analyze compounds against a wide array of human cancer cell line models.^51,52^ Through this system, which tags cancer cell lines with distinct DNA sequences for pooled screening, we examined over 578 adherent cell lines across 24 tumor types.

We are thrilled to report that (−)-4α,9α,10α-trihydroxyguaia-11(13)­en-12,6α-olide (**1**) showed promising anticancer properties, in particular against BT12, UM-UC-6, and CHL-1 cell lines (Table 1). Among the cancer cells tested, compound **1** showed strong proliferation inhibition activity against BT12 cells. BT12 cells are a human cancer cell line derived from a tumor in the posterior fossa of the brain. These cells are of particular interest because they represent a specific type of rare and aggressive childhood cancer known as an atypical teratoid rhabdoid tumor (ATRT).^53^ ATRT is a rare and aggressive tumor that occurs most often in children aged 3 and younger. ATRT can appear in various parts of the CNS but is often found in the cerebellum, which controls balance and movement, or the brainstem, which regulates basic body functions like breathing and heart rate. Treatment options for ATRTs usually involve surgery to remove as much of the tumor as possible, followed by chemotherapy and possibly radiation therapy. However, their clinical efficacy has been limited.^54^ Therefore, there is an urgent need for developing more efficacious and safer treatment options for ATRTs. When we tested the anticancer potential of compound **1** using PRISM, it showed an IC_50_ value of 6.1 μM and an EC_50_ value of 5.0 μM. This potency is comparable to that of cisplatin (IC_50_: 4.7 ± 1.1 μM) and slightly better than etoposide (IC_50_: 9.2 ± 0.7 μM) for BT12 ATRT cells.^55^ Compound **1** also showed IC_50_ values of 16.4 and 18.2 μM against urothelial cell carcinoma (UM-UC-6 cells) and melanoma (CHL-1 cells), respectively. In contrast, treatment with **1** resulted in over 55% survival of noncancerous cell lines, such as MMNK1 (immortalized cholangiocyte) and HS729 (fibroblast), even at a concentration of 50 μM. Motivated by the notable efficacy of compound **1** in combating tumors, we plan to further investigate its therapeutic potential in various cancer types. This will involve the design, creation, and evaluation of various analogs of **1**. Additionally, we aim to create a chemical probe based on **1** which will be useful in determining its molecular targets and understanding how it functions.

**Table 1:** Representative Anticancer Activity of (−)-4α,9α,10α-Trihydroxyguaia-11(13)­en-12,6α-olide (**1**)

Cell lines	IC_50_ (μM)	EC_50_ (μM)
BT12 (atypical teratoid rhabdoid tumor)	6.1	5.0
UM-UC-6 (urothelial cell carcinoma)	16.4	16.0
CHL-1 (melanoma)	18.2	17.9

## Conclusions

In summary, this work reports the asymmetric total synthesis and initial assessment of the anticancer potential of (−)-4α,9α,10α-trihydroxyguaia-11(13)­en-12,6α-olide (**1**). Starting from (*R*)-limonene, the synthesis was accomplished in 20 steps with an overall yield of 4%. It incorporated stereoselective reductive epoxide opening and stereoselective additions of methyl lithiopropiolate and allyl cuprate. Preliminary biological studies revealed that while compound **1** lacks antifungal activity, it demonstrates promising anticancer activity against brain, bladder, and skin cancer cell lines. We anticipate that the efficient synthetic route developed for **1** will facilitate the generation of derivatives for structure–activity relationship studies, the optimization of potency and selectivity, and the development of chemical probes to identify molecular targets and mechanisms of action.

## Supplementary Material









## Notes

Detailed descriptions of all synthetic procedures (PDF)
NMR spectra (PDF)
Computational details (PDF)
X-ray crystallography data (PDF)
