# Supplementary material for: Asymmetric Total Synthesis of 4,9,10-Trihydroxyguaia-11(13)en-12,6-olide and Discovery of Its Anticancer Activity against Atypical Teratoid Rhabdoid Tumor
Source: ACS Cent Sci. 2025 Jun 3;11(7):1103–10. doi: 10.1021/acscentsci.5c00332 (PMC12291133; doi:10.1021/acscentsci.5c00332)
Supplement: Supplementary file 1 [file oc5c00332_si_001.pdf]

–Supporting Information: Part A–

Asymmetric Total Synthesis of 4,9,10-Trihydroxyguaia-11(13)en-12,6-olide and Discovery of Its Anticancer Activity Against Atypical Teratoid Rhabdoid Tumor

Hyejin Lee[1], Hongjun Jang[1,2], Hwan Myung[3,4], Angela Rivera[5], Anna F. Averette[5], Joseph Heitman[5,6], Jiyong Park[4,3], Deukjoon Kim[7], Hyoungsu Kim[2], and Jiyong Hong[1,6,*]

[1] Department of Chemistry, Duke University, Durham, North Carolina 27708, United States

[2] College of Pharmacy and Research Institute of Pharmaceutical Science and Technology (RIPST), Ajou University, Suwon 16499, Republic of Korea

[3] Department of Chemistry, Korea Advanced Institute of Science and Technology (KAIST), Daejeon 34141, Republic of Korea

[4] Center for Catalytic Hydrocarbon Functionalizations, Institute for Basic Science (IBS), Daejeon 34141, Republic of Korea

[5] Department of Molecular Genetics and Microbiology, Duke University School of Medicine, Durham, North Carolina 27710, United States

[6] Department of Pharmacology and Cancer Biology, Duke University School of Medicine, Durham, North Carolina 27710, United States

[7] College of Pharmacy, Seoul National University, Seoul 08826, Republic of Korea

1. General Methods S2
2. Synthetic Procedure and Analytical Data S3
3. Table S1. Mukaiyama Hydration of Bicyclic Lactone **10** S29
4. Table S2. Dehydration of Mono TBS-Ether **13** S30
5. Table S3. SmI_2-Mediated Reductive Opening of α,β-Epoxy Ester **19** S31
6. Mosher Ester Analysis of Alcohol **3** S32
7. Antifungal Activity of **1** S35
8. Comparison of Spectral Data of the Natural Product and Synthetic **1** S39
9. References S41

1. General Methods

All reactions were conducted in oven-dried glassware under nitrogen. Unless otherwise stated, all reagents were purchased from commercial suppliers (Sigma–Aldrich, Acros, Fisher, Alfa Aesar, TCI, or Ambeed) and used without further purification. All solvents were American Chemical Society (ACS) grade or better and used without further purification. Analytical thin layer chromatography (TLC) was performed with glass-backed silica gel (60 Å) plates with fluorescent indication (Whatman). Visualization was accomplished by UV irradiation at 254 nm and/or by staining with *p*-anisaldehyde solution or potassium permanganate solution followed by heating. Flash column chromatography was performed by using silica gel (particle size 230−400 mesh, 60 Å) purchased from Silicycle. All ^{1}H NMR and ^{13}C NMR spectra were recorded with a Varian 400 (400 MHz) and a Bruker 500 (500 MHz) spectrometer. All NMR δ values are given in parts per million (ppm) and are referenced to the residual isotopomer solvent signals ($CDCl_3$: δ = 7.26 ppm, CD_3OD: δ = 3.31 ppm, $(CD_3)_2CO$: δ = 2.05 ppm) for ^{1}H NMR spectra, or the solvent signals ($CDCl_3$: δ = 77.16 ppm, CD_3OD: δ = 49.00 ppm, $(CD_3)_2CO$: δ = 29.84 ppm) for ^{13}C NMR spectra. Coupling constants (*J*) are given in Hertz (Hz) and multiplicities are indicated using the conventional abbreviation (s = singlet, d = doublet, t = triplet, q = quartet, quint = quintet, m = multiplet or overlap of non-equivalent resonances, br = broad). Electrospray ionization (ESI) mass spectrometry (MS) was recorded with an Agilent 6224 series (LC/MS–TOF) spectrometer to obtain the molecular masses of the compounds. Optical rotation values were measured with a Rudolph Research Analytical (A21102 API/1W) polarimeter. Single-crystal X-ray diffraction data were collected on a Bruker D8 VENTURE diffractometer.

2. Synthetic Procedure and Analytical Data

2.1. (*R*)-Carvone Route

Preparation of Bicyclic Lactone 10

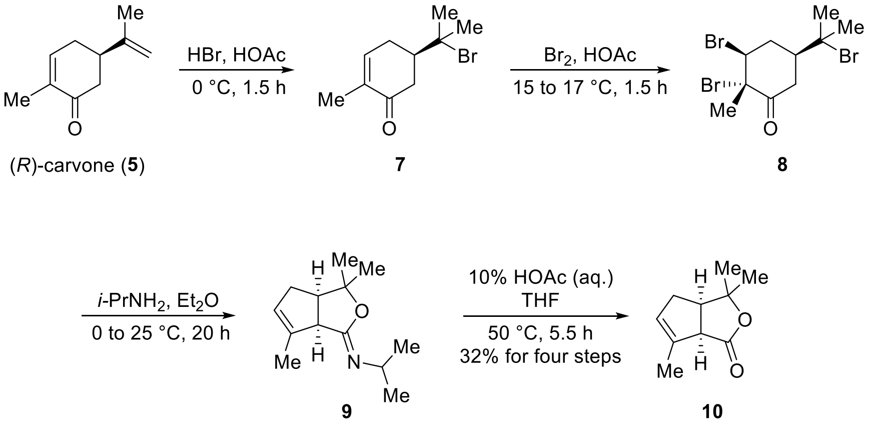

[*Hydrobromination*] To a cooled (0 °C) solution of 33% HBr in HOAc (2.2 mL, 13 mmol) was added a solution of (*R*)-carvone (1.04 mL, 6.7 mmol) in HOAc (1 mL) dropwise. After stirring at 0 °C for 1.5 h, the reaction was quenched with an addition of ice H_2O, and the resulting mixture was diluted with EtOAc. The layers were separated, and the aqueous layer was extracted with EtOAc. The combined organic layers were washed with saturated aqueous $NaHCO_3$ and brine, dried over anhydrous Na_2SO_4, and concentrated *in vacuo* to afford α,β-unsaturated ketone **7** as an amber oil. **7** was used in the following step without further purification. For **7**: ^{1}H NMR (500 MHz, $CDCl_3$) δ 6.75 (dq, *J* = 5.1, 1.5 Hz, 1H), 2.74 (ddd, *J* = 16.0, 3.8, 1.9 Hz, 1H), 2.59 (dtq, *J* = 18.5, 4.7, 1.4 Hz, 1H), 2.46–2.36 (m, 2H), 2.10–2.01 (m, 1H), 1.79 (s, 6H), 1.77 (s, 3H); ^{13}C NMR (125 MHz, $CDCl_3$) δ 199.2, 144.3, 135.4, 70.1, 48.3, 41.2, 32.4, 32.2, 29.1, 15.7; [*Bromination*] To a cooled (15 to 17 °C) solution of the above crude **7** in HOAc (4.4 mL) was added a solution of Br_2 (0.41 mL, 8.0 mmol) in HOAc (0.7 mL) dropwise over 20 min. After stirring at the same temperature for 1.5 h, the reaction was quenched with an addition of ice H_2O, and the resulting mixture was diluted with Et_2O. The layers were separated, and the aqueous layer was extracted with Et_2O. The combined organic layers were washed with H_2O, saturated aqueous $NaHCO_3$ and brine, dried over anhydrous Na_2SO_4, and concentrated *in vacuo* to afford α,β-dibromo ketone **8** as an amber oil. **8** was used in the following step without further purification. For **8**: ^{1}H NMR (500 MHz, $CDCl_3$) δ 4.83 (t, *J* = 3.0 Hz, 1H), 3.35 (dd, *J* = 15.0, 12.6 Hz, 1H), 2.98 (ddd, *J* = 14.5, 11.7, 2.8 Hz, 1H), 2.68 (ddd, *J* = 15.0, 4.6, 2.3 Hz, 1H), 2.44 (dq, *J* = 14.6, 3.1 Hz, 1H), 2.42–2.34 (m,

1H), 2.00 (s, 3H), 1.82 (s, 3H), 1.78 (s, 3H); ^{13}C NMR (125 MHz, $CDCl_3$) δ 201.0, 69.7, 62.2, 58.9, 46.2, 38.5, 33.9, 32.8, 32.6, 27.9; [*Favorskii Rearrangement*] To a cooled (0 °C) solution of the above crude **8** in Et_2O (27 mL) was added isopropyl amine (6.4 mL, 74 mmol) dropwise. After stirring at 0 °C for 10 min, the reaction mixture was warmed to 25 °C and stirred for 20 h. The reaction mixture was cooled to 0 °C before an addition of 10% aqueous H_2SO_4. The layers were separated, and the organic layer was extracted with 10% H_2SO_4. The combined aqueous layers were cooled to 0 °C, basified to pH 8.0 with 10 N NaOH and extracted with EtOAc. The combined organic layers were washed brine, dried over anhydrous Na_2SO_4 and concentrated *in vacuo* to afford imidate **9** as an amber oil. **9** was used in the following step without further purification. For **9**: ^1H NMR (500 MHz, $CDCl_3$) δ 5.33 (s, 1H), 3.79 (dt, J = 12.9, 6.3 Hz, 1H), 3.68–3.55 (m, 1H), 2.79 (td, J = 8.3, 2.8 Hz, 1H), 2.46–2.30 (m, 2H), 1.86 (s, 3H), 1.36 (s, 3H), 1.27 (s, 3H), 1.12 (d, J = 6.4 Hz, 3H), 1.07 (d, J = 6.4 Hz, 3H); ^{13}C NMR (125 MHz, $CDCl_3$) δ 160.9, 139.4, 125.2, 86.3, 57.3, 48.6, 47.1, 33.5, 30.8, 24.2, 23.9, 23.7, 14.8; [*Hydrolysis*] A solution of the above crude **9** in THF/10% aqueous HOAc (3:1, 8 mL) was heated to 50 °C and stirred for 5.5 h. After cooling to 0 °C, the reaction was quenched with an addition of ice H_2O and saturated aqueous $NaHCO_3$, and the resulting mixture was diluted with EtOAc. The layers were separated, and the aqueous layer was extracted with EtOAc. The combined organic layers were dried over anhydrous Na_2SO_4 and concentrated *in vacuo*. The residue was purified by column chromatography (silica gel, hexanes/EtOAc, 9:1) to afford bicyclic lactone **10**[1,2] (350 mg, 32% for four steps) as a brown solid: $[\alpha]_D^{24}$ +142.9° (*c* 1.0, Et_2O); ^1H NMR (500 MHz, $CDCl_3$) δ 5.41 (s, 1H), 3.58 (d, J = 9.0 Hz, 1H), 2.96 (ddd, J = 9.1, 7.0, 5.4 Hz, 1H), 2.51–2.44 (m, 2H), 1.88 (s, 3H), 1.45 (s, 3H), 1.36 (s, 3H); ^{13}C NMR (125 MHz, CDCl3) δ 176.0, 136.5, 126.7, 86.0, 56.8, 48.6, 33.8, 31.0, 24.1, 14.8.

Preparation of α-Tertiary Alcohol 11

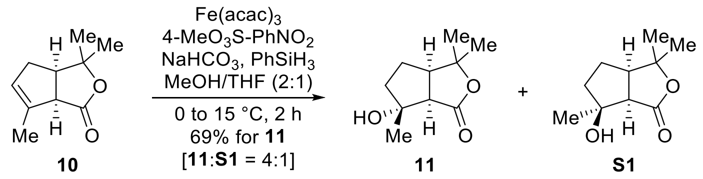

To a cooled solution (0 °C) of **10** (88 mg, 0.53 mmol), $Fe(acac)_3$ (5.6 mg, 0.016 mmol), 4-MeO_3S-$PhNO_2$ (149 mg, 0.69 mmol), and $NaHCO_3$ (89 mg, 1.1 mmol) in anhydrous MeOH/THF (2:1, 2.1 mL) was added $PhSiH_3$ (0.2 mL, 1.6 mmol) dropwise. The resulting mixture was allowed to stir

with gradual warming to 15 °C over 2 h and concentrated *in vacuo*. The residue was purified by column chromatography (silica gel, CH_2Cl_2/EtOAc, 4:1) to afford α-tertiary alcohol **11** (67 mg, 69%, **11**:**S1** = 4:1 as determined by ^{1}H NMR) as a white solid.

For α-alcohol **11**: white solid; ^{1}H NMR (500 MHz, $CDCl_3$) δ 3.05 (dd, J = 8.5, 1.4 Hz, 1H), 2.87 (td, J = 9.1, 4.6 Hz, 1H), 2.02 (dtd, J = 13.7, 9.8, 7.3 Hz, 1H), 1.84 (ddt, J = 13.7, 8.9, 4.3 Hz, 1H), 1.76 (dddd, J = 13.0, 7.4, 4.0, 1.5 Hz, 1H), 1.63 (ddd, J = 13.2, 10.1, 8.0 Hz, 1H), 1.59 (s, 3H), 1.41 (s, 3H), 1.39 (s, 3H); ^{13}C NMR (125 MHz, $CDCl_3$) δ 175.7, 84.4, 81.7, 58.1, 49.0, 40.9, 30.9, 25.4, 24.7, 24.1; HRMS (ESI) *m/z* 207.0990 [(M + Na)$^+$ calcd for $C_{10}H_{16}O_3$ 207.0992].

For β-alcohol **S1**: white solid; ^{1}H NMR (500 MHz, $CDCl_3$) δ 3.09 (br s, 1H), 3.04 (d, J = 9.5 Hz, 1H), 2.67 (q, J = 9.0 Hz, 1H), 2.02–1.91 (m, 1H), 1.87 (ddd, J = 12.7, 6.6, 3.7 Hz, 1H), 1.79–1.70 (m, 1H), 1.57 (ddd, J = 12.6, 10.5, 6.2 Hz, 1H), 1.47 (s, 3H), 1.42 (s, 3H), 1.41 (s, 3H); ^{13}C NMR (125 MHz, $CDCl_3$) δ 177.3, 85.1, 79.1, 56.6, 50.2, 41.6, 30.5, 28.8, 26.0, 23.6; HRMS (ESI) *m/z* 207.0990 [(M + Na)$^+$ calcd for $C_{10}H_{16}O_3$ 207.0992].

Preparation of (4*R*)-MOM-Ether 12

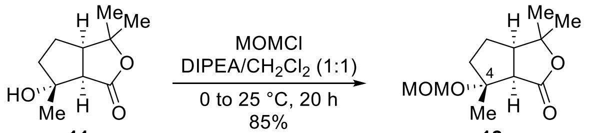

To a cooled solution (0 °C) of **11** (61 mg, 0.33 mmol) in DIPEA/CH_2Cl_2 (1:1, 2 mL) was added MOMCl (0.25 mL, 3.3 mmol). The reaction mixture was warmed to 25 °C and stirred for 20 h. The reaction was quenched with an addition of saturated aqueous $NaHCO_3$, and the resulting mixture was diluted with EtOAc. The layers were separated, and the aqueous layer was extracted with EtOAc. The combined organic layers were dried over anhydrous Na_2SO_4 and concentrated *in vacuo*. The residue was purified by column chromatography (silica gel, hexanes/EtOAc, 9:1) to afford (4*R*)-MOM-ether **12** (64 mg, 85%) as a colorless oil: ^{1}H NMR (500 MHz, $CDCl_3$) δ 4.75 (AB, J_{AB} = 7.3 Hz, $\Delta\nu_{AB}$ = 31.43 Hz, 2H), 3.38 (s, 3H), 3.31 (d, J = 8.5 Hz, 1H), 2.81 (td, J = 9.0, 5.3 Hz, 1H), 2.02–1.90 (m, 2H), 1.83–1.74 (m, 1H), 1.62–1.50 (m, 4H), 1.40 (s, 3H), 1.39 (s, 3H); ^{13}C NMR (125 MHz, $CDCl_3$) δ 175.5, 91.7, 86.7, 84.1, 56.0, 55.6, 49.2, 39.4, 30.7, 25.2, 24.0, 20.5; HRMS (ESI) *m/z* 251.1254 [(M + Na)$^+$ calcd for $C_{12}H_{20}O_4$ 251.1254].

Preparation of (4*S*)-MOM-Ether S2

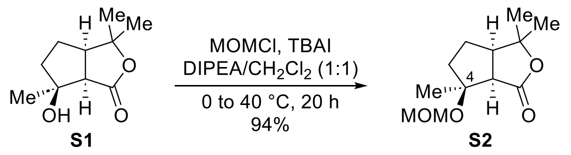

To a cooled solution (0 °C) of **S1** (6 mg, 0.033 mmol) in DIPEA/CH_2Cl_2 (1:1, 0.2 mL) were added MOMCl (15 µL, 0.20 mmol) and TBAI (3.6 mg, 0.0098 mmol). The reaction mixture was warmed to 40 °C and stirred for 20 h. The reaction was quenched with an addition of saturated aqueous $NaHCO_3$, and the resulting mixture was diluted with EtOAc. The layers were separated, and the aqueous layer was extracted with EtOAc. The combined organic layers were dried over anhydrous Na_2SO_4 and concentrated *in vacuo*. The residue was purified by column chromatography (silica gel, hexanes/EtOAc, 3:1) to afford (4*S*)-MOM-ether **S2** (7 mg, 94%) as a colorless oil: ^{1}H NMR (500 MHz, $CDCl_3$) δ 4.73 (AB, J_{AB} = 7.6 Hz, $\Delta\nu_{AB}$ = 52.13 Hz, 2H), 3.37 (s, 3H), 3.01 (d, *J* = 11.1 Hz, 1H), 2.75 (dt, *J* = 11.1, 8.7 Hz, 1H), 2.13 (ddd, *J* = 13.1, 6.3, 1.9 Hz, 1H), 2.00 (tdd, *J* = 12.4, 8.5, 6.3 Hz, 1H), 1.76 (dddd, *J* = 12.8, 8.9, 7.0, 1.9 Hz, 1H), 1.55 (s, 3H), 1.49 (td, *J* = 12.6, 7.1 Hz, 1H), 1.41 (s, 6H).

Preparation of Diol S3

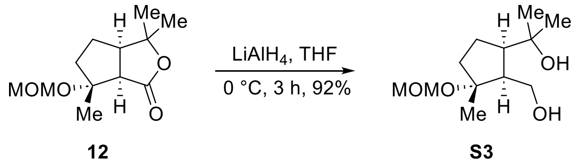

To a cooled solution (0 °C) of **12** (33 mg, 0.15 mmol) in anhydrous THF (1.4 mL) was added $LiAlH_4$ (2.0 M in THF, 0.145 mL, 0.29 mmol) dropwise. After stirring at the same temperature for 3 h, the reaction was quenched with an addition of 15% NaOH solution. The resulting mixture was diluted with Et_2O and treated with Rochelle salt. The layers were separated, and the aqueous layer was extracted with CH_2Cl_2. The combined organic layers were dried over anhydrous Na_2SO_4 and concentrated *in vacuo*. The residue was purified by column chromatography (silica gel, hexanes/CH_2Cl_2/EtOAc, 1:1:2) to afford diol **S3** (31 mg, 92%) as a colorless oil, which was solidified upon standing: ^{1}H NMR (500 MHz, $CDCl_3$) δ 4.69 (AB, J_{AB} = 7.2 Hz, $\Delta\nu_{AB}$ = 8.33 Hz, 2H), 3.89 (dd, *J* = 12.2, 3.2 Hz, 1H), 3.60 (dd, *J* = 12.2, 5.8 Hz, 1H), 3.36 (s, 3H), 2.64 (br s, 2H), 2.59–2.50 (m, 1H), 2.13 (ddt, *J* = 9.5, 6.0, 1.6 Hz, 1H), 1.99–1.89 (m, 1H), 1.88–1.70 (m, 3H), 1.42 (s, 3H), 1.33 (s, 3H), 1.30 (s, 3H); ^{13}C NMR (125 MHz, $CDCl_3$) δ 91.3, 88.1, 72.5, 60.3, 55.5,

51.5, 50.4, 36.6, 31.4, 29.8, 23.5, 21.1; HRMS (ESI) *m/z* 255.1567 [$(M + Na)^+$ calcd for $C_{12}H_{24}O_4$ 255.1567].

Preparation of Mono TBS-Ether 13

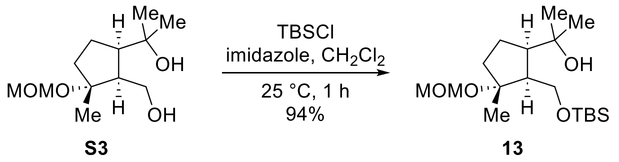

To a solution of **S3** (20 mg, 0.086 mmol) in CH_2Cl_2 (1 mL) were added TBSCl (26 mg, 0.17 mmol) and imidazole (23 mg, 0.34 mmol) at 25 °C. After stirring at 25 °C for 1 h, the reaction was quenched with an addition of saturated aqueous $NaHCO_3$, and the resulting mixture was diluted with EtOAc. The layers were separated, and the aqueous layer was extracted with EtOAc. The combined organic layers were dried over anhydrous Na_2SO_4 and concentrated *in vacuo*. The residue was purified by column chromatography (silica gel, hexanes/EtOAc, 9:1) to afford mono TBS-ether **13** (28 mg, 94%) as a colorless oil: ^1H NMR (500 MHz, $CDCl_3$) δ 4.70 (AB, J_{AB} = 7.3 Hz, $\Delta\nu_{AB}$ = 8.17 Hz, 2H), 4.27 (s, 1H), 3.81 (dd, *J* = 10.8, 3.2 Hz, 1H), 3.67 (dd, *J* = 10.8, 8.0 Hz, 1H), 3.38 (s, 3H), 2.57 (ddd, *J* = 11.3, 9.0, 5.9 Hz, 1H), 2.21 (m, 1H), 1.98–1.90 (m, 1H), 1.90–1.74 (m, 2H), 1.57 (ddd, *J* = 13.9, 11.5, 5.2 Hz, 1H), 1.35 (s, 3H), 1.28 (s, 3H), 1.20 (s, 3H), 0.92 (s, 9H), 0.11 (s, 3H), 0.11 (s, 3H); ^{13}C NMR (125 MHz, $CDCl_3$) δ 91.4, 87.2, 70.6, 61.2, 55.6, 51.33, 51.28, 36.4, 30.4, 29.9, 26.0, 22.8, 21.4, 18.3, -5.4, -5.5; HRMS (ESI) *m/z* 369.2435 [$(M + Na)^+$ calcd for $C_{18}H_{38}O_4Si$ 369.2432].

Preparation of Disubstituted Alkene 14

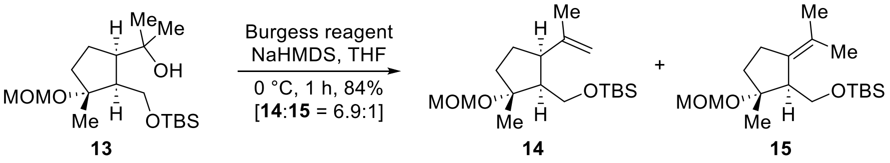

To a cooled (0 °C) solution of **13** (44 mg, 0.13 mmol) in anhydrous THF (1.3 mL) were added NaHMDS (1.0 M in THF, 0.254 mL, 0.25 mmol) and Burgess reagent (45 mg, 0.19 mmol). After stirring at 0 °C for 1 h, the reaction was quenched with an addition of saturated aqueous $NaHCO_3$, and the resulting mixture was diluted with Et_2O. The layers were separated, and the aqueous layer was extracted with EtOAc. The combined organic layers were dried over anhydrous Na_2SO_4 and

concentrated *in vacuo*. The residue was purified by column chromatography (silica gel, hexanes/Et_2O, 70:1) to afford a mixture of disubstituted alkene **14** and tetrasubstituted alkene **15** (35 mg, 84%, **14**:**15** = 6.9:1 as determined by ^{1}H NMR).

For disubstituted alkene **14**: colorless oil; ^{1}H NMR (500 MHz, $CDCl_3$) δ 4.85 (sextet, *J* = 1.4 Hz, 1H), 4.76–4.70 (m, 3H), 3.51 (dd, *J* = 10.8, 3.1 Hz, 1H), 3.38 (s, 3H), 3.38 (dd, *J* = 10.7, 6.0 Hz, 1H), 3.02 (q, *J* = 8.5 Hz, 1H), 2.15–2.08 (m, 1H), 1.98 (dddd, *J* = 12.9, 9.3, 5.1, 1.4 Hz, 1H), 1.90–1.66 (m, 6H), 1.39 (s, 3H), 0.87 (s, 9H), 0.00 (s, 3H), -0.01 (s, 3H); ^{13}C NMR (125 MHz, $(CD_3)_2CO$) δ 146.5, 110.5, 91.9, 88.6, 61.1, 55.4, 52.8, 48.2, 38.0, 26.6, 26.3, 24.0, 21.9, 18.6, -5.4, -5.6; HRMS (ESI) *m/z* 351.2333 $[(M + Na)^+]$ calcd for $C_{18}H_{36}O_3Si$ 351.2326].

For tetrasubstituted alkene **15**: colorless oil; ^{1}H NMR (500 MHz, $CDCl_3$) δ 4.70 (AB, J_{AB} = 7.4 Hz, $\Delta\nu_{AB}$ = 23.25 Hz, 2H), 3.58 (dd, *J* = 10.5, 3.3 Hz, 1H), 3.53 (dd, *J* = 10.5, 6.2 Hz, 1H), 3.30 (s, 3H), 2.72 (br s, 1H), 2.37–2.26 (m, 1H), 2.23–2.13 (m, 1H), 1.90 (ddt, *J* = 13.5, 8.4, 1.7 Hz, 1H), 1.83–1.73 (m, 1H), 1.68 (s, 3H), 1.61 (s, 3H), 1.38 (s, 3H), 0.86 (s, 9H), 0.00 (s, 3H), -0.03 (s, 3H); ^{13}C NMR (125 MHz, $CDCl_3$) δ 136.7, 123.9, 91.0, 87.9, 63.5, 55.1, 54.2, 36.7, 28.3, 26.0, 21.3, 21.1, 20.7, 18.3, -5.46, -5.54; HRMS (ESI) *m/z* 351.2329 $[(M + Na)^+]$ calcd for $C_{18}H_{36}O_3Si$ 351.2326].

Preparation of Primary Alcohol 4

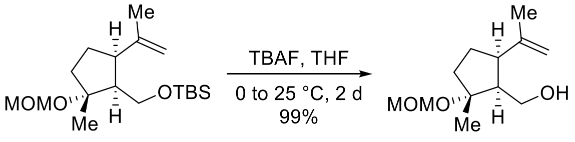

To a cooled (0 °C) solution of **14** (104 mg, 0.32 mmol) in THF (1.5 mL) was added TBAF (1 M in THF, 0.475 mL, 0.48 mmol). After stirring at 0 °C for 5 min, the reaction mixture was warmed to 25 °C. After stirring for 2 d, the reaction was quenched with an addition of saturated aqueous NH_4Cl, and the resulting mixture was diluted with Et_2O. The layers were separated, and the aqueous layer was extracted with EtOAc. The combined organic layers were dried over anhydrous Na_2SO_4 and concentrated *in vacuo*. The residue was purified by column chromatography (silica gel, hexanes/EtOAc, 5:1) to afford primary alcohol **4** (67 mg, 99%) as a colorless oil: ^{1}H NMR (500 MHz, $(CD_3)_2CO$) δ 4.82 (sextet, *J* = 1.4 Hz, 1H), 4.74–4.67 (m, 3H), 3.49 (dt, *J* = 11.3, 3.7 Hz, 1H), 3.35 (ddd, *J* = 11.4, 7.4, 5.7 Hz, 1H), 3.31 (s, 3H), 3.20 (dd, *J* = 5.7, 4.1 Hz, 1H), 3.07–2.99 (m, 1H), 2.21 (td, *J* = 7.3, 3.3 Hz, 1H), 2.02–1.88 (m, 1H), 1.83–1.72 (m, 5H), 1.71–1.62 (m,

1H), 1.40 (s, 3H); ^{13}C NMR (125 MHz, $(CD_3)_2CO$) δ 147.2, 110.3, 91.9, 88.6, 60.1, 55.3, 53.3, 48.0, 37.7, 26.2, 23.8, 22.0; HRMS (ESI) *m*/*z* 237.1460 $[(M + Na)^+$ calcd for $C_{12}H_{22}O_3$ 237.1461].

2.2. (*R*)-Limonene Route

Preparation of Epoxide S4

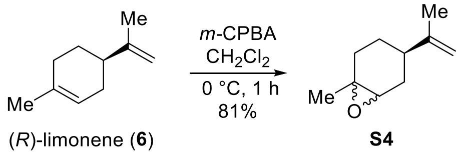

To a cooled solution (0 °C) of (*R*)-limonene (2.38 mL, 15 mmol) in CH_2Cl_2 (15 mL) was added a solution of *m*-CPBA (3.55 g, 21 mmol) in CH_2Cl_2 (35 mL) dropwise. After stirring at 0 °C for 1 h, the reaction was quenched with an addition of saturated aqueous $NaHCO_3$, and the resulting mixture was diluted with CH_2Cl_2. The layers were separated, and the aqueous layer was extracted with CH_2Cl_2. The combined organic layers were dried over anhydrous Na_2SO_4 and concentrated *in vacuo*. The residue was purified by column chromatography (silica gel, hexanes/Et_2O, 30:1) to afford a mixture of diastereomeric epoxides **S4**[3] (1.82 g, 81%) as a colorless oil: ^{1}H NMR (500 MHz, $CDCl_3$) δ 4.73 and 4.67 (s, 4H, trans and cis), 3.08–3.03 (m, 1H, trans) and 2.99 (d, *J* = 5.4 Hz, 1H, cis), 2.18–2.07 (m, 2H, trans), 2.07–1.99 (m, 2H, cis), 1.91–1.78 (m, 3H), 1.75–1.62 (m, 9H), 1.56–1.50 (m, 1H, trans), 1.41–1.34 (m, 2H, cis), 1.32 (s, 3H, cis), 1.31 (s, 3H, trans), 1.26–1.15 (m, 1H, trans).

Preparation of Keto Aldehyde 16

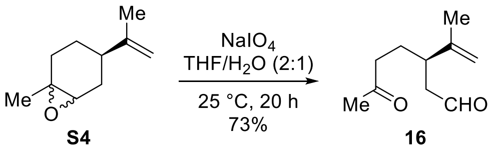

To a solution of **S4** (1.82 g, 12 mmol) in THF/H_2O (2:1, 39 mL) was added $NaIO_4$ (3.83 g, 18 mmol) at 25 °C. After stirring at 25 °C for 20 h, the iodate salts were filtered off and washed with Et_2O, creating two layers. The layers were separated, and the aqueous layer was extracted with CH_2Cl_2. The combined organic layers were dried over anhydrous Na_2SO_4 and concentrated *in vacuo*. The residue was purified by column chromatography (silica gel, hexanes/Et_2O, 2:1) to afford keto aldehyde **16**[4] (1.46 g, 73%) as a colorless oil: ^{1}H NMR (500 MHz, $CDCl_3$) δ 9.68 (t, *J* = 2.3 Hz, 1H), 4.84 (quint, *J* = 1.6 Hz, 1H), 4.78 (d, *J* = 1.7 Hz, 1H), 2.73–2.63 (m, 1H), 2.51–2.41 (m, 2H), 2.39 (t, *J* = 7.4 Hz, 2H), 2.13 (s, 3H), 1.72 (dtd, *J* = 14.0, 7.8, 4.8 Hz, 1H), 1.68–1.57 (m, 4H).

Preparation of α,β-Unsaturated Aldehyde 17

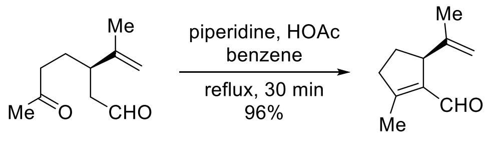

To a solution of **16** (1.46 g, 8.7 mmol) in anhydrous benzene (35 mL) were added piperidine (0.35 mL, 3.5 mmol) and HOAc (0.35 mL, 6.1 mmol) at 25 °C. After refluxing for 30 min, the reaction was quenched with an addition of H_2O, and the resulting mixture was diluted with Et_2O. The layers were separated, and the aqueous layer was extracted with Et_2O. The combined organic layers were dried over anhydrous Na_2SO_4 and concentrated *in vacuo*. The residue was purified by column chromatography (silica gel, hexanes/Et_2O, 30:1) to afford α,β-unsaturated aldehyde **17**[5] (1.26 g, 96%) as a colorless oil: $[\alpha]_D^{24}$ +45.7° (*c* 1.0, $CHCl_3$); ^{1}H NMR (500 MHz, $CDCl_3$) δ 9.98 (s, 1H), 4.71 (quint, *J* = 1.6 Hz, 1H), 4.62 (s, 1H), 3.62 (d, *J* = 9.4 Hz, 1H), 2.68–2.57 (m, 1H), 2.46 (ddd, *J* = 18.8, 9.4, 4.0 Hz, 1H), 2.19 (q, *J* = 1.4 Hz, 3H), 2.10 (dtd, *J* = 13.1, 9.4, 7.5 Hz, 1H), 1.74–1.65 (m, 4H).

Preparation of α,β-Epoxy Aldehyde 18

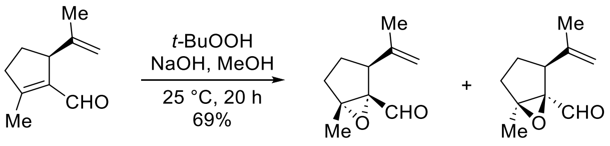

To a solution of **17** (111 mg, 0.74 mmol) in MeOH (1 mL) were added *t*-BuOOH (70 wt. % in H_2O, 0.2 mL, 1.5 mmol) and 1 N NaOH (0.3 mL) at 25 °C. After stirring at 25 °C for 20 h, the reaction was quenched with an addition of H_2O, and the resulting mixture was diluted with Et_2O. The layers were separated, and the aqueous layer was extracted with Et_2O. The combined organic layers were dried over anhydrous Na_2SO_4 and concentrated *in vacuo*. The residue was filtered through a short pad of silica gel (hexanes/Et_2O, 7:1) to afford a mixture of diastereomeric α,β-epoxy aldehydes **18**[6] and **S5** (85 mg, 69%, **18**:**S5** = 11:1 as determined by ^{1}H NMR) as a colorless oil.

For major isomer **18**: ^{1}H NMR (500 MHz, $CDCl_3$, major isomer): δ 9.61 (s, 1H), 4.87 (s, 1H), 4.65 (s, 1H), 3.00 (d, *J* = 8.2 Hz, 1H), 1.99 (dd, *J* = 14.0, 8.5 Hz, 1H), 1.91 (ddd, *J* = 14.0, 10.4, 8.2 Hz,

1H), 1.83–1.69 (m, 4H), 1.57 (s, 3H), 1.42 (dd, J = 12.9, 8.1 Hz, 1H); HRMS (ESI) m/z 167.1064 [(M + H)$^+$ calcd for $C_{10}H_{14}O_2$ 167.1067].

Preparation of α,β-Epoxy Ester 19

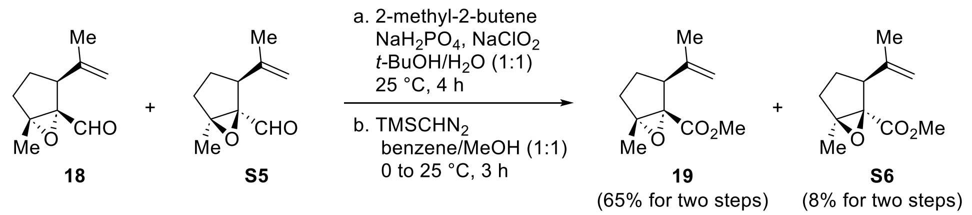

[*Pinnick Oxidation*] To a solution of a mixture **18** and **S5** (11:1, 42.3 mg, 0.26 mmol) in *t*-BuOH/H_2O (1:1, 2 mL) were added 2-methyl-2-butene (1.0 mL, 9.4 mmol), NaH_2PO_4 (153 mg, 1.3 mmol), and $NaClO_2$ (115 mg, 1.3 mmol) at 25 °C. After stirring at 25 °C for 4 h, the reaction mixture was diluted with EtOAc and H_2O. The layers were separated, and the aqueous layer was extracted with EtOAc. The combined organic layers were dried over anhydrous Na_2SO_4 and concentrated *in vacuo* to afford the corresponding carboxylic acid as a colorless oil. The carboxylic acid was used in the following step without further purification; [*Methyl Ester Formation*] To a cooled (0 °C) solution of the crude carboxylic acid in anhydrous benzene/MeOH (1:1, 1.4 mL) was added $TMSCHN_2$ (2.0 M in Et_2O, 0.380 mL, 0.76 mmol). After stirring at 0 °C for 5 min, the reaction mixture was warmed to 25 °C. After stirring for 3 h, the reaction mixture was concentrated *in vacuo*. The residue was purified by column chromatography (silica gel, hexanes/Et_2O, 30:1) to afford the major α,β-epoxy ester **19** (32.3 mg, 65% for two steps) and the minor α,β-epoxy ester **S6** (4.1 mg, 8% for two steps).

For major α,β-epoxy ester **19**: colorless oil; ^{1}H NMR (500 MHz, $CDCl_3$) δ 4.73 (quint, J = 1.6 Hz, 1H), 4.62 (s, 1H), 3.72 (s, 3H), 3.03 (d, J = 8.3 Hz, 1H), 1.93 (dd, J = 14.0, 8.6 Hz, 1H), 1.85 (ddd, J = 14.0, 10.2, 8.2 Hz, 1H), 1.78–1.67 (m, 4H), 1.59 (s, 3H), 1.44–1.35 (m, 1H); ^{13}C NMR (125 MHz, $CDCl_3$) δ 168.6, 145.8, 111.4, 71.1, 69.7, 52.1, 48.5, 31.8, 26.2, 21.4, 15.8; HRMS (ESI) m/z 219.0991 [(M + Na)$^+$ calcd for $C_{11}H_{16}O_3$ 219.0992].

For minor α,β-epoxy ester **S6**: colorless oil; ^{1}H NMR (500 MHz, $CDCl_3$) δ 4.87 (quint, J = 0.9 Hz, 1H), 4.78 (quint, J = 1.5 Hz, 1H), 3.78 (s, 3H), 3.20 (dd, J = 11.2, 7.5 Hz, 1H), 2.05 (dd, J = 13.0, 8.2 Hz, 1H), 1.80–1.70 (m, 5H), 1.44 (s, 3H), 1.40–1.28 (m, 1H); ^{13}C NMR (125 MHz, $CDCl_3$) δ 169.6, 144.1, 111.7, 69.65, 69.64, 52.4, 48.4, 32.7, 25.8, 21.1, 15.4; HRMS (ESI) m/z 219.0993 [(M + Na)$^+$ calcd for $C_{11}H_{16}O_3$ 219.0992].

Preparation of β-Hydroxy Ester 20

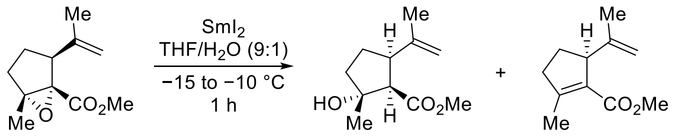

19 **20** (64%) **21** (18%)

To a cooled (−15 to −10 °C) solution of **19** (9.3 mg, 0.047 mmol) in degassed THF/H_2O (9:1, 0.6 mL) was added SmI_2 (0.1 M in THF, 3.76 mL, 0.38 mmol) dropwise. After stirring at the same temperature for 1 h, the reaction was quenched with an addition of saturated aqueous $NaHCO_3$, and the resulting mixture was diluted with Et_2O. The layers were separated, and the aqueous layer was extracted with Et_2O. The combined organic layers were dried over anhydrous Na_2SO_4 and concentrated *in vacuo*. The residue was purified by column chromatography (silica gel, hexanes/Et_2O, 8:1 to 3:2) to afford β-hydroxy ester **20** (6.0 mg, 64%, single diastereomer) and α,β-unsaturated ester **21** (1.5 mg, 18%).

For β-hydroxy ester **20**: colorless oil; ^1H NMR (500 MHz, $CDCl_3$) δ 4.80 (q, J = 1.5 Hz, 1H), 4.75 (s, 1H), 3.60 (s, 3H), 3.21 (q, J = 8.5 Hz, 1H), 2.94 (d, J = 6.7 Hz, 1H), 2.26–2.13 (m, 2H), 1.95–1.86 (m, 1H), 1.83–1.73 (m, 4H), 1.36 (s, 3H); ^{13}C NMR (125 MHz, $CDCl_3$) δ 172.9, 144.9, 110.2, 82.3, 61.4, 51.2, 48.3, 39.3, 26.3, 25.7, 23.4; HRMS (ESI) *m/z* 221.1147 $[(M + Na)^+$ calcd for $C_{11}H_{18}O_3$ 221.1148].

For α,β-unsaturated ester **21**: colorless oil; ^1H NMR (500 MHz, $CDCl_3$) δ 4.67 (quint, J = 1.7 Hz, 1H), 4.62 (s, 1H), 3.69 (s, 3H), 3.59 (d, J = 9.5 Hz, 1H), 2.55 (dt, J = 17.0, 8.2 Hz, 1H), 2.37 (ddd, J = 17.8, 9.6, 4.0 Hz, 1H), 2.16–2.04 (m, 4H), 1.69 (s, 3H), 1.64 (ddt, J = 12.8, 8.4, 3.9 Hz, 1H); HRMS (ESI) *m/z* 181.1223 $[(M + H)^+$ calcd for $C_{11}H_{16}O_2$ 181.1223].

Preparation of MOM-Ether S7

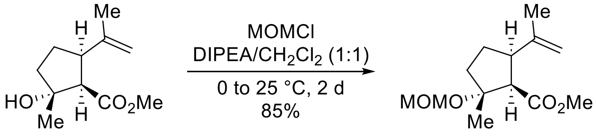

20 **S7**

To a cooled solution (0 °C) of **20** (14.0 mg, 0.071 mmol) in DIPEA/CH_2Cl_2 (1:1, 1.4 mL) was added MOMCl (54 μL, 0.71 mmol). After stirring at 0 °C for 5 min, the reaction mixture was warmed to 25 °C and stirred for 2 d. The reaction was quenched with an addition of saturated aqueous $NaHCO_3$, and the resulting mixture was diluted with Et_2O. The layers were separated, and

the aqueous layer was extracted with Et_2O. The combined organic layers were dried over anhydrous Na_2SO_4 and concentrated *in vacuo*. The residue was purified by column chromatography (silica gel, hexanes/Et_2O, 10:1) to afford MOM-ether **S7** (14.6 mg, 85%) as a colorless oil: ^{1}H NMR (500 MHz, $CDCl_3$) δ 4.79 (m, 1H), 4.74 (s, 1H), 4.73 (AB, J_{AB} = 7.4 Hz, $\Delta\nu_{AB}$ = 32.47 Hz, 2H), 3.59 (s, 3H), 3.39 (s, 3H), 3.20–3.11 (m, 2H), 2.23–2.11 (m, 1H), 2.09–1.98 (m, 2H), 1.91–1.81 (m, 1H), 1.76 (s, 3H), 1.32 (s, 3H); ^{13}C NMR (125 MHz, $CDCl_3$) δ 172.8, 145.0, 110.1, 91.6, 87.8, 59.1, 55.7, 51.2, 48.2, 37.1, 26.2, 23.4, 21.5; HRMS (ESI) *m*/*z* 265.1413 $[(M + Na)^+]$ calcd for $C_{13}H_{22}O_4$ 265.1410].

Preparation of Primary Alcohol 4

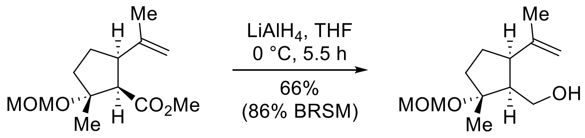

S7 **4**

To a cooled solution (0 °C) of **S7** (4.8 mg, 0.020 mmol) in anhydrous THF (0.8 mL) was added $LiAlH_4$ (2.0 M in THF, 40 µL, 0.080 mmol). After stirring at the same temperature for 5.5 h, the reaction was quenched with an addition of 15% NaOH solution. The resulting mixture was diluted with Et_2O and treated with Rochelle salt. The layers were separated, and the aqueous layer was extracted with CH_2Cl_2. The combined organic layers were dried over anhydrous Na_2SO_4 and concentrated *in vacuo*. The residue was purified by column chromatography (silica gel, hexanes/EtOAc, 4:1) to afford the primary alcohol **4** (2.8 mg, 66%, 86% BRSM) as a colorless oil with the recovered starting material (1.1 mg, 23%). Compound **4** was identical to the material previously prepared from **4** as described in **2.1. (*R*)-Carvone Route**.

2.3. Synthesis of 6,12-Guaianolide Core by Addition of Methyl Lithiopropiolate & Allyl Cuprate

Preparation of Propargylic Alcohol 3

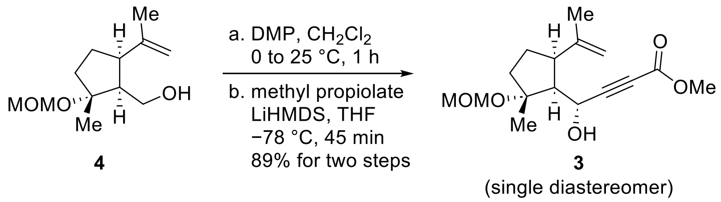

[*Oxidation*] To a cooled (0 °C) solution of **4** (290 mg, 1.4 mmol) in CH_2Cl_2 (6.8 mL) was added Dess–Martin periodinane (631 mg, 1.5 mmol). The reaction mixture was warmed to 25 °C and stirred for 1 h. The reaction was quenched with an addition of saturated aqueous $Na_2S_2O_3$ and saturated aqueous $NaHCO_3$, and the resulting mixture was diluted with CH_2Cl_2. The layers were separated, and the aqueous layer was extracted with CH_2Cl_2. The combined organic layers were dried over anhydrous Na_2SO_4 and concentrated *in vacuo* to afford the corresponding aldehyde as a colorless oil. The aldehyde was used in the following step without further purification; [*Methyl Lithiopropiolate Addition*] To a cooled (−78 °C) solution of methyl propiolate (0.6 mL, 6.8 mmol) in anhydrous THF (10 mL) was added LiHMDS (1.0 M in THF, 6.77 mL, 6.8 mmol) dropwise over 15 min. The resulting mixture was stirred at the same temperature for 30 min and treated with a solution of the crude aldehyde in anhydrous THF (10 mL). After stirring at –78 °C for 45 min, the reaction was quenched with an addition of saturated aqueous NH_4Cl, and the resulting mixture was diluted with Et_2O. The layers were separated, and the aqueous layer was extracted with EtOAc. The combined organic layers were dried over anhydrous Na_2SO_4 and concentrated *in vacuo*. The residue was purified by column chromatography (silica gel, hexanes/CH_2Cl_2/EtOAc 10:1:1) to afford propargylic alcohol **3** (356 mg, 89% for two steps, single diastereomer) as a colorless oil: ^{1}H NMR (500 MHz, $CDCl_3$) δ 5.01 (m, 1H), 4.96 (s, 1H), 4.74 (AB, J_{AB} = 7.4 Hz, $\Delta\nu_{AB}$ = 22.13 Hz, 2H), 4.58 (d, J = 5.3 Hz, 1H), 3.77 (s, 3H), 3.38 (s, 3H), 3.10 (q, J = 8.6 Hz, 1H), 2.68 (br s, 1H), 2.54 (dd, J = 8.1, 5.4 Hz, 1H), 2.03–1.91 (m, 3H), 1.91–1.81 (m, 4H), 1.53 (s, 3H); ^{13}C NMR (125 MHz, $CDCl_3$) δ 153.9, 145.9, 112.9, 91.6, 88.7, 87.6, 77.1, 61.5, 55.8, 55.7, 52.9, 47.1, 38.2, 27.6, 24.7, 21.4; HRMS (ESI) *m/z* 319.1522 [$(M + Na)^+$ calcd for $C_{16}H_{24}O_5$ 319.1516].

Preparation of α,β-Unsaturated γ-Lactone 22

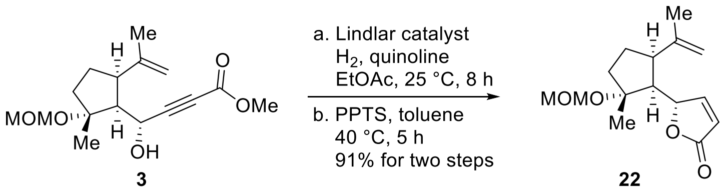

[*Partial Reduction*] To a solution of **3** (377 mg, 1.3 mmol) in EtOAc (12.7 mL) were added quinoline (75 μL, 0.64 mmol) and Lindlar catalyst (188 mg), and the resulting mixture was stirred for 2 h under H_2 atmosphere at 25 °C. An addition of Lindlar catalyst (188 mg) was repeated twice every 2 h. After stirring at the same temperature for an additional 2 h, the reaction mixture was filtered through a pad of Celite, washed with 0.1 N HCl, dried over Na_2SO_4, and concentrated *in vacuo* to afford the partially reduced α,β-unsaturated ester as a colorless oil. The α,β-unsaturated ester was used in the following step without further purification; [*Lactone Formation*] To a solution of the crude α,β-unsaturated ester in toluene (13 mL) was treated with pyridinium *p*-toluene sulfonate (64 mg, 0.25 mmol) at 25 °C. After stirring at 40 °C for 5 h, the reaction was quenched with an addition of saturated aqueous $NaHCO_3$, and the resulting mixture was diluted with EtOAc. The layers were separated, and the aqueous layer was extracted with EtOAc. The combined organic layers were dried over anhydrous Na_2SO_4 and concentrated *in vacuo*. The residue was purified by column chromatography (silica gel, hexanes/EtOAc, 4:1) to afford α,β-unsaturated γ-lactone **22** (308 mg, 91% for two steps) as a colorless oil: ^{1}H NMR (500 MHz, $CDCl_3$) δ 7.61 (dd, J = 5.7, 1.6 Hz, 1H), 6.06 (dd, J = 5.7, 2.3 Hz, 1H), 4.98 (dt, J = 5.7, 2.0 Hz, 1H), 4.96 (m, 1H), 4.86 (s, 1H), 4.71 (AB, J_{AB} = 7.4 Hz, $\Delta\nu_{AB}$ = 43.42 Hz, 2H), 3.38 (s, 3H), 3.17 (dt, J = 10.3, 7.4 Hz, 1H), 2.50 (t, J = 6.3 Hz, 1H), 2.07–1.98 (m, 1H), 1.97–1.79 (m, 3H), 1.78 (s, 3H), 1.32 (s, 3H); ^{13}C NMR (125 MHz, $CDCl_3$) δ 173.3, 156.8, 145.1, 121.8, 112.8, 91.6, 87.6, 82.6, 55.8, 53.2, 47.9, 37.6, 26.3, 23.8, 22.5; HRMS (ESI) *m/z* 289.1409 [(M + Na)$^+$ calcd for $C_{15}H_{22}O_4$ 289.1410].

Preparation of β-Allyl γ-Lactone 2

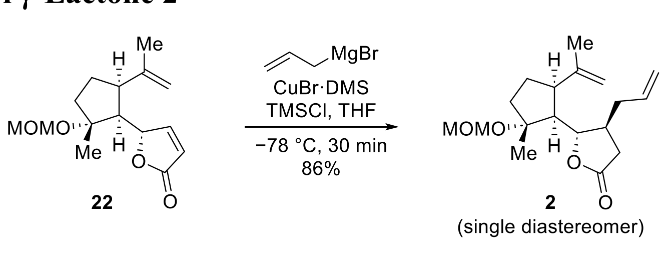

To a cooled solution (–78 °C) of CuBr•SMe_2 (683 mg, 3.3 mmol) in anhydrous THF (17.7 mL) were added allylmagnesium bromide (1.0 M in Et_2O, 6.65 mL, 6.7 mmol) and TMSCl (0.25 mL, 2.0 mmol) dropwise. After stirring for approximately 1 min, the solution of **22** (177 mg, 0.66 mmol) in anhydrous THF (17.7 mL) was added dropwise over 10 min at –78 °C. The stirring was continued at the same temperature for 30 min. The reaction was quenched with an addition of MeOH and saturated aqueous NH_4Cl, and the resulting mixture was diluted with Et_2O. The layers were separated, and the aqueous layer was extracted with EtOAc. The combined organic layers were dried over anhydrous Na_2SO_4 and concentrated *in vacuo*. The residue was purified by column chromatography (silica gel, hexanes/EtOAc, 9:1) to afford β-allyl γ-lactone **2** (176 mg, 86%, single diastereomer) as a colorless oil: ^{1}H NMR (500 MHz, $CDCl_3$) δ 5.76–5.64 (m, 1H), 5.13–5.06 (m, 2H), 4.95 (s, 1H), 4.85 (s, 1H), 4.73 (AB, J_{AB} = 7.5 Hz, $\Delta\nu_{AB}$ = 28.75 Hz, 2H), 4.13 (dd, *J* = 7.4, 3.6 Hz, 1H), 3.39 (s, 3H), 3.12 (q, *J* = 8.3 Hz, 1H), 2.65–2.51 (m, 2H), 2.38 (dd, *J* = 7.6, 3.7 Hz, 1H), 2.35–2.26 (m, 1H), 2.25–2.14 (m, 1H), 2.04 (dt, *J* = 14.7, 7.8 Hz, 1H), 1.99–1.76 (m, 4H), 1.75 (s, 3H), 1.42 (s, 3H); ^{13}C NMR (125 MHz, $CDCl_3$) δ 176.6, 144.7, 134.5, 118.0, 113.2, 91.5, 87.7, 83.4, 55.6, 51.5, 48.5, 39.3, 38.2, 37.3, 33.9, 26.5, 23.7, 22.2; HRMS (ESI) *m*/*z* 331.1883 [$(M + Na)^+$ calcd for $C_{18}H_{28}O_4$ 331.1899].

Preparation of Tricyclic Core 23

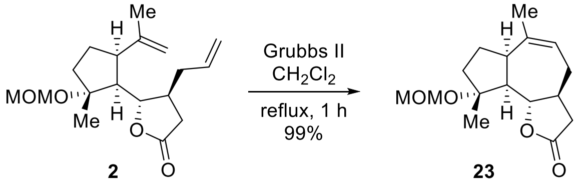

To a solution of **2** (44.5 mg, 0.14 mmol) in anhydrous CH_2Cl_2 (28.9 mL) was added Grubbs second-generation catalyst (6.1 mg, 0.0072 mmol) at 25 °C. The reaction mixture was heated to reflux for 1 h. The mixture was cooled to 25 °C, and DMSO (26 μL) was added to the solution. After stirring at 25 °C for 20 h, the reaction mixture was concentrated *in vacuo*. The residue was purified by column chromatography (silica gel, hexanes/EtOAc, 6:1) to afford the tricyclic core **23** (40 mg, 99%) as a colorless oil: ^{1}H NMR (500 MHz, $CDCl_3$) δ 5.45 (dq, *J* = 7.7, 1.5 Hz, 1H), 4.73 (AB, J_{AB} = 7.3 Hz, $\Delta\nu_{AB}$ = 12.41 Hz, 2H), 4.02 (dd, *J* = 10.8, 9.3 Hz, 1H), 3.39 (s, 3H), 3.18 (dt, *J* = 10.9, 7.6 Hz, 1H), 2.62–2.54 (m, 1H), 2.39–2.19 (m, 4H), 2.19–2.09 (m, 1H), 2.06–1.94 (m, 2H), 1.80–1.71 (m, 4H), 1.45 (m, 4H); ^{13}C NMR (125 MHz, $CDCl_3$) δ 176.0, 138.8, 121.4,

91.5, 88.5, 85.6, 55.7, 54.8, 44.2, 42.4, 36.8, 36.2, 30.5, 29.3, 27.8, 23.4; HRMS (ESI) *m*/*z* 303.1572 [$(M + Na)^+$ calcd for $C_{16}H_{24}O_4$ 303.1567].

2.4. Synthesis of 6,12-Guaianolide Core by Tandem Hydroallylation/Cyclization

2.4.1. Tandem Hydroallylation/Cyclization of MOM-Protected Alcohol 3

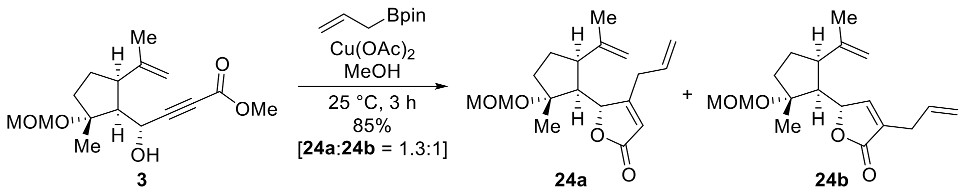

To a solution of **3** (8 mg, 0.027 mmol) in anhydrous MeOH (0.3 mL) were added allylboronic acid pinacol ester (10 μL, 0.054 mmol) and $Cu(OAc)_2$ (1.0 mg, 0.0027 mmol) at 25 °C. After 1.5 h, additional allylboronic acid pinacol ester (10 μL) and $Cu(OAc)_2$ (1.0 mg, 0.0027 mmol) were added. The resulting mixture was stirred at the same temperature for an additional 1.5 h and concentrated *in vacuo*. The residue was purified by column chromatography (silica gel, hexanes/EtOAc, 9:1) to afford a mixture of β-allyl α,β-unsaturated γ-lactone **24a** and α-allyl α,β-unsaturated γ-lactone **24b** (7 mg, 85%, **24a**:**24b** = 1.3:1 as determined by ^{1}H NMR).

For β-allyl α,β-unsaturated γ-lactone **24a**: colorless oil; ^{1}H NMR (500 MHz, $CDCl_3$) δ 5.90–5.78 (m, 2H), 5.29–5.21 (m, 2H), 5.03 (q, J = 1.5 Hz, 1H), 5.00 (s, 1H), 4.85 (t, J = 1.9 Hz, 1H), 4.72 (AB, J_{AB} = 7.7 Hz, $\Delta\nu_{AB}$ = 79.23 Hz, 2H), 3.40 (s, 3H), 3.34–3.22 (m, 2H), 3.07 (dd, J = 17.3, 7.3 Hz, 1H), 2.70 (d, J = 6.9 Hz, 1H), 2.15 (qd, J = 12.0, 6.2 Hz, 1H), 1.96 (dddd, J = 15.3, 9.0, 6.0, 1.2 Hz, 1H), 1.86 (ddd, J = 14.5, 11.8, 4.1 Hz, 1H), 1.82–1.72 (m, 4H), 1.10 (s, 3H); ^{13}C NMR (125 MHz, $CDCl_3$) δ 173.4, 172.8, 144.0, 131.8, 119.7, 117.2, 113.5, 91.5, 87.9, 82.5, 55.8, 49.9, 48.5, 38.1, 33.2, 25.7, 23.9, 21.5; HRMS (ESI) m/z 329.1724 [(M + Na)$^+$ calcd for $C_{18}H_{26}O_4$ 329.1723].

For α-allyl α,β-unsaturated γ-lactone **24b**: colorless oil; ^{1}H NMR (500 MHz, $CDCl_3$) δ 7.15 (q, J = 1.7 Hz, 1H), 5.85 (ddt, J = 17.0, 10.1, 6.8 Hz, 1H), 5.20–5.11 (m, 2H), 4.93 (q, J = 1.5 Hz, 1H), 4.89 (dq, J = 4.3, 2.1 Hz, 1H), 4.82 (s, 1H), 4.72 (AB, J_{AB} = 7.5 Hz, $\Delta\nu_{AB}$ = 41.09 Hz, 2H), 3.38 (s, 3H), 3.15 (q, J = 7.8 Hz, 1H), 3.01–2.95 (m, 2H), 2.50 (t, J = 6.1 Hz, 1H), 2.08–1.99 (m, 1H), 1.96–1.79 (m, 3H), 1.78 (s, 3H), 1.31 (s, 3H); ^{13}C NMR (125 MHz, $CDCl_3$) δ 173.5, 148.9, 145.2, 133.15, 133.08, 118.0, 112.4, 91.6, 87.5, 80.7, 55.8, 53.2, 47.9, 37.6, 29.7, 26.3, 23.8, 22.5; HRMS (ESI) m/z 329.1726 [(M + Na)$^+$ calcd for $C_{18}H_{26}O_4$ 329.1723].

2.4.2. Preparation of Epoxide Substrate 25

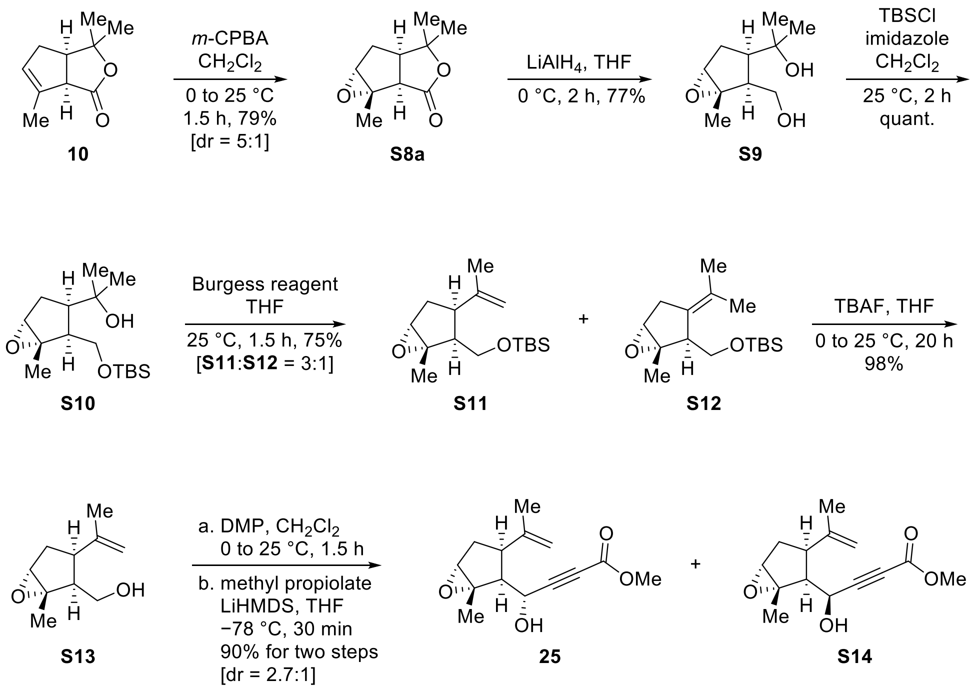

Preparation of α-Epoxide S8a

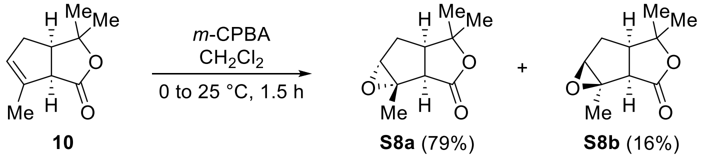

To a cooled solution (0 °C) of **10** (1.46 g, 8.8 mmol) in CH_2Cl_2 (35 mL) was added *m*-CPBA (2.42 g, 14 mmol) portionwise. The reaction mixture was warmed to 25 °C and stirred for 1.5 h. The reaction was quenched with an addition of saturated aqueous $NaHCO_3$ and saturated aqueous Na_2SO_3. The layers were separated, and the aqueous layer was extracted with EtOAc. The combined organic layers were washed with saturated aqueous $NaHCO_3$ and brine, dried over anhydrous Na_2SO_4, and concentrated *in vacuo*. The residue was purified by column chromatography (silica gel, hexanes/EtOAc, 7:1 to 7:3) to afford α-epoxide **S8a** (12.8 g, 79%) and β-epoxide **S8b** (250 mg, 16%).

For α-epoxide **S8a**: white solid; ^{1}H NMR (500 MHz, $CDCl_3$) δ 3.36 (s, 1H), 3.35 (d, J = 7.5 Hz, 1 H), 2.42 (q, J = 8.3 Hz, 1H), 2.17 (dd, J = 14.4, 8.3 Hz, 1H), 1.87 (dd, J = 14.4, 8.8 Hz, 1H), 1.69 (s, 3H), 1.40 (s, 3H), 1.38 (s, 3H); ^{13}C NMR (100 MHz, $CDCl_3$) δ 174.6, 83.4, 63.7, 62.7,

51.9, 45.5, 29.4, 28.8, 23.3, 14.7; HRMS (ESI) *m*/*z* 183.1018 [(M + H)$^+$ calcd for $C_{10}H_{14}O_3$ 183.1016].

For β-epoxide **S8b**: white solid; ^{1}H NMR (400 MHz, $CDCl_3$) δ 3.41 (s, 1H), 3.12 (d, *J* = 10.7 Hz, 1H), 2.90 (td, *J* = 10.4, 2.0 Hz, 1H), 2.21 (dd, *J* = 15.6, 1.9 Hz, 1H), 1.99 (ddd, *J* = 15.6, 10.1, 1.8 Hz, 1H), 1.62 (s, 3H), 1.37 (s, 3H), 1.35 (s, 3H); ^{13}C NMR (100 MHz, $CDCl_3$) δ 174.6, 86.3, 67.1, 66.9, 51.8, 48.8, 32.7, 29.9, 24.8, 16.3; HRMS (ESI) *m*/*z* 183.1020 [(M + H)$^+$ calcd for $C_{10}H_{14}O_3$ 183.1016].

Preparation of Diol S9

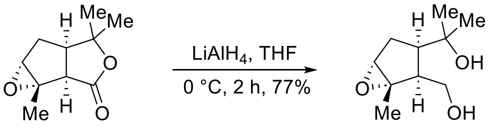

S8a **S9**

To a cooled solution (0 °C) of **S8a** (89 mg, 0.49 mmol) in anhydrous THF (3 mL) was added $LiAlH_4$ (2.0 M in THF, 0.49 mL, 0.98 mmol) dropwise. After stirring at the same temperature for 2 h, the reaction was quenched with an addition of H_2O. The resulting mixture was diluted with CH_2Cl_2 and treated with Rochelle salt. The layers were separated, and the aqueous layer was extracted with CH_2Cl_2. The combined organic layers were dried over anhydrous Na_2SO_4 and concentrated *in vacuo*. The residue was purified by column chromatography (silica gel, hexanes/CH_2Cl_2/EtOAc, 1:1:1) to afford diol **S9** (70 mg, 77%) as a white solid: ^{1}H NMR (500 MHz, $CDCl_3$) δ 4.15 (dd, *J* = 12.6, 2.4 Hz, 1H), 3.73 (dd, *J* = 12.6, 2.9 Hz, 1H), 3.38 (s, 1H), 2.10 (dt, *J* = 5.5, 2.6 Hz, 1H), 2.06–1.93 (m, 2H), 1.93–1.83 (m, 1H), 1.53 (s, 3H), 1.39 (s, 3H), 1.28 (s, 3H); ^{13}C NMR (100 MHz, $CDCl_3$) δ 72.2, 65.4, 63.1, 60.9, 46.3, 45.5, 31.6, 29.7, 29.5, 16.0; HRMS (ESI) *m*/*z* 169.1223 [(M + H – H_2O)$^+$ calcd for $C_{10}H_{18}O_3$ 169.1223].

Preparation of Mono TBS-Ether S10

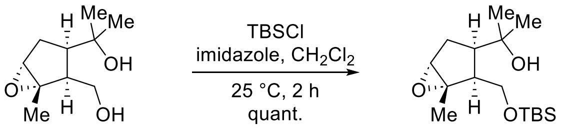

S9 **S10**

To a solution of **S9** (70 mg, 0.38 mmol) in CH_2Cl_2 (4 mL) were added TBSCl (113 mg, 0.75 mmol) and imidazole (102 mg, 1.5 mmol) at 25 °C. After stirring at 25 °C for 2 h, the reaction was

quenched with an addition of saturated aqueous $NaHCO_3$, and the resulting mixture was diluted with EtOAc. The layers were separated, and the aqueous layer was extracted with EtOAc. The combined organic layers were dried over anhydrous Na_2SO_4 and concentrated *in vacuo*. The residue was purified by column chromatography (silica gel, hexanes/EtOAc, 10:1) to afford mono TBS-ether **S10** (113 mg, quant.) as a white solid: ^{1}H NMR (400 MHz, $CDCl_3$) δ 4.06 (dd, J = 11.1, 2.7 Hz, 1H), 3.96 (s, 1H), 3.80 (dd, J = 11.1, 4.8 Hz, 1H), 3.29 (s, 1H), 2.19 (ddd, J = 7.2, 4.7, 2.5 Hz, 1H), 2.07–1.95 (m, 2H), 1.90–1.78 (m, 1H), 1.44 (s, 3H), 1.30 (s, 3H), 1.17 (s, 3H), 0.90 (s, 9H), 0.10 (s, 6H); ^{13}C NMR (100 MHz, $CDCl_3$) δ 70.5, 64.2, 62.6, 61.5, 46.8, 45.1, 30.6, 29.8, 29.0, 25.87, 25.84, 25.81, 18.1, 16.1, -5.56, -5.63; HRMS (ESI) m/z 283.2094 [(M + H – H_2O)$^+$ calcd for $C_{16}H_{32}O_3Si$ 283.2088].

Preparation of Disubstituted Alkene S11

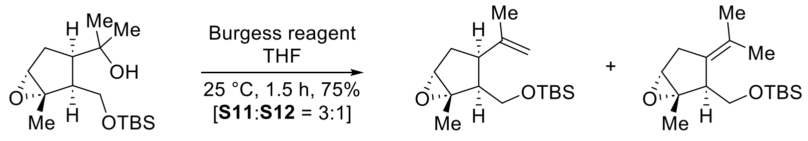

To a solution of **S10** (77 mg, 0.26 mmol) in anhydrous THF (3 mL) was added Burgess reagent (122 mg, 0.51 mmol) at 25 °C. After stirring at 25 °C for 1.5 h, the reaction was quenched with an addition of saturated aqueous $NaHCO_3$, and the resulting mixture was diluted with Et_2O. The layers were separated, and the aqueous layer was extracted with EtOAc. The combined organic layers were dried over anhydrous Na_2SO_4 and concentrated *in vacuo*. The residue was purified by column chromatography (silica gel, hexanes/EtOAc, 70:1) to afford a mixture of disubstituted alkene **S11** and tetrasubstituted alkene **S12** (54 mg, 75%, **S11**:**S12** = 3:1 as determined by ^{1}H NMR).

For disubstituted alkene **S11**: colorless oil; ^{1}H NMR (500 MHz, $CDCl_3$) δ 4.87 (q, J = 1.5 Hz, 1H), 4.68 (s, 1H), 3.63 (dd, J = 10.4, 3.0 Hz, 1H), 3.49 (dd, J = 10.5, 3.5 Hz, 1H), 3.30 (s, 1H), 2.54–2.46 (m, 1H), 2.13 (dt, J = 7.1, 3.3 Hz, 1H), 1.97 (ddd, J = 12.6, 11.2, 1.3 Hz, 1H), 1.89 (dd, J = 13.0, 7.0 Hz, 1H), 1.71 (s, 3H), 1.51 (s, 3H), 0.87 (s, 9H), 0.01 (s, 3H), -0.02 (s, 3H); ^{13}C NMR (125 MHz, $CDCl_3$) δ 143.6, 111.0, 65.0, 63.5, 60.8, 45.6, 43.1, 31.1, 25.9, 23.8, 18.1, 16.6, -5.5, -5.8; HRMS (ESI) m/z 283.2094 [(M + H)$^+$ calcd for $C_{16}H_{30}O_2Si$ 283.2088].

For tetrasubstituted alkene **S12**: colorless oil; ^{1}H NMR (500 MHz, $CDCl_3$) δ 3.75 (dd, J = 10.1, 2.9 Hz, 1H), 3.66 (dd, J = 10.1, 4.6 Hz, 1H), 3.32 (s, 1H), 2.78 (s, 1H), 2.62 (d, J = 16.6 Hz, 1H),

2.33 (ddt, J = 16.6, 3.9, 1.9 Hz, 1H), 1.62 (s, 3H), 1.58 (s, 3H), 1.51 (s, 3H), 0.87 (s, 9H), 0.02 (s, 3H), -0.02 (s, 3H); HRMS (ESI) m/z 283.2094 $[(M + H)^+$ calcd for $C_{16}H_{30}O_2Si$ 283.2088].

Preparation of Primary Alcohol S13

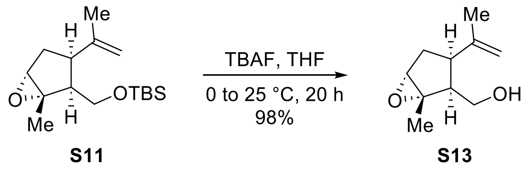

To a cooled (0 °C) solution of **S11** (764 mg, 2.7 mmol) in THF (14 mL) was added TBAF (1 M in THF, 4.1 mL, 4.1 mmol). After stirring at 0 °C for 5 min, the reaction mixture was warmed to 25 °C. After stirring for 20 h, the reaction was quenched with an addition of saturated aqueous NH_4Cl, and the resulting mixture was diluted with Et_2O. The layers were separated, and the aqueous layer was extracted with EtOAc. The combined organic layers were dried over anhydrous Na_2SO_4 and concentrated *in vacuo*. The residue was purified by column chromatography (silica gel, hexanes/ CH_2Cl_2/EtOAc, 6:1:1) to afford primary alcohol **S13** (443 mg, 98%) as a white solid: ^1H NMR (500 MHz, $CDCl_3$) δ 4.94 (q, J = 1.5 Hz, 1H), 4.79 (s, 1H), 3.69 (dd, J = 12.0, 3.6 Hz, 1H), 3.65 (dd, J = 12.0, 3.5 Hz, 1H), 3.38 (s, 1H), 2.57 (q, J = 8.2 Hz, 1H), 2.23 (dt, J = 7.4, 3.6 Hz, 1H), 2.01–1.89 (m, 2H), 1.80 (s, 3H), 1.54 (s, 3H); HRMS (ESI) m/z 169.1224 $[(M + H)^+$ calcd for $C_{10}H_{16}O_2$ 169.1223].

Preparation of Epoxide Substrate 25

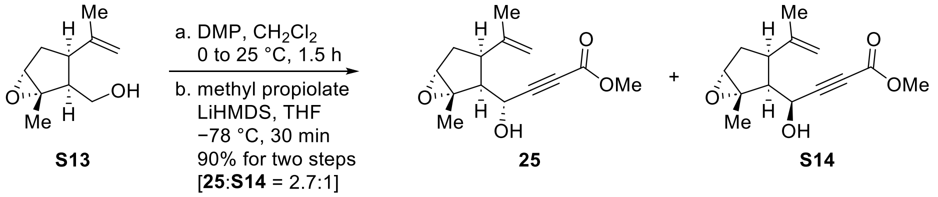

[*Oxidation*] To a cooled (0 °C) solution of **S13** (180 mg, 1.1 mmol) in CH_2Cl_2 (5.3 mL) was added Dess–Martin periodinane (476 mg, 1.1 mmol). The reaction mixture was warmed to 25 °C and stirred for 1.5 h. The reaction was quenched with an addition of saturated aqueous $Na_2S_2O_3$ and saturated aqueous $NaHCO_3$, and the resulting mixture was diluted with CH_2Cl_2. The layers were separated, and the aqueous layer was extracted with CH_2Cl_2. The combined organic layers were washed with saturated aqueous $NaHCO_3$, dried over anhydrous Na_2SO_4, and concentrated *in vacuo* to afford the corresponding aldehyde as a colorless oil. The aldehyde was used in the following

step without further purification; [*Methyl Lithiopropiolate Addition*] To a cooled (−78 °C) solution of methyl propiolate (0.48 mL, 5.3 mmol) in anhydrous THF (9 mL) was added LiHMDS (1.0 M in THF, 5.3 mL, 5.3 mmol) dropwise. The resulting mixture was stirred at the same temperature for 30 min and treated with a solution of the crude aldehyde in anhydrous THF (9 mL). After stirring at –78 °C for 30 min, the reaction was quenched with an addition of saturated aqueous NH_4Cl, and the resulting mixture was diluted with Et_2O. The layers were separated, and the aqueous layer was extracted with EtOAc. The combined organic layers were dried over anhydrous Na_2SO_4 and concentrated *in vacuo*. The residue was purified by column chromatography (silica gel, hexanes/EtOAc 6:1) to afford a mixture of diastereomeric α,β-propargylic alcohols **25** and **S14** (240 mg, 90% for two steps, **25**:**S14** = 2.7:1 as determined by ^{1}H NMR) as a colorless oil.

For α-propargylic alcohol **25**: ^{1}H NMR (500 MHz, $CDCl_3$) δ 5.03 (q, *J* = 1.4 Hz, 1H), 4.89 (s, 1H), 4.60 (dd, *J* = 5.9, 1.9 Hz, 1H), 3.80 (s, 3H), 3.39 (s, 1H), 2.65–2.57 (m, 1H), 2.50 (dd, *J* = 7.7, 1.9 Hz, 1H), 2.08 (ddd, *J* = 12.9, 11.4, 1.3 Hz, 1H), 1.98 (d, *J* = 6.0 Hz, 1H), 1.92 (dd, *J* = 13.4, 7.0 Hz, 1H), 1.77 (s, 3H), 1.66 (s, 3H); HRMS (ESI) *m*/*z* 251.1283 [$(M + H)^+$ calcd for $C_{14}H_{18}O_4$ 251.1205].

For β-propargylic alcohol **S14**: ^{1}H NMR (500 MHz, $CDCl_3$) δ 4.97 (q, *J* = 1.4 Hz, 1H), 4.83 (s, 1H), 4.48 (d, *J* = 2.7 Hz, 1H), 3.79 (s, 3H), 3.42 (s, 1H), 2.67–2.58 (m, 1H), 2.52 (dd, *J* = 8.0, 2.7 Hz, 1H), 2.17 (ddd, *J* = 13.0, 11.5, 1.3 Hz, 1H), 1.94 (dd, *J* = 13.7, 7.0 Hz, 2H), 1.78 (s, 3H), 1.65 (s, 3H); HRMS (ESI) *m*/*z* 251.1277 [$(M + H)^+$ calcd for $C_{14}H_{18}O_4$ 251.1205].

2.4.3. Tandem Hydroallylation/Cyclization of Epoxide Substrate 25

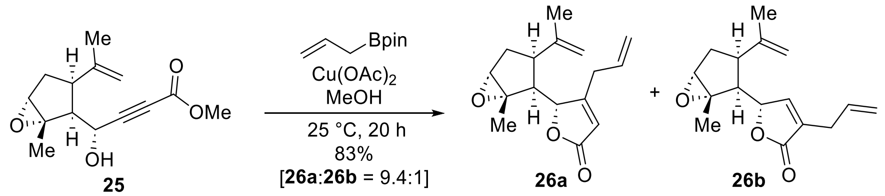

To a solution of **25** (65 mg, 0.26 mmol) in MeOH (0.87 mL) were added allylboronic acid pinacol ester (97 μL, 0.52 mmol) and $Cu(OAc)_2$ (4.7 mg, 0.026 mmol), and the resulting mixture was stirred at 25 °C for 2 h. An addition of allylboronic acid pinacol ester (49 μL, 0.26 mmol) and $Cu(OAc)_2$ (2.4 mg, 0.013 mmol) were repeated twice every 2 h. When the starting material was completely consumed, the reaction mixture was concentrated *in vacuo*. The residue was purified by column chromatography (silica gel, hexanes/EtOAc, 9:1) to afford a mixture of β-allyl α,β-

unsaturated γ-lactone **26a** and α-allyl α,β-unsaturated γ-lactone **26b** (56 mg, 83%, **26a**:**26b** = 9.4:1 as determined by ^{1}H NMR).

For β-allyl α,β-unsaturated γ-lactone **26a**: colorless oil; ^{1}H NMR (500 MHz, $CDCl_3$) δ 5.90 (q, *J* = 1.8 Hz, 1H), 5.89–5.79 (m, 1H), 5.31–5.23 (m, 2H), 5.04 (q, *J* = 1.4 Hz, 1H), 4.91 (s, 1H), 4.85 (s, 1H), 3.37 (s, 1H), 3.29 (dd, *J* = 17.2, 6.5 Hz, 1H), 3.08 (dd, *J* = 17.2, 7.3 Hz, 1H), 2.72 (dt, *J* = 12.5, 7.1 Hz, 1H), 2.64 (d, *J* = 7.5 Hz, 1H), 2.17 (dd, *J* = 13.0, 11.5 Hz, 1H), 1.97 (dd, *J* = 13.3, 6.6 Hz, 1H), 1.72 (s, 3H), 1.23 (s, 3H); HRMS (ESI) *m*/*z* 261.1486 [$(M + H)^+$ calcd for $C_{16}H_{20}O_3$ 261.1485].

For α-allyl α,β-unsaturated γ-lactone **26b**: colorless oil; ^{1}H NMR (500 MHz, $CDCl_3$) δ 7.06 (q, *J* = 1.7 Hz, 1H), 5.86 (ddt, *J* = 17.0, 10.1, 6.9 Hz, 1H), 5.23–5.14 (m, 2H), 4.99–4.93 (m, 2H), 4.79 (s, 1H), 3.36 (s, 1H), 3.01 (dq, *J* = 7.1, 1.9 Hz, 2H), 2.64 (dt, *J* = 11.4, 7.1 Hz, 1H), 2.60 (dd, *J* = 7.3, 2.3 Hz, 1H), 2.05 (ddd, *J* = 12.6, 11.3 Hz, 1H), 1.97 (dd, *J* = 13.5, 6.6 Hz, 1H), 1.75 (s, 3H), 1.26 (s, 3H); HRMS (ESI) *m*/*z* 283.1313 [$(M + Na)^+$ calcd for $C_{16}H_{20}O_3$ 283.1305].

2.5. Completion of the Synthesis of 4α,9α,10α-Trihydroxyguaia-11(13)en-12,6α-olide (1)

Preparation of Diol 27

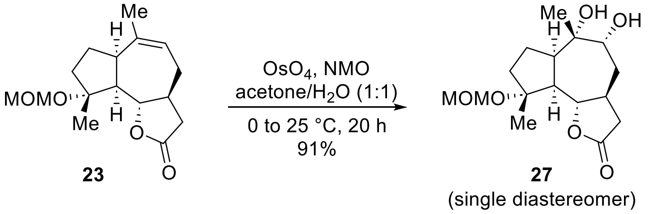

To a cooled (0 °C) solution of **23** (40 mg, 0.14 mmol) in acetone/H_2O (1:1, 2.8 mL) were added OsO_4 (4 wt. % in H_2O, 9 μL, 0.0014 mmol) and NMO (33 mg, 0.29 mmol). The reaction mixture was warmed to 25 °C and stirred for 20 h. The reaction was quenched with an addition of saturated aqueous $Na_2S_2O_3$, and the resulting mixture was diluted with EtOAc. The layers were separated, and the aqueous layer was extracted with CH_2Cl_2. The combined organic layers were dried over anhydrous Na_2SO_4 and concentrated *in vacuo*. The residue was purified by column chromatography (silica gel, EtOAc/MeOH, 49:1) to afford diol **27** (40 mg, 91%, single diastereomer) as a white solid: ^1H NMR (500 MHz, CD_3OD) δ 4.73 (AB, J_{AB} = 7.5 Hz, $\Delta\nu_{AB}$ = 22.16 Hz, 2H), 4.44 (dd, J = 11.6, 9.9 Hz, 1H), 3.70 (dd, J = 4.8, 2.5 Hz, 1H), 3.36 (s, 3H), 2.99 (ddd, J = 12.3, 10.1, 7.9 Hz, 1H), 2.73–2.60 (m, 1H), 2.50 (dd, J = 17.0, 7.9 Hz, 1H), 2.41–2.30 (m, 2H), 1.98 (ddd, J = 14.7, 4.8, 2.9 Hz, 1H), 1.94–1.81 (m, 3H), 1.77 (ddd, J = 14.6, 12.0, 2.6 Hz, 1H), 1.73–1.62 (m, 1H), 1.36 (s, 3H), 1.18 (s, 3H); ^{13}C NMR (125 MHz, CD_3OD) δ 178.9, 92.5, 87.0, 85.4, 77.9, 77.7, 55.5, 54.9, 42.4, 39.6, 38.3, 36.8, 34.7, 26.2, 23.3, 20.7; HRMS (ESI) *m*/*z* 353.1371 [$(M + K)^+$ calcd for $C_{16}H_{26}O_6$ 353.1622].

Preparation of 1,2-Acetonide 28

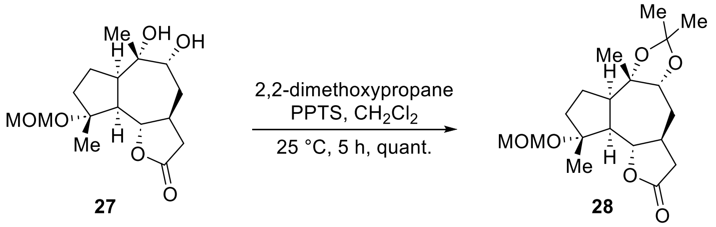

To a solution of **27** (14 mg, 0.045 mmol) in CH_2Cl_2 (1 mL) were added 2,2-dimethoxypropane (55 μL, 0.45 mmol) and pyridinium *p*-toluene sulfonate (1.1 mg, 0.0045 mmol) at 25 °C. After stirring at 25 °C for 5 h, the reaction was quenched with an addition of Et_3N (10 μL), and the resulting mixture was concentrated *in vacuo*. The residue was purified by column chromatography (silica

gel, hexanes/EtOAc, 3:2) to afford 1,2-acetonide **28** (16 mg, quant.) as a white solid: ^{1}H NMR (500 MHz, $CDCl_3$) δ 4.74 (AB, J_{AB} = 7.4 Hz, $\Delta\nu_{AB}$ = 31.56 Hz, 2H), 4.04 (dd, *J* = 11.3, 9.0 Hz, 1H), 3.87 (dd, *J* = 4.6, 2.2 Hz, 1H), 3.37 (s, 3H), 2.97 (ddd, *J* = 9.8, 8.1, 4.3 Hz, 1H), 2.74–2.61 (m, 2H), 2.58 (t, *J* = 10.6 Hz, 1H), 2.37 (dt, *J* = 15.7, 2.2 Hz, 1H), 2.29–2.18 (m, 1H), 2.08 (ddd, *J* = 13.8, 7.7, 4.2 Hz, 1H), 2.02–1.88 (m, 2H), 1.78–1.66 (m, 2H), 1.46 (s, 3H), 1.40 (s, 3H), 1.34 (s, 3H), 1.26 (s, 3H); ^{13}C NMR (125 MHz, $CDCl_3$) δ 175.5, 106.2, 91.7, 86.0, 84.8, 84.1, 81.7, 57.4, 55.3, 43.6, 40.2, 36.7, 36.1, 31.7, 28.3, 27.2, 25.4, 23.1, 21.9; HRMS (ESI) *m/z* 393.1683 $[(M + K)^+]$ calcd for $C_{19}H_{30}O_6$ 393.1674].

Preparation of α-*exo*-Methylene γ-Lactone 29

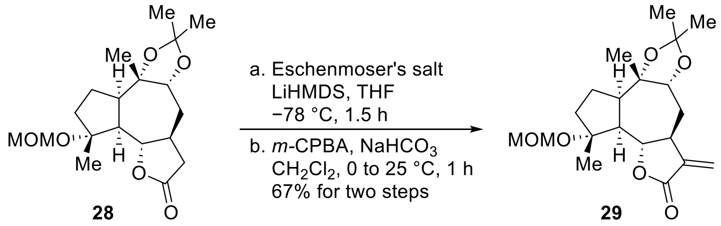

[*Alkylation with Eschenmoser's Salt*] To a cooled (–78 °C) solution of **28** (23 mg, 0.065 mmol) in anhydrous THF (3.3 mL) was added LiHMDS (1.0 M in THF, 0.324 mL, 0.32 mmol) dropwise. The reaction mixture was stirred at –78 °C for 30 min before Eschenmoser's salt (60 mg, 0.32 mmol) was added. After stirring at the same temperature for 1.5 h, the reaction was quenched with an addition of saturated aqueous NH_4Cl, and the resulting mixture was diluted with Et_2O. The layers were separated, and the aqueous layer was extracted with CH_2Cl_2. The combined organic layers were dried over anhydrous Na_2SO_4 and concentrated *in vacuo* to afford the intermediate amine as a colorless oil. The amine was used in the following step without further purification; [*Oxidation/Elimination*] To a cooled (0 °C) solution of the above crude amine in CH_2Cl_2 (6.2 mL) was added *m*-CPBA (58 mg, 0.34 mmol). The reaction mixture was warmed to 25 °C and stirred for 1 h. The reaction was quenched with an addition of saturated aqueous $NaHCO_3$ and saturated aqueous Na_2SO_3, and the resulting mixture was diluted with CH_2Cl_2. The layers were separated, and the aqueous layer was extracted with CH_2Cl_2. The combined organic layers were dried over anhydrous Na_2SO_4 and concentrated *in vacuo*. The residue was purified by column chromatography (silica gel, hexanes/EtOAc, 3:1) to afford α-*exo*-methylene γ-lactone **29** (16 mg, 67% for two steps) as a colorless oil: ^{1}H NMR (500 MHz, $CDCl_3$) δ 6.26 (d, *J* = 3.4 Hz, 1H), 5.54

(d, J = 3.0 Hz, 1H), 4.75 (AB, J_{AB} = 7.3 Hz, $\Delta\nu_{AB}$ = 39.38 Hz, 2H), 3.99 (dd, J = 11.6, 8.7 Hz, 1H), 3.92 (dd, J = 4.8, 2.0 Hz, 1H), 3.37 (s, 3H), 3.09 (ddq, J = 11.8, 9.0, 3.1 Hz, 1H), 2.96 (ddd, J = 11.3, 7.2, 4.7 Hz, 1H), 2.64 (t, J = 10.9 Hz, 1H), 2.53 (dt, J = 15.5, 2.4 Hz, 1H), 2.09 (dt, J = 13.6, 5.9 Hz, 1H), 2.04–1.92 (m, 2H), 1.82–1.68 (m, 2H), 1.46 (s, 3H), 1.42 (s, 3H), 1.35 (s, 3H), 1.25 (s, 3H); ^{13}C NMR (125 MHz, $CDCl_3$) δ 169.9, 138.9, 121.1, 106.3, 91.8, 85.9, 84.4, 82.9, 81.5, 57.6, 55.3, 43.5, 40.60, 40.55, 30.3, 28.3, 27.1, 25.4, 22.7, 21.8; HRMS (ESI) *m/z* 405.1680 [(M + K)$^+$ calcd for $C_{20}H_{30}O_6$ 405.1674].

Preparation of 4α,9α,10α-Trihydroxyguaia-11(13)en-12,6α-olide (1)

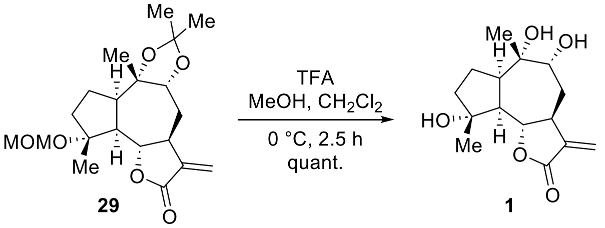

To a cooled (0 °C) solution of **29** (6 mg, 0.016 mmol) in CH_2Cl_2 (0.6 mL) were added MeOH (12 μL) and TFA (60 μL). After stirring at 0 °C for 2.5 h, the solvents were removed under reduced pressure to afford 4α,9α,10α-trihydroxyguaia-11(13)en-12,6α-olide (**1**) (5 mg, quant.) as a white solid: $[\alpha]_D^{24}$ –14.9° (*c* 0.1, MeOH); ^{1}H NMR (500 MHz, CD_3OD) δ 6.12 (d, J = 3.6 Hz, 1H), 5.57 (d, J = 3.3 Hz, 1H), 4.38 (dd, J = 11.8, 9.4 Hz, 1H), 3.79 (dd, J = 4.8, 2.5 Hz, 1H), 3.16–3.02 (m, 2H), 2.26 (t, J = 12.1 Hz, 1H), 2.21 (ddd, J = 14.6, 4.9, 2.9 Hz, 1H), 1.88 (dddd, J = 12.7, 8.9, 6.6, 2.2 Hz, 1H), 1.83–1.72 (m, 3H), 1.67 (tdd, J = 12.3, 10.1, 6.7 Hz, 1H), 1.33 (s, 3H), 1.18 (s, 3H); ^{13}C NMR (125 MHz, CD_3OD) δ 172.1, 141.4, 119.7, 84.0, 81.0, 77.9, 77.5, 56.1, 42.6, 42.0, 41.9, 33.1, 25.9, 23.3, 23.1; HRMS (ESI) *m/z* 300.1810 [(M + NH_4)$^+$ calcd for $C_{15}H_{22}O_5$ 300.1806].

3. Table S1. Mukaiyama Hydration of Bicyclic Lactone **10**[7-9]

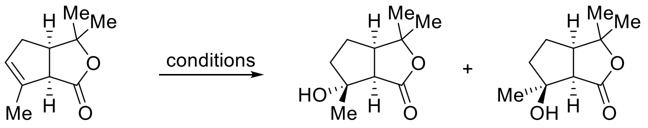

Entry	Conditions	dr (**11**:**S1**)[a]	Yield of **11** (%)[b]
1	Co(acac)$_2$ (0.3 eq), PhSiH$_3$ (2 eq), O$_2$, THF (0.1 M), 25 ºC, 3 h	1:1	33
2	Co(acac)$_2$ (0.3 eq), PhSiH$_3$ (2 eq), O$_2$, *i*-PrOH (0.1 M), 25 ºC, 3 h	1:1	ND[c]
3	Mn(dpm)$_3$ (0.05 eq), PhSiH$_3$ (2 eq), O$_2$, *i*-PrOH (0.14 M), 0 to 25 ºC, 20 h	1:1	38
4	Fe(acac)$_3$ (0.03 eq), 4-MeO$_3$S-PhNO$_2$ (1.3 eq), NaHCO$_3$ (2 eq), PhSiH$_3$ (3 eq), MeOH/THF (2:1, 0.25 M), 0 to 15 °C, 2 h	4:1	69

[a] Diastereomeric ratio determined by integration of the ^{1}H NMR spectrum of the crude product.

[b] Yield of isolated **11**.

[c] Not determined.

4. Table S2. Dehydration of Mono TBS-Ether **13**[10,11]

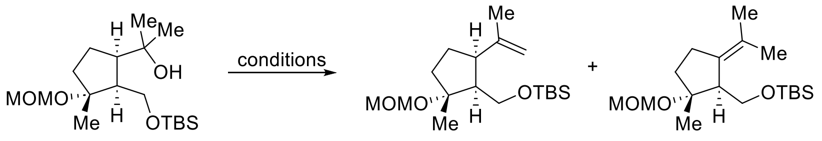

Entry	Conditions	Ratio of **14**:**15**[a]	Yield (%)[b]
1	Burgess reagent (4 eq), THF (0.06 M), 25 °C, 1 h	ND[c]	47
2	Burgess reagent (2 eq), toluene (0.07 M), 25 °C, 3 d	3.4:1	19
3	Burgess reagent (2 eq), THF (0.07 M), 0 °C, 1 h	2.9:1	61
4	Burgess reagent (2 eq), K_2CO_3 (10 eq), THF (0.1 M), 0 °C, 1 h	3:1	ND
5	Burgess reagent (1.5 eq), NaHMDS (2 eq), THF (0.1 M), 0 °C, 1 h	6.9:1	84

[a] Ratio determined by integration of the ^{1}H NMR spectrum of the crude product.

[b] Combined yield of isolated **14** and **15**.

[c] Not determined.

5. Table S3. SmI_2-Mediated Reductive Opening of α,β-Epoxy Ester **19**[12]

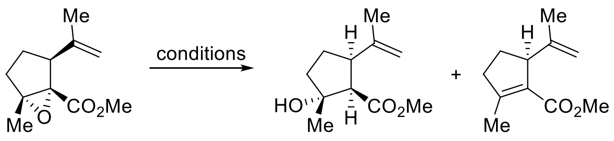

19 **20** **21**

Entry	Conditions	Ratio of **20**:**21**[a]	Yield of **20** (%)[b]
1	SmI_2 (13 eq), THF/MeOH (5:2, 0.05 M), −70 to 25 °C, 3 h	2.7:1	75[c]
2	SmI_2 (6 eq), THF/H_2O (9:1, 0.08 M), 0 °C, 1 h	3:1	61
3	SmI_2 (8 eq), THF/H_2O (9:1, 0.08 M), −15 to −10 °C, 1 h	3.7:1	64

[a] Ratio determined by integration of the ^{1}H NMR spectrum of the crude product.

[b] Yield of isolated **20**.

[c] The yield includes inseparable impurities.

6. Mosher Ester Analysis of Alcohol 3

6.1. Preparation of Mosher Esters (S15 and S16)

Preparation of (*S*)-Mosher Ester S15

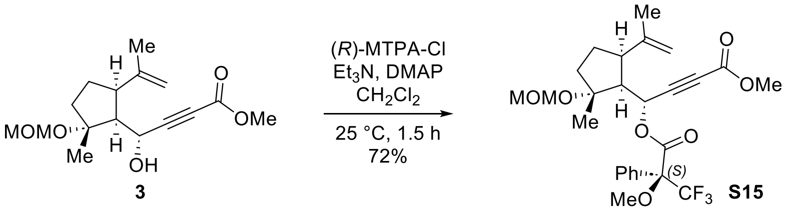

To a solution of **3** (2.4 mg, 0.0081 mmol) in anhydrous CH_2Cl_2 (0.25 mL) were added (*R*)-MTPA-Cl (16 μL, 0.088 mmol), Et_3N (24 μL, 0.18 mmol), and DMAP (10 crystals) 25 °C. After stirring at 25 °C for 1.5 h, the reaction was quenched with an addition of saturated aqueous $NaHCO_3$, and the resulting mixture was diluted with CH_2Cl_2. The layers were separated, and the aqueous layer was extracted with CH_2Cl_2. The combined organic layers were dried over anhydrous Na_2SO_4 and concentrated *in vacuo*. The residue was purified by column chromatography (silica gel, hexanes/EtOAc, 9:1) to afford (*S*)-Mosher ester **S15** (3.0 mg, 72%) as a colorless oil: ^{1}H NMR (500 MHz, $CDCl_3$) δ 7.53–7.46 (m, 2H), 7.45–7.38 (m, 3H), 5.39 (d, *J* = 1.8 Hz, 1H), 4.91 (q, *J* = 1.5 Hz, 1H), 4.69 (s, 1H), 4.68 (AB, J_{AB} = 7.5 Hz, $\Delta\nu_{AB}$ = 37.59 Hz, 2H), 3.79 (s, 3H), 3.53 (s, 3H), 3.35 (s, 3H), 3.11 (q, *J* = 8.6 Hz, 1H), 2.62 (dt, *J* = 7.3, 1.4 Hz, 1H), 1.92–1.82 (m, 1H), 1.74 (s, 3H), 1.68–1.57 (m, 3H), 1.24 (s, 3H).

Preparation of (*R*)-Mosher Ester S16

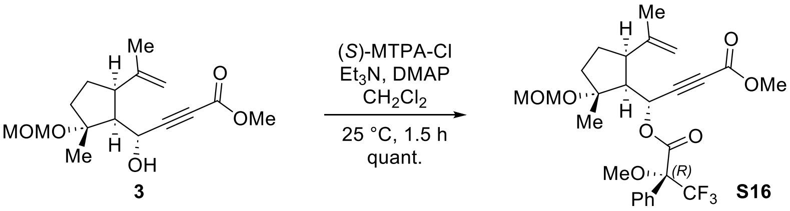

To a solution of **3** (2.6 mg, 0.0088 mmol) in anhydrous CH_2Cl_2 (0.25 mL) were added (*S*)-MTPA-Cl (16 μL, 0.088 mmol), Et_3N (24 μL, 0.18 mmol), and DMAP (10 crystals) 25 °C. After stirring at 25 °C for 2 h, the reaction was quenched with an addition of saturated aqueous $NaHCO_3$, and the resulting mixture was diluted with CH_2Cl_2. The layers were separated, and the aqueous layer was extracted with CH_2Cl_2. The combined organic layers were dried over anhydrous Na_2SO_4 and concentrated *in vacuo*. The residue was purified by column chromatography (silica gel,

hexanes/EtOAc, 9:1) to afford (*R*)-Mosher ester **S16** (5 mg, quant.) as a colorless oil: ^{1}H NMR (500 MHz, $CDCl_3$) δ 7.57–7.52 (m, 2H), 7.45–7.36 (m, 3H), 5.44 (d, J = 1.7 Hz, 1H), 4.87 (q, J = 1.5 Hz, 1H), 4.69 (AB, J_{AB} = 7.5 Hz, $\Delta\nu_{AB}$ = 41.44 Hz, 2H), 4.42 (s, 1H), 3.79 (s, 3H), 3.63 (s, 3H), 3.35 (s, 3H), 3.02 (dt, J = 11.4, 8.0 Hz, 1H), 2.59 (d, J = 7.1 Hz, 1H), 1.81 (ddd, J = 15.0, 9.4, 6.0 Hz, 1H), 1.69 (s, 3H), 1.54–1.51 (m, 1H), 1.50 (s, 3H), 1.37 (dtd, J = 13.1, 9.0, 4.4 Hz, 1H), 1.02 (qd, J = 12.1, 5.8 Hz, 1H).

6.2. Table S4. Comparative ^{1}H NMR Data for Mosher Esters (**S15** and **S16**)

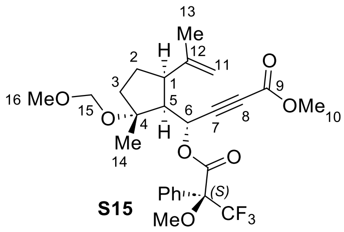

S15

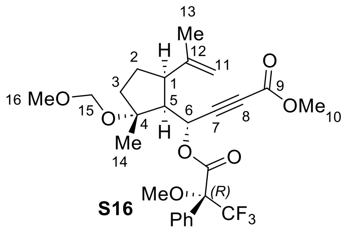

S16

Carbon No.	δ *S*-ester **S15** (ppm)	δ *R*-ester **S16** (ppm)	$\Delta\delta^{SR}$ (= $\delta_S - \delta_R$)	
			ppm	Hz (500 MHz)
1	3.11	3.02	+0.09	+45
5	2.62	2.59	+0.03	+15
10	3.79	3.79	0	0
11a	4.91	4.87	+0.04	+20
11b	4.69	4.42	+0.27	+135
13	1.74	1.69	+0.05	+25
14	1.24	1.50	-0.26	-130
15a	4.71	4.73	-0.02	-10
15b	4.64	4.65	-0.01	-5
16	3.35	3.35	0	0

7. Antifungal Activity of 1

7.1. Antifungal Susceptibility Testing

For the disc inhibition testing, strains were grown overnight in 5 mL YPD broth at 30 °C in a roller drum. Cells were pelleted by centrifugation, washed twice and then resuspended with sterile water. Cell density (OD600) was measured using a BioRad SmartSpec spectrophotometer and was adjusted to OD600 = 1, and 100 µL of cell suspension was spread on a plate using glass beads. The compound was dissolved in DMSO at a concentration of 100 mM (28.23 mg/mL), diluted in DMSO, and spotted on disc (Becton-Dickinson, 6 mm diameter) or directly onto the plate. The disc was then placed face-down on the agar plate prepared with the lawn of cells. *C. albicans* strain SC5314 was tested on YPD, SD, SDA, and YNB agar plates. DMSO only and Amphotericin B (4 µg) were also included as controls. Plates were incubated at 30 °C and 37 °C for 48 hours before images were taken.

The antifungal susceptibility testing was conducted using modified CLSI methods M27-A3 in 96-well microtiter plates (Falcon # 353072) with 200 µL total volume in each well. Strains were grown overnight in 5 mL YPD broth at 30 °C in a roller drum. Cells were pelleted by centrifugation, washed twice with sterile water, and resuspended in RPMI-1640 medium (Sigma-Aldrich). *C. albicans* strain SC5314 was inoculated at a starting density of OD600 = 0.0005 for each well. Two-fold serial dilutions of the compound were prepared and added to the wells to achieve final concentrations in the wells ranging between 16 µg/mL and 0.03125 µg/mL. Plates were incubated at 30 °C for 48 h, and growth was measured using a Molecular Devices iD3 Spectra plate reader, as well as visual scoring, to calculate minimal inhibitory concentration (MIC).

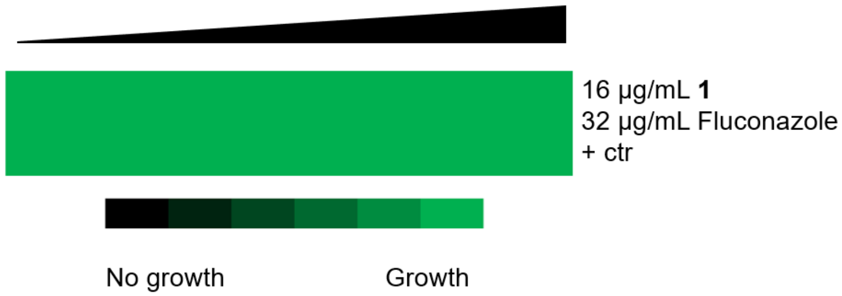

Figure S1. No antifungal efficacy of compound 1 seen against *C. albicans.* Broth microdilution assays were conducted in 96-well microtiter plates according to a modified CLSI protocol for yeasts in RPMI medium with MOPS and 2% glucose. Final drug concentrations in the wells ranged from 16 µg/mL and 0.03125 µg/mL. Plates were incubated at 30 °C for 48 h before measuring OD600 with a plate reader. Relative growth is displayed on a scale of no growth (black) to full growth without drugs added (green).

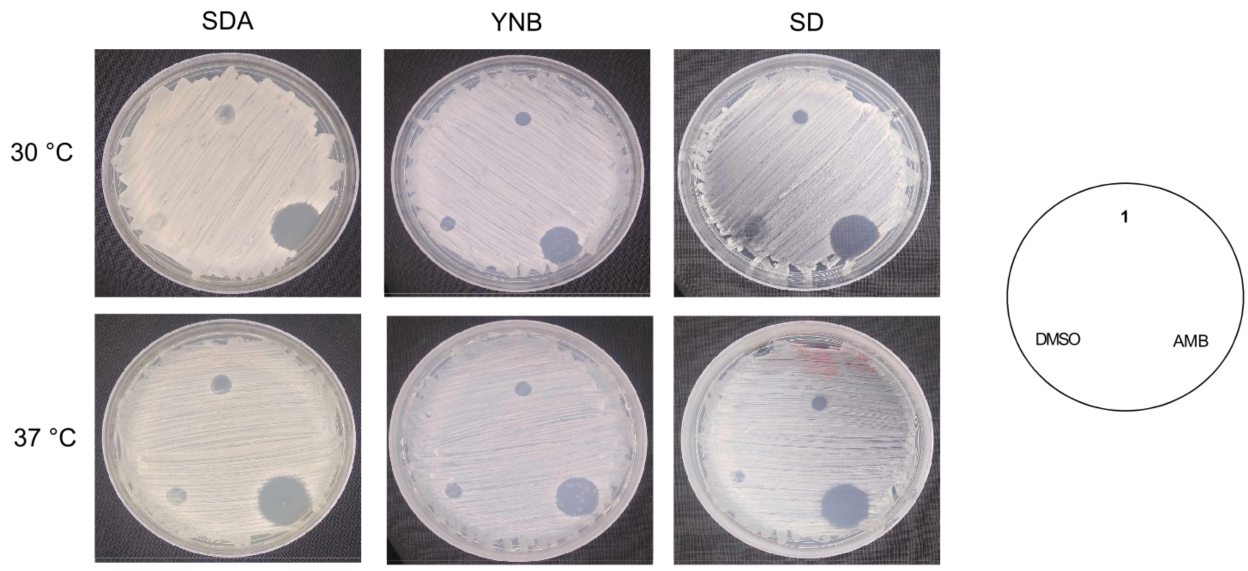

Figure S2. Compound 1 displays little to no observable activity against *C. albicans* SC5314 at 30 °C and 37 °C. Compound **1** was dissolved in DMSO and 10 µg was spotted onto each plate. DMSO and 4 µg Amphotericin B were used as controls. Each of the three drugs was spotted on SDA, YNB, or SD plates, which were incubated for 48 h at 30 °C or 37 °C. Compounds are arranged on each plate as follows. Top: compound **1**; lower left: DMSO; lower right: Amphotericin B.

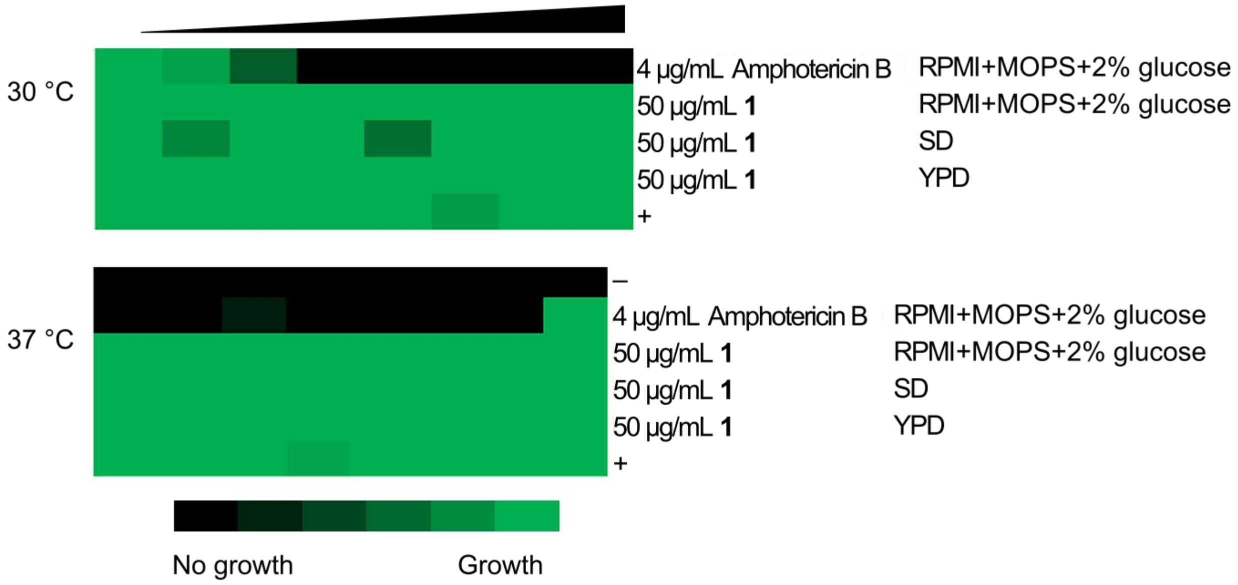

Figure S3. Compound 1 displays no detectable activity against *C. albicans* SC5314 under various temperature and growth media conditions. Broth microdilution assays were conducted according to modified CLSI protocols using RPMI+MOPS+2% glucose, SD, and YPD broth in 96-well microtiter plates. Plates were incubated at 30 °C or 37 °C for 48 h and read with a plate reader. Relative growth is displayed from black (0) to green coloration (full growth without drugs).

8. Comparison of Spectral Data of the Natural Product and Synthetic 1

(The ^{1}H and ^{13}NMR spectra of the natural product by Perveen *et al.* (Ref. #17) is licensed under CC BY 4.0)

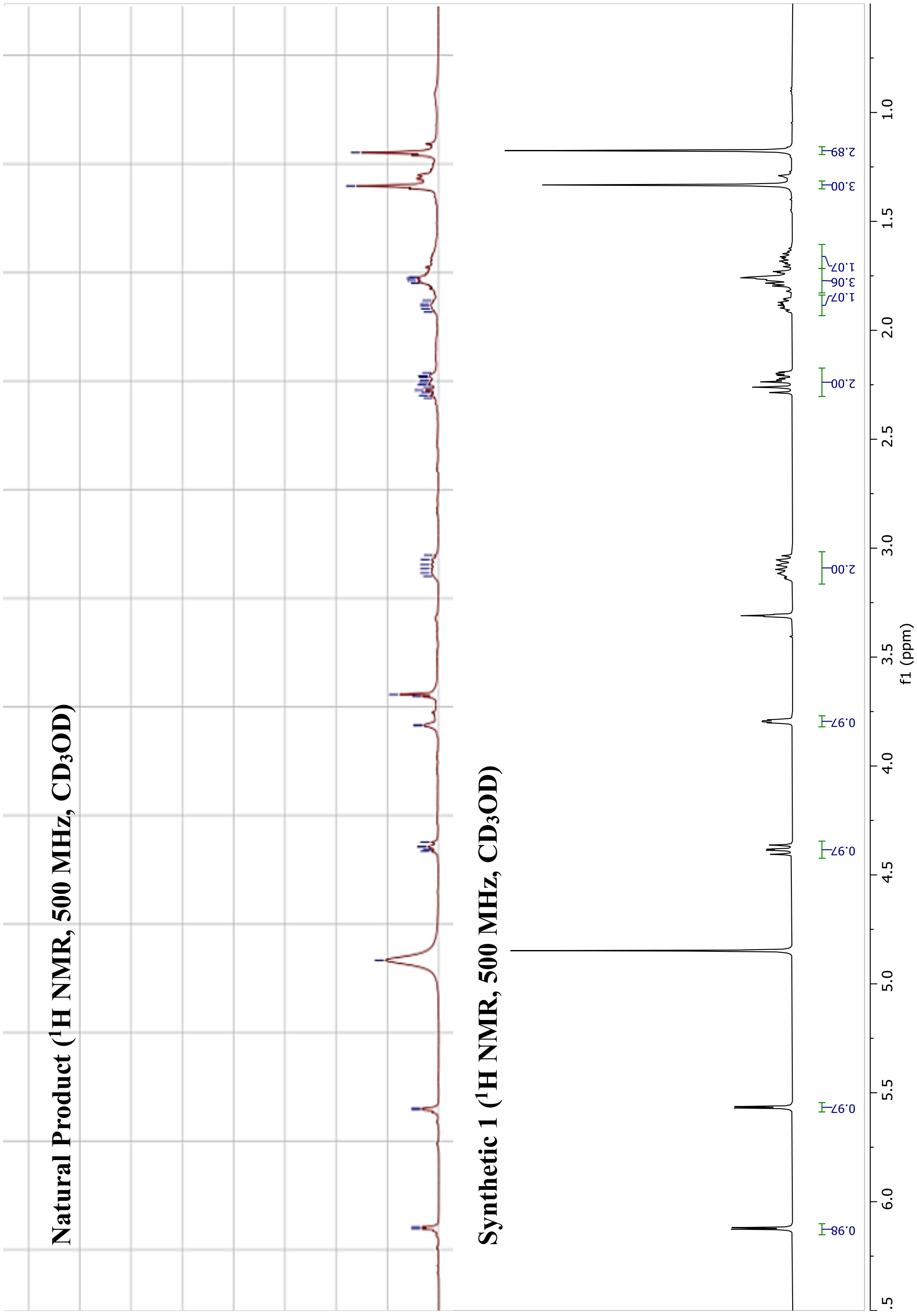

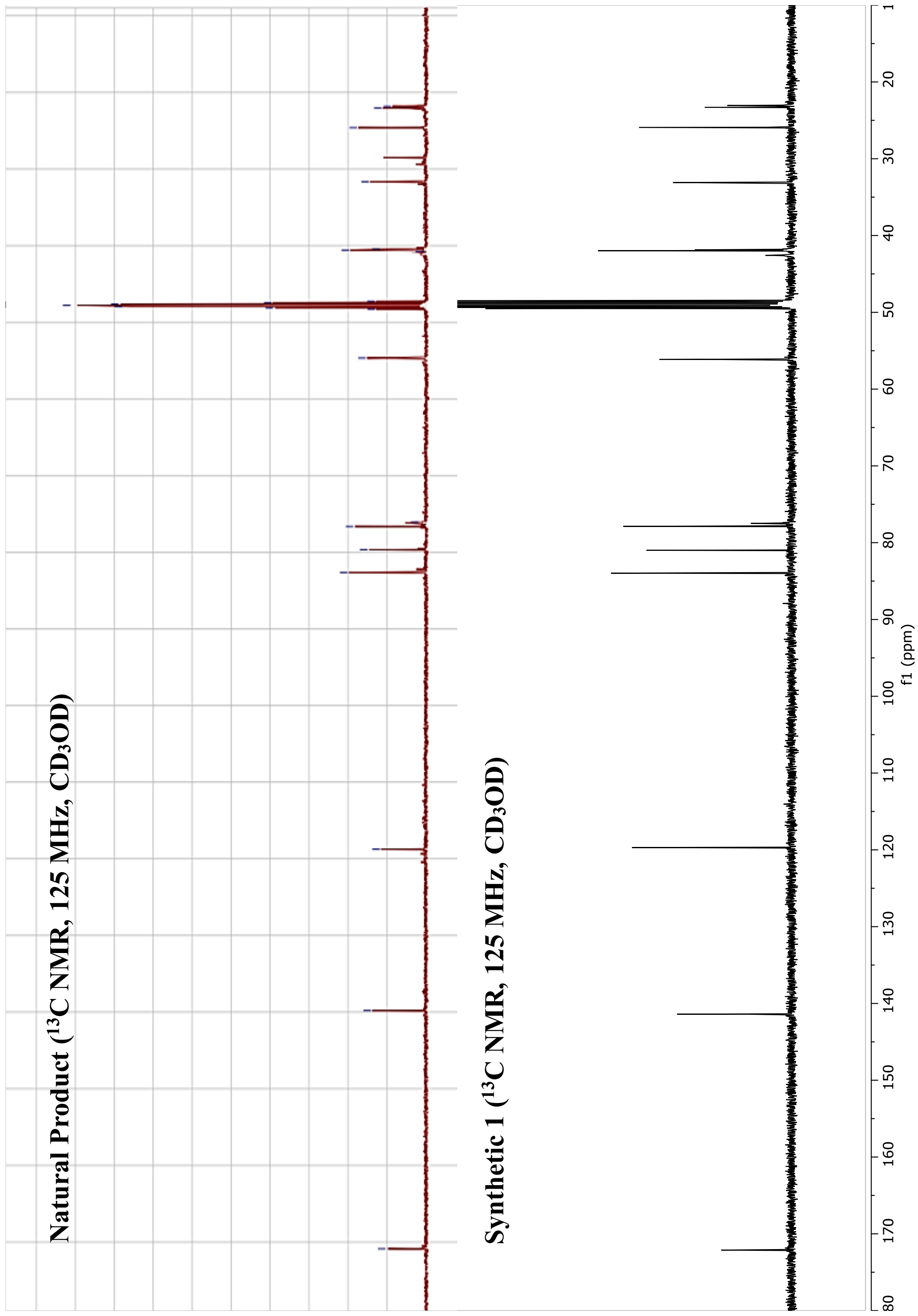
Natural Product (^{13}C NMR, 125 MHz, CD_3OD)
Synthetic 1 (^{13}C NMR, 125 MHz, CD_3OD)
f1 (ppm)

9. References

(1) Johnson, T. C.; Chin, M. R.; Han, T.; Shen, J. P.; Rana, T.; Siegel, D. Synthesis of Eupalinilide E, a Promoter of Human Hematopoietic Stem and Progenitor Cell Expansion. *J. Am. Chem. Soc.* **2016**, *138* (18), 6068–6073. DOI: 10.1021/jacs.6b03055

(2) Johnson, T. C.; Chin, M. R.; Siegel, D. Synthetic Route Development for the Laboratory Preparation of Eupalinilide E. *J. Org. Chem.* **2017**, *82* (9), 4640–4653. DOI: 10.1021/acs.joc.7b00266

(3) Carlson, R.; Behn, N.; Cowles, C. Epoxidation. III. The Relative Reactivities of Some Representative Olefins with Peroxybenzimidic Acid. *J. Org. Chem.* **1971**, *36* (24), 3832–3833. DOI: 10.1021/jo00823a615

(4) Binder, C. M.; Dixon, D. D.; Almaraz, E.; Tius, M. A.; Singaram, B. A Simple Procedure for C-C Bond Cleavage of Aromatic and Aliphatic Epoxides with Aqueous Sodium Periodate Under Ambient Conditions. *Tetrahedron Lett.* **2008**, *49* (17), 2764–2767. DOI: 10.1016/j.tetlet.2008.02.142

(5) Wolinsky, J.; Slabaugh, M. R.; Gibson, T. Synthesis of 4-(2-Methyl-5-isopropenyl-1-cyclopenten-1-yl)butan-2-one. A By-Product in the Synthesis of Pseudoionone. *J. Org. Chem.* **1964**, *29* (12), 3740–3742. DOI: 10.1021/jo01035a537

(6) Santos, R. B. d.; Cunha Neto, Á.; M Junior, E. d. S.; Lacerda Jr, V.; Greco, S. J.; Leite, J. d. A.; Faria, B. B. d.; Felippe, I. S. A. Synthesis and Determination of the Relative Stereochemistry of a New Epoxide Aldehyde Cyclopentane Monoterpenoid. *Quim. Nova* **2015**, *38*, 1260–1264. DOI: 10.5935/0100-4042.20150151

(7) Isayama, S.; Mukaiyama, T. A New Method for Preparation of Alcohols from Olefins with Molecular Oxygen and Phenylsilane by the Use of Bis(acetylacetonato)cobalt(II) *Chem. Lett.* **1989**, *18* (6), 1071–1074. DOI: 10.1246/cl.1989.1071

(8) Inoki, S.; Kato, K.; Isayama, S.; Mukaiyama, T. A New and Facile Method for the Direct Preparation of α-Hydroxycarboxylic Acid Esters from α,β-Unsaturated Carboxylic Acid Esters with Molecular Oxygen and Phenylsilane Catalyzed by Bis(dipivaloylmethanato)manganese(II) Complex. *Chem. Lett.* **1990**, *19* (10), 1869–1872. DOI: 10.1246/cl.1990.1869

(9) Schindler, C. S.; Stephenson, C. R.; Carreira, E. M. Enantioselective Synthesis of the Core of Banyaside, Suomilide, and Spumigin HKVV. *Angew. Chem. Int. Ed.* **2008**, *47* (46), 8852–8855. DOI: 10.1002/anie.200803655

(10) Iiyama, S.; Fukaya, K.; Yamaguchi, Y.; Watanabe, A.; Yamamoto, H.; Mochizuki, S.; Saio, R.; Noguchi, T.; Oishi, T.; Sato, T.; et al. Total Synthesis of Paclitaxel. *Org. Lett.* **2022**, *24* (1), 202–206. DOI: 10.1021/acs.orglett.1c03851

(11) Gampe, C. M.; Carreira, E. M. Cyclohexyne Cycloinsertion in the Divergent Synthesis of Guanacastepenes. *Chem. Eur. J.* **2012**, *18* (49), 15761–15771. DOI: 10.1002/chem.201202222

(12) Egger, J.; Bretscher, P.; Freigang, S.; Kopf, M.; Carreira, E. M. Synthesis of Epoxyisoprostanes: Effects in Reducing Secretion of Pro-inflammatory Cytokines IL-6 and IL-12. *Angew. Chem. Int. Ed.* **2013**, *52* (20), 5382–5385. DOI: 10.1002/anie.201300739
