# Supplementary material for: Asymmetric Total Synthesis of 4,9,10-Trihydroxyguaia-11(13)en-12,6-olide and Discovery of Its Anticancer Activity against Atypical Teratoid Rhabdoid Tumor
Source: ACS Cent Sci. 2025 Jun 3;11(7):1103–10. doi: 10.1021/acscentsci.5c00332 (PMC12291133; doi:10.1021/acscentsci.5c00332)
Supplement: Supplementary file 2 [file oc5c00332_si_002.pdf]

–Supporting Information: Part B-

Asymmetric Total Synthesis of 4,9,10-Trihydroxyguaia-11(13)en-12,6-olide and Discovery of Its Anticancer Activity Against Atypical Teratoid Rhabdoid Tumor

Hyejin Lee[1], Hongjun Jang[1,2], Hwan Myung[3,4], Angela Rivera[5], Anna F. Averette[5], Joseph Heitman[5,6], Jiyong Park[4,3], Deukjoon Kim[7], Hyoungsu Kim[2], and Jiyong Hong[1,6,*]

[1] Department of Chemistry, Duke University, Durham, North Carolina 27708, United States

[2] College of Pharmacy and Research Institute of Pharmaceutical Science and Technology (RIPST), Ajou University, Suwon 16499, Republic of Korea

[3] Department of Chemistry, Korea Advanced Institute of Science and Technology (KAIST), Daejeon 34141, Republic of Korea

[4] Center for Catalytic Hydrocarbon Functionalizations, Institute for Basic Science (IBS), Daejeon 34141, Republic of Korea

[5] Department of Molecular Genetics and Microbiology, Duke University School of Medicine, Durham, North Carolina 27710, United States

[6] Department of Pharmacology and Cancer Biology, Duke University School of Medicine, Durham, North Carolina 27710, United States

[7] College of Pharmacy, Seoul National University, Seoul 08826, Republic of Korea

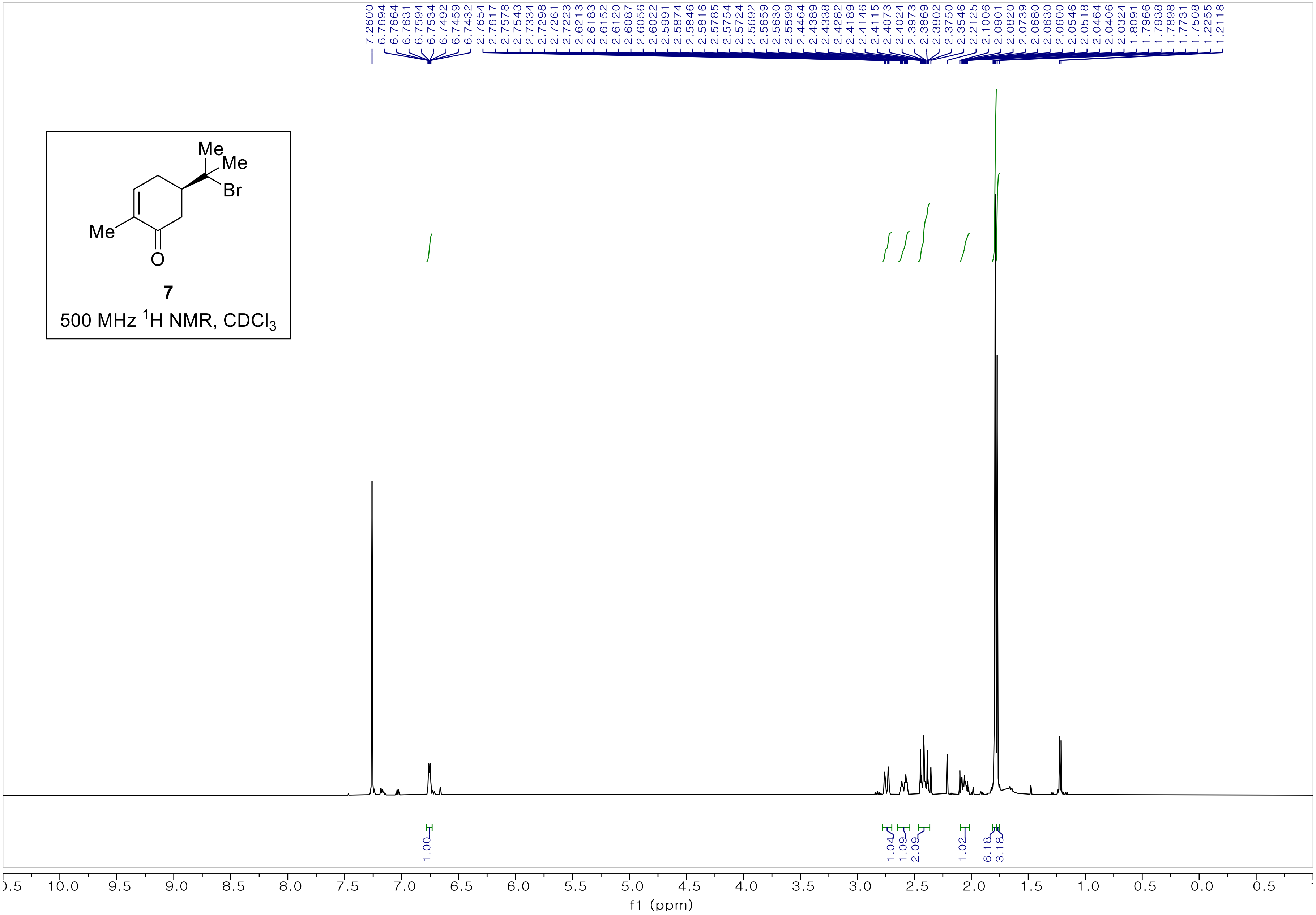
7.2600
6.7694
6.7664
6.7631
6.7594
6.7534
6.7492
6.7459
6.7432
2.7654
2.7617
2.7578
2.7543
2.7334
2.7298
2.7261
2.7223
2.6213
2.6183
2.6152
2.6120
2.6087
2.6056
2.6022
2.5991
2.5874
2.5846
2.5816
2.5785
2.5754
2.5724
2.5692
2.5659
2.5630
2.5599
2.4464
2.4389
2.4338
2.4282
2.4189
2.4146
2.4115
2.4073
2.4024
2.3973
2.3869
2.3802
2.3750
2.3546
2.2125
2.1006
2.0901
2.0820
2.0739
2.0680
2.0630
2.0600
2.0546
2.0518
2.0464
2.0406
2.0324
1.8091
1.7966
1.7938
1.7898
1.7731
1.7508
1.2255
1.2118
Me
Me
Br
Me
O
7
500 MHz 1H NMR, CDCl3
1.00
1.04
1.09
2.09
1.02
6.18
3.18
10.0
9.5
9.0
8.5
8.0
7.5
7.0
6.5
6.0
5.5
5.0
4.5
4.0
3.5
3.0
2.5
2.0
1.5
1.0
0.5
0.0
−0.5
f1 (ppm)

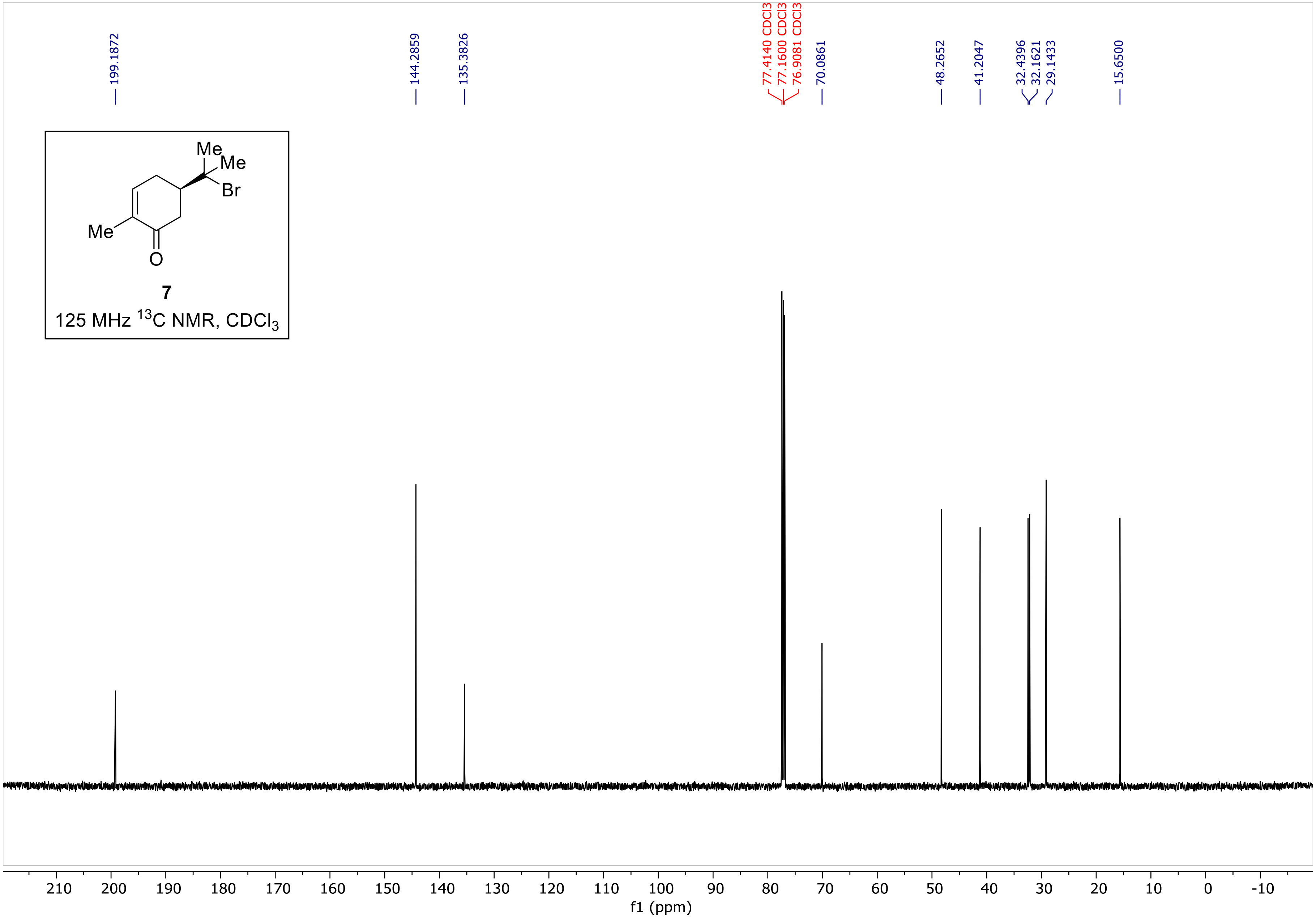
199.1872
144.2859
135.3826
77.4140 CDCl3
77.1600 CDCl3
76.9081 CDCl3
70.0861
48.2652
41.2047
32.4396
32.1621
29.1433
15.6500
Me
Me
Br
Me
O
7
125 MHz 13C NMR, CDCl3
210 200 190 180 170 160 150 140 130 120 110 100 90 80 70 60 50 40 30 20 10 0 -10
f1 (ppm)

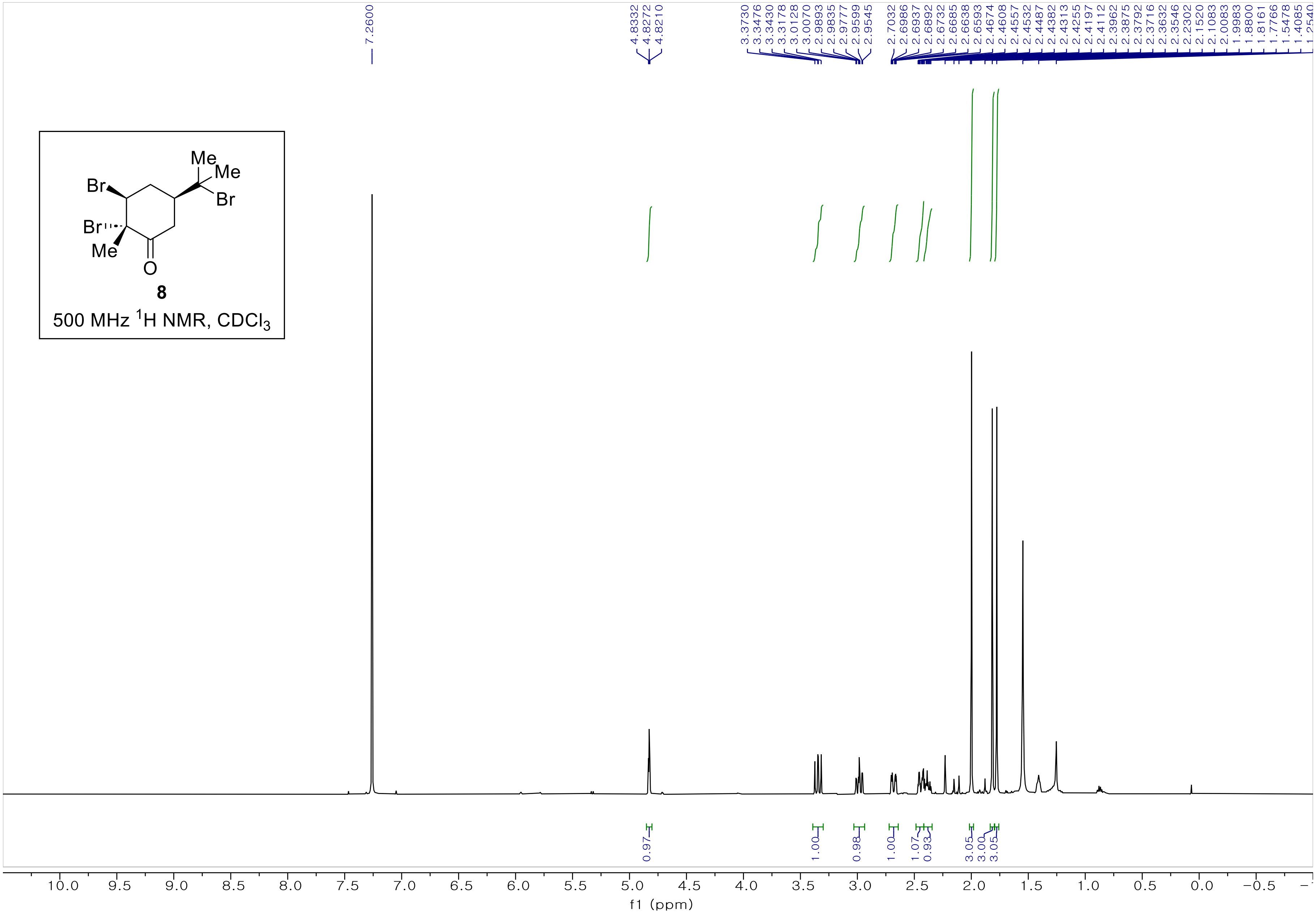
7.2600
4.8332
4.8272
4.8210
3.3730
3.3476
3.3430
3.3178
3.0128
3.0070
2.9893
2.9835
2.9777
2.9599
2.9545
2.7032
2.6986
2.6937
2.6892
2.6732
2.6685
2.6638
2.6593
2.4674
2.4608
2.4557
2.4532
2.4487
2.4382
2.4313
2.4255
2.4197
2.4112
2.3962
2.3875
2.3792
2.3716
2.3632
2.3546
2.2302
2.1520
2.1083
2.0083
1.9983
1.8800
1.8161
1.7766
1.5478
1.4085
Me
Me
Br
Br
Br
Me
O
8
500 MHz 1H NMR, CDCl3
0.97
1.00
0.98
1.00
1.07
0.93
3.05
3.00
3.05
10.0
9.5
9.0
8.5
8.0
7.5
7.0
6.5
6.0
5.5
5.0
4.5
4.0
3.5
3.0
2.5
2.0
1.5
1.0
0.5
0.0
−0.5
f1 (ppm)

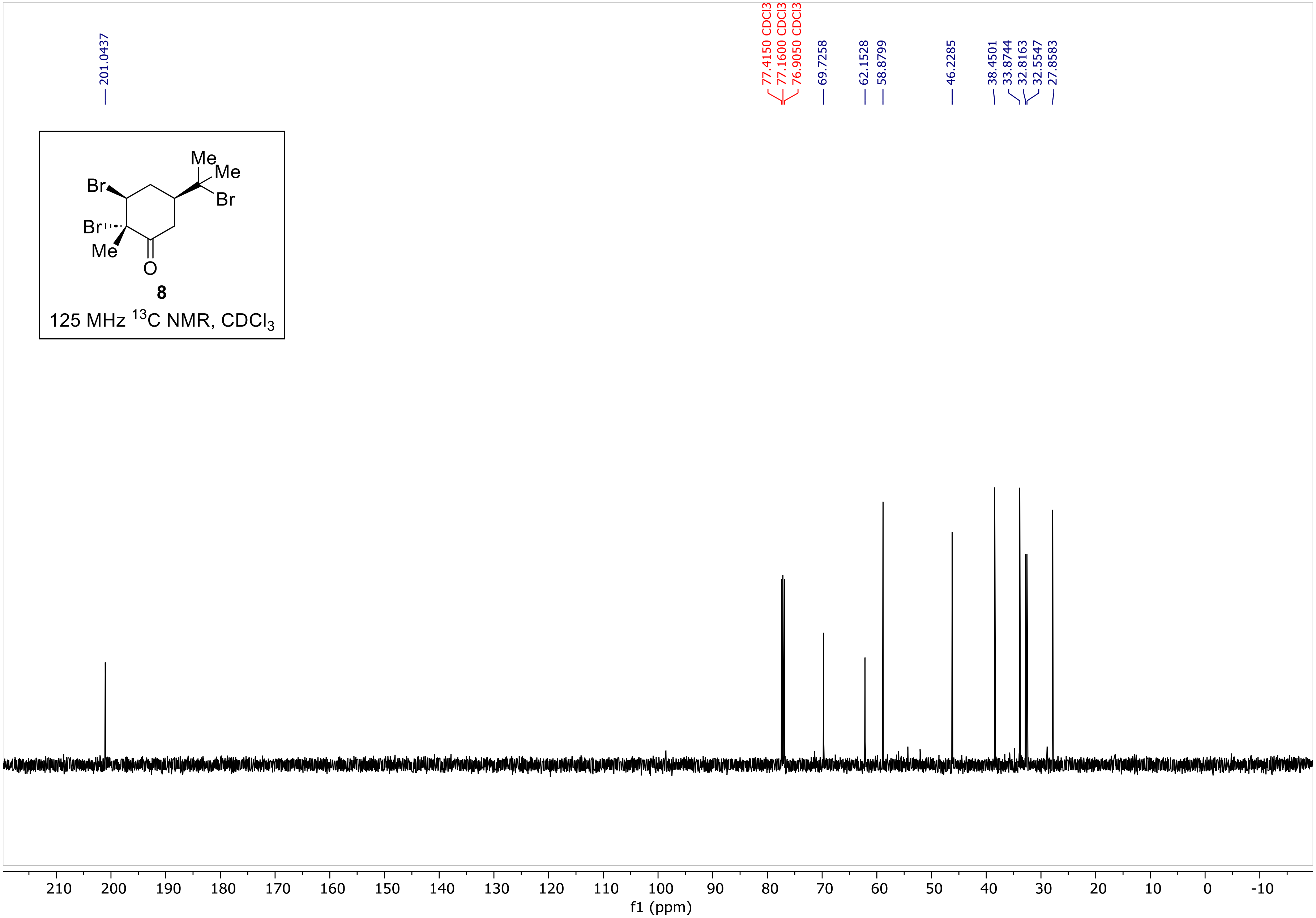
201.0437
77.4150 CDCl3
77.1600 CDCl3
76.9050 CDCl3
69.7258
62.1528
58.8799
46.2285
38.4501
33.8744
32.8163
32.5547
27.8583
Me
Me
Br
Br
Br
Me
O
8
125 MHz ^{13}C NMR, $CDCl_3$
210
200
190
180
170
160
150
140
130
120
110
100
90
80
70
60
50
40
30
20
10
0
-10
f1 (ppm)

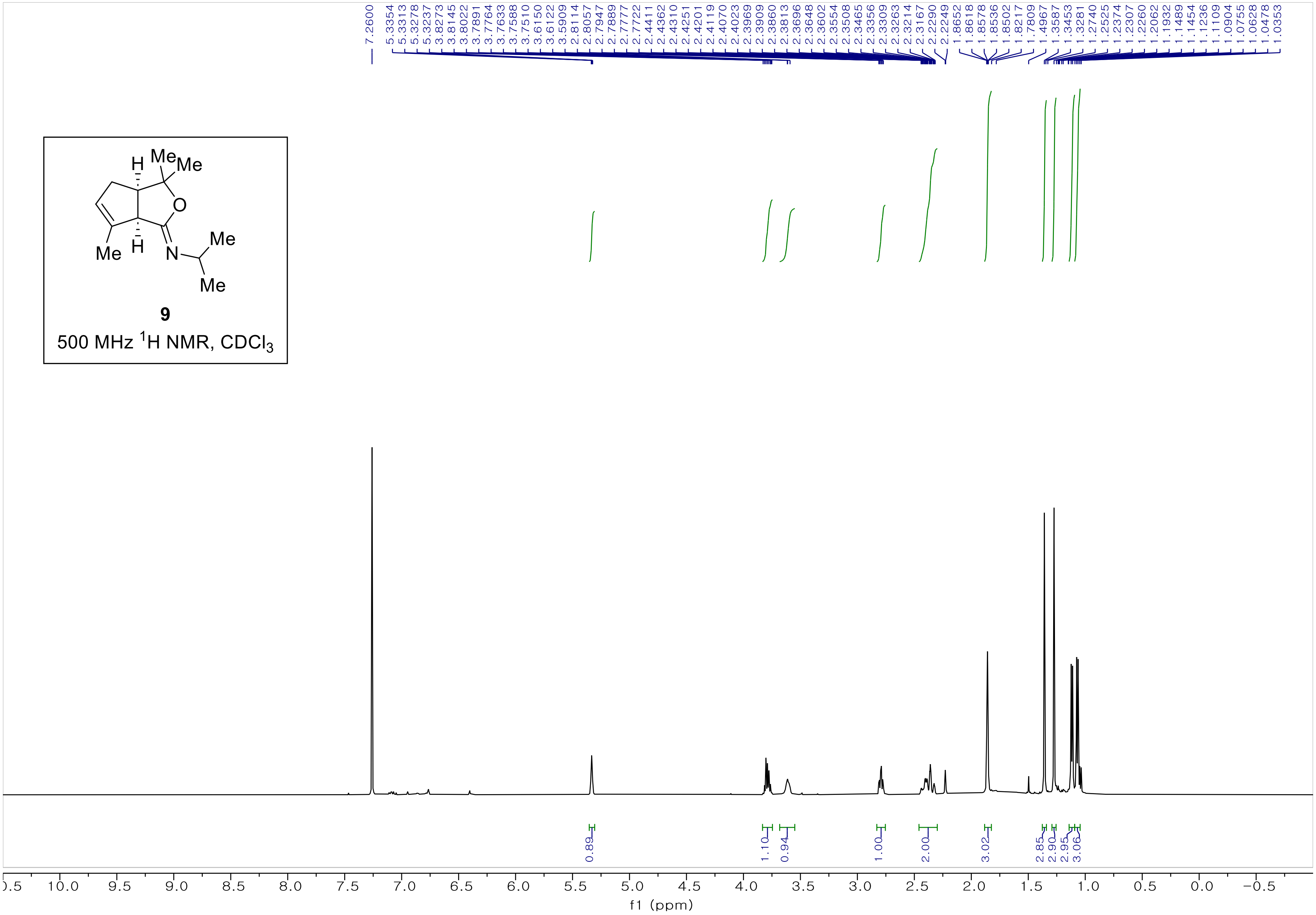

9
500 MHz ¹H NMR, CDCl₃
f1 (ppm)

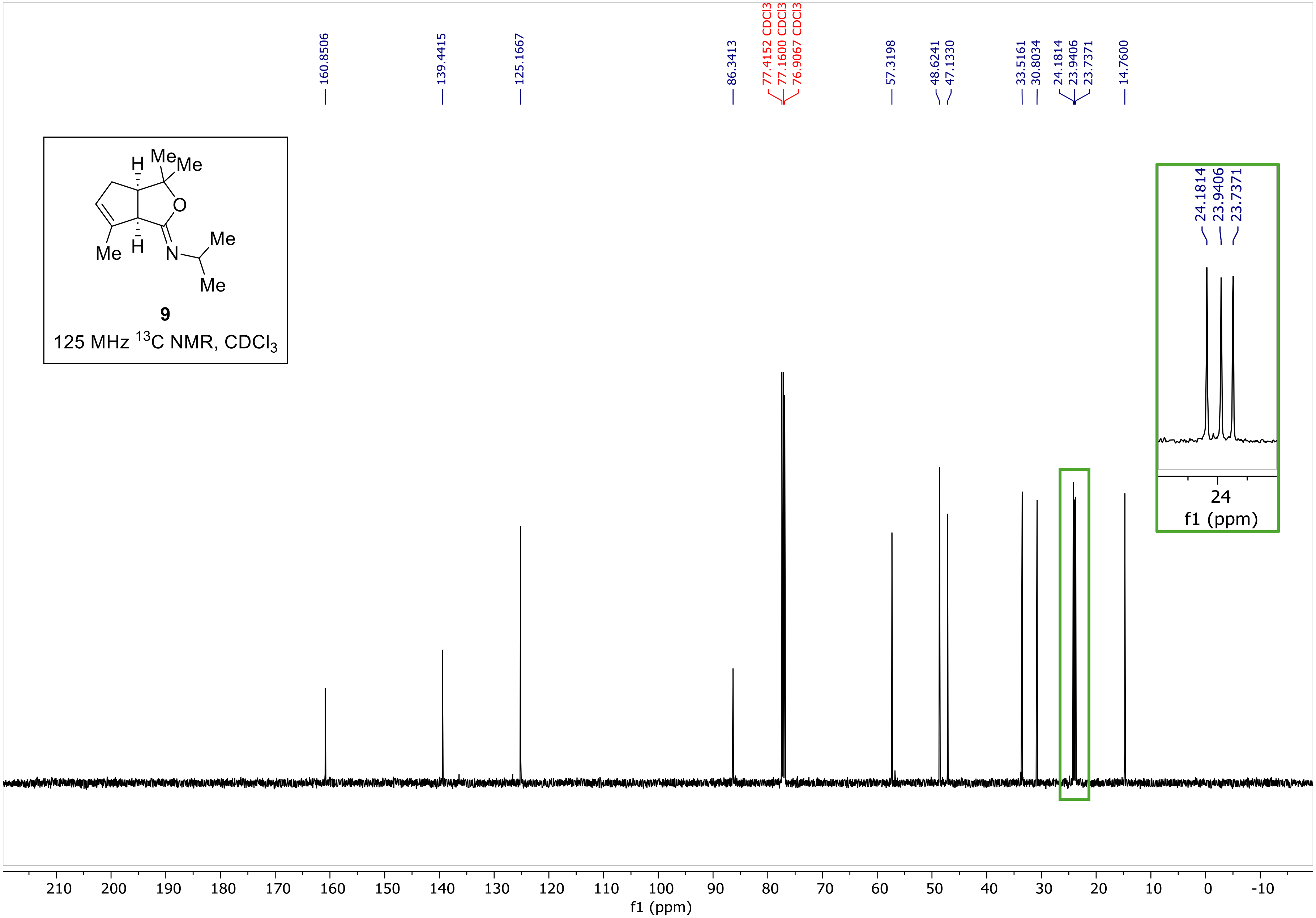
160.8506
139.4415
125.1667
86.3413
77.4152 CDCl3
77.1600 CDCl3
76.9067 CDCl3
57.3198
48.6241
47.1330
33.5161
30.8034
24.1814
23.9406
23.7371
14.7600
H
Me
Me
O
Me
N
Me
Me
H
9
125 MHz 13C NMR, CDCl3
24.1814
23.9406
23.7371
24
f1 (ppm)
210
200
190
180
170
160
150
140
130
120
110
100
90
80
70
60
50
40
30
20
10
0
-10
f1 (ppm)

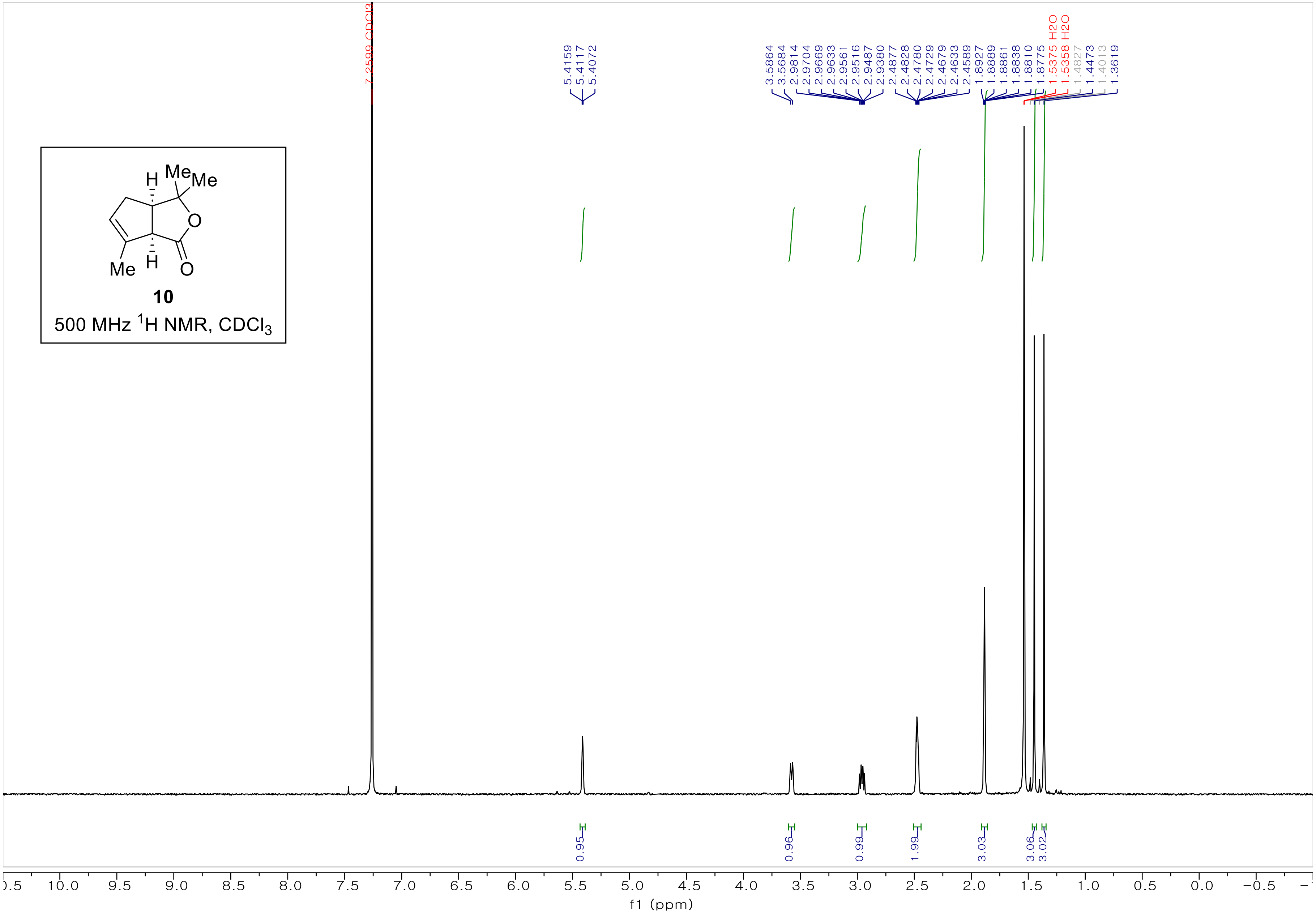

10
500 MHz ¹H NMR, CDCl₃
f1 (ppm)

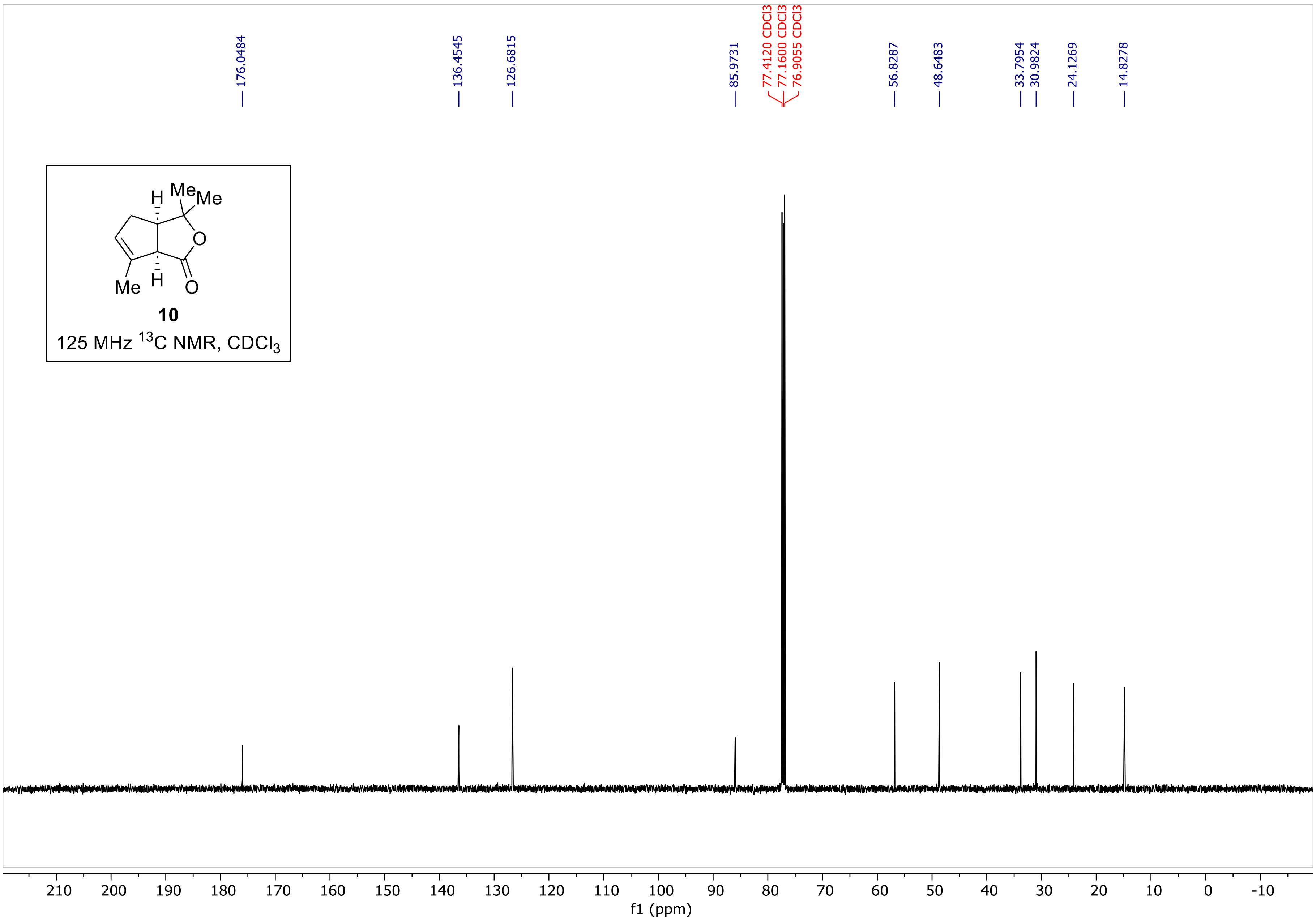
176.0484
136.4545
126.6815
85.9731
77.4120 CDCl3
77.1600 CDCl3
76.9055 CDCl3
56.8287
48.6483
33.7954
30.9824
24.1269
14.8278
H
Me
Me
O
O
Me
H
10
125 MHz 13C NMR, CDCl3
210 200 190 180 170 160 150 140 130 120 110 100 90 80 70 60 50 40 30 20 10 0 -10
f1 (ppm)

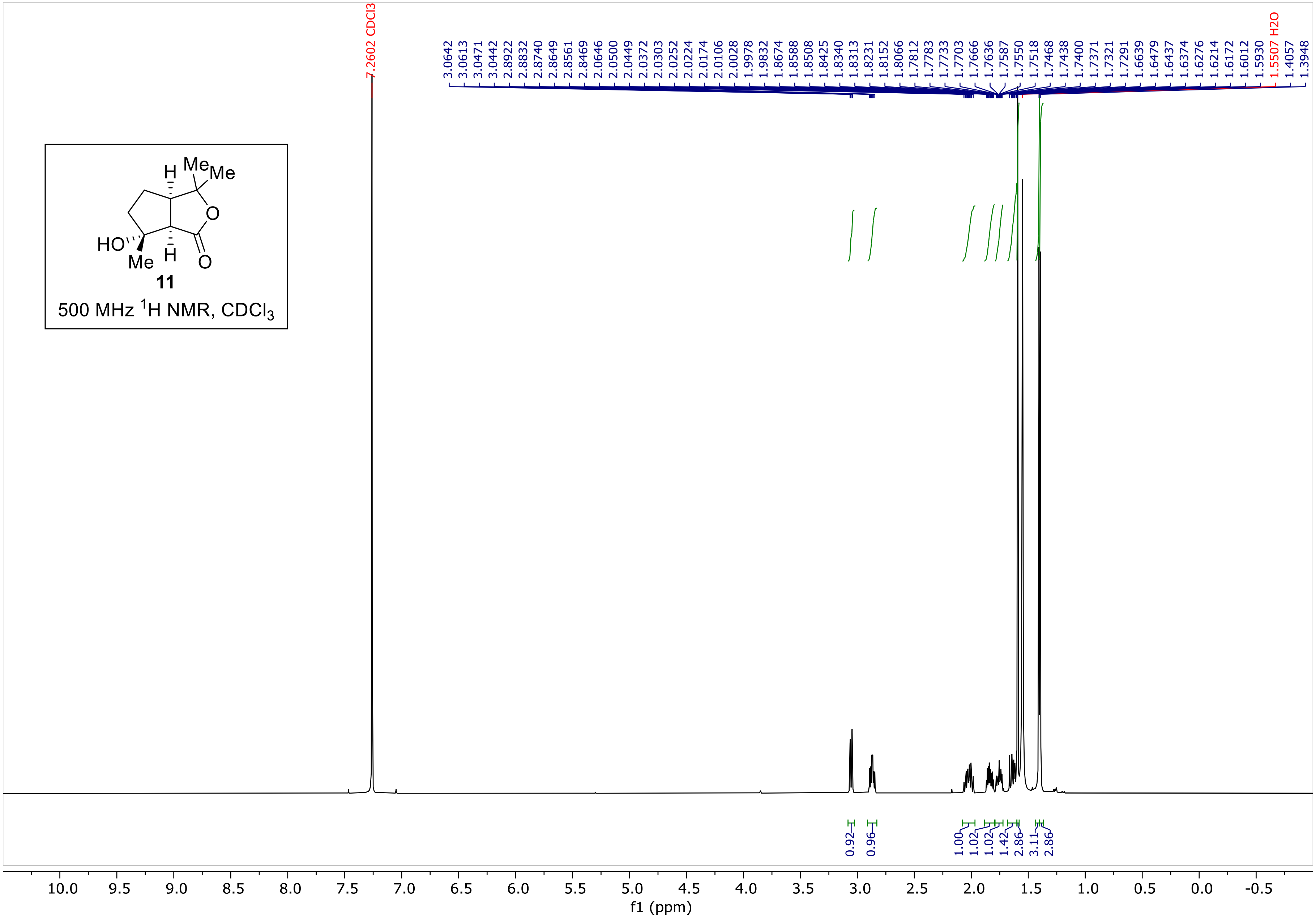

11
500 MHz 1H NMR, CDCl3
7.2602 CDCl3
3.0642
3.0613
3.0471
3.0442
2.8922
2.8832
2.8740
2.8649
2.8561
2.8469
2.0646
2.0500
2.0449
2.0372
2.0303
2.0252
2.0224
2.0174
2.0106
2.0028
1.9978
1.9832
1.8674
1.8588
1.8508
1.8425
1.8340
1.8313
1.8231
1.8152
1.8066
1.7812
1.7783
1.7733
1.7703
1.7666
1.7636
1.7587
1.7550
1.7518
1.7468
1.7438
1.7400
1.7371
1.7321
1.7291
1.6639
1.6479
1.6437
1.6374
1.6276
1.6214
1.6172
1.6012
1.5930
1.5507 H2O
1.4057
1.3948
0.92
0.96
1.00
1.02
1.02
1.42
2.86
3.11
2.86
f1 (ppm)

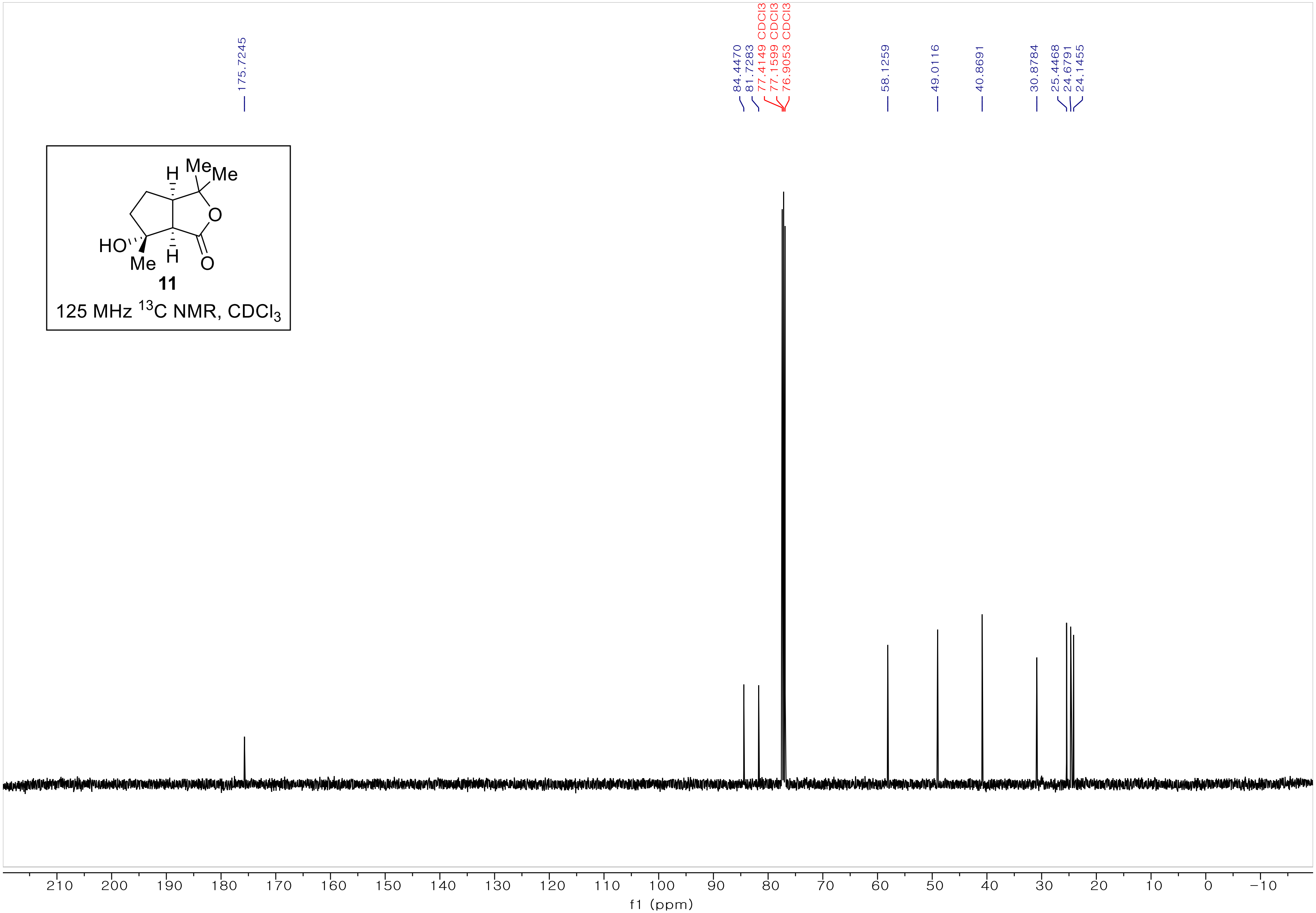
175.7245
84.4470
81.7283
77.4149 CDCl3
77.1599 CDCl3
76.9053 CDCl3
58.1259
49.0116
40.8691
30.8784
25.4468
24.6791
24.1455
11
125 MHz ^{13}C NMR, $CDCl_3$
f1 (ppm)

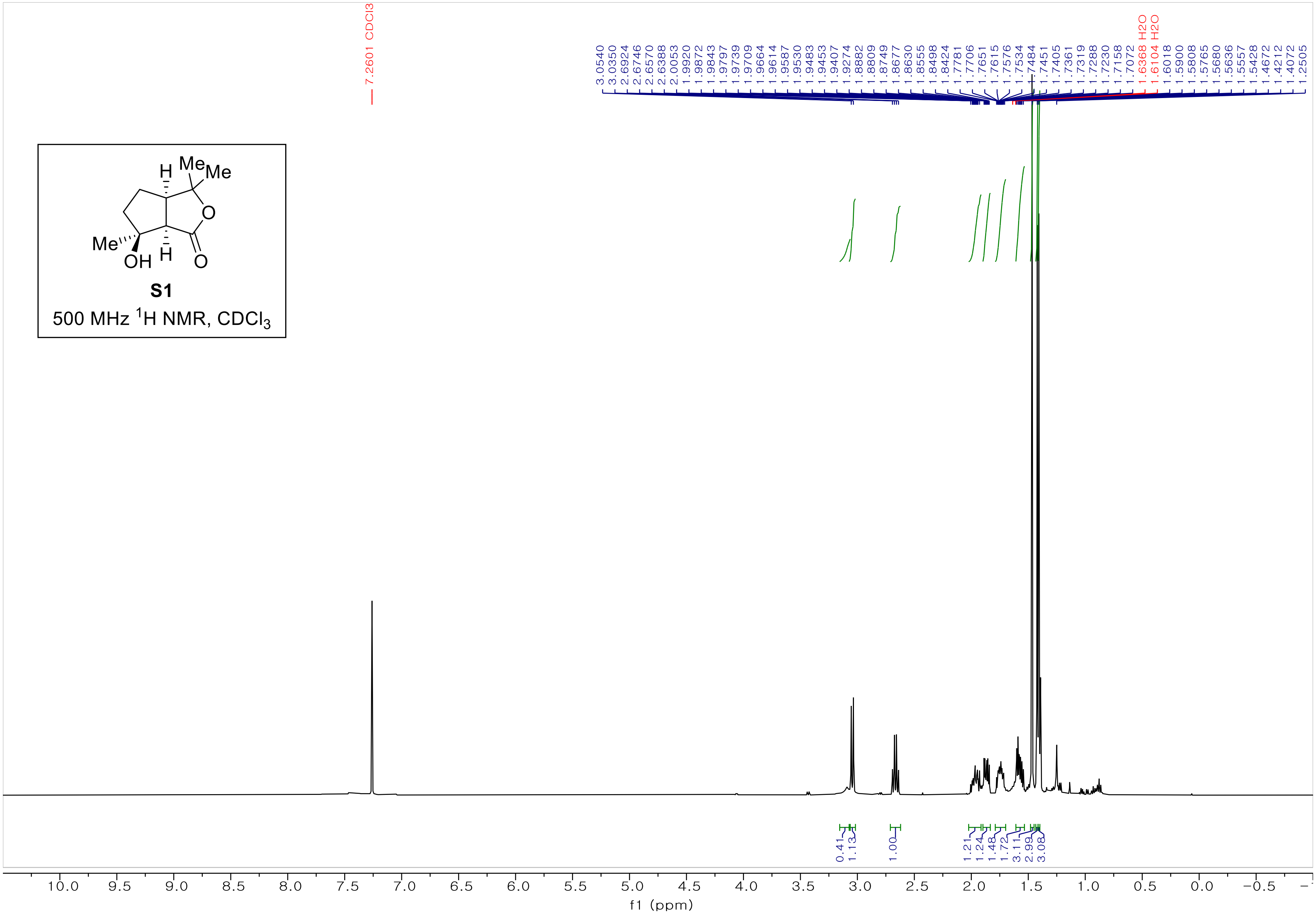
7.2601 CDCl3
1.6368 H2O
1.6104 H2O
S1
500 MHz ¹H NMR, CDCl₃
f1 (ppm)

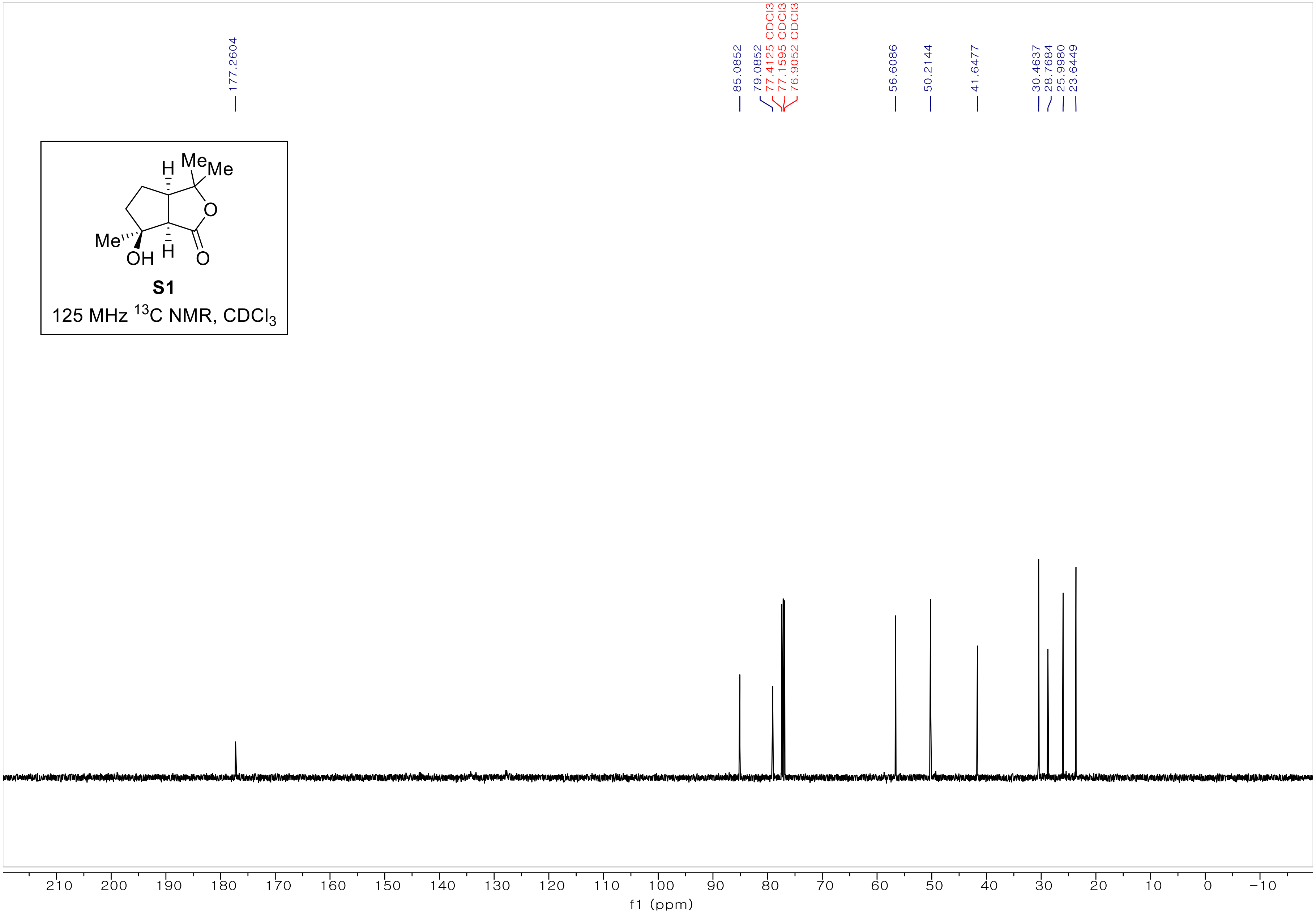
177.2604
85.0852
79.0852
77.4125 CDCl3
77.1595 CDCl3
76.9052 CDCl3
56.6086
50.2144
41.6477
30.4637
28.7684
25.9980
23.6449
S1
125 MHz 13C NMR, CDCl3
210 200 190 180 170 160 150 140 130 120 110 100 90 80 70 60 50 40 30 20 10 0 −10
f1 (ppm)

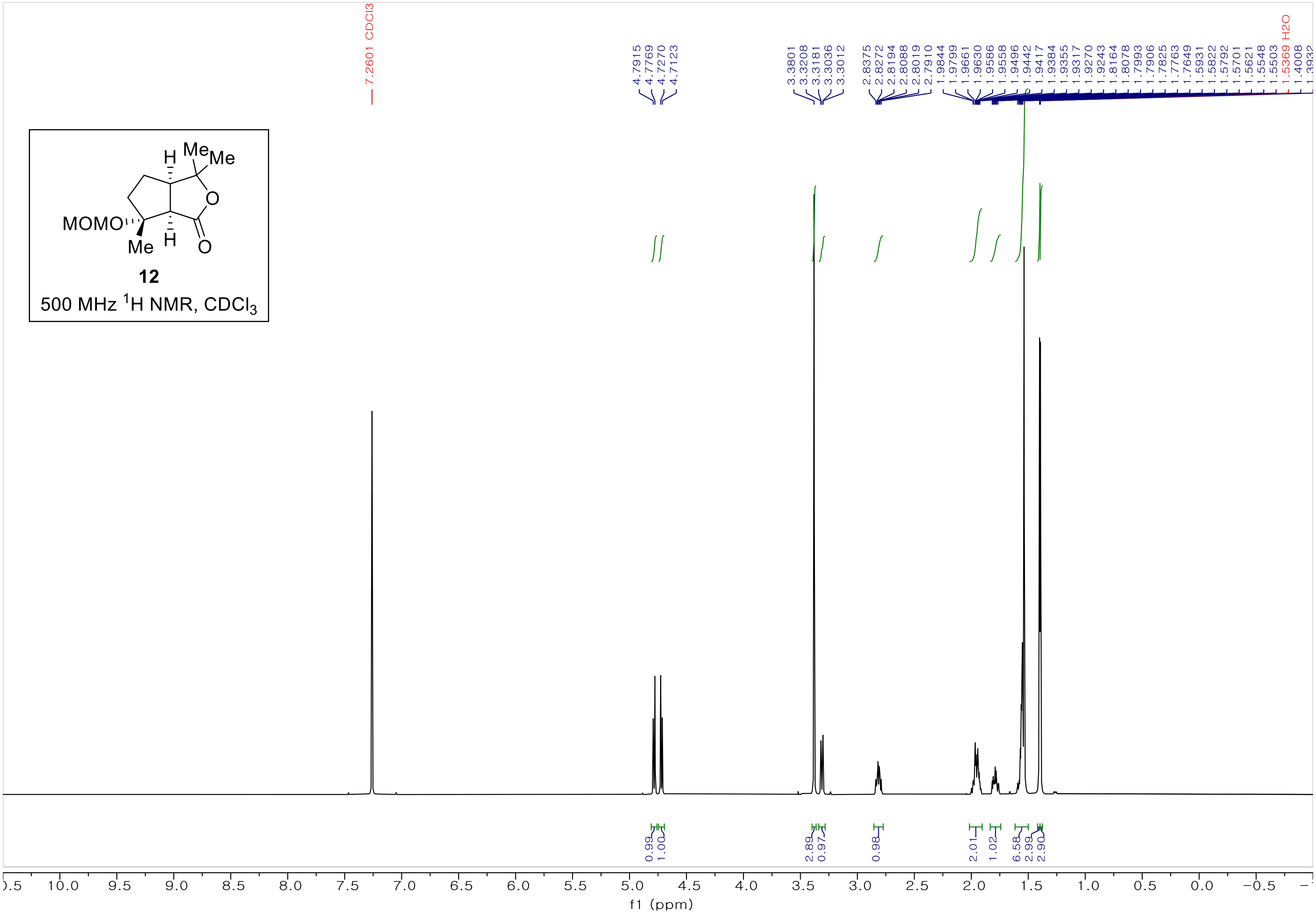
7.2601 CDCl3
4.7915
4.7769
4.7270
4.7123
3.3801
3.3208
3.3181
3.3036
3.3012
2.8375
2.8272
2.8194
2.8088
2.8019
2.7910
1.9844
1.9799
1.9661
1.9630
1.9586
1.9558
1.9496
1.9442
1.9417
1.9384
1.9355
1.9317
1.9270
1.9243
1.8164
1.8078
1.7993
1.7906
1.7825
1.7763
1.7649
1.5931
1.5822
1.5792
1.5701
1.5621
1.5548
1.5503
1.5369 H2O
1.4008
MOMO
Me
Me
Me
H
H
O
O
12
500 MHz 1H NMR, CDCl3
0.99
1.00
2.89
0.97
0.98
2.01
1.02
6.58
2.99
2.90
f1 (ppm)

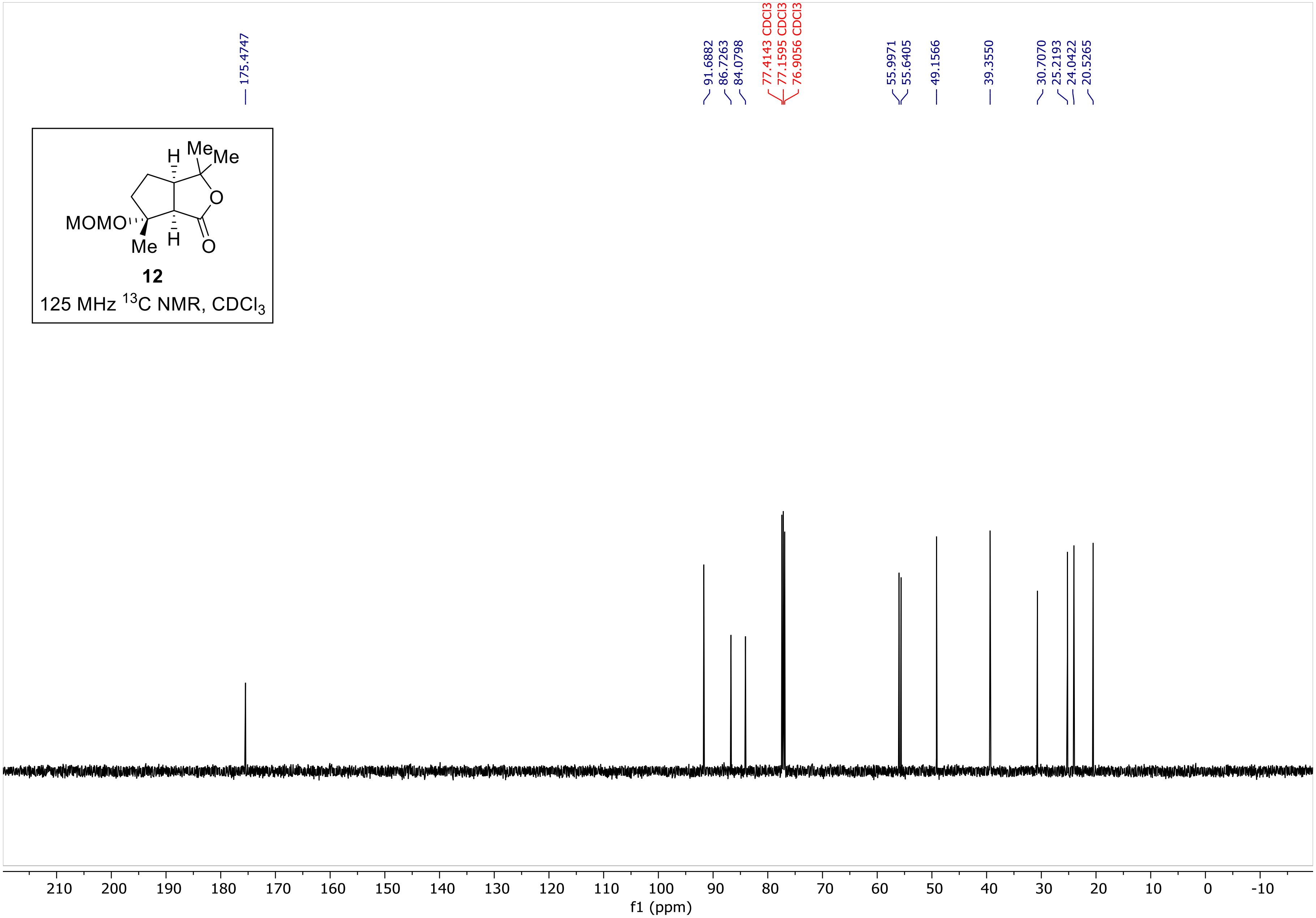
175.4747
91.6882
86.7263
84.0798
77.4143 CDCl3
77.1595 CDCl3
76.9056 CDCl3
55.9971
55.6405
49.1566
39.3550
30.7070
25.2193
24.0422
20.5265
H
Me
Me
O
MOMO
Me
H
O
12
125 MHz 13C NMR, CDCl3
210 200 190 180 170 160 150 140 130 120 110 100 90 80 70 60 50 40 30 20 10 0 -10
f1 (ppm)

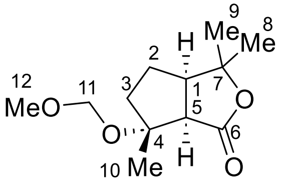

12

500 MHz ^{1}H-^{1}H NOESY NMR

$CDCl_3$

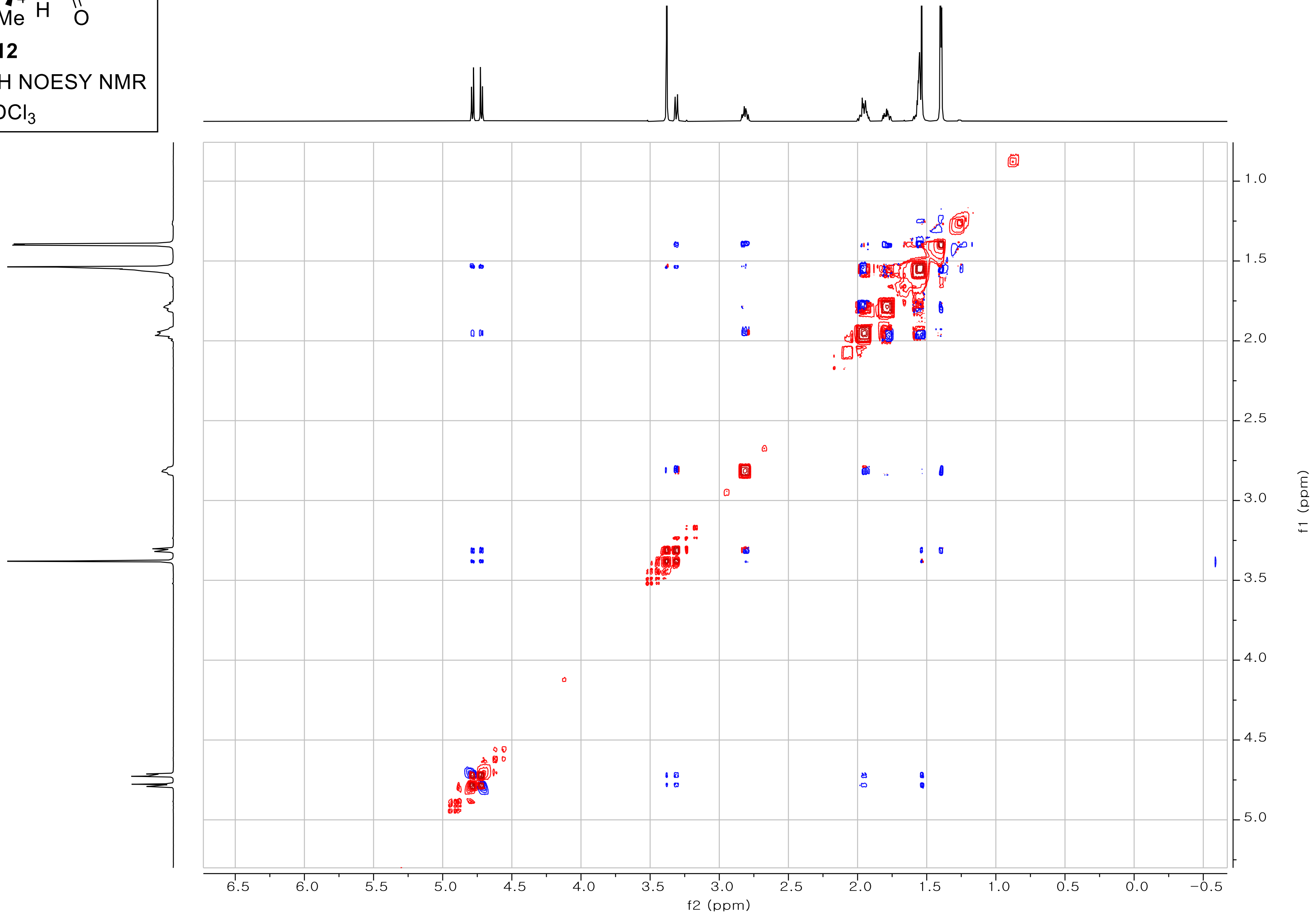

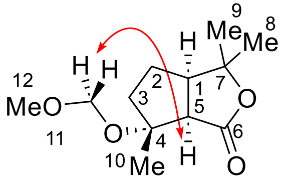

12

500 MHz ^{1}H-^{1}H NOESY NMR

$CDCl_3$

nOe: ←→

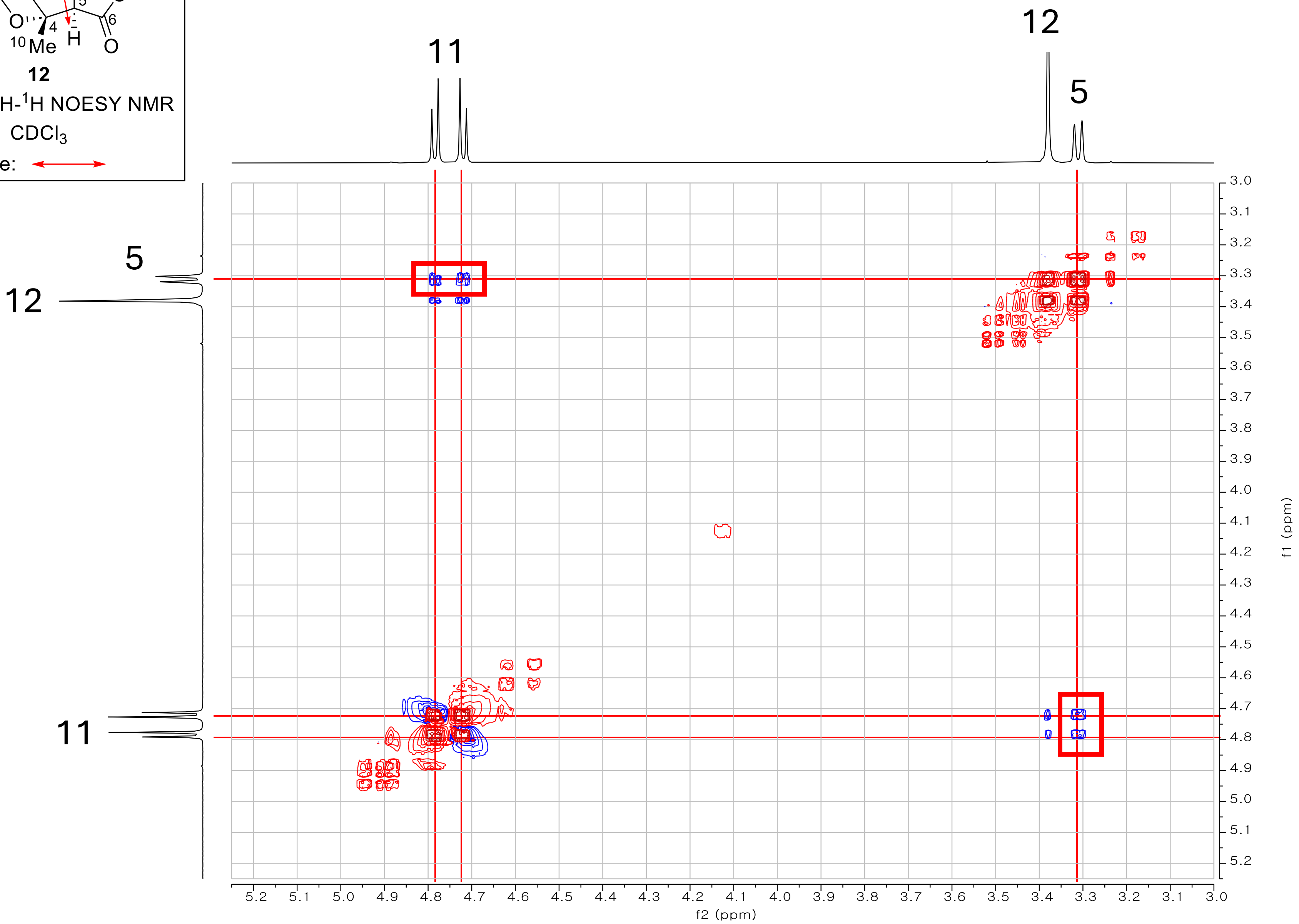

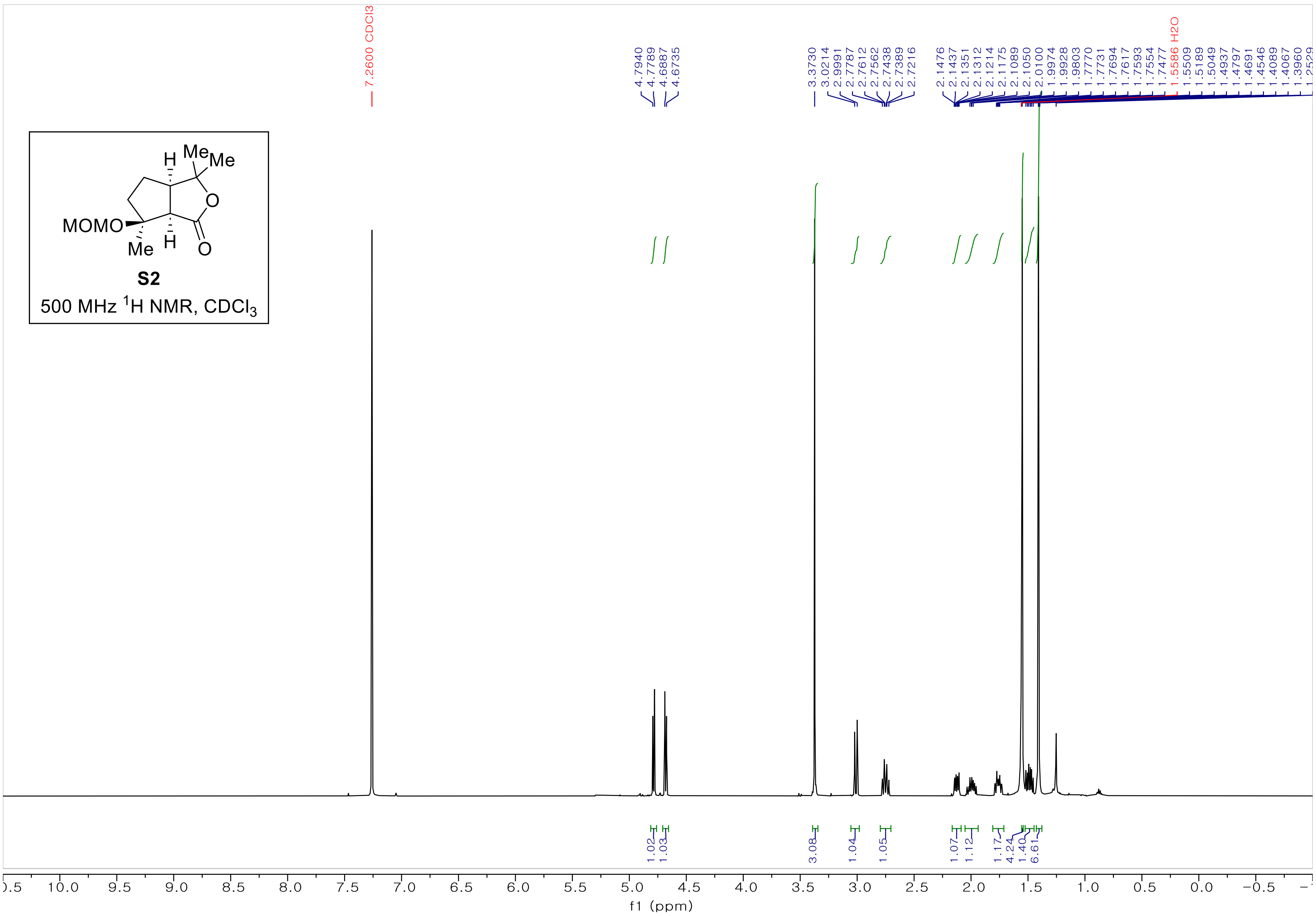

S2
500 MHz ^{1}H NMR, CDCl$_3$
MOMO
Me
H
O
7.2600 CDCl3
4.7940
4.7789
4.6887
4.6735
3.3730
3.0214
2.9991
2.7787
2.7612
2.7562
2.7438
2.7389
2.7216
2.1476
2.1437
2.1351
2.1312
2.1214
2.1175
2.1089
2.1050
2.0100
1.9974
1.9928
1.9803
1.7770
1.7731
1.7694
1.7617
1.7593
1.7554
1.7477
1.5586 H2O
1.5509
1.5189
1.5049
1.4937
1.4797
1.4691
1.4546
1.4089
1.4067
1.4060
1.3960
1.02
1.03
3.08
1.04
1.05
1.07
1.12
1.17
4.24
1.40
6.61
f1 (ppm)

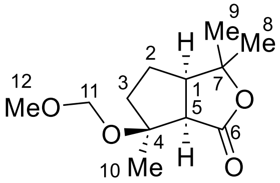

S2

500 MHz ^{1}H-^{1}H NOESY NMR

$CDCl_3$

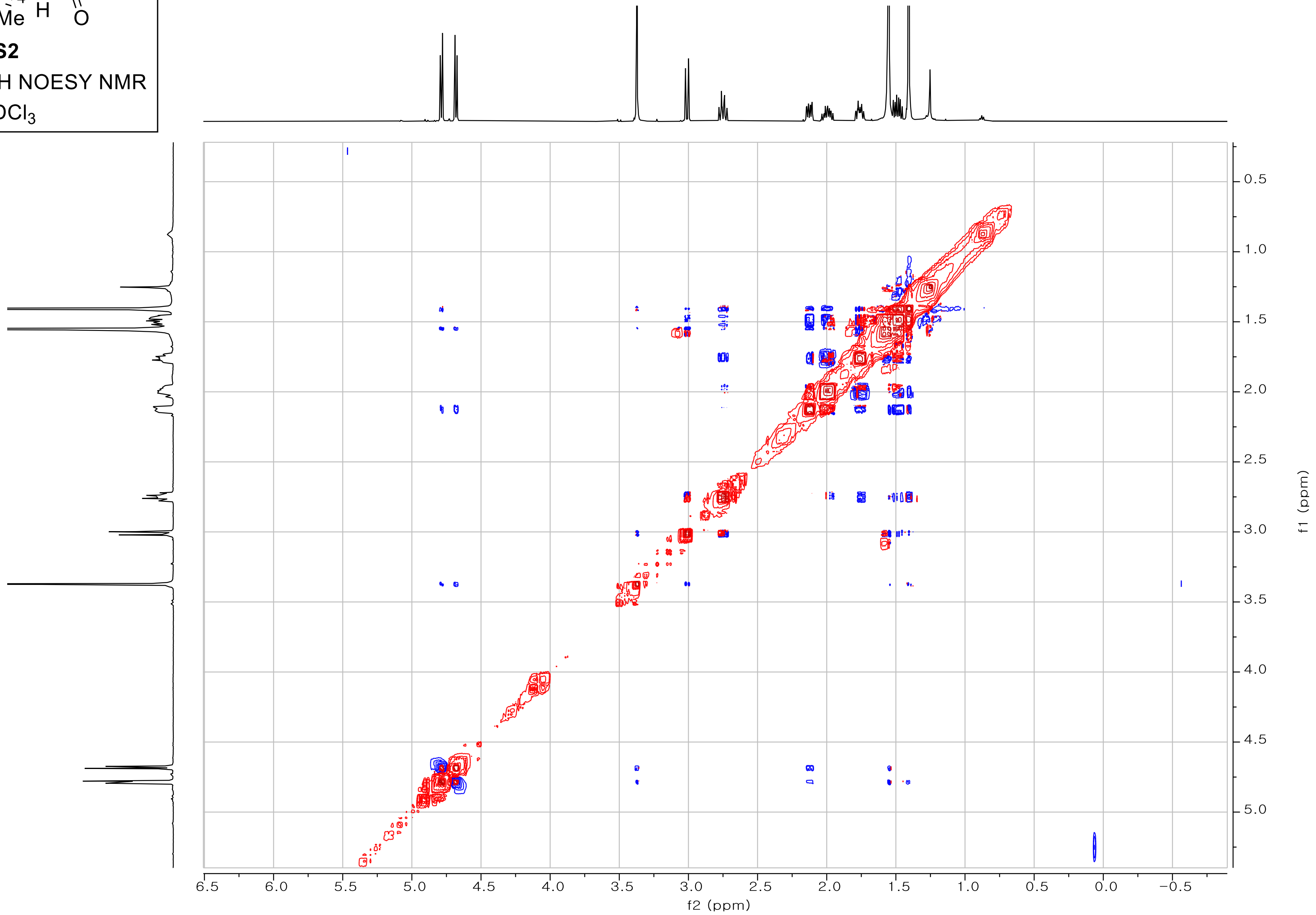

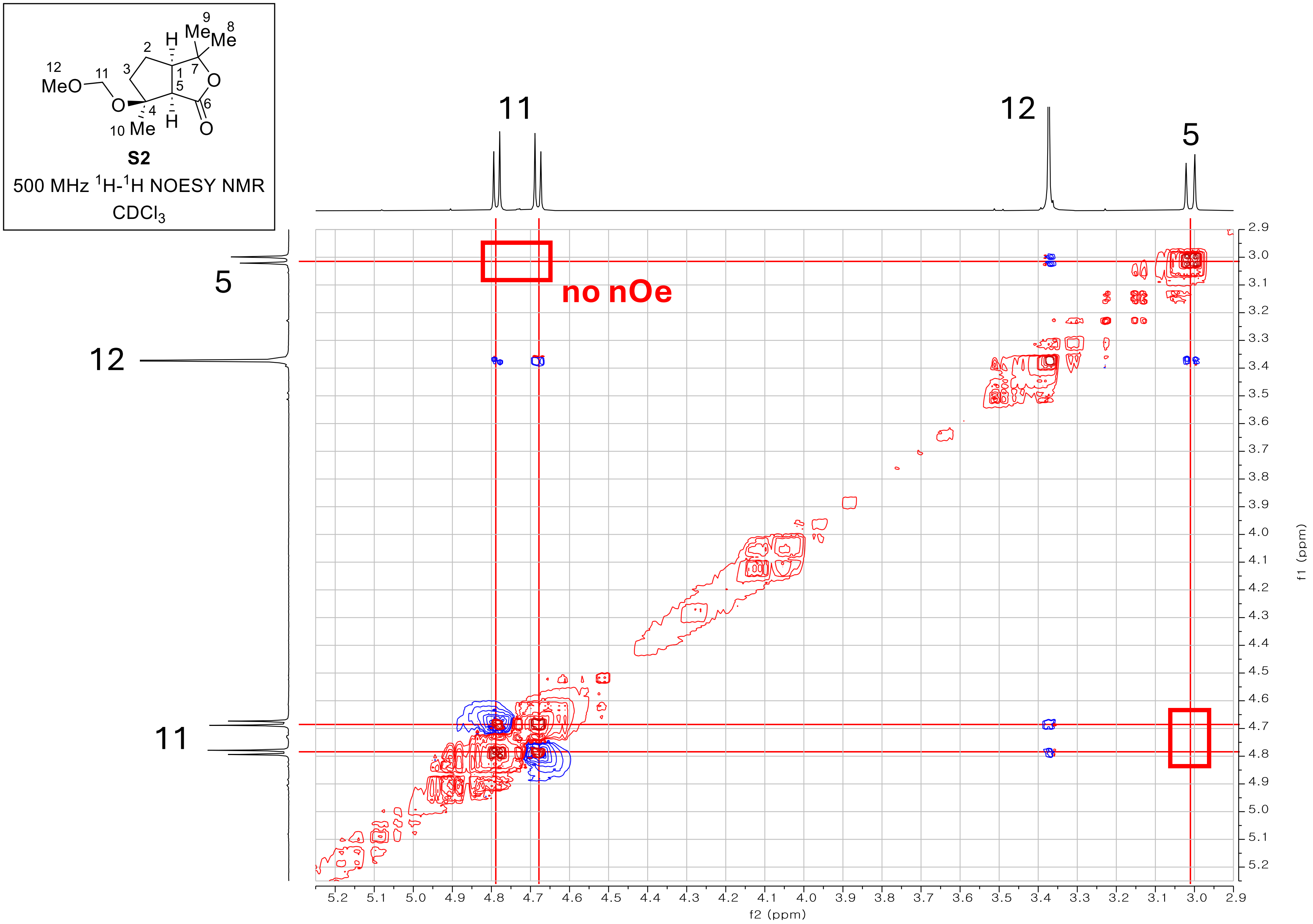

S2
500 MHz ¹H-¹H NOESY NMR
CDCl3
11
12
5
no nOe
f2 (ppm)
f1 (ppm)

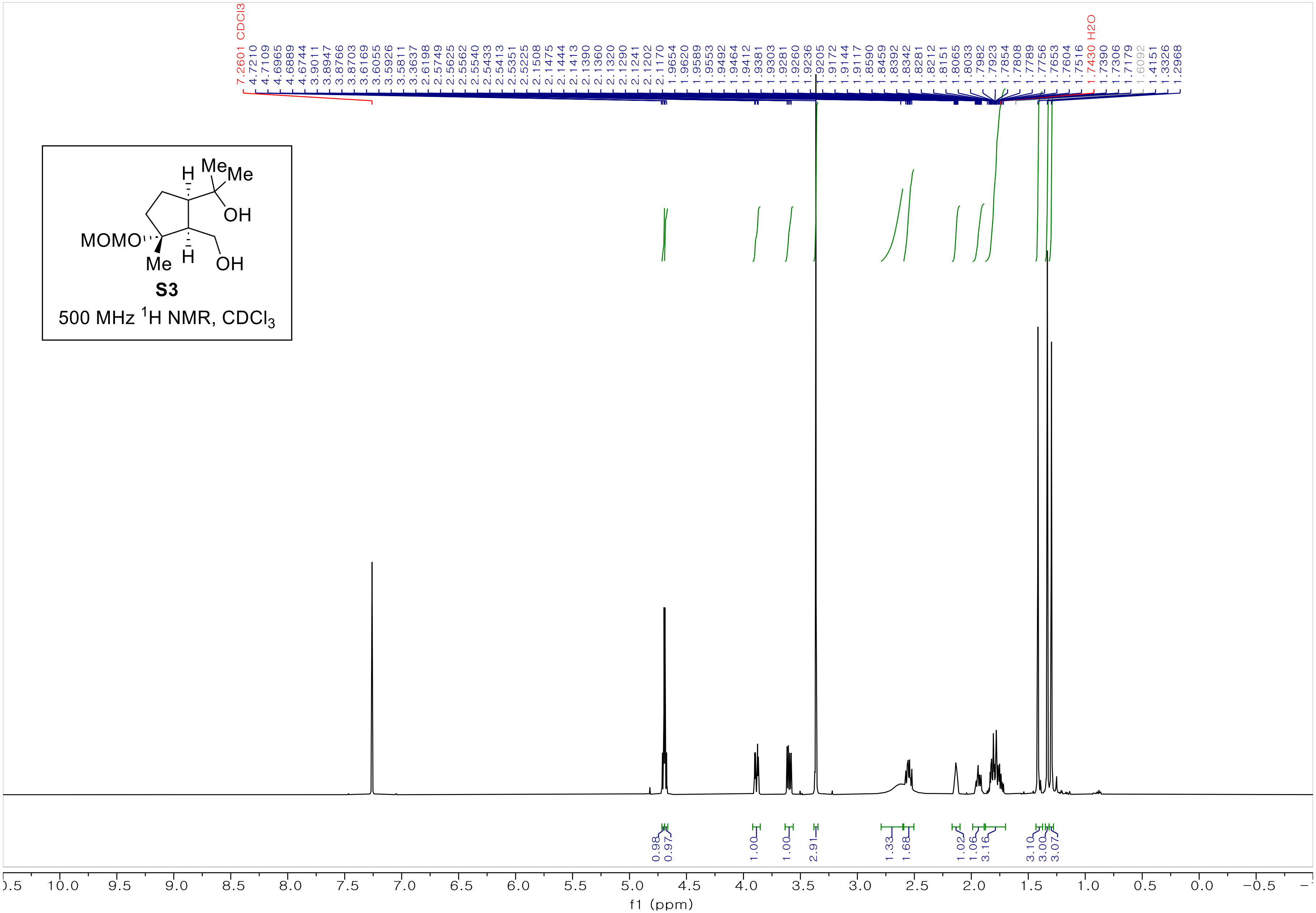

S3
500 MHz ¹H NMR, CDCl3
f1 (ppm)

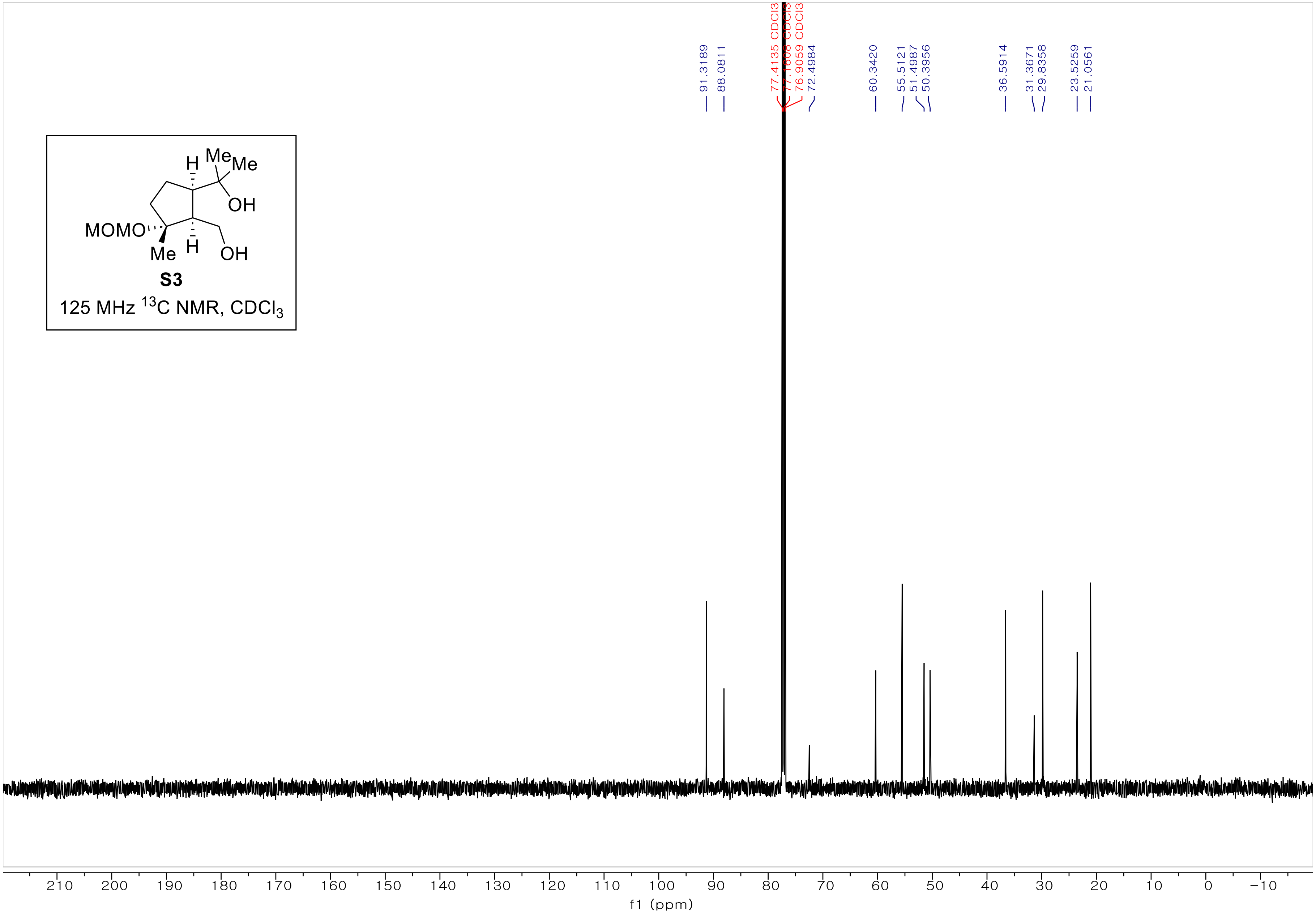
91.3189
88.0811
77.4135 CDCl3
77.1608 CDCl3
76.9059 CDCl3
72.4984
60.3420
55.5121
51.4987
50.3956
36.5914
31.3671
29.8358
23.5259
21.0561
MOMO
Me
H
Me
Me
OH
OH
S3
125 MHz ^{13}C NMR, $CDCl_3$
210 200 190 180 170 160 150 140 130 120 110 100 90 80 70 60 50 40 30 20 10 0 −10
f1 (ppm)

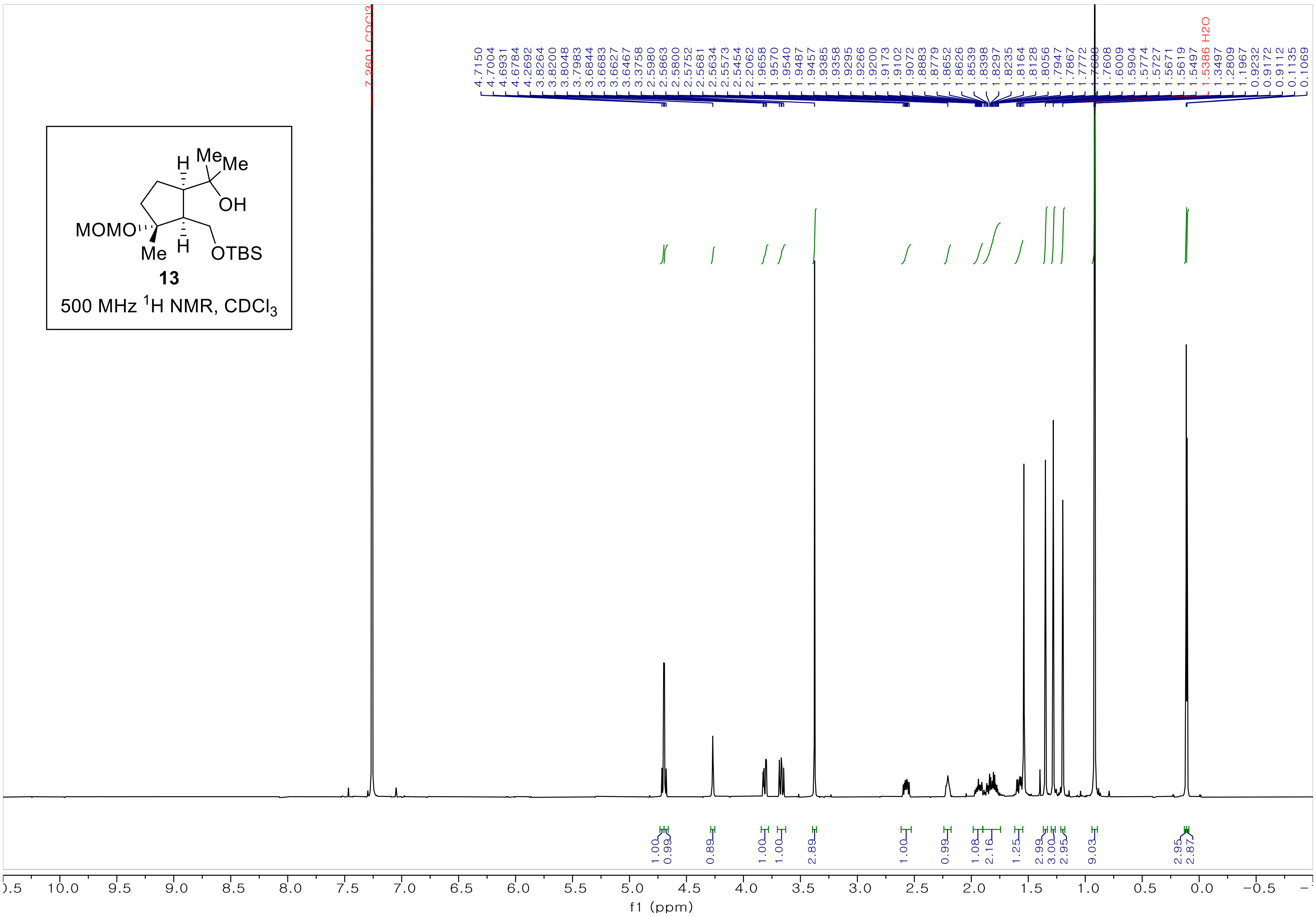
13
500 MHz ¹H NMR, CDCl3
f1 (ppm)

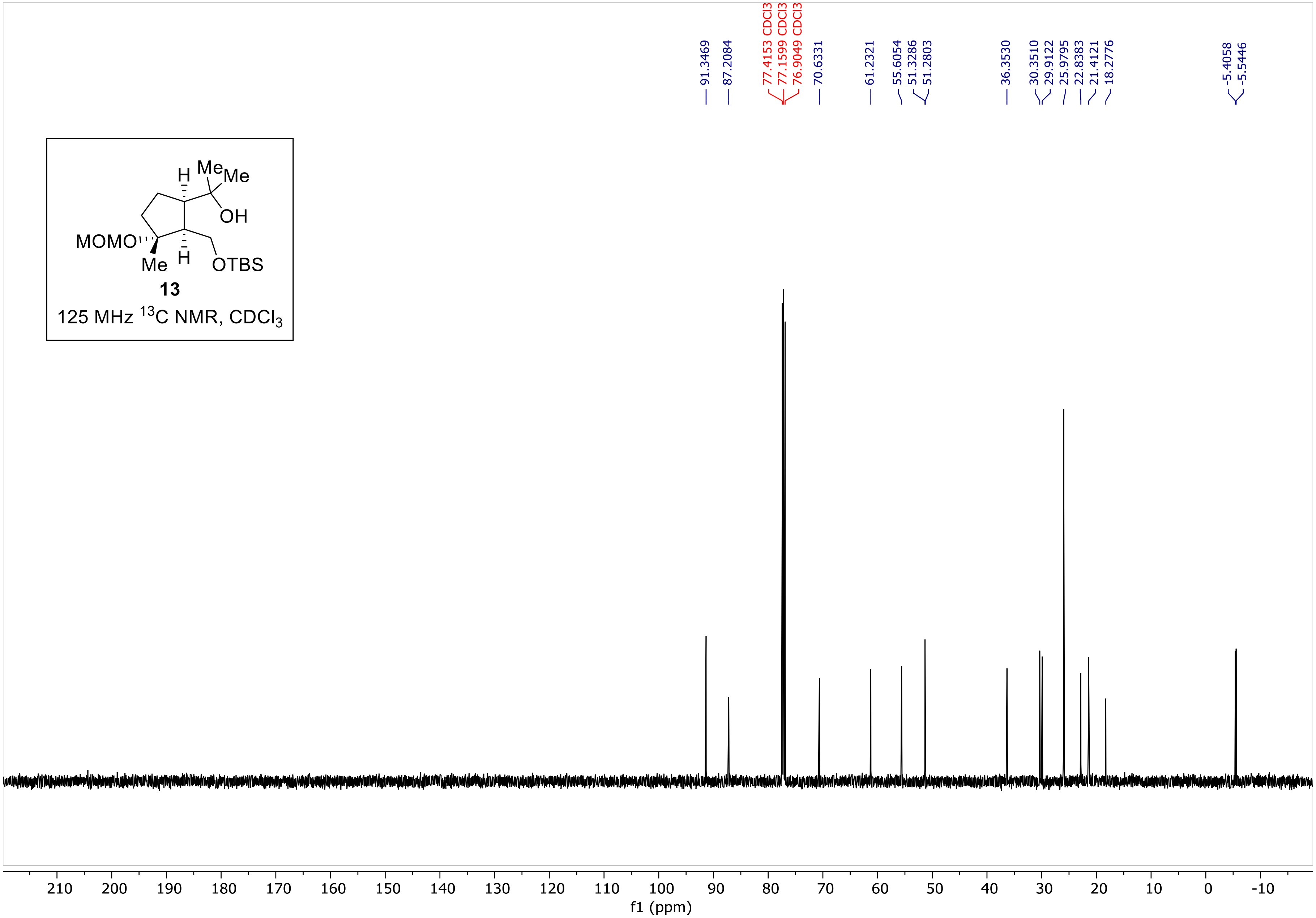

91.3469
87.2084
77.4153 CDCl3
77.1599 CDCl3
76.9049 CDCl3
70.6331
61.2321
55.6054
51.3286
51.2803
36.3530
30.3510
29.9122
25.9795
22.8383
21.4121
18.2776
-5.4058
-5.5446
H
Me
Me
OH
MOMO
Me
H
OTBS
13
125 MHz 13C NMR, CDCl3
f1 (ppm)

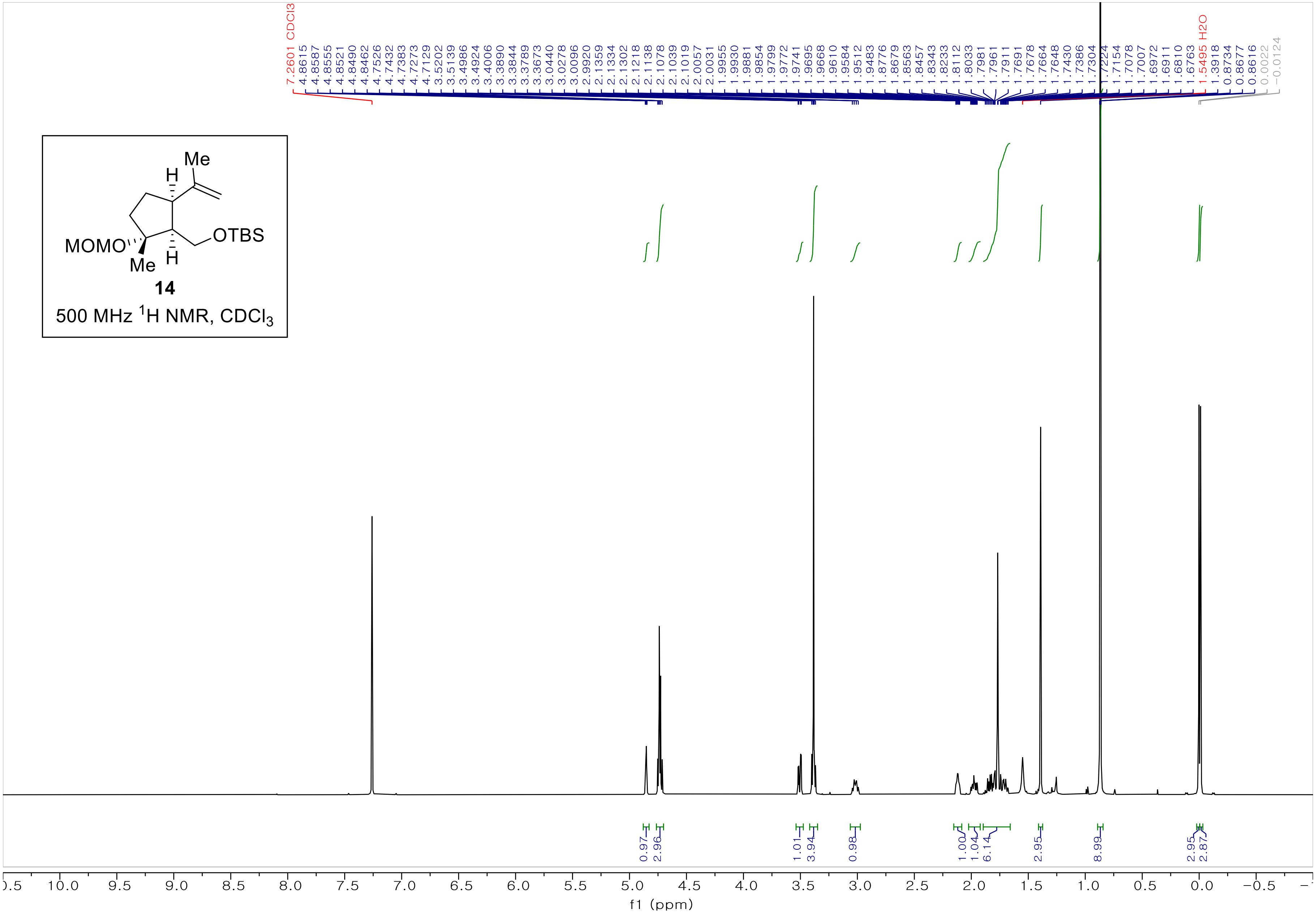
Me
H
MOMO
OTBS
Me
H
14
500 MHz ¹H NMR, CDCl₃
f1 (ppm)

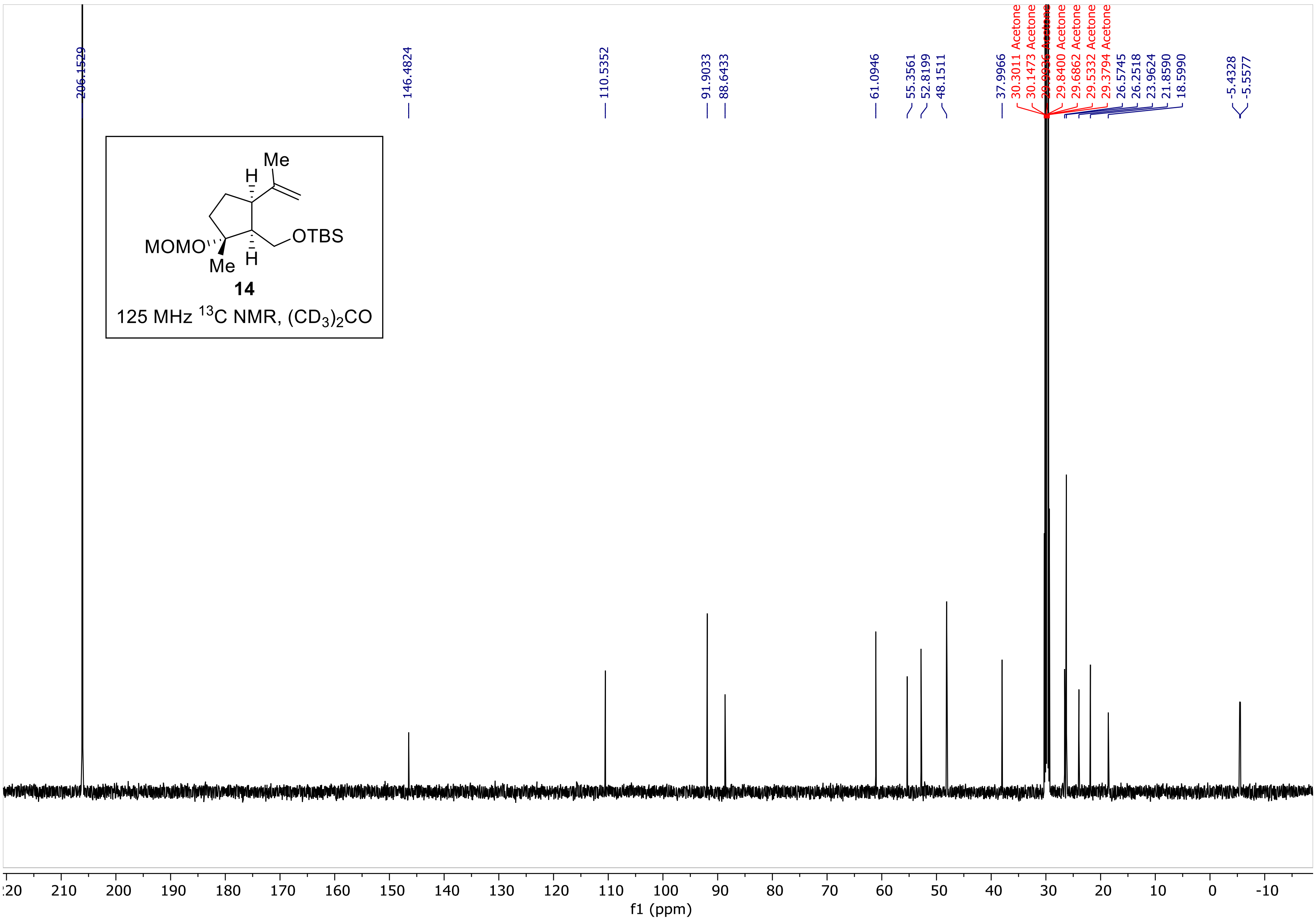

206.1529
146.4824
110.5352
91.9033
88.6433
61.0946
55.3561
52.8199
48.1511
37.9966
30.3011 Acetone
30.1473 Acetone
29.9936 Acetone
29.8400 Acetone
29.6862 Acetone
29.5332 Acetone
29.3794 Acetone
26.5745
26.2518
23.9624
21.8590
18.5990
-5.4328
-5.5577
Me
H
MOMO
OTBS
Me
H
14
125 MHz 13C NMR, (CD3)2CO
f1 (ppm)

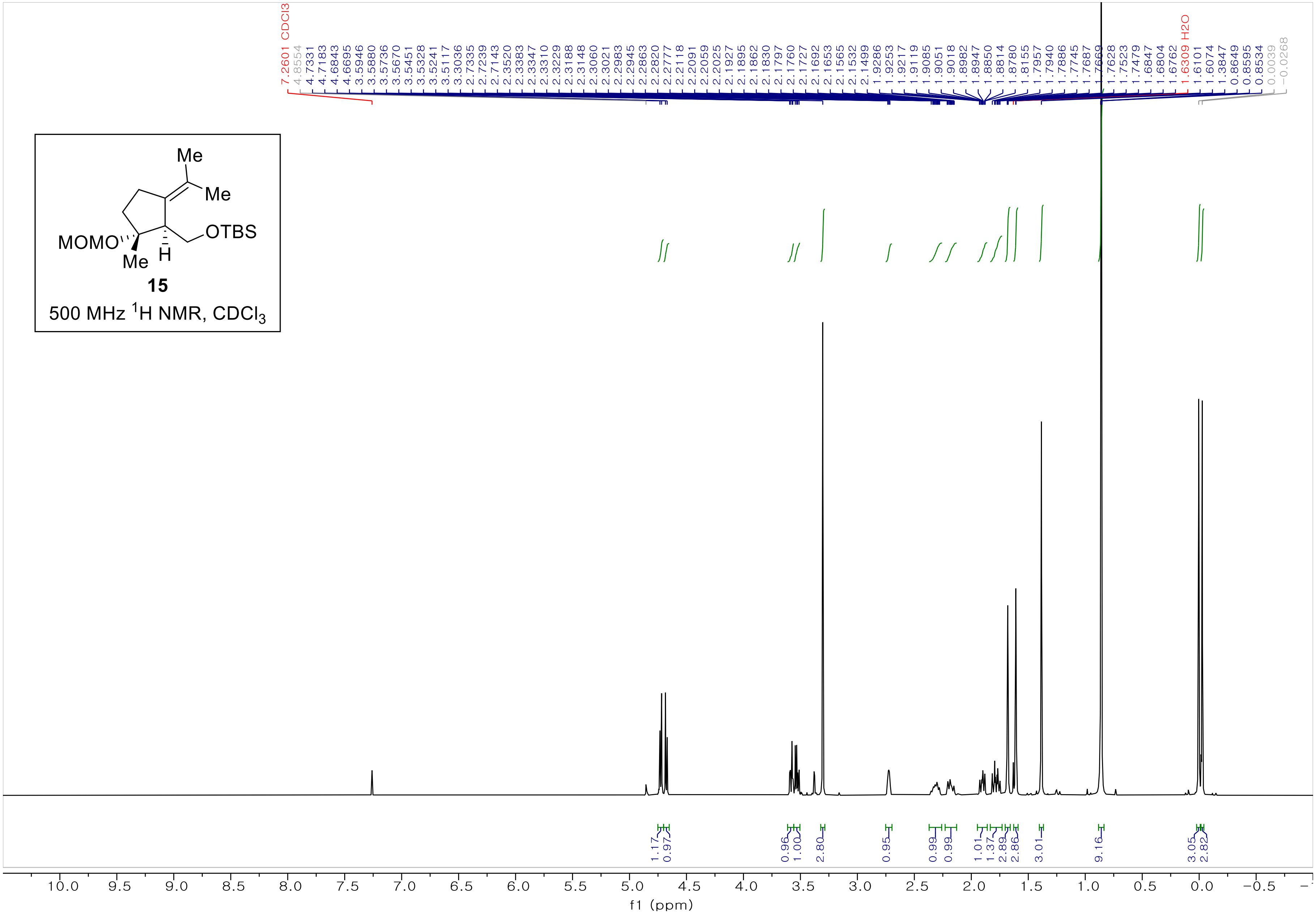
Me
Me
OTBS
MOMO
Me
H
15
500 MHz 1H NMR, CDCl3
7.2601 CDCl3
1.6309 H2O
f1 (ppm)

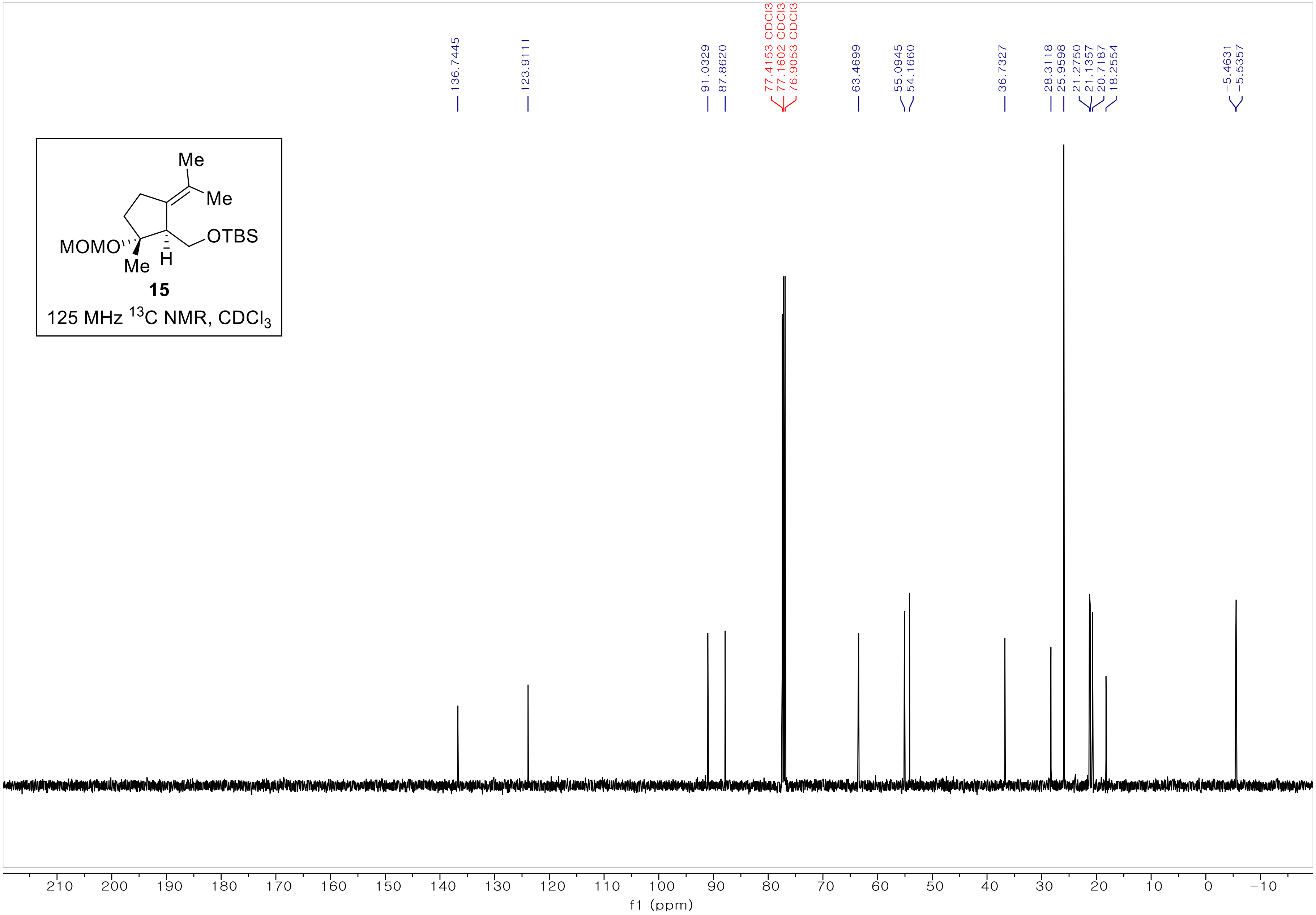
136.7445
123.9111
91.0329
87.8620
77.4153 CDCl3
77.1602 CDCl3
76.9053 CDCl3
63.4699
55.0945
54.1660
36.7327
28.3118
25.9598
21.2750
21.1357
20.7187
18.2554
−5.4631
−5.5357
Me
Me
OTBS
MOMO
Me
H
15
125 MHz ^{13}C NMR, $CDCl_3$
210
200
190
180
170
160
150
140
130
120
110
100
90
80
70
60
50
40
30
20
10
0
−10
f1 (ppm)

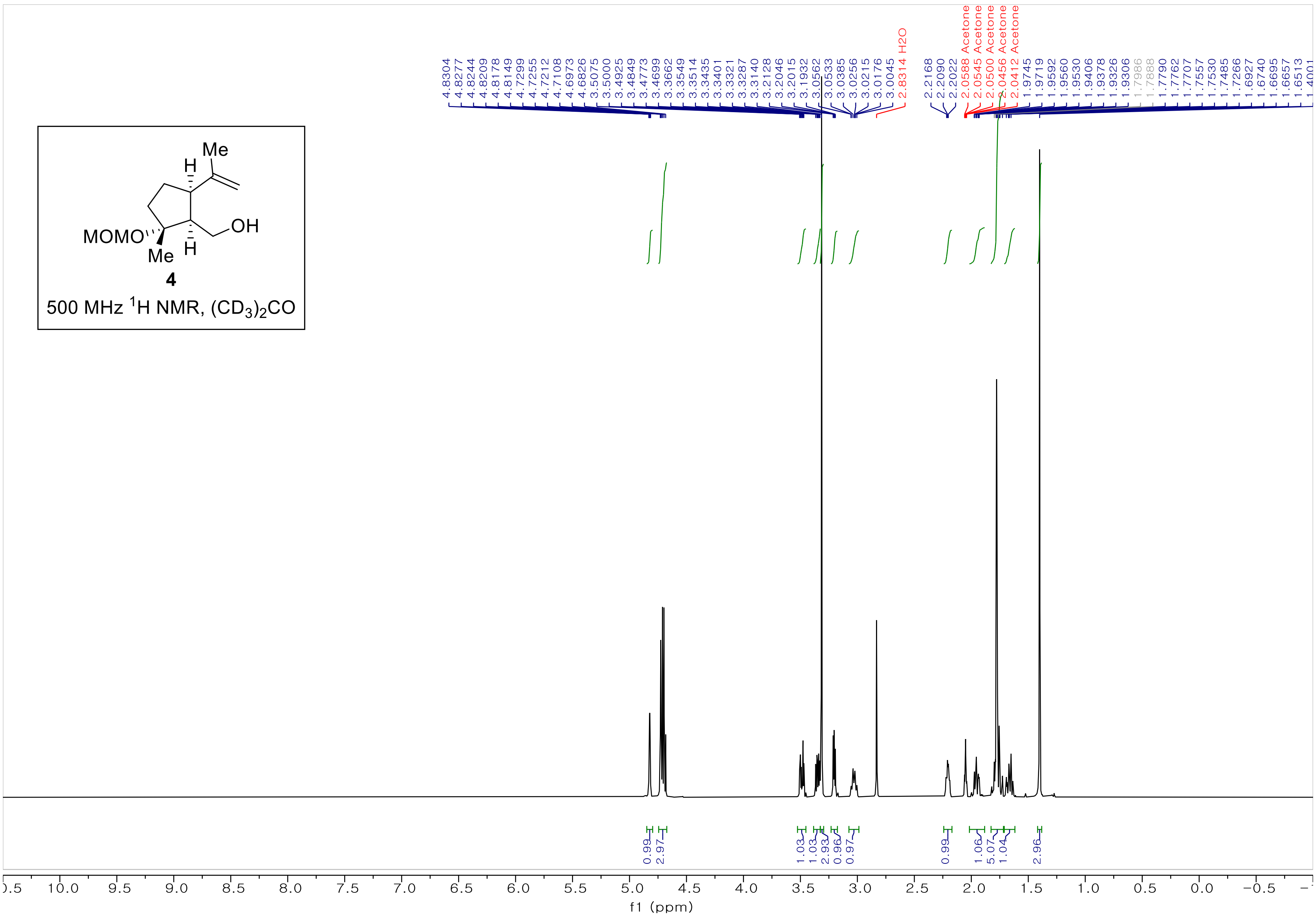
4.8304
4.8277
4.8244
4.8209
4.8178
4.8149
4.7299
4.7255
4.7212
4.7108
4.6973
4.6826
3.5075
3.5000
3.4925
3.4849
3.4773
3.4699
3.3662
3.3549
3.3514
3.3435
3.3401
3.3321
3.3287
3.3140
3.2128
3.2046
3.2015
3.1932
3.0562
3.0533
3.0385
3.0256
3.0215
3.0176
3.0045
2.8314 H2O
2.2168
2.2090
2.2022
2.0588 Acetone
2.0545 Acetone
2.0500 Acetone
2.0456 Acetone
2.0412 Acetone
1.9745
1.9719
1.9592
1.9560
1.9530
1.9406
1.9378
1.9326
1.9306
1.7986
1.7888
1.7790
1.7762
1.7707
1.7557
1.7530
1.7485
1.7266
1.6927
1.6740
1.6695
1.6657
1.6513
1.4001
Me
H
MOMO
OH
Me
H
4
500 MHz 1H NMR, (CD3)2CO
0.99
2.97
1.03
1.03
2.93
0.96
0.97
0.99
1.06
5.07
1.04
2.96
f1 (ppm)

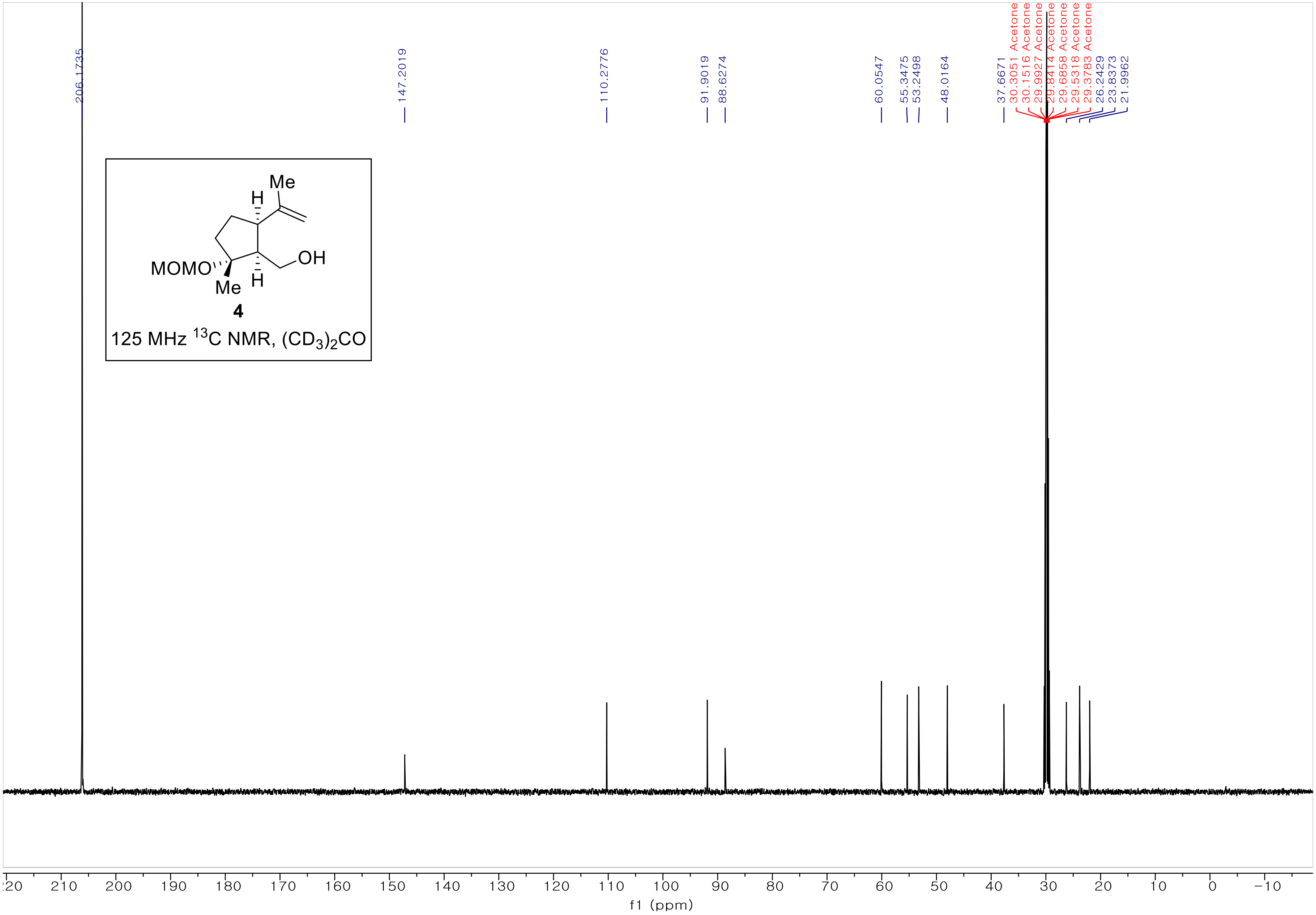

206.1735
147.2019
110.2776
91.9019
88.6274
60.0547
55.3475
53.2498
48.0164
37.6671
30.3051 Acetone
30.1516 Acetone
29.9927 Acetone
29.8414 Acetone
29.6858 Acetone
29.5318 Acetone
29.3783 Acetone
26.2429
23.8373
21.9962
Me
H
MOMO
OH
Me
H
4
125 MHz 13C NMR, (CD3)2CO
f1 (ppm)

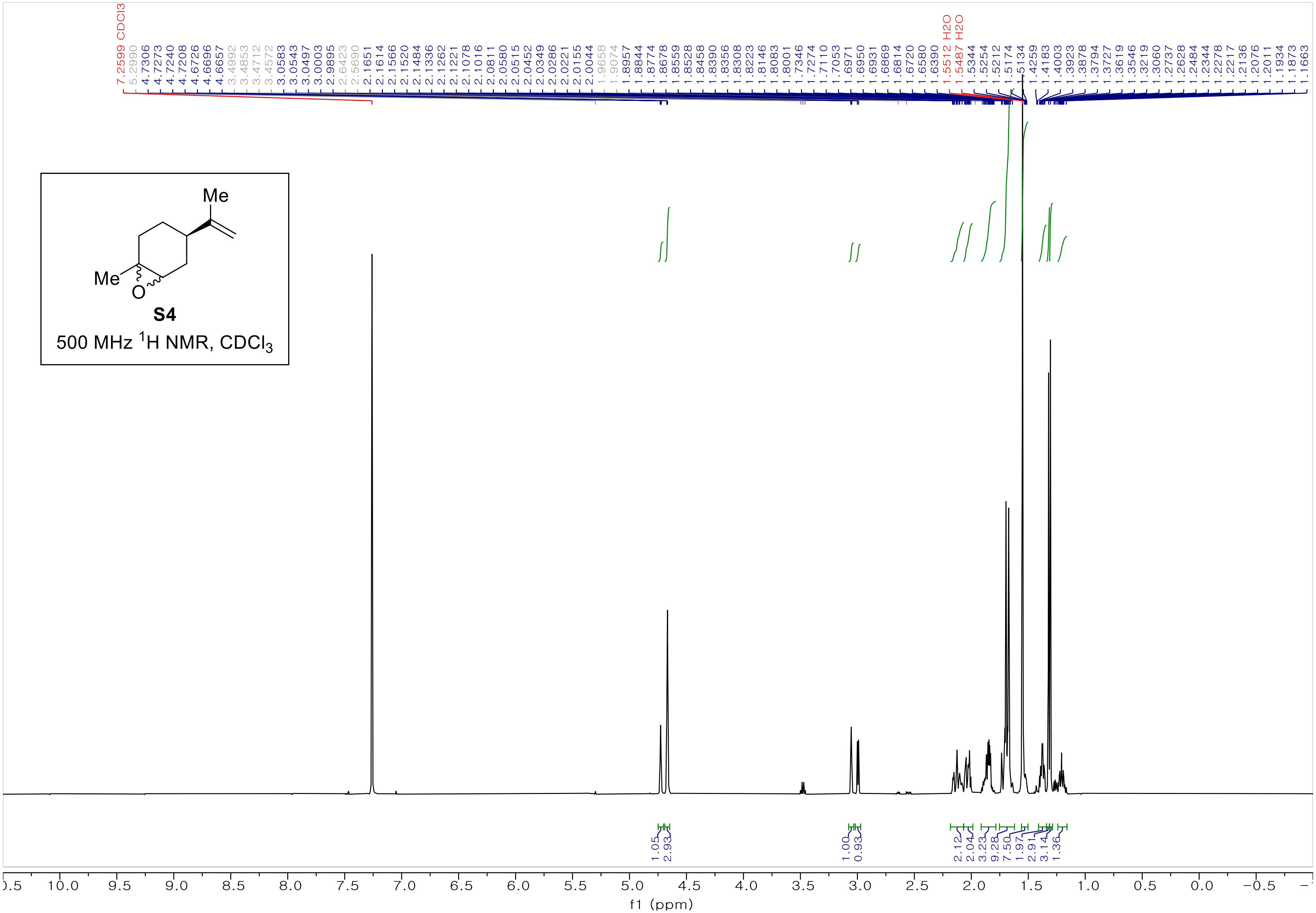

Me
Me
O
S4
500 MHz 1H NMR, CDCl3
f1 (ppm)

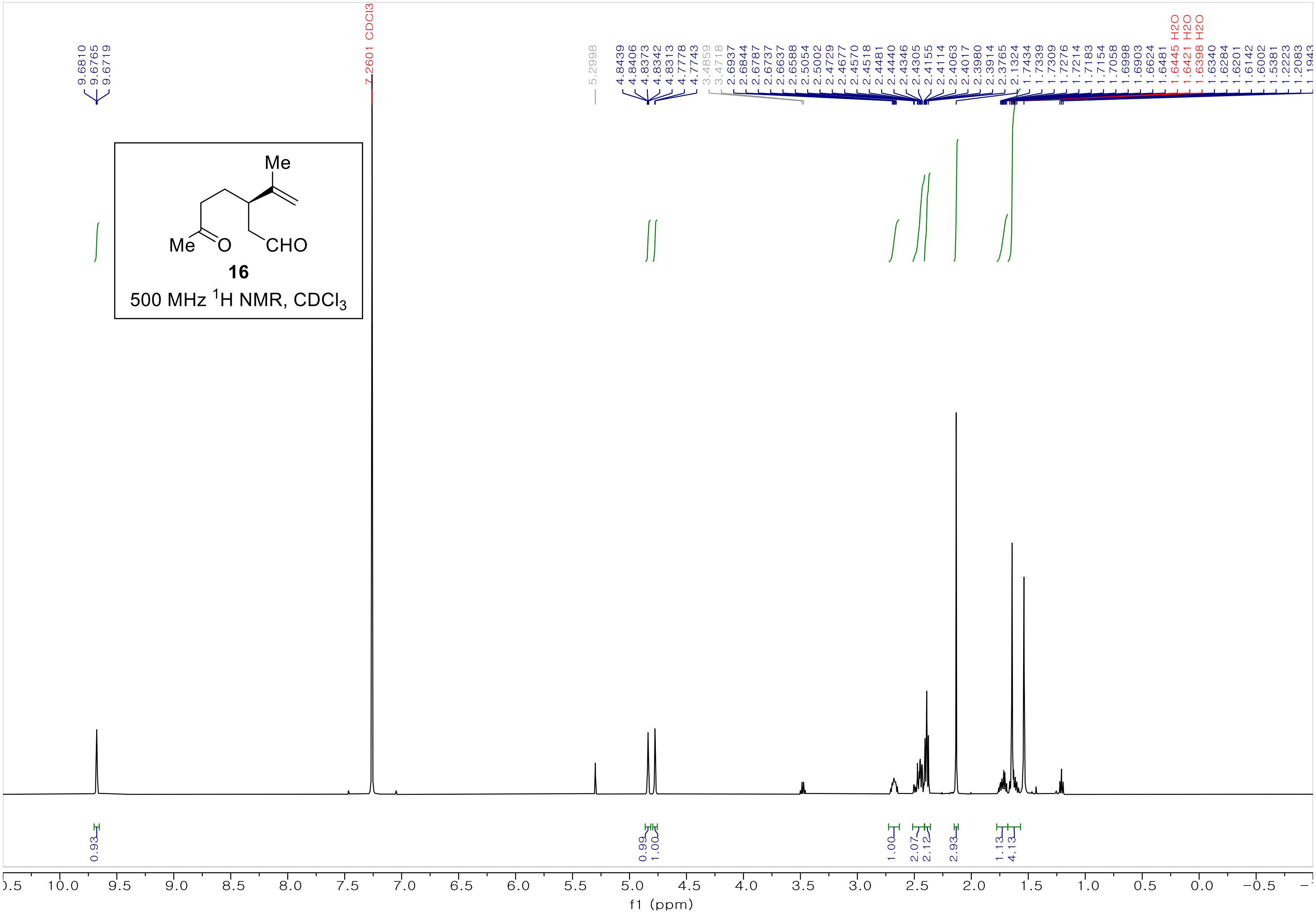

Me
Me
O
CHO
16
500 MHz 1H NMR, CDCl3
9.6810
9.6765
9.6719
7.2601 CDCl3
5.2998
4.8439
4.8406
4.8373
4.8342
4.8313
4.7778
4.7743
3.4859
3.4718
2.6937
2.6844
2.6787
2.6737
2.6637
2.6588
2.5054
2.5002
2.4729
2.4677
2.4570
2.4518
2.4481
2.4440
2.4346
2.4305
2.4155
2.4114
2.4063
2.4017
2.3980
2.3914
2.3765
2.1324
1.7434
1.7339
1.7309
1.7276
1.7214
1.7183
1.7154
1.7058
1.6998
1.6903
1.6624
1.6481
1.6445 H2O
1.6421 H2O
1.6398 H2O
1.6340
1.6284
1.6201
1.6142
1.6002
1.5381
1.2223
1.2083
1.1943
0.93
0.99
1.00
1.00
2.07
2.12
2.93
1.13
4.13
f1 (ppm)

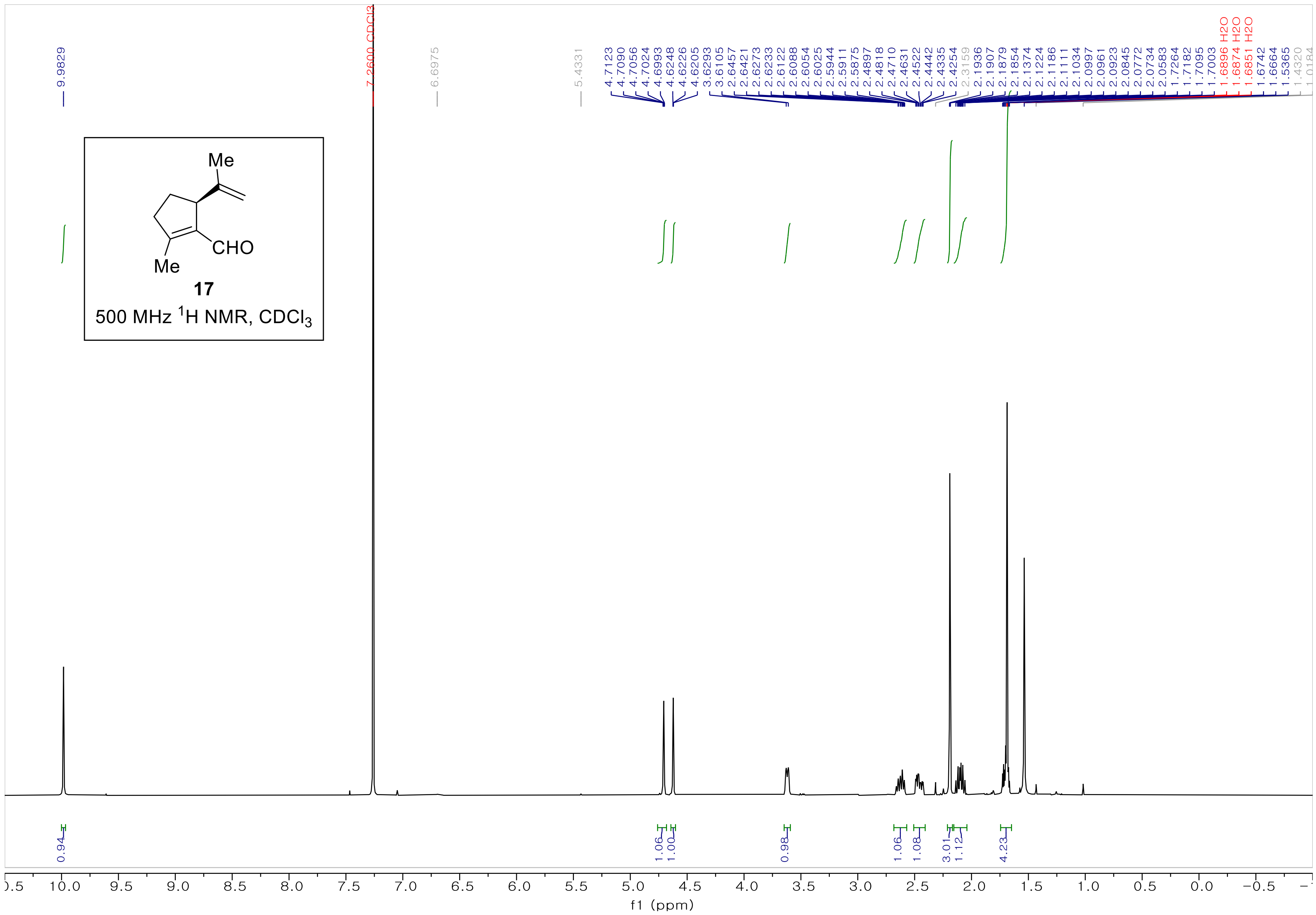

17
500 MHz 1H NMR, CDCl3
f1 (ppm)

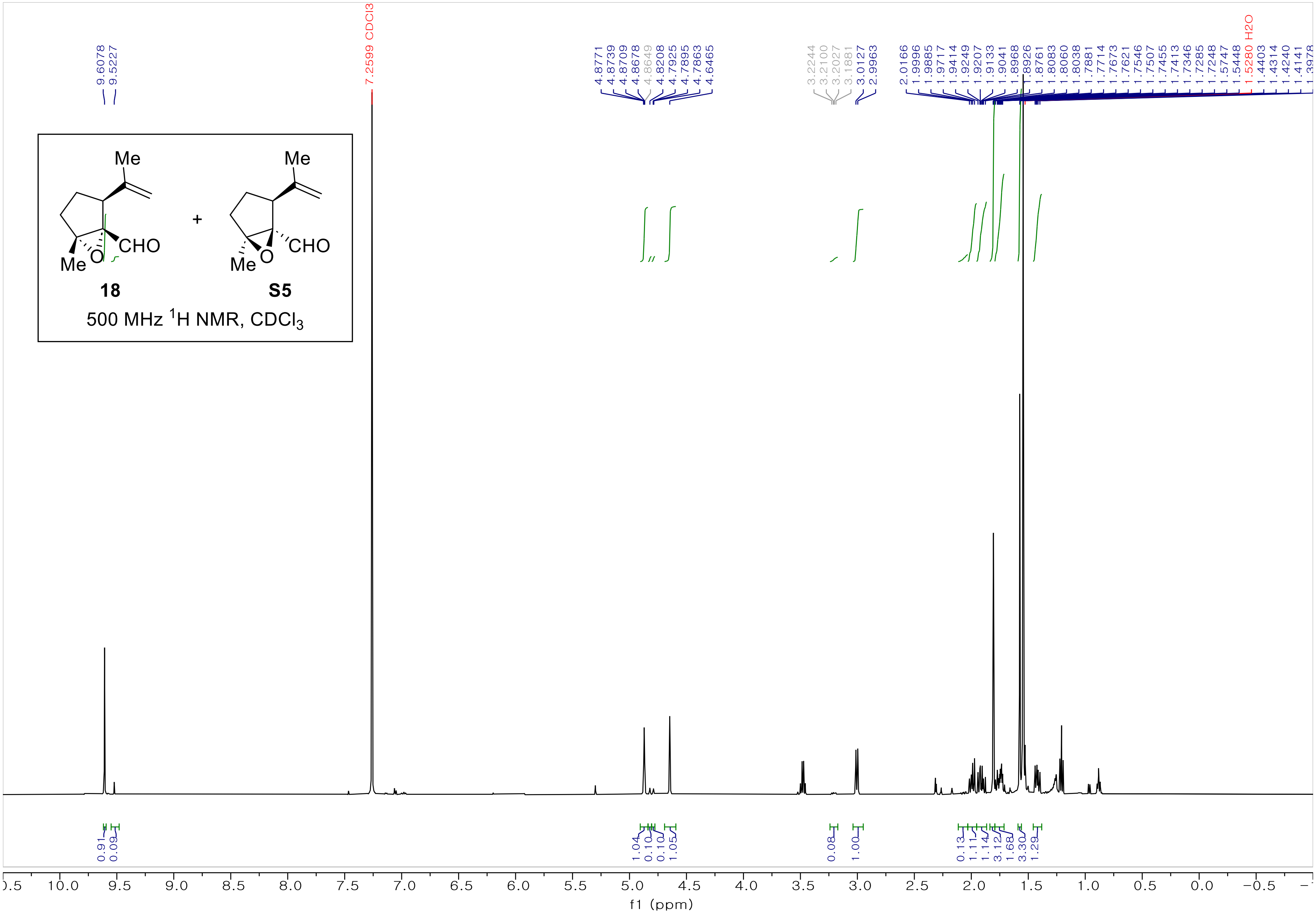
Me
CHO
Me
O
18
S5
500 MHz 1H NMR, CDCl3
7.2599 CDCl3
1.5280 H2O
f1 (ppm)

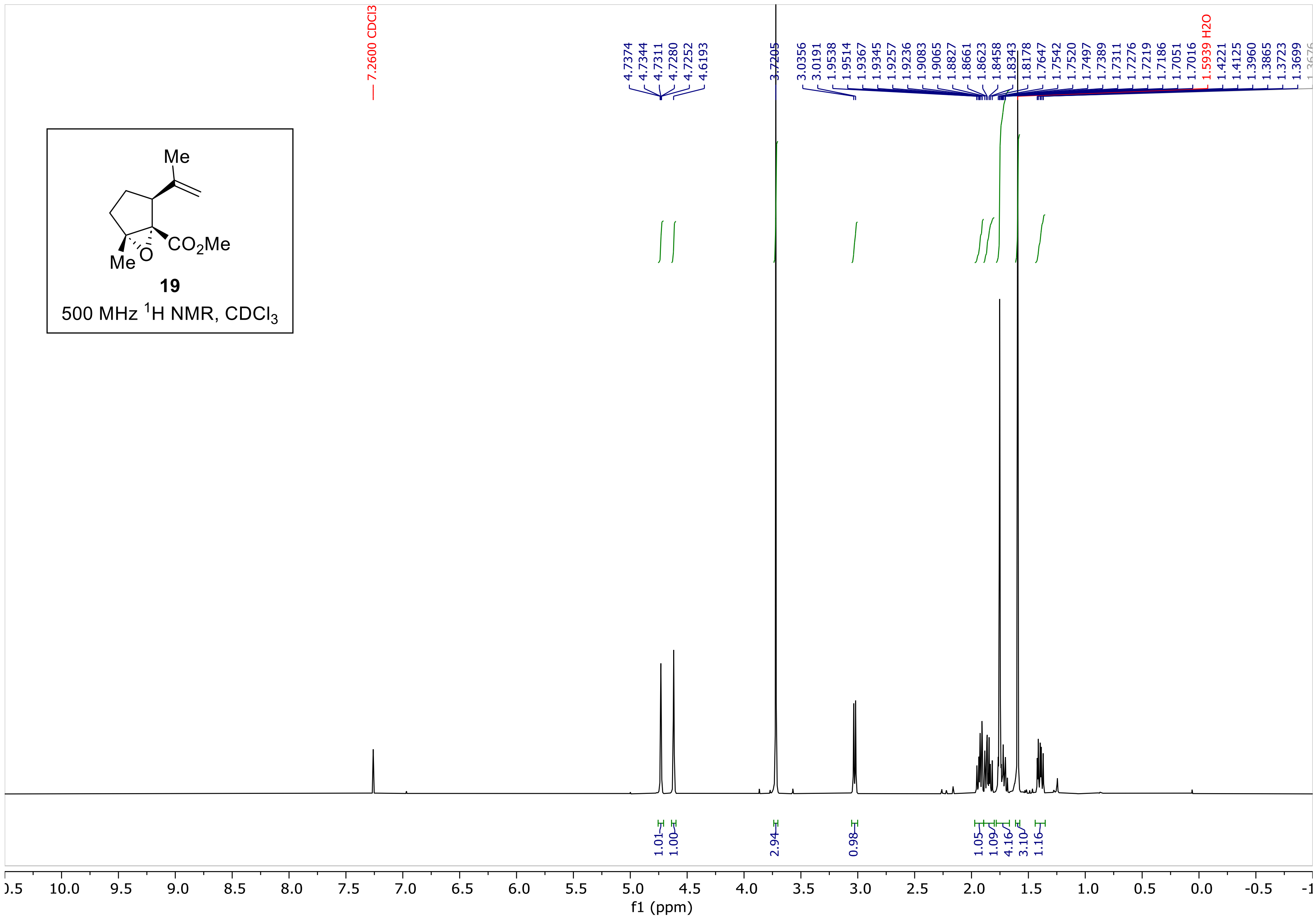

19
500 MHz ¹H NMR, CDCl3
7.2600 CDCl3
1.5939 H2O
f1 (ppm)

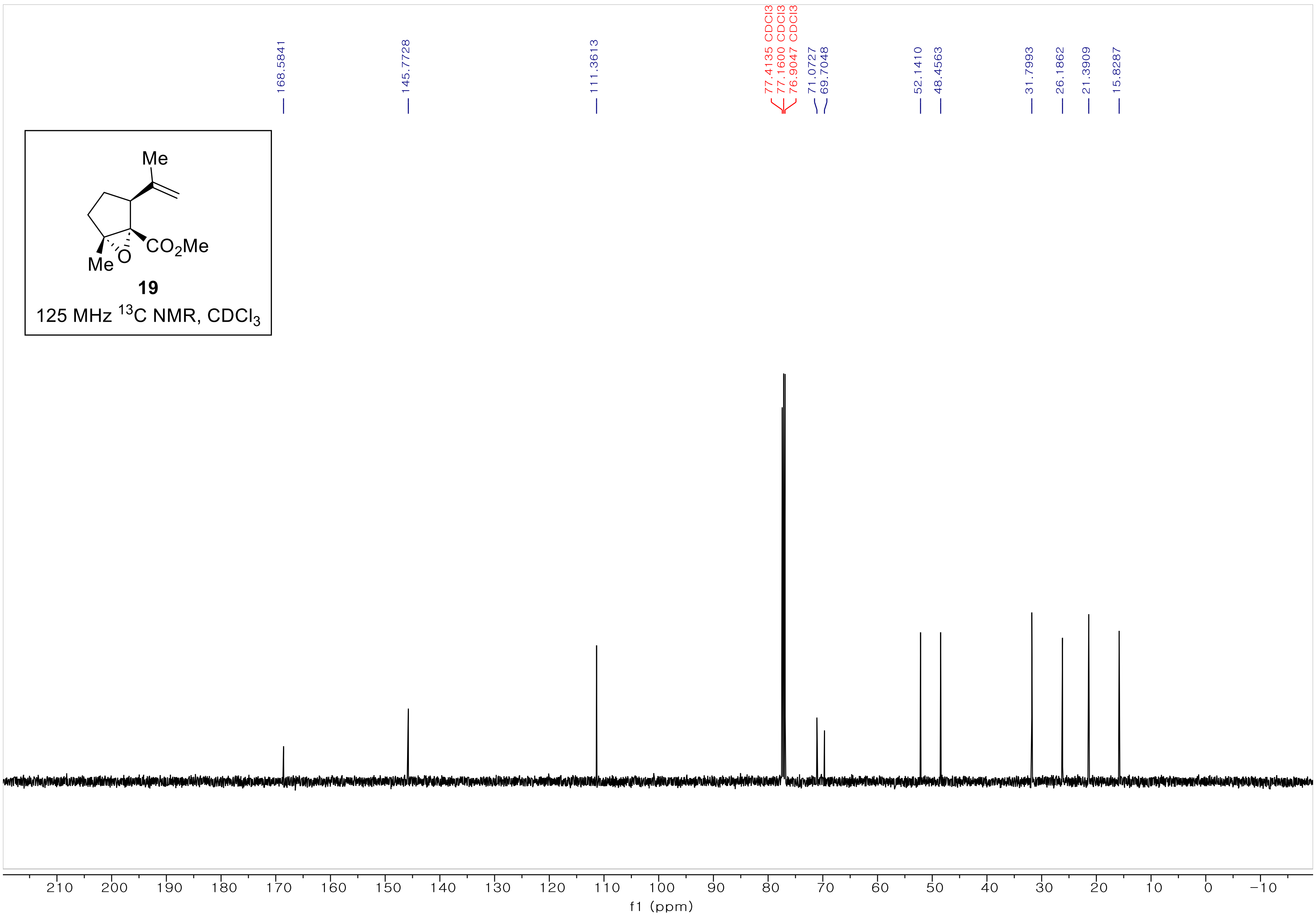
168.5841
145.7728
111.3613
77.4135 CDCl3
77.1600 CDCl3
76.9047 CDCl3
71.0727
69.7048
52.1410
48.4563
31.7993
26.1862
21.3909
15.8287
Me
CO_2Me
Me
O
19
125 MHz ^{13}C NMR, $CDCl_3$
210 200 190 180 170 160 150 140 130 120 110 100 90 80 70 60 50 40 30 20 10 0 −10
f1 (ppm)

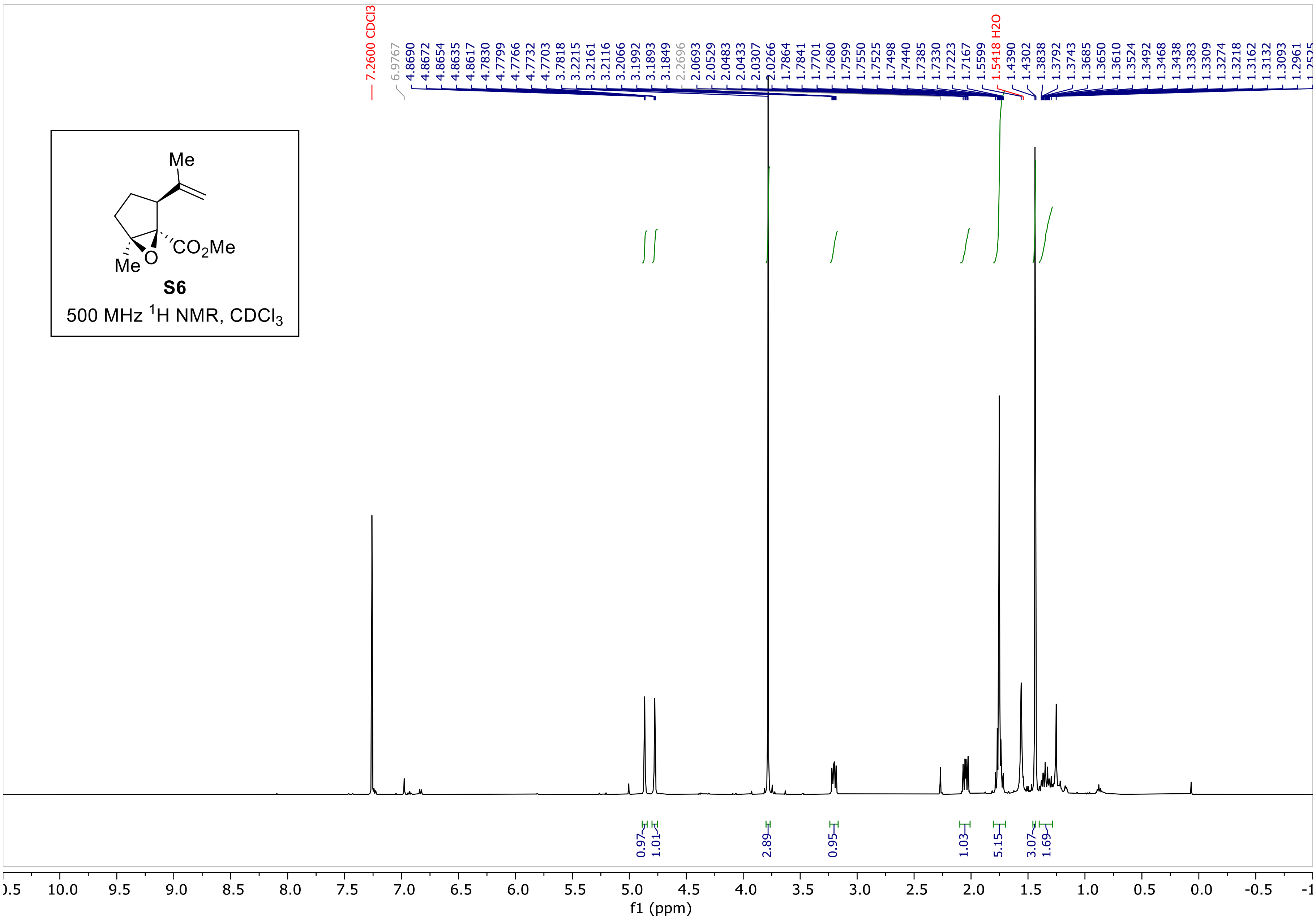
7.2600 CDCl3
6.9767
4.8690
4.8672
4.8654
4.8635
4.8617
4.7830
4.7799
4.7766
4.7732
4.7703
3.7818
3.2215
3.2161
3.2116
3.2066
3.1992
3.1893
3.1849
2.2696
2.0693
2.0529
2.0483
2.0433
2.0307
2.0266
1.7864
1.7841
1.7701
1.7680
1.7599
1.7550
1.7525
1.7498
1.7440
1.7385
1.7330
1.7223
1.7167
1.5599
1.5418 H2O
1.4390
1.4302
1.3838
1.3792
1.3743
1.3685
1.3650
1.3610
1.3524
1.3492
1.3468
1.3438
1.3383
1.3309
1.3274
1.3218
1.3162
1.3132
1.3093
1.2961
Me
CO2Me
Me
O
S6
500 MHz 1H NMR, CDCl3
0.97
1.01
2.89
0.95
1.03
5.15
3.07
1.69
10.0
9.5
9.0
8.5
8.0
7.5
7.0
6.5
6.0
5.5
5.0
4.5
4.0
3.5
3.0
2.5
2.0
1.5
1.0
0.5
0.0
-0.5
f1 (ppm)

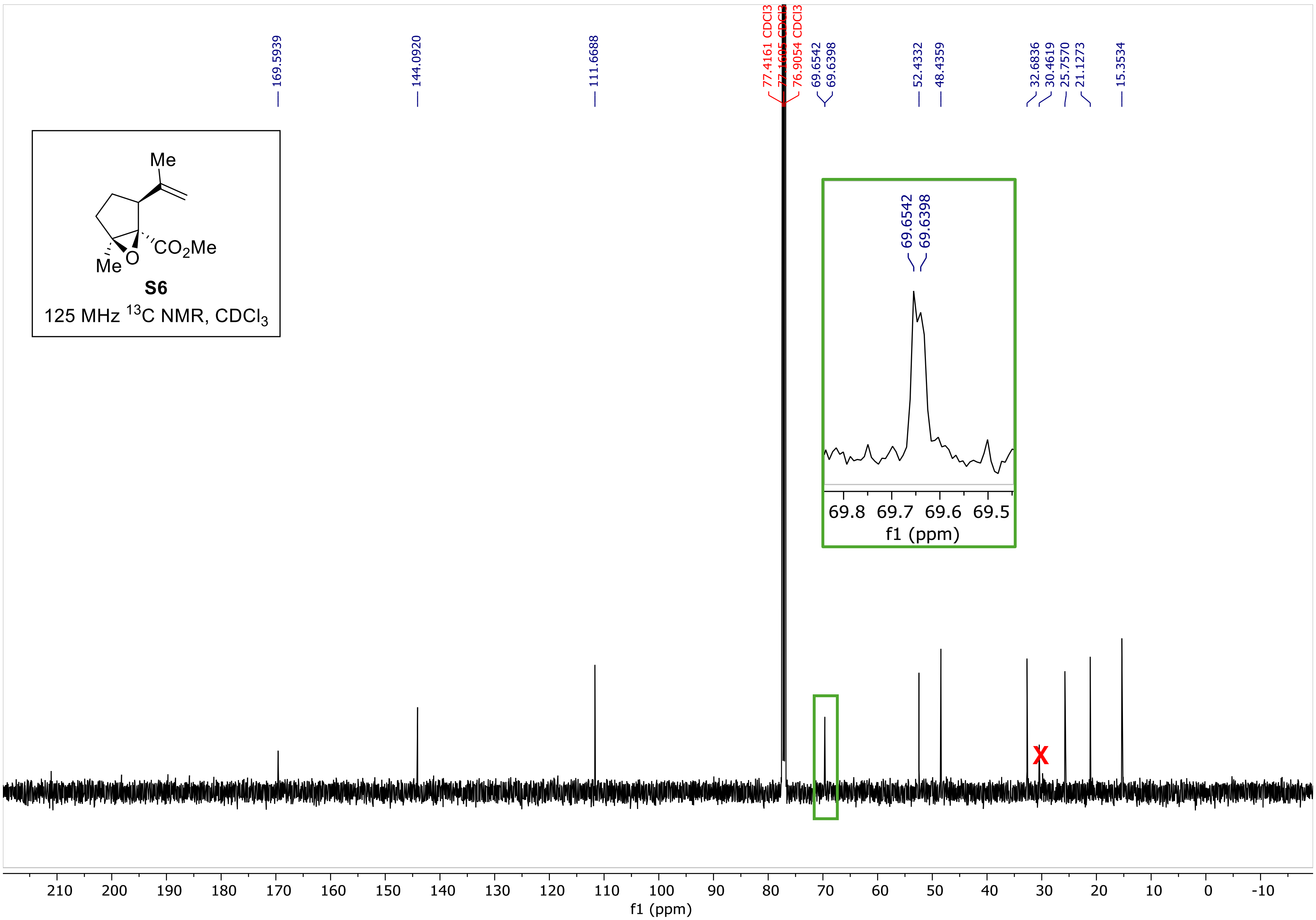
169.5939
144.0920
111.6688
77.4161 CDCl3
76.9054 CDCl3
69.6542
69.6398
52.4332
48.4359
32.6836
30.4619
25.7570
21.1273
15.3534
Me
CO2Me
Me
O
S6
125 MHz 13C NMR, CDCl3
69.6542
69.6398
69.8
69.7
69.6
69.5
f1 (ppm)
X
210
200
190
180
170
160
150
140
130
120
110
100
90
80
70
60
50
40
30
20
10
0
-10
f1 (ppm)

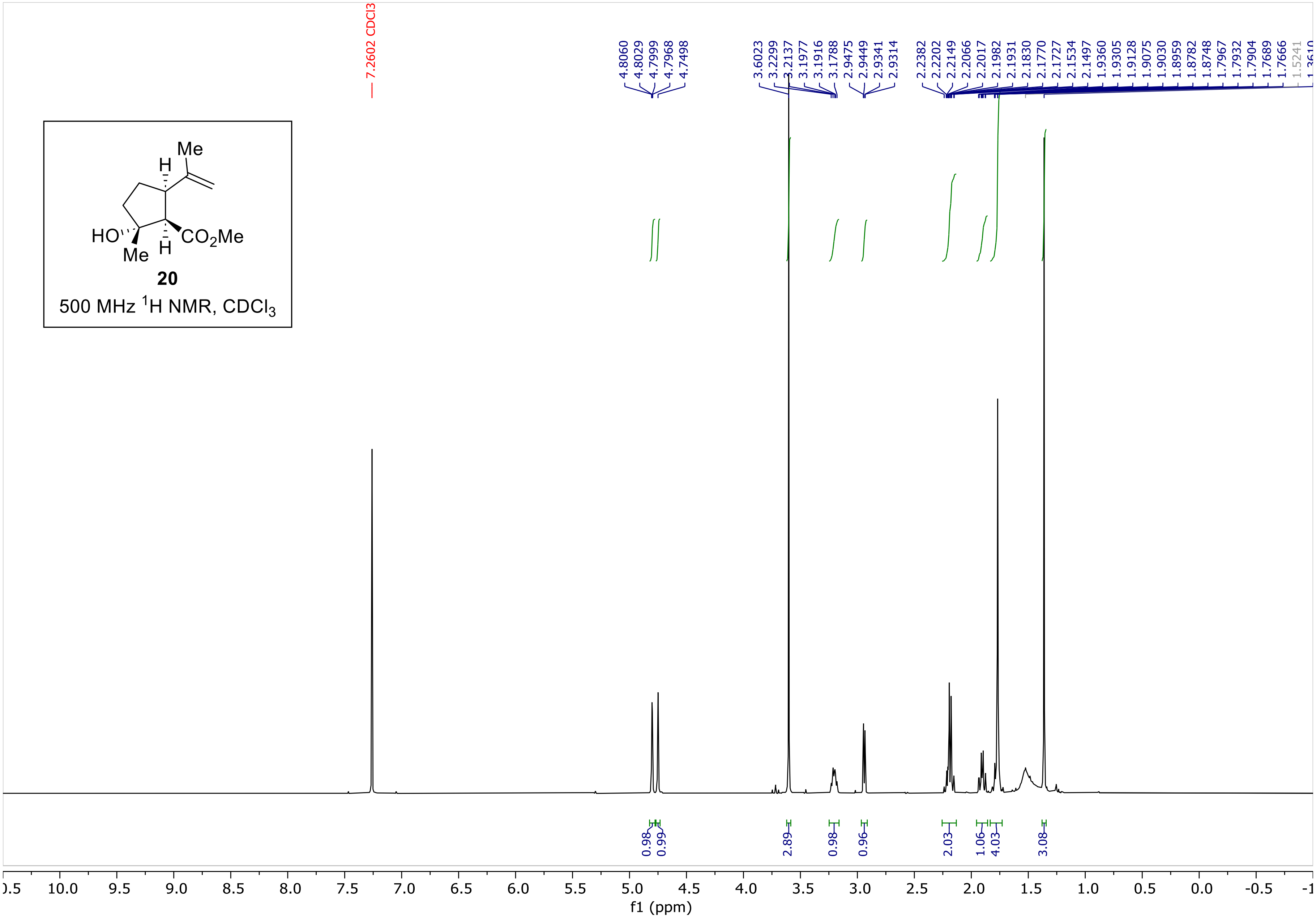
7.2602 CDCl3
4.8060
4.8029
4.7999
4.7968
4.7498
3.6023
3.2299
3.2137
3.1977
3.1916
3.1788
2.9475
2.9449
2.9341
2.9314
2.2382
2.2202
2.2149
2.2066
2.2017
2.1982
2.1931
2.1830
2.1770
2.1727
2.1534
2.1497
1.9360
1.9305
1.9128
1.9075
1.9030
1.8959
1.8782
1.8748
1.7967
1.7932
1.7904
1.7689
1.7666
1.5241
Me
H
HO
CO2Me
Me
H
20
500 MHz 1H NMR, CDCl3
0.98
0.99
2.89
0.98
0.96
2.03
1.06
4.03
3.08
10.0
9.5
9.0
8.5
8.0
7.5
7.0
6.5
6.0
5.5
5.0
4.5
4.0
3.5
3.0
2.5
2.0
1.5
1.0
0.5
0.0
-0.5
f1 (ppm)

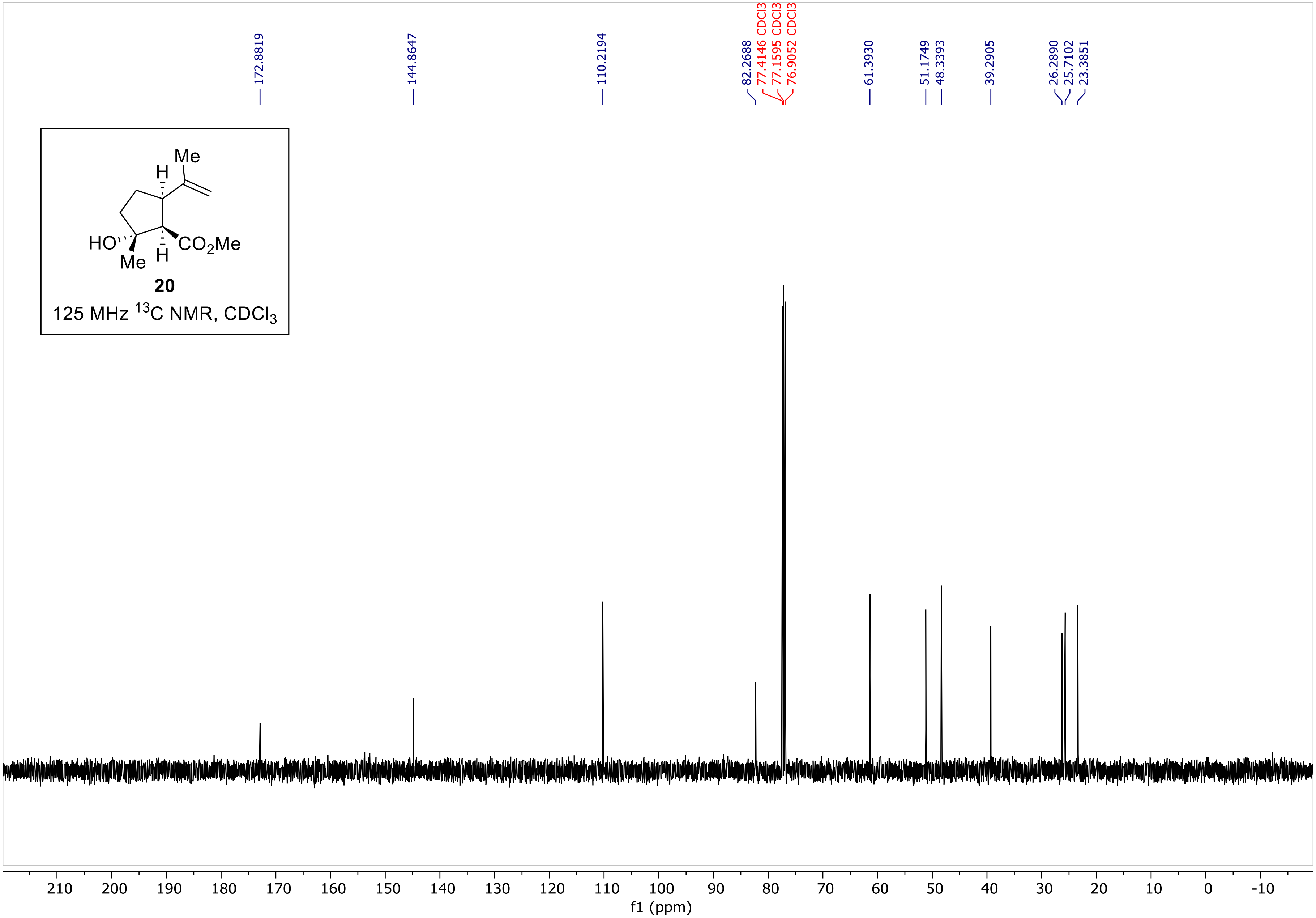
172.8819
144.8647
110.2194
82.2688
77.4146 CDCl3
77.1595 CDCl3
76.9052 CDCl3
61.3930
51.1749
48.3393
39.2905
26.2890
25.7102
23.3851
Me
H
HO
CO2Me
Me
H
20
125 MHz 13C NMR, CDCl3
210 200 190 180 170 160 150 140 130 120 110 100 90 80 70 60 50 40 30 20 10 0 -10
f1 (ppm)

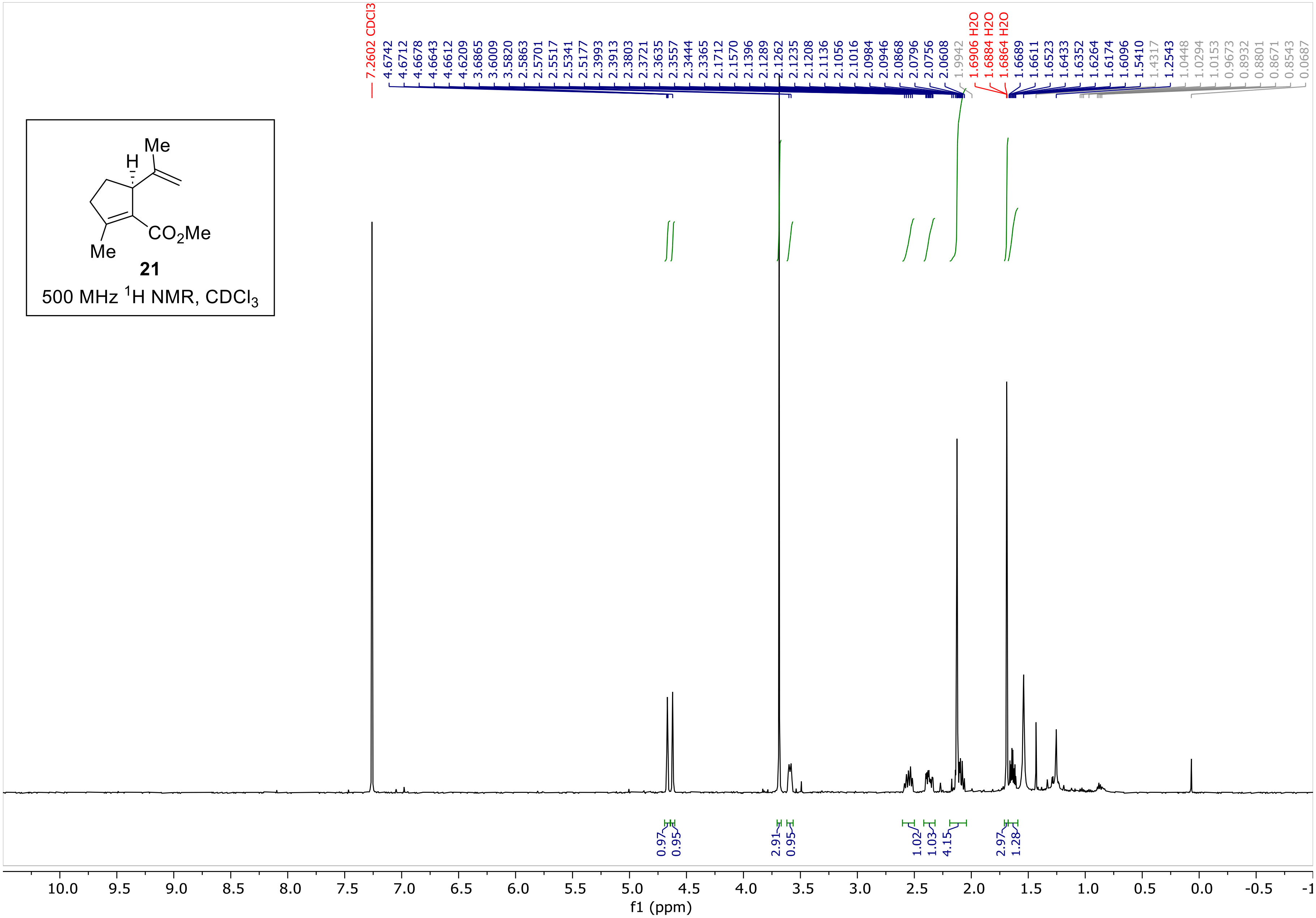

Me
H
CO2Me
Me
21
500 MHz 1H NMR, CDCl3
f1 (ppm)

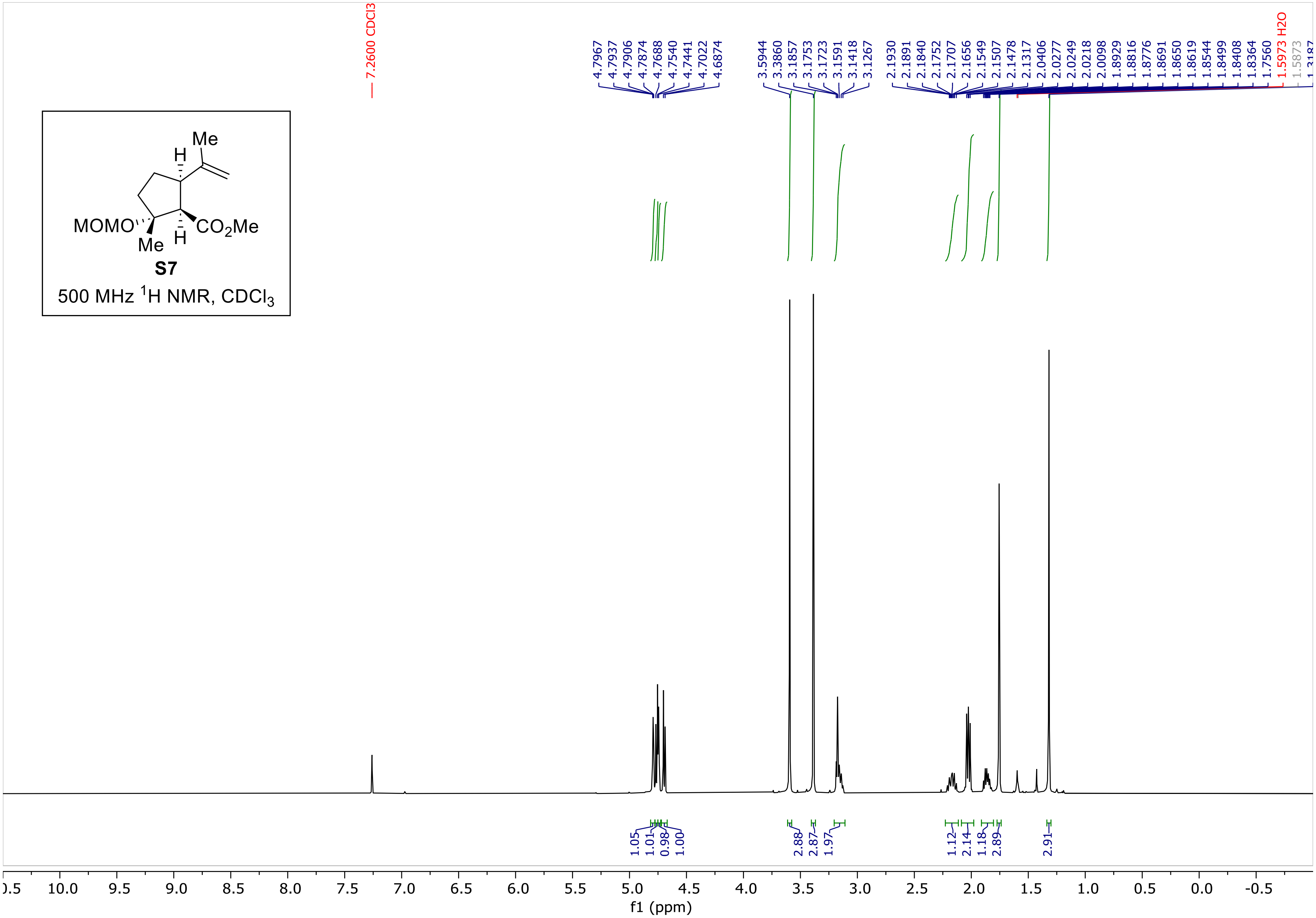

7.2600 CDCl3
4.7967
4.7937
4.7906
4.7874
4.7688
4.7540
4.7441
4.7022
4.6874
3.5944
3.3860
3.1857
3.1753
3.1723
3.1591
3.1418
3.1267
2.1930
2.1891
2.1840
2.1752
2.1707
2.1656
2.1549
2.1507
2.1478
2.1317
2.0406
2.0277
2.0249
2.0218
2.0098
1.8929
1.8816
1.8776
1.8691
1.8650
1.8619
1.8544
1.8499
1.8408
1.8364
1.7560
1.5973 H2O
1.5873
Me
H
MOMO
CO2Me
Me
H
S7
500 MHz 1H NMR, CDCl3
1.05
1.01
0.98
1.00
2.88
2.87
1.97
1.12
2.14
1.18
2.89
2.91
10.0
9.5
9.0
8.5
8.0
7.5
7.0
6.5
6.0
5.5
5.0
4.5
4.0
3.5
3.0
2.5
2.0
1.5
1.0
0.5
0.0
-0.5
f1 (ppm)

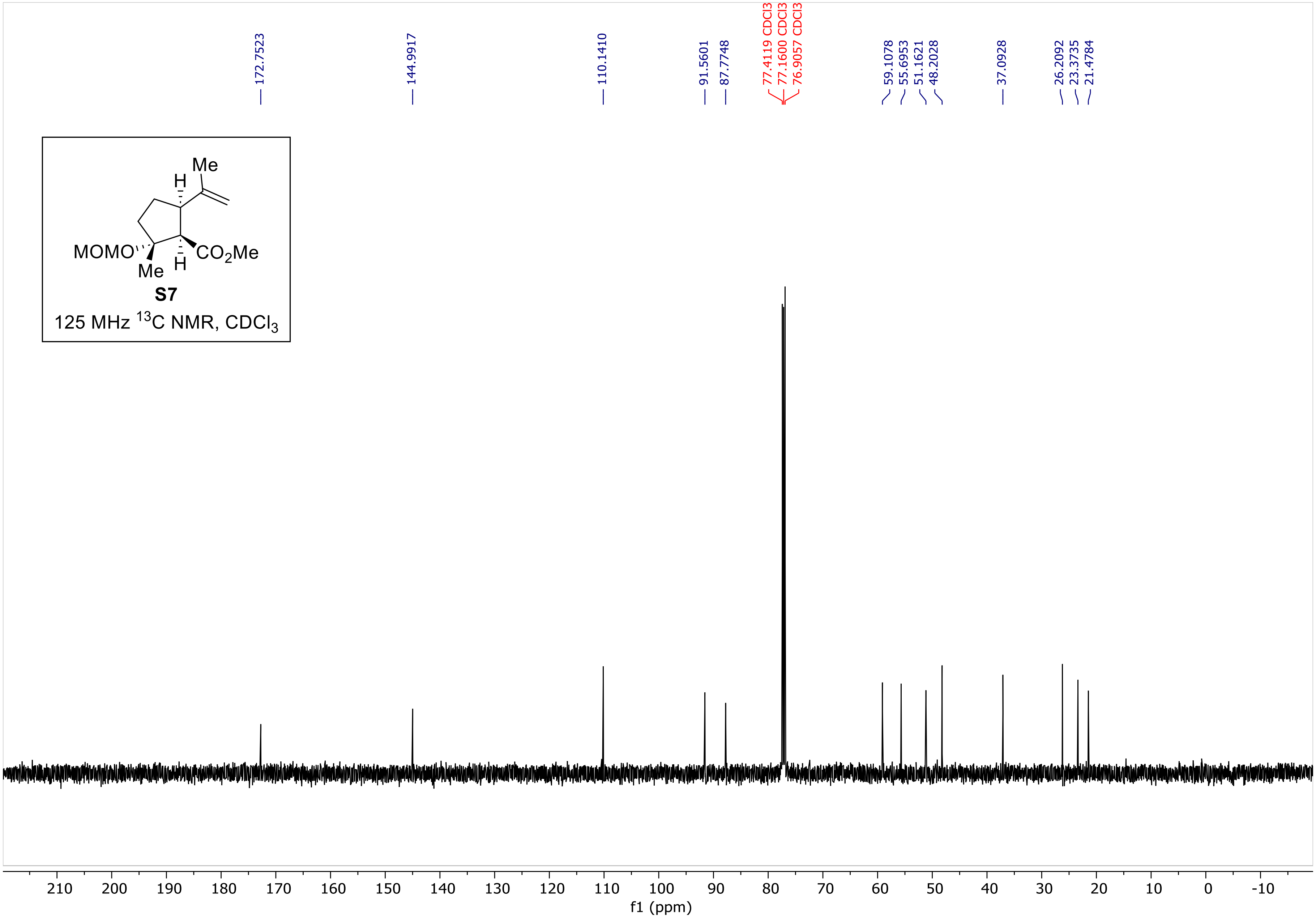
172.7523
144.9917
110.1410
91.5601
87.7748
77.4119 CDCl3
77.1600 CDCl3
76.9057 CDCl3
59.1078
55.6953
51.1621
48.2028
37.0928
26.2092
23.3735
21.4784
Me
H
MOMO
Me
H
CO2Me
S7
125 MHz 13C NMR, CDCl3
210 200 190 180 170 160 150 140 130 120 110 100 90 80 70 60 50 40 30 20 10 0 -10
f1 (ppm)

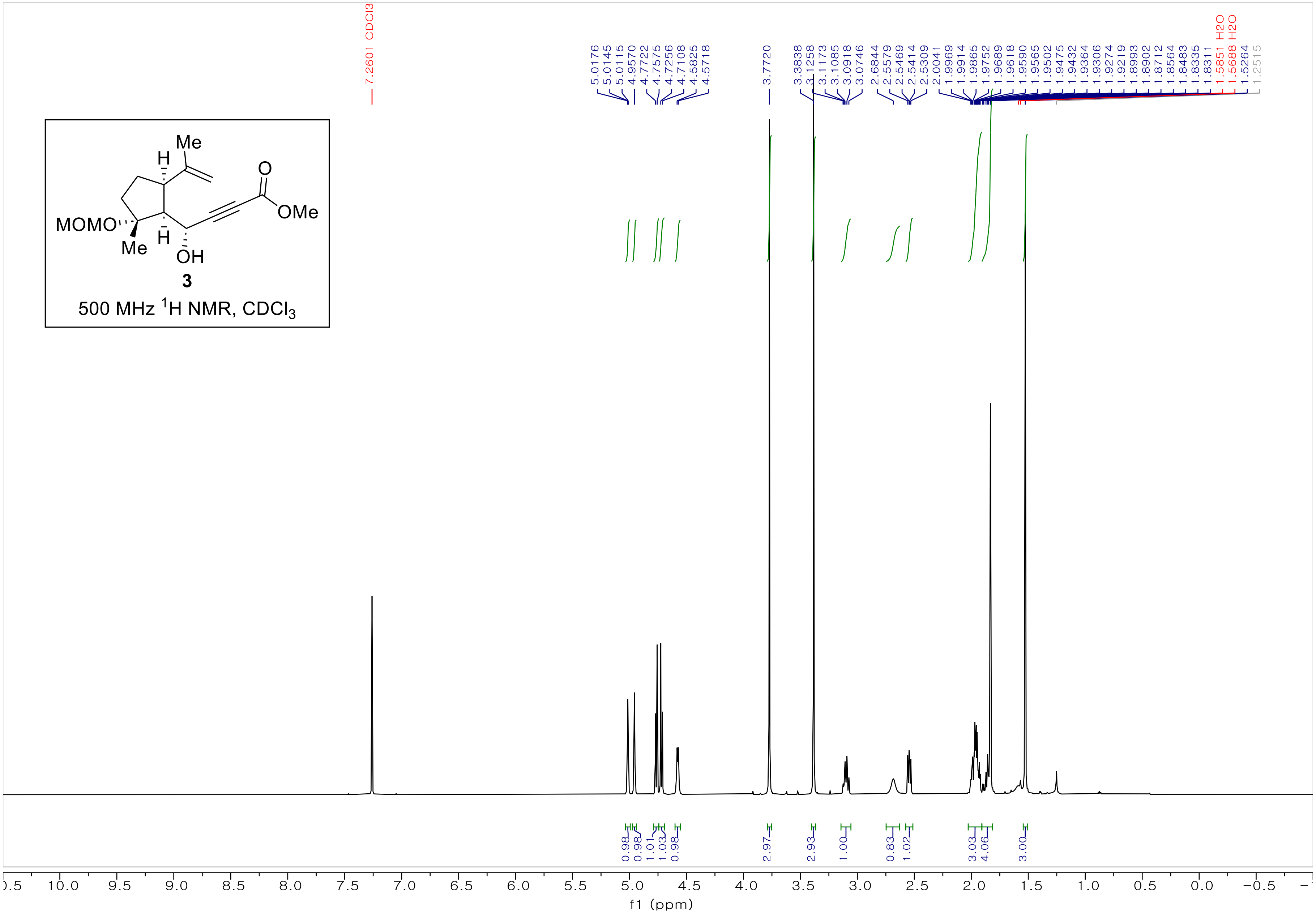
Me
H
O
MOMO
OMe
Me
H
OH
3
500 MHz ¹H NMR, CDCl₃
7.2601 CDCl3
f1 (ppm)

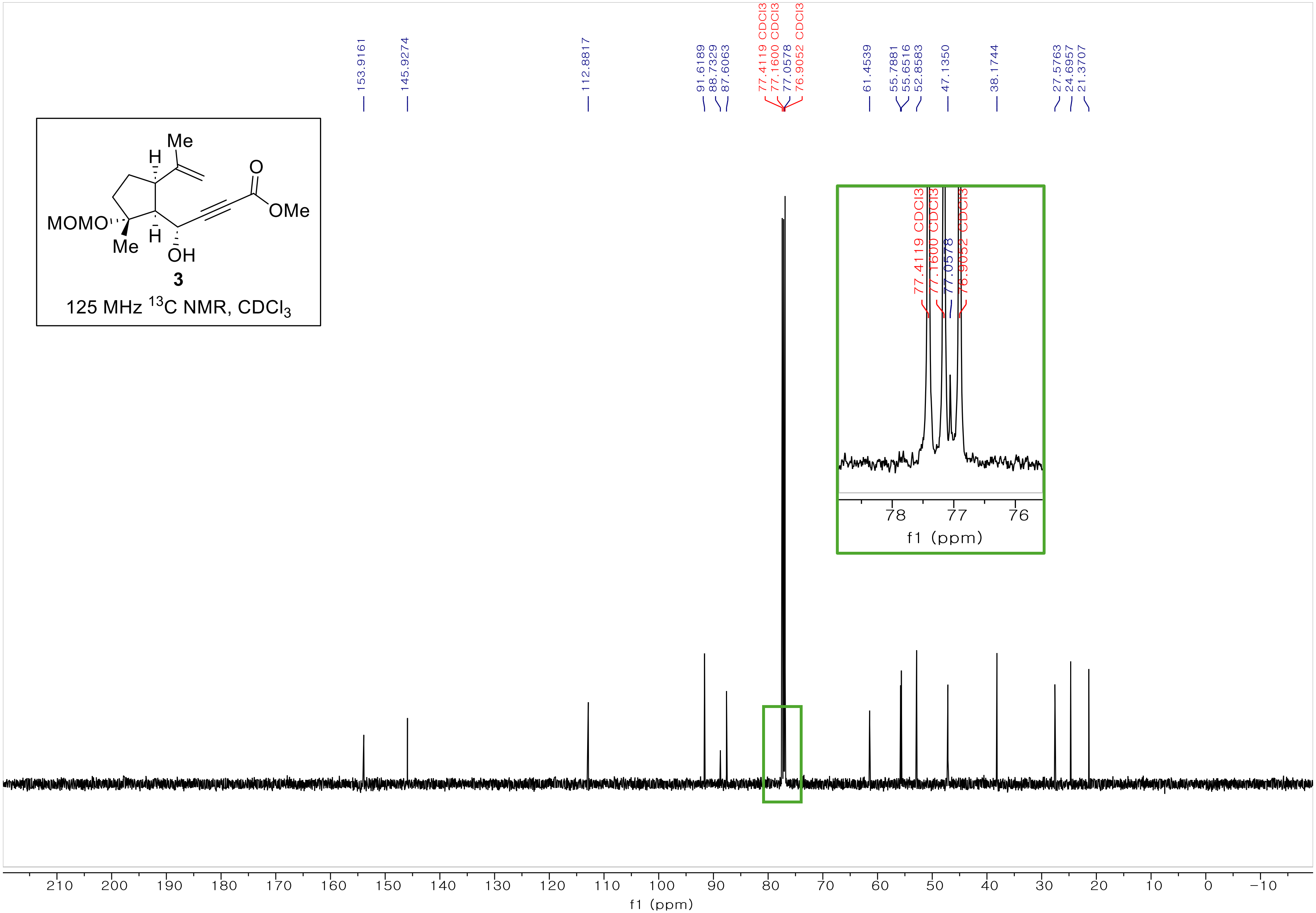
153.9161
145.9274
112.8817
91.6189
88.7329
87.6063
77.4119 CDCl3
77.1600 CDCl3
77.0578
76.9052 CDCl3
61.4539
55.7881
55.6516
52.8583
47.1350
38.1744
27.5763
24.6957
21.3707
Me
H
O
OMe
MOMO
Me
H
OH
3
125 MHz 13C NMR, CDCl3
78
77
76
f1 (ppm)
210
200
190
180
170
160
150
140
130
120
110
100
90
80
70
60
50
40
30
20
10
0
-10
f1 (ppm)

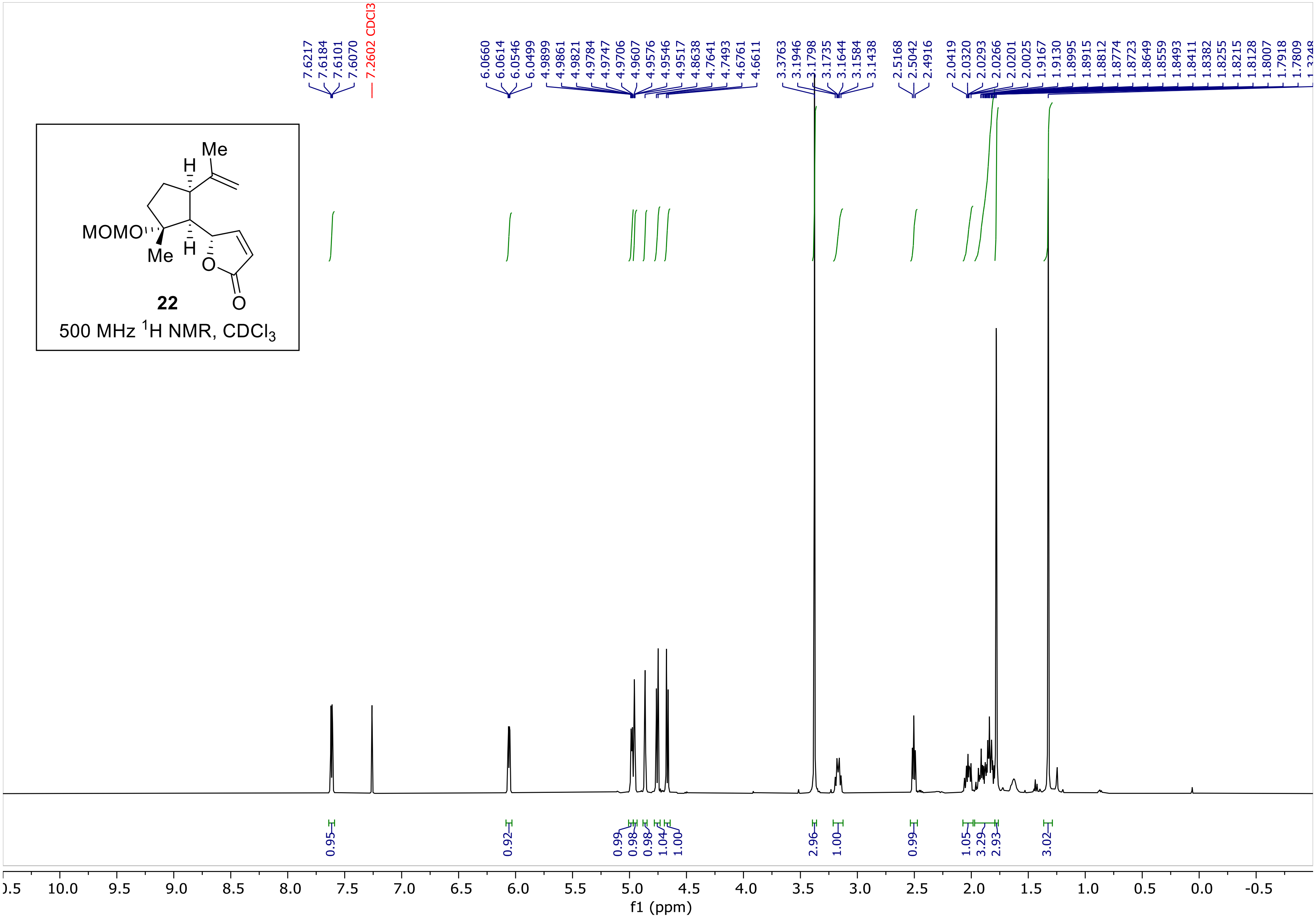
MOMO
Me
H
Me
H
O
O
22
500 MHz ¹H NMR, CDCl₃
7.6217
7.6184
7.6101
7.6070
7.2602 CDCl3
6.0660
6.0614
6.0546
6.0499
3.3763
2.5168
2.5042
2.4916
0.95
0.92
0.99
0.98
0.98
1.04
1.00
2.96
1.00
0.99
1.05
3.29
2.93
3.02
f1 (ppm)

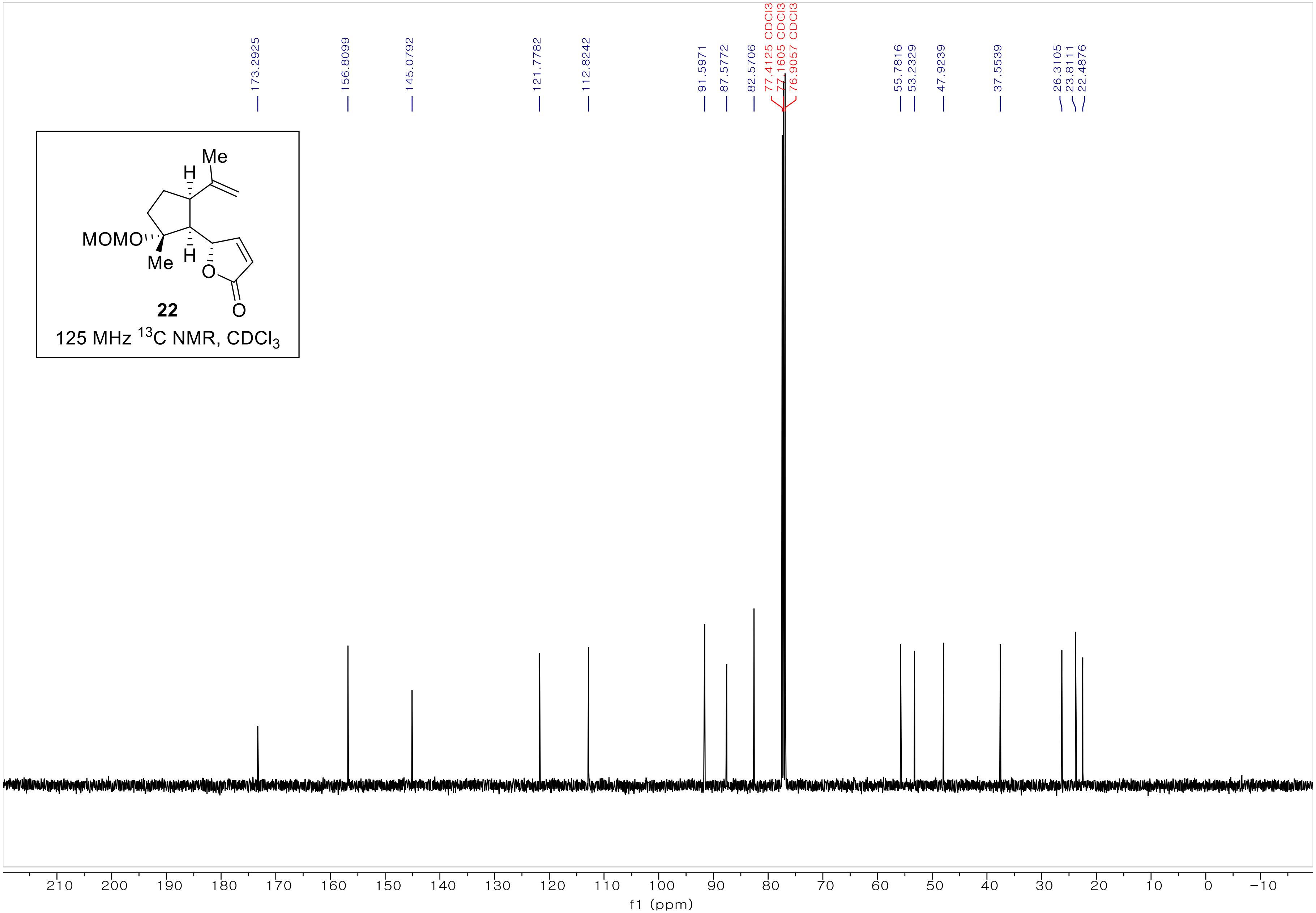
173.2925
156.8099
145.0792
121.7782
112.8242
91.5971
87.5772
82.5706
77.4125 CDCl3
77.1605 CDCl3
76.9057 CDCl3
55.7816
53.2329
47.9239
37.5539
26.3105
23.8111
22.4876
Me
H
MOMO
Me
H
O
O
22
125 MHz 13C NMR, CDCl3
210 200 190 180 170 160 150 140 130 120 110 100 90 80 70 60 50 40 30 20 10 0 −10
f1 (ppm)

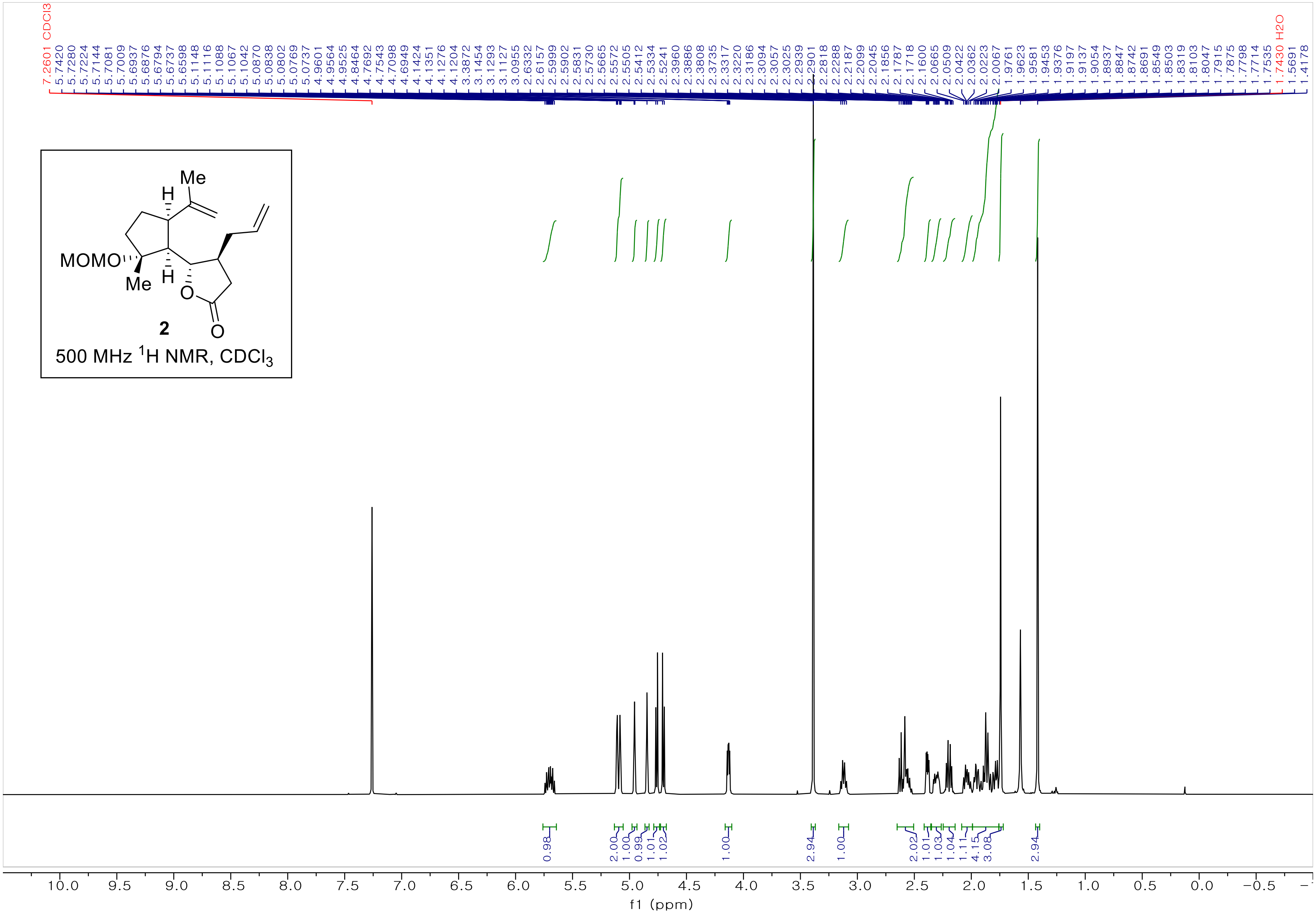

MOMO
Me
H
O
2
500 MHz ^{1}H NMR, $CDCl_3$
7.2601 CDCl3
1.7430 H2O
0.98
2.00
1.00
0.99
1.01
1.02
1.00
2.94
1.00
2.02
1.01
1.03
1.04
1.11
4.15
3.08
2.94
f1 (ppm)

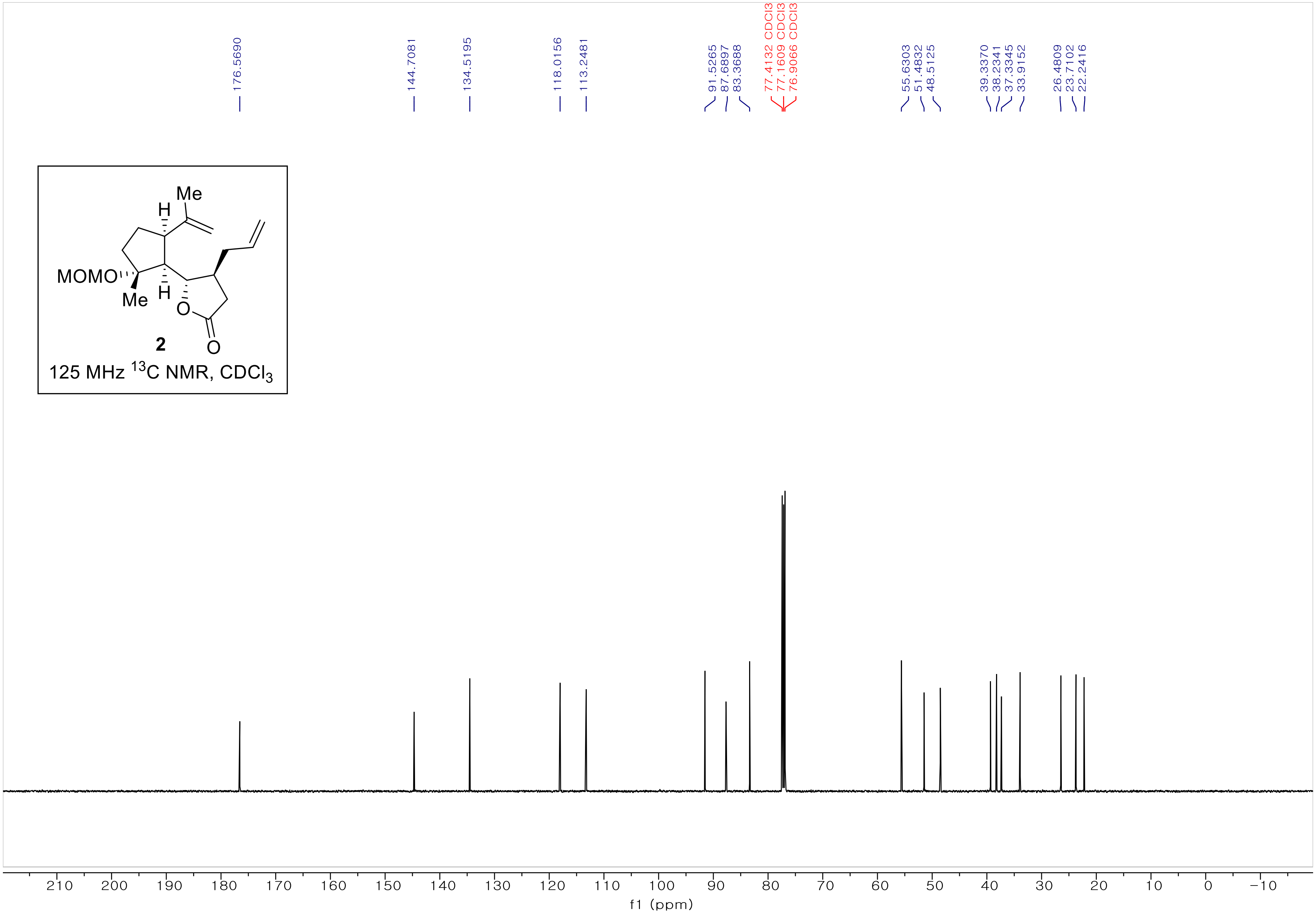

176.5690
144.7081
134.5195
118.0156
113.2481
91.5265
87.6897
83.3688
77.4132 CDCl3
77.1609 CDCl3
76.9066 CDCl3
55.6303
51.4832
48.5125
39.3370
38.2341
37.3345
33.9152
26.4809
23.7102
22.2416
Me
H
MOMO
Me
H
O
O
2
125 MHz ^{13}C NMR, $CDCl_3$
210 200 190 180 170 160 150 140 130 120 110 100 90 80 70 60 50 40 30 20 10 0 −10
f1 (ppm)

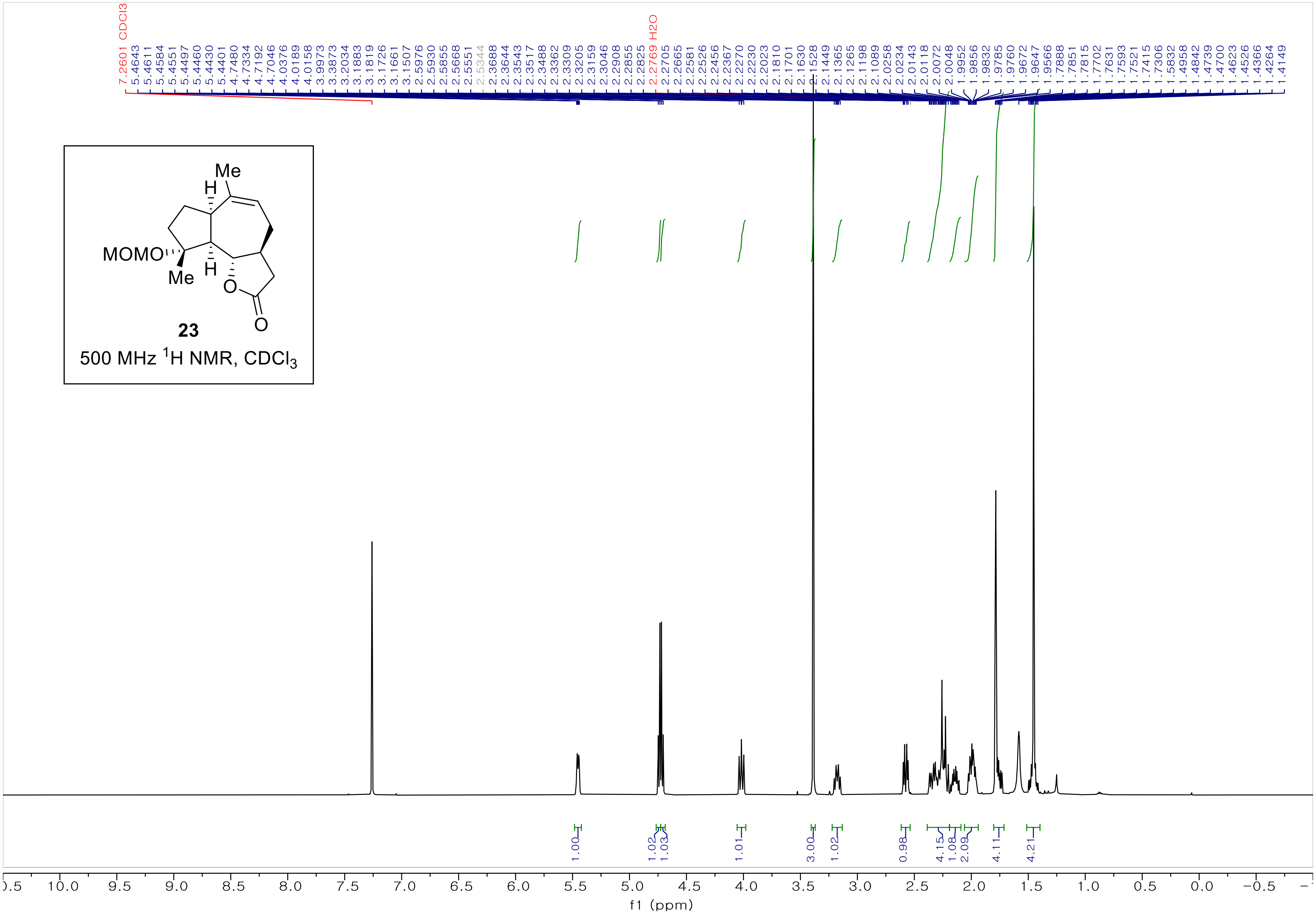

23

500 MHz ^{1}H NMR, $CDCl_3$

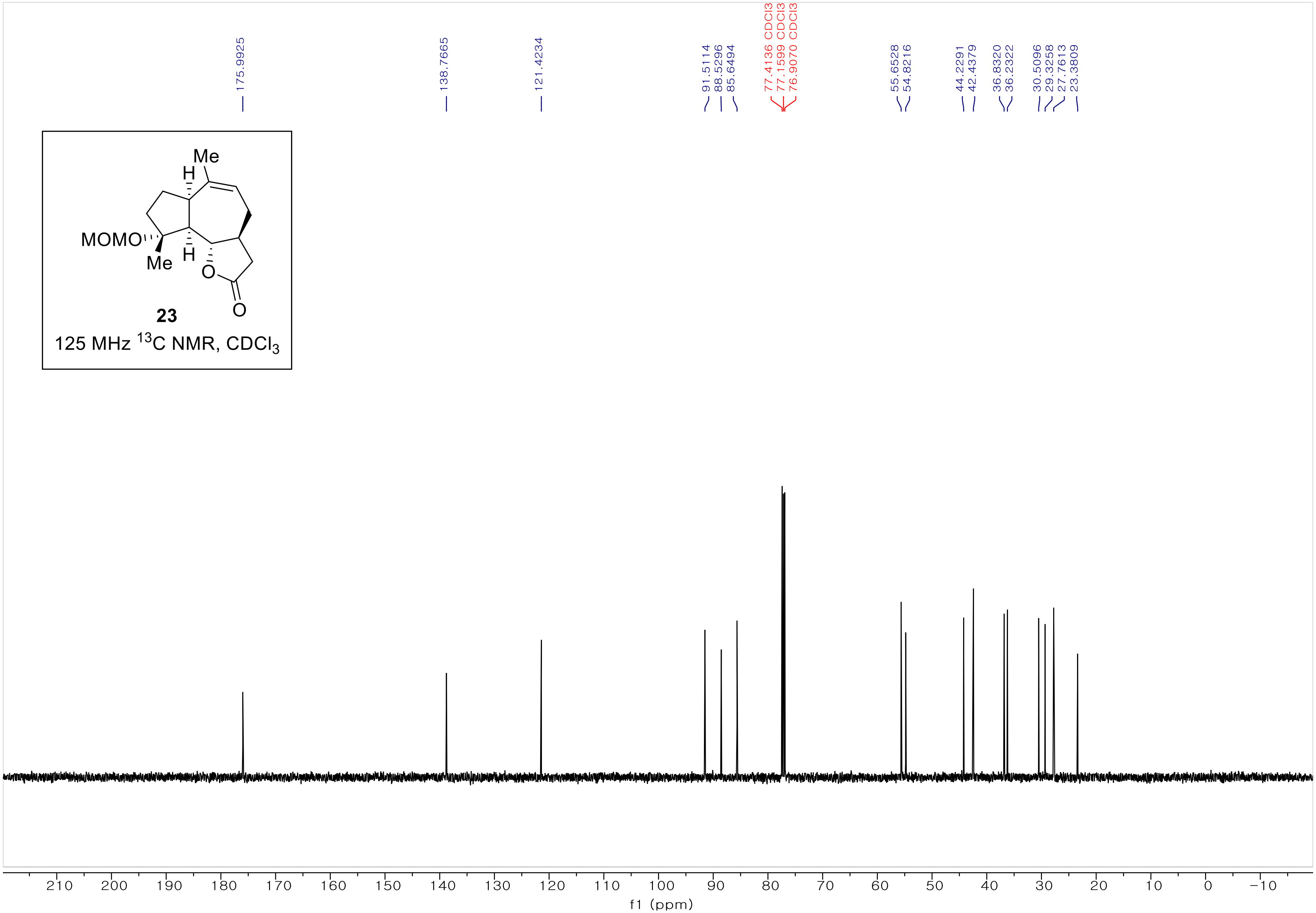
175.9925
138.7665
121.4234
91.5114
88.5296
85.6494
77.4136 CDCl3
77.1599 CDCl3
76.9070 CDCl3
55.6528
54.8216
44.2291
42.4379
36.8320
36.2322
30.5096
29.3258
27.7613
23.3809
Me
H
MOMO
Me
H
O
O
23
125 MHz 13C NMR, CDCl3
210 200 190 180 170 160 150 140 130 120 110 100 90 80 70 60 50 40 30 20 10 0 -10
f1 (ppm)

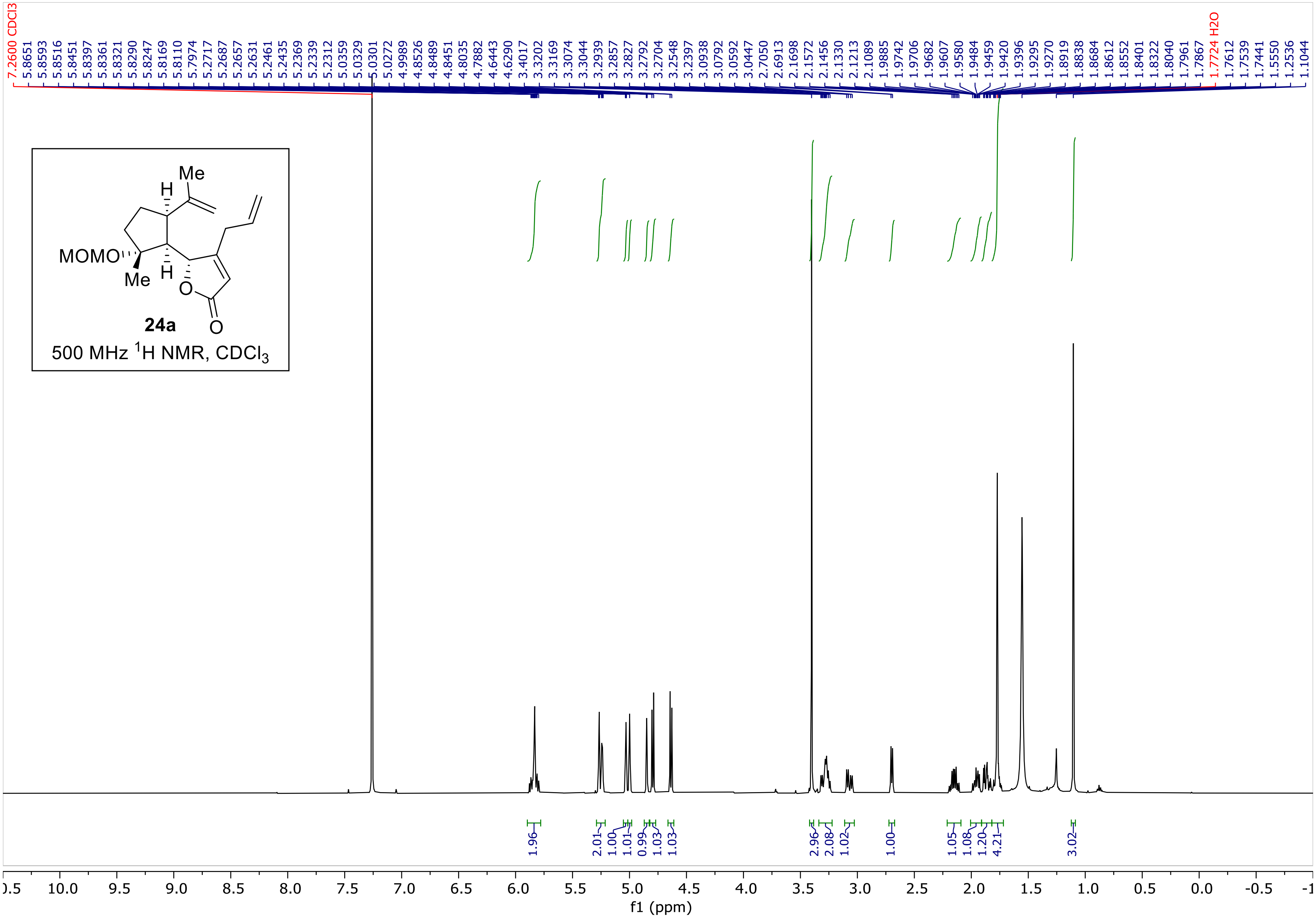
24a
500 MHz ^{1}H NMR, $CDCl_3$
f1 (ppm)

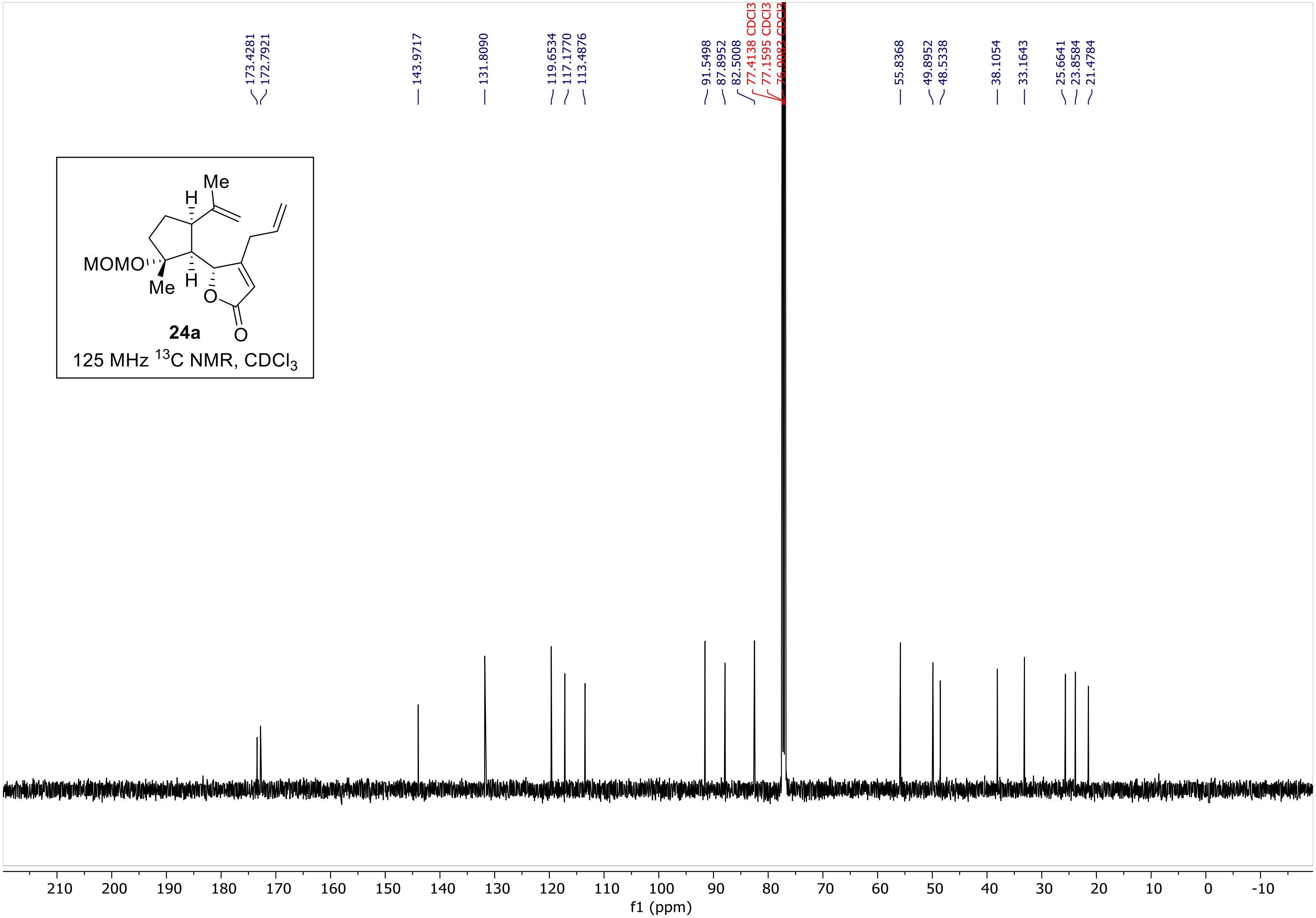
173.4281
172.7921
143.9717
131.8090
119.6534
117.1770
113.4876
91.5498
87.8952
82.5008
77.4138 CDCl3
77.1595 CDCl3
76.9083 CDCl3
55.8368
49.8952
48.5338
38.1054
33.1643
25.6641
23.8584
21.4784
Me
H
MOMO
Me
H
O
O
24a
125 MHz 13C NMR, CDCl3
210
200
190
180
170
160
150
140
130
120
110
100
90
80
70
60
50
40
30
20
10
0
-10
f1 (ppm)

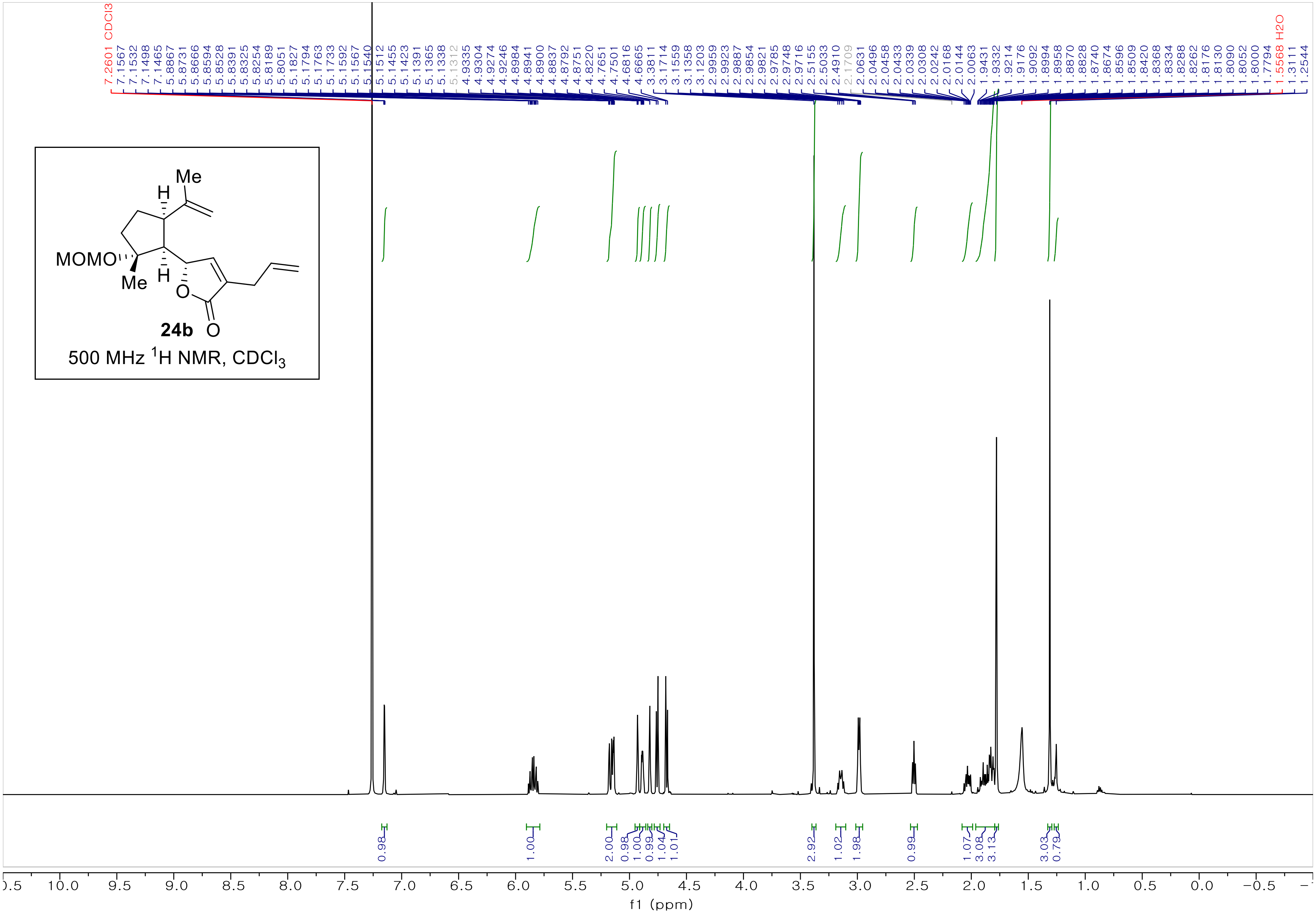
24b
500 MHz 1H NMR, CDCl3
f1 (ppm)

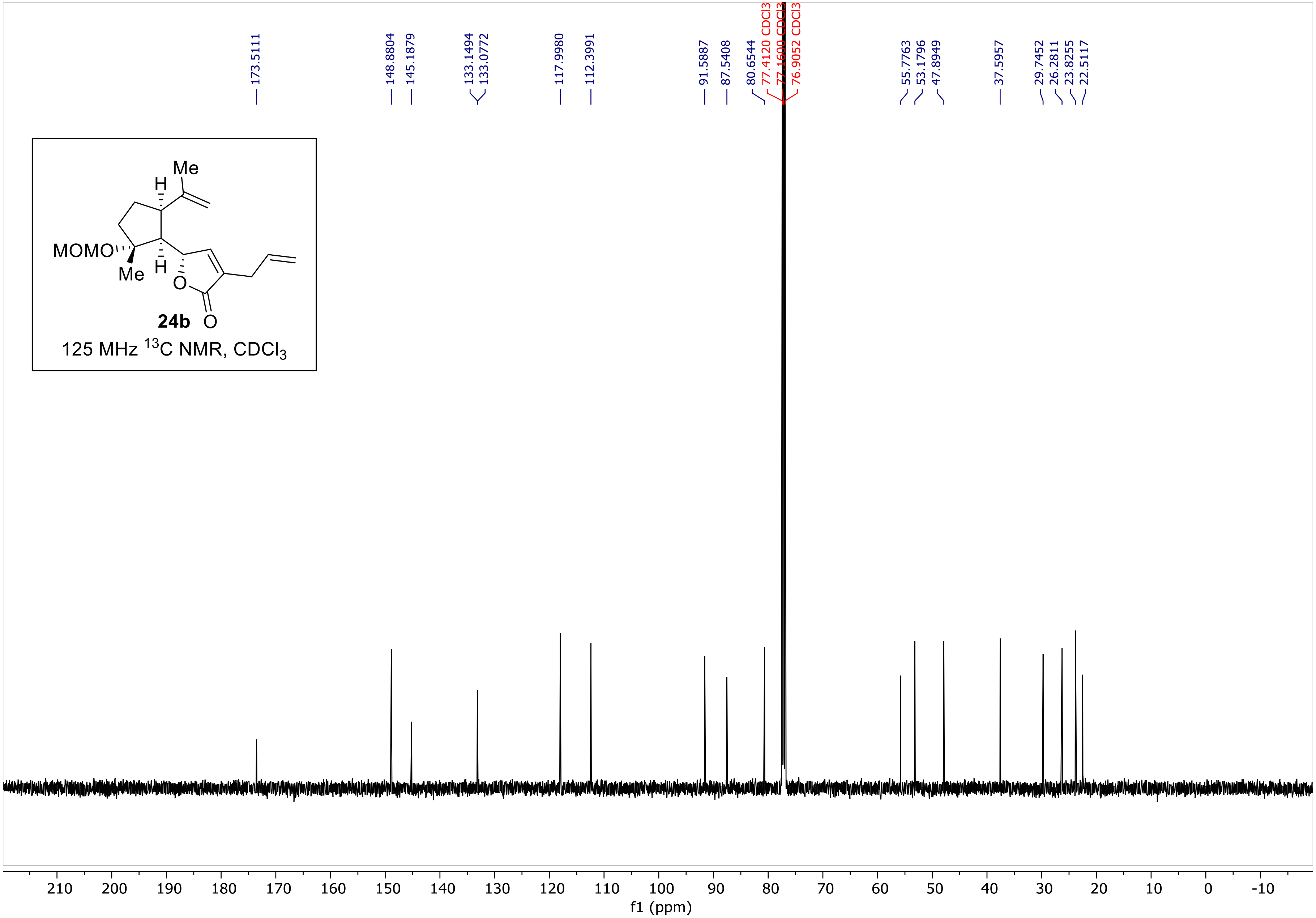
173.5111
148.8804
145.1879
133.1494
133.0772
117.9980
112.3991
91.5887
87.5408
80.6544
77.4120 CDCl3
77.1600 CDCl3
76.9052 CDCl3
55.7763
53.1796
47.8949
37.5957
29.7452
26.2811
23.8255
22.5117
Me
H
MOMO
Me
H
O
24b
O
125 MHz 13C NMR, CDCl3
210 200 190 180 170 160 150 140 130 120 110 100 90 80 70 60 50 40 30 20 10 0 -10
f1 (ppm)

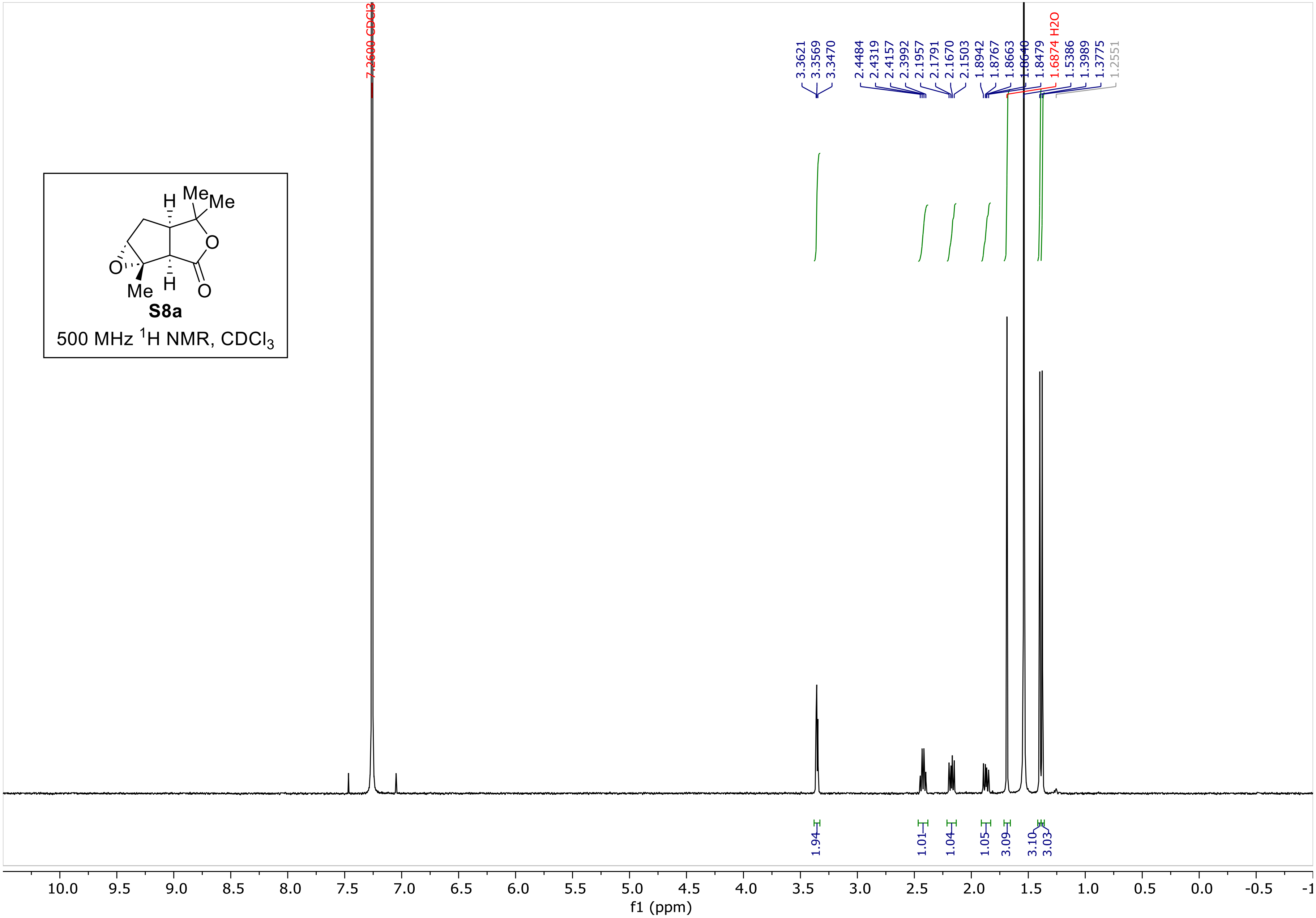

S8a
500 MHz ¹H NMR, CDCl₃
7.2600 CDCl3
3.3621
3.3569
3.3470
2.4484
2.4319
2.4157
2.3992
2.1957
2.1791
2.1670
2.1503
1.8942
1.8767
1.8663
1.8640
1.8479
1.6874 H2O
1.5386
1.3989
1.3775
1.2551
1.94
1.01
1.04
1.05
3.09
3.10
3.03
10.0 9.5 9.0 8.5 8.0 7.5 7.0 6.5 6.0 5.5 5.0 4.5 4.0 3.5 3.0 2.5 2.0 1.5 1.0 0.5 0.0 -0.5 -1
f1 (ppm)

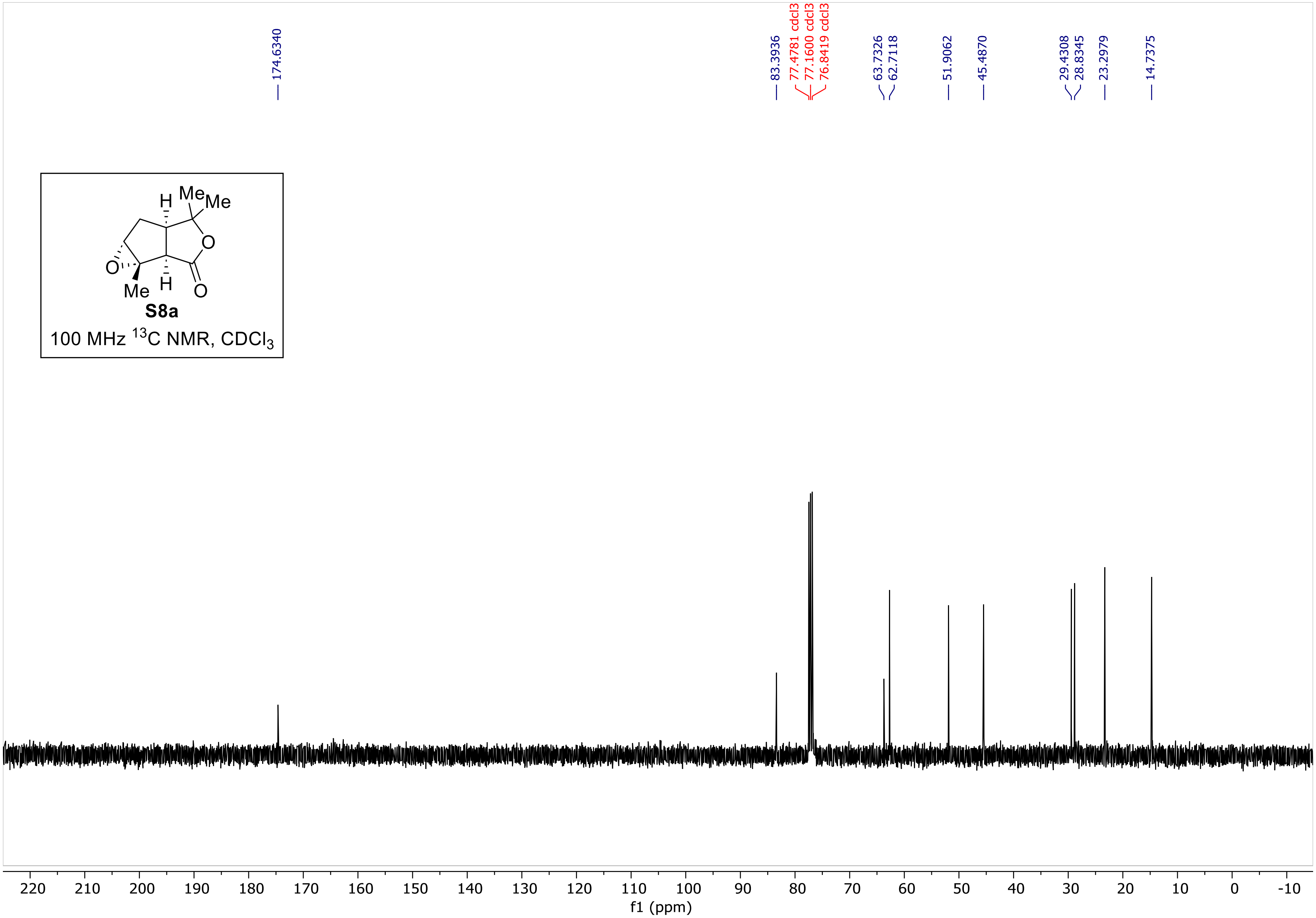
174.6340
83.3936
77.4781 cdcl3
77.1600 cdcl3
76.8419 cdcl3
63.7326
62.7118
51.9062
45.4870
29.4308
28.8345
23.2979
14.7375
S8a
100 MHz 13C NMR, CDCl3
220 210 200 190 180 170 160 150 140 130 120 110 100 90 80 70 60 50 40 30 20 10 0 -10
f1 (ppm)

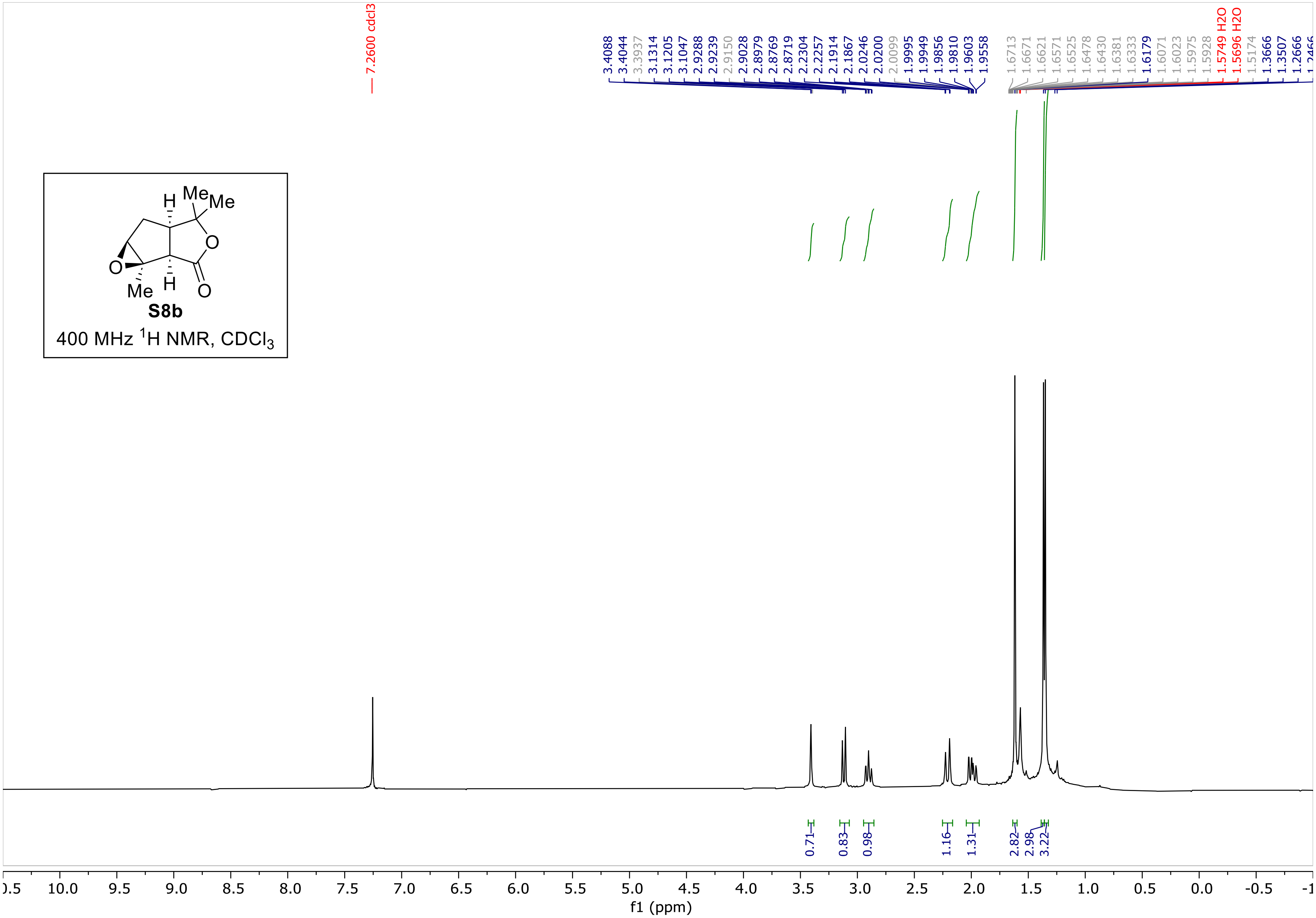
7.2600 cdcl3
3.4088
3.4044
3.3937
3.1314
3.1205
3.1047
2.9288
2.9239
2.9150
2.9028
2.8979
2.8769
2.8719
2.2304
2.2257
2.1914
2.1867
2.0246
2.0200
2.0099
1.9995
1.9949
1.9856
1.9810
1.9603
1.9558
1.6713
1.6671
1.6621
1.6571
1.6525
1.6478
1.6430
1.6381
1.6333
1.6179
1.6071
1.6023
1.5975
1.5928
1.5749 H2O
1.5696 H2O
1.5174
1.3666
1.3507
1.2666
S8b
400 MHz ¹H NMR, CDCl3
0.71
0.83
0.98
1.16
1.31
2.82
2.98
3.22
f1 (ppm)

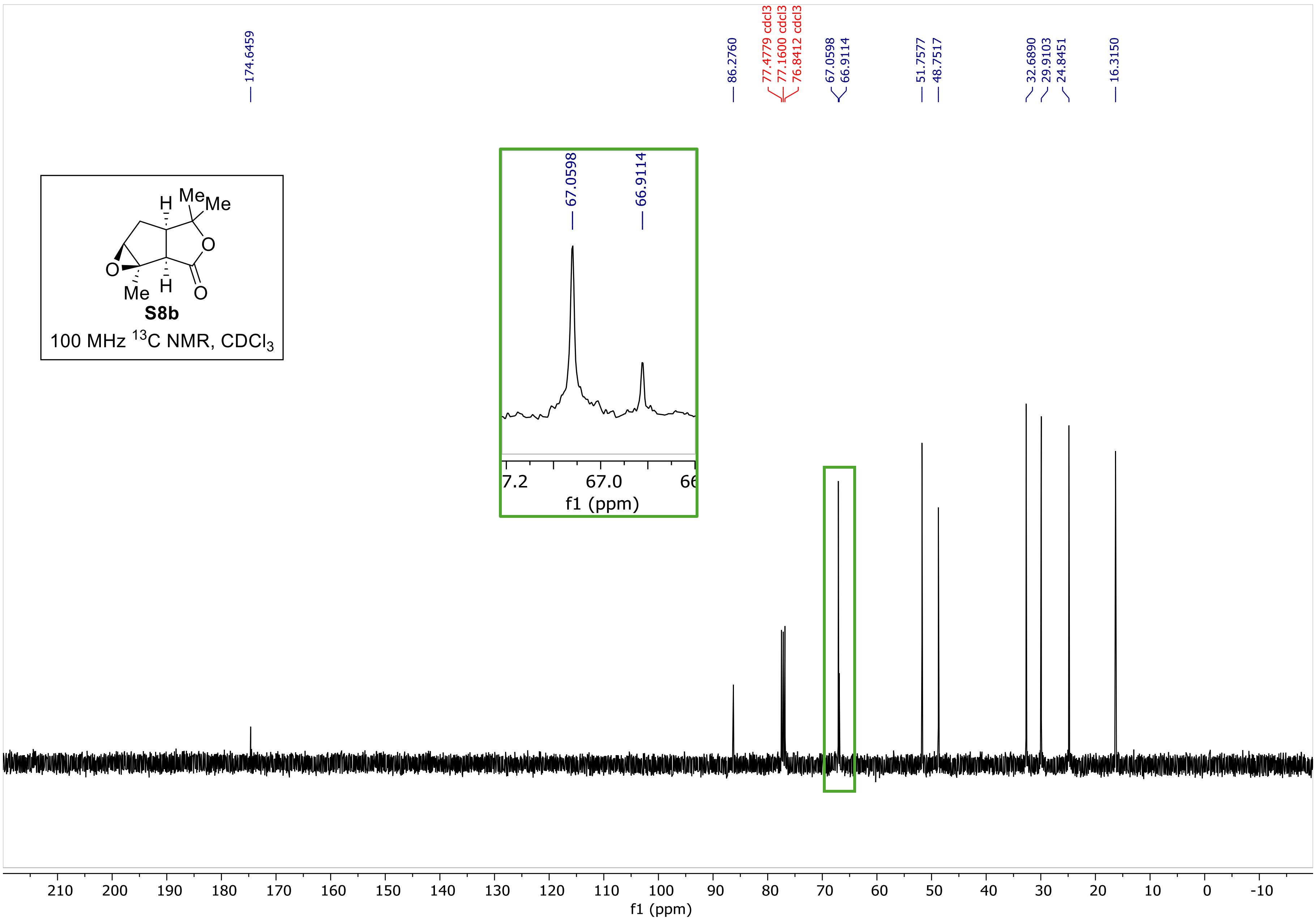

174.6459
86.2760
77.4779 cdcl3
77.1600 cdcl3
76.8412 cdcl3
67.0598
66.9114
51.7577
48.7517
32.6890
29.9103
24.8451
16.3150
H
Me
Me
O
O
Me
H
O
S8b
100 MHz ^{13}C NMR, $CDCl_3$
67.0598
66.9114
7.2
67.0
f1 (ppm)
210
200
190
180
170
160
150
140
130
120
110
100
90
80
70
60
50
40
30
20
10
0
-10
f1 (ppm)

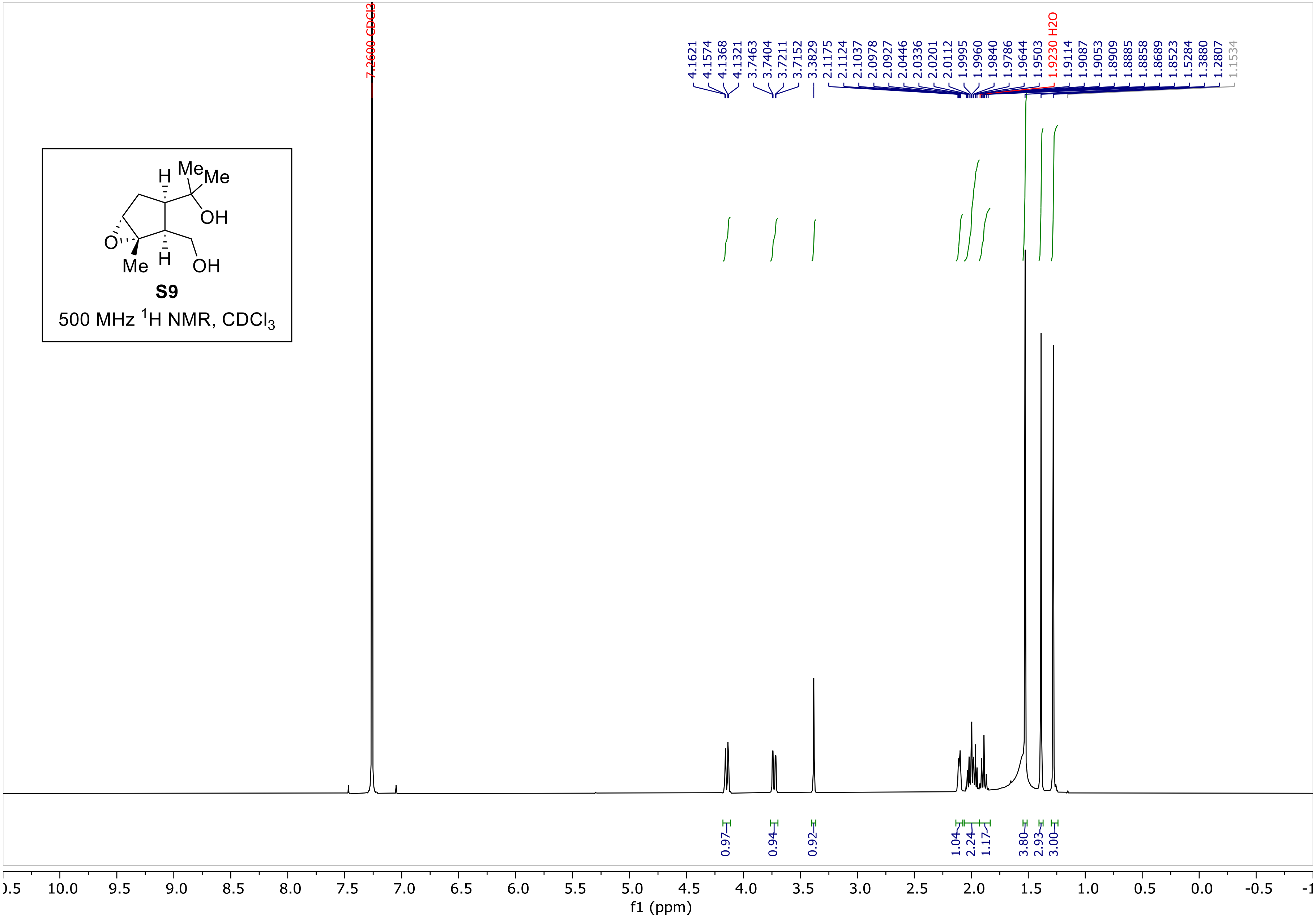

S9
500 MHz 1H NMR, CDCl3
7.2600 CDCl3
4.1621
4.1574
4.1368
4.1321
3.7463
3.7404
3.7211
3.7152
3.3829
2.1175
2.1124
2.1037
2.0978
2.0927
2.0446
2.0336
2.0201
2.0112
1.9995
1.9960
1.9840
1.9786
1.9644
1.9503
1.9230 H2O
1.9114
1.9087
1.9053
1.8909
1.8885
1.8858
1.8689
1.8523
1.5284
1.3880
1.2807
1.1534
0.97
0.94
0.92
1.04
2.24
1.17
3.80
2.93
3.00
f1 (ppm)

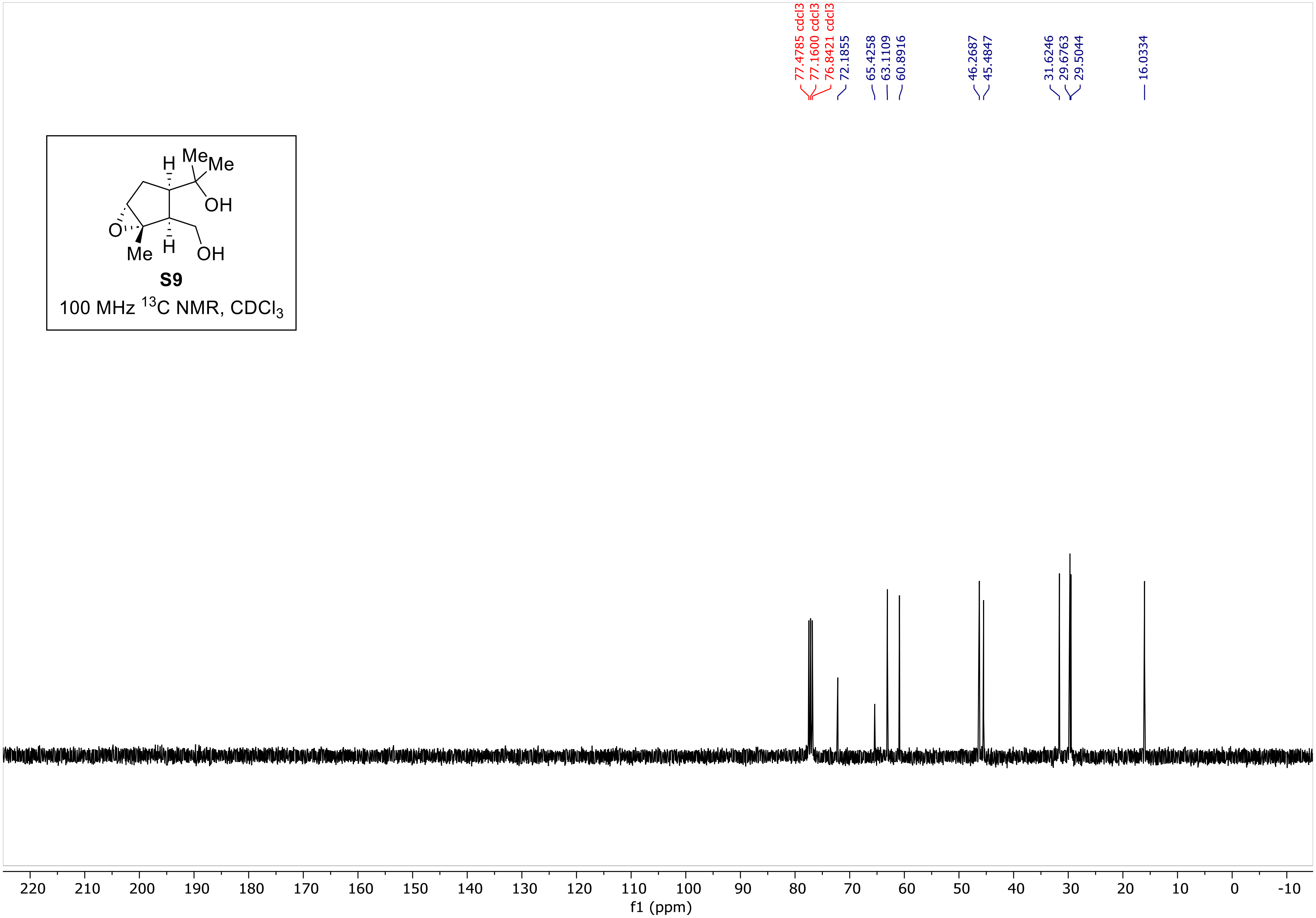
77.4785 cdcl3
77.1600 cdcl3
76.8421 cdcl3
72.1855
65.4258
63.1109
60.8916
46.2687
45.4847
31.6246
29.6763
29.5044
16.0334
H
Me
Me
OH
O
Me
H
OH
S9
100 MHz ¹³C NMR, CDCl₃
220 210 200 190 180 170 160 150 140 130 120 110 100 90 80 70 60 50 40 30 20 10 0 -10
f1 (ppm)

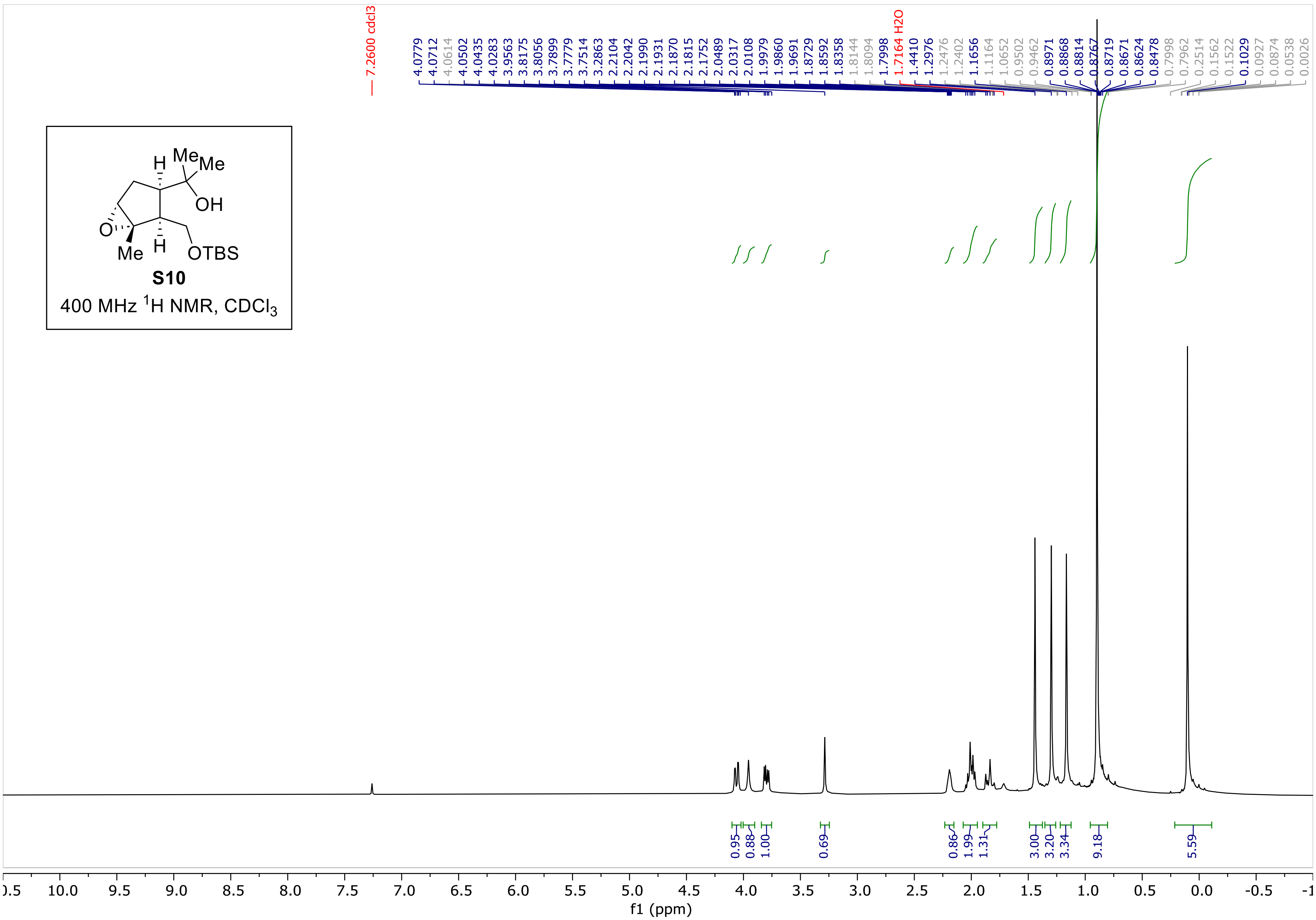

H
Me
Me
OH
O
Me
H
OTBS
S10
400 MHz ¹H NMR, CDCl₃
7.2600 cdcl3
4.0779
4.0712
4.0614
4.0502
4.0435
4.0283
3.9563
3.8175
3.8056
3.7899
3.7779
3.7514
3.2863
2.2104
2.2042
2.1990
2.1931
2.1870
2.1815
2.1752
2.0489
2.0317
2.0108
1.9979
1.9860
1.9691
1.8729
1.8592
1.8358
1.8144
1.8094
1.7998
1.7164 H2O
1.4410
1.2976
1.2476
1.2402
1.1656
1.1164
1.0652
0.9502
0.9462
0.8971
0.8868
0.8814
0.8767
0.8719
0.8671
0.8624
0.8478
0.7998
0.7962
0.2514
0.1562
0.1522
0.1029
0.0927
0.0874
0.0538
0.0026
0.95
0.88
1.00
0.69
0.86
1.99
1.31
3.00
3.20
3.34
9.18
5.59
f1 (ppm)

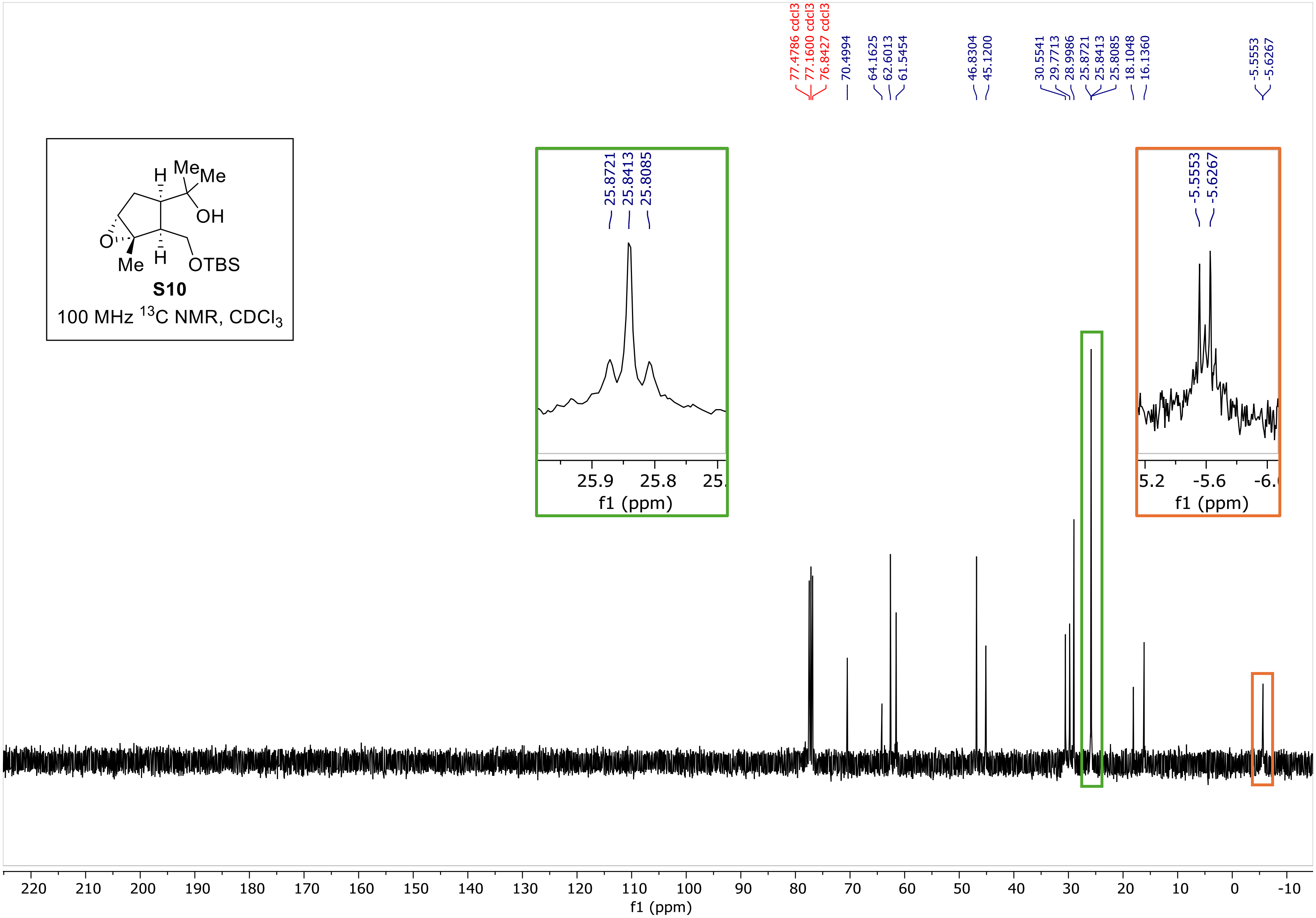
77.4786 cdcl3
77.1600 cdcl3
76.8427 cdcl3
70.4994
64.1625
62.6013
61.5454
46.8304
45.1200
30.5541
29.7713
28.9986
25.8721
25.8413
25.8085
18.1048
16.1360
-5.5553
-5.6267
H
Me
Me
OH
O
Me
H
OTBS
S10
100 MHz ^{13}C NMR, $CDCl_3$
f1 (ppm)

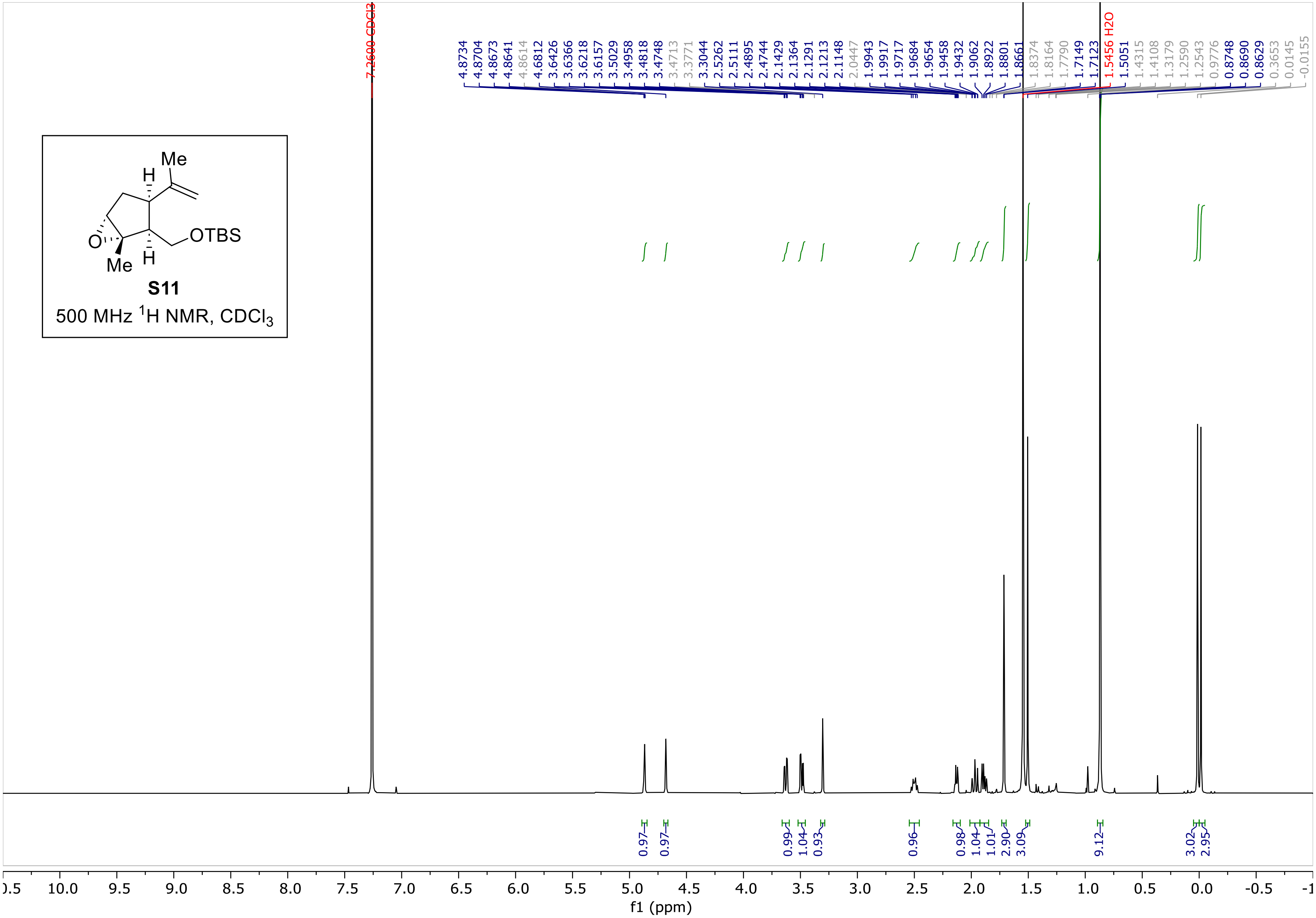

Me
H
OTBS
O
Me
H
S11
500 MHz 1H NMR, CDCl3
7.2600 CDCl3
1.5456 H2O
f1 (ppm)

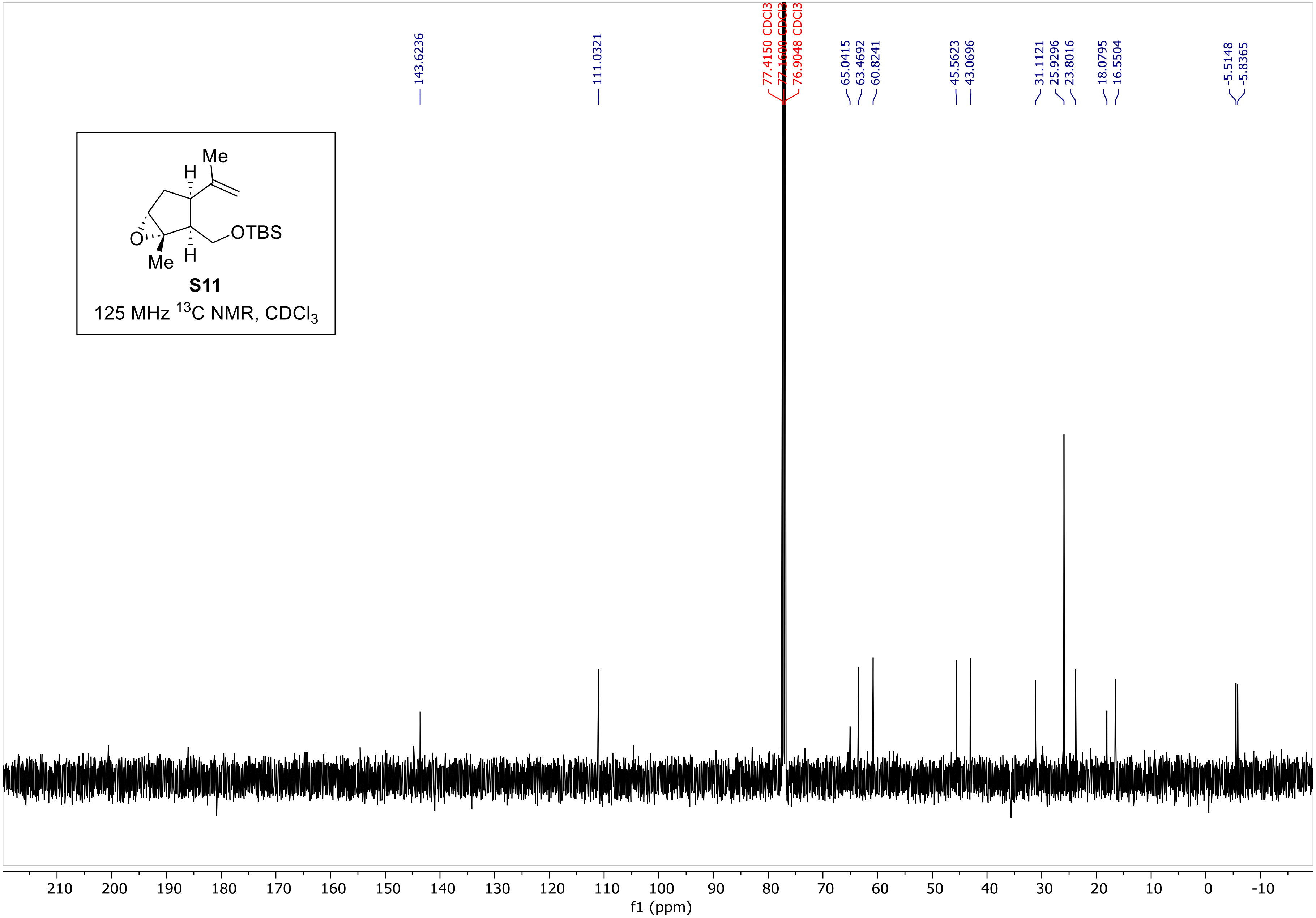
143.6236
111.0321
77.4150 CDCl3
77.1600 CDCl3
76.9048 CDCl3
65.0415
63.4692
60.8241
45.5623
43.0696
31.1121
25.9296
23.8016
18.0795
16.5504
-5.5148
-5.8365
Me
H
OTBS
O
Me
H
S11
125 MHz ^{13}C NMR, $CDCl_3$
210 200 190 180 170 160 150 140 130 120 110 100 90 80 70 60 50 40 30 20 10 0 -10
f1 (ppm)

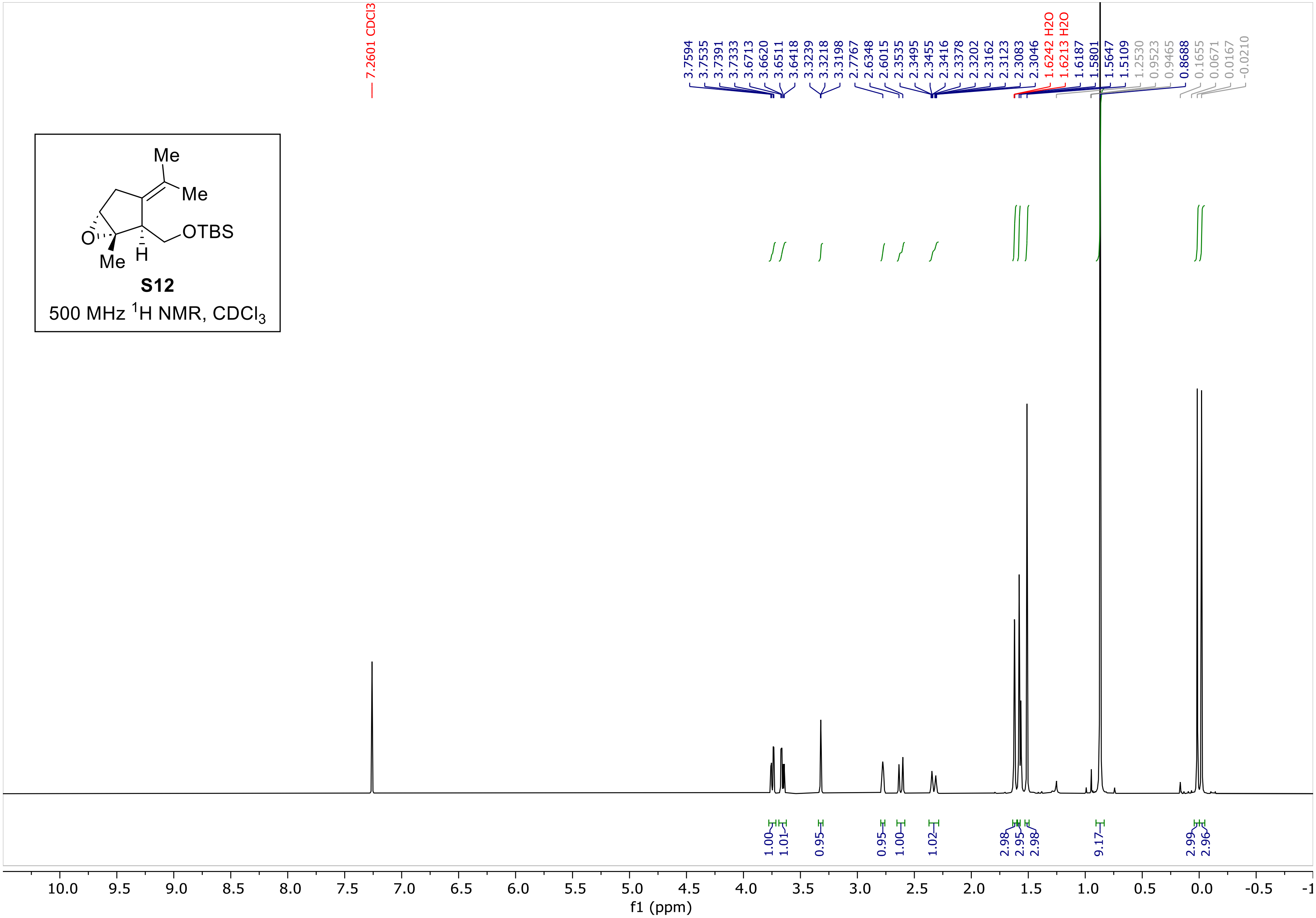

Me
Me
OTBS
O
Me
H
S12
500 MHz 1H NMR, CDCl3
7.2601 CDCl3
3.7594
3.7535
3.7391
3.7333
3.6713
3.6620
3.6511
3.6418
3.3239
3.3218
3.3198
2.7767
2.6348
2.6015
2.3535
2.3495
2.3455
2.3416
2.3378
2.3202
2.3162
2.3123
2.3083
2.3046
1.6242 H2O
1.6213 H2O
1.6187
1.5801
1.5647
1.5109
1.2530
0.9523
0.9465
0.8688
0.1655
0.0671
0.0167
-0.0210
1.00
1.01
0.95
0.95
1.00
1.02
2.98
2.95
2.98
9.17
2.99
2.96
10.0
9.5
9.0
8.5
8.0
7.5
7.0
6.5
6.0
5.5
5.0
4.5
4.0
3.5
3.0
2.5
2.0
1.5
1.0
0.5
0.0
-0.5
-1
f1 (ppm)

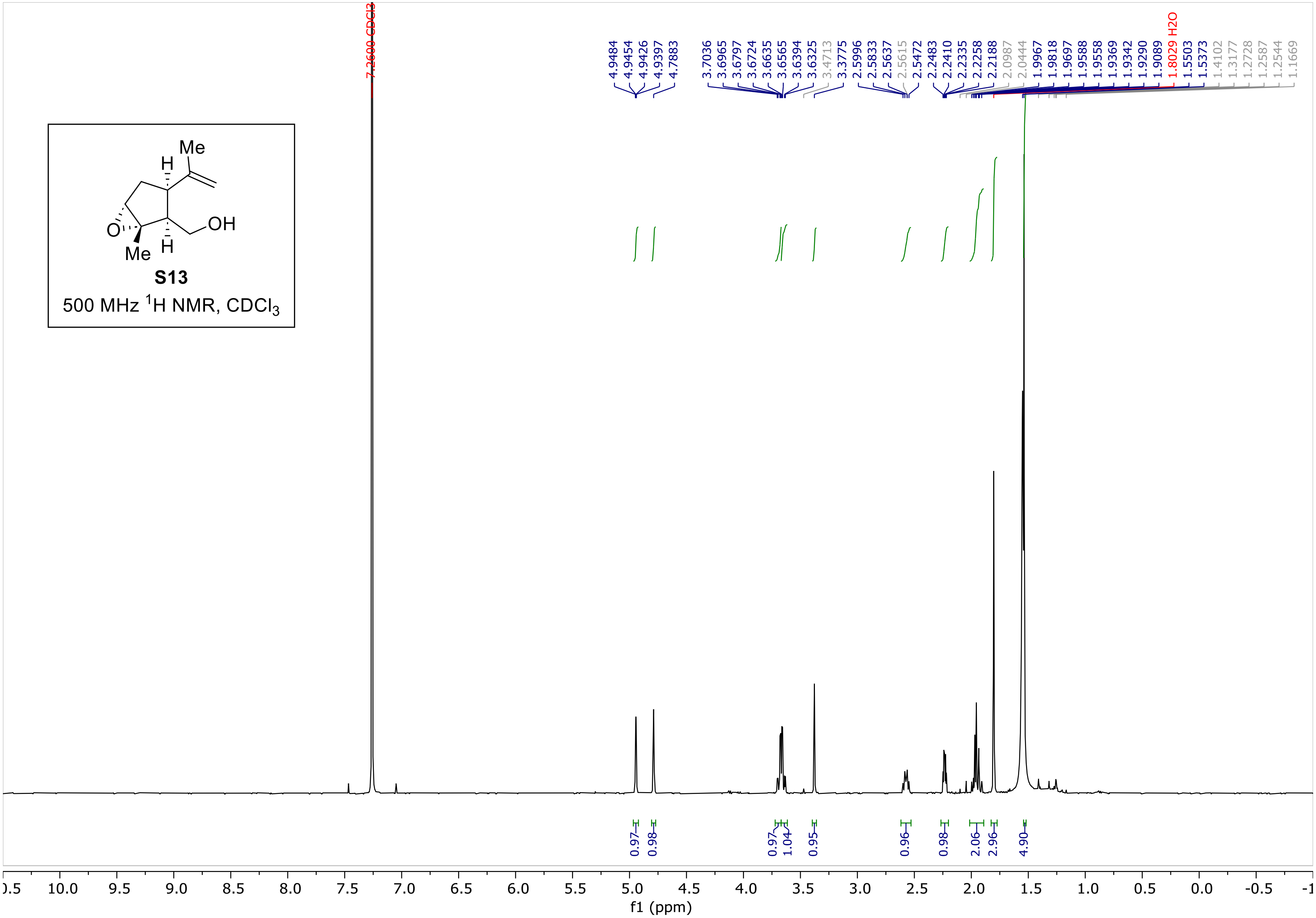
S13
500 MHz ¹H NMR, CDCl₃
f1 (ppm)

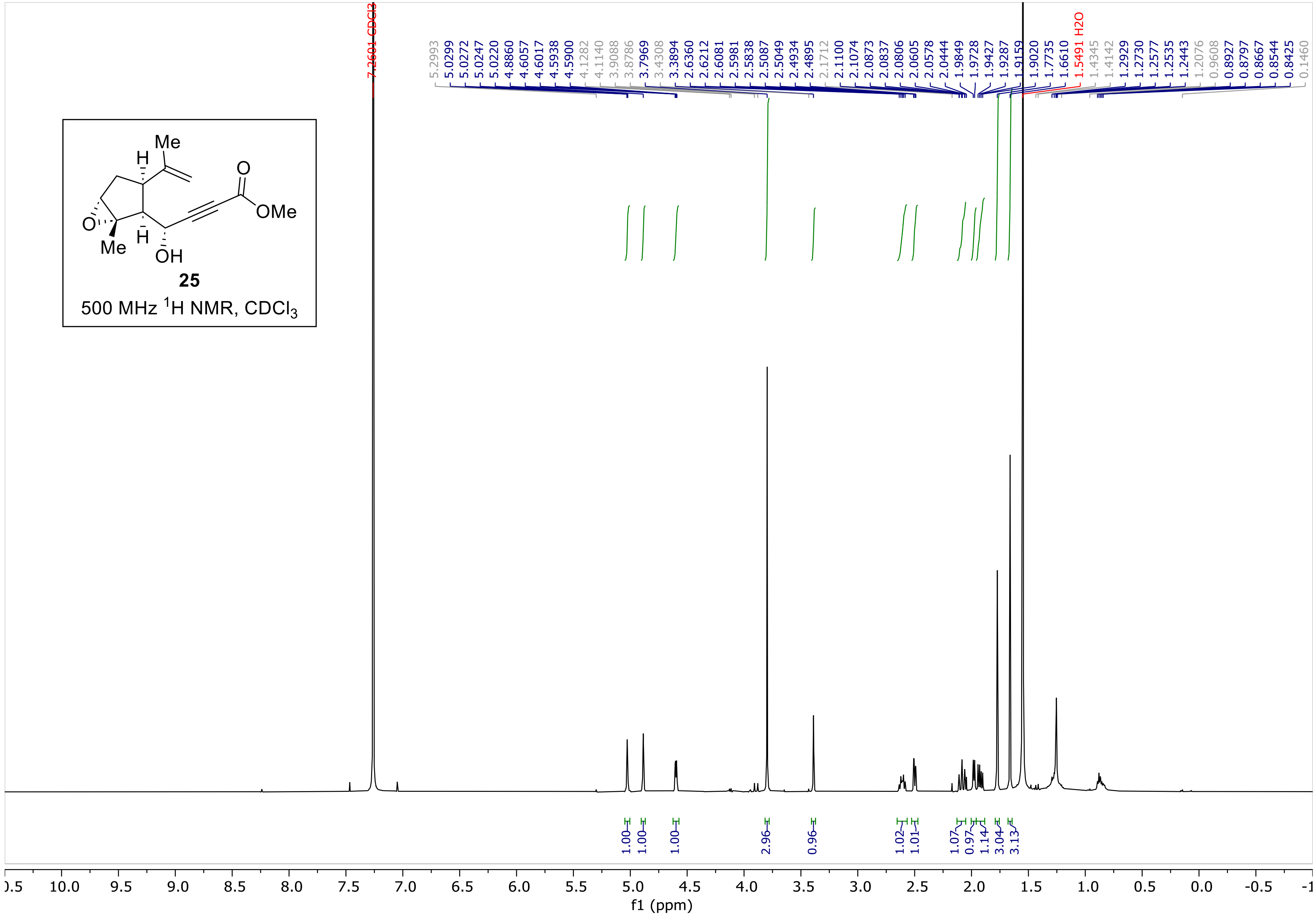
Me
H
O
OMe
O
Me
H
OH
25
500 MHz ^{1}H NMR, $CDCl_3$
7.2601 CDCl3
5.2993
5.0299
5.0272
5.0247
5.0220
4.8860
4.6057
4.6017
4.5938
4.5900
4.1282
4.1140
3.9088
3.8786
3.7969
3.4308
3.3894
2.6360
2.6212
2.6081
2.5981
2.5838
2.5087
2.5049
2.4934
2.4895
2.1712
2.1100
2.1074
2.0873
2.0837
2.0806
2.0605
2.0578
2.0444
1.9849
1.9728
1.9427
1.9287
1.9159
1.9020
1.7735
1.6610
1.5491 H2O
1.4345
1.4142
1.2929
1.2730
1.2577
1.2535
1.2443
1.2076
0.9608
0.8927
0.8797
0.8667
0.8544
0.8425
0.1460
1.00
1.00
1.00
2.96
0.96
1.02
1.01
1.07
0.97
1.14
3.04
3.13
f1 (ppm)

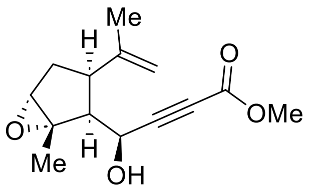

S14

500 MHz ^{1}H NMR, $CDCl_3$

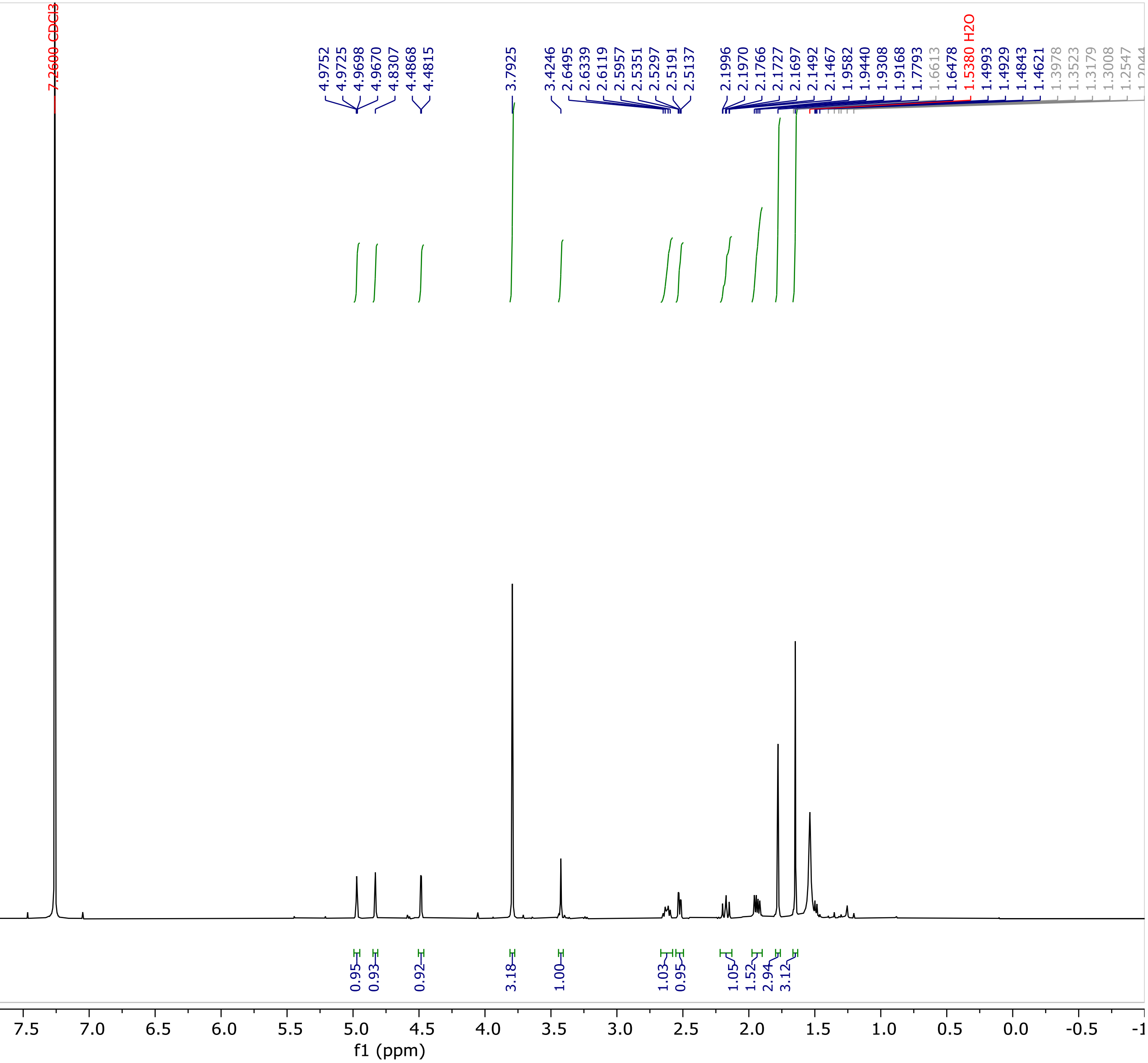

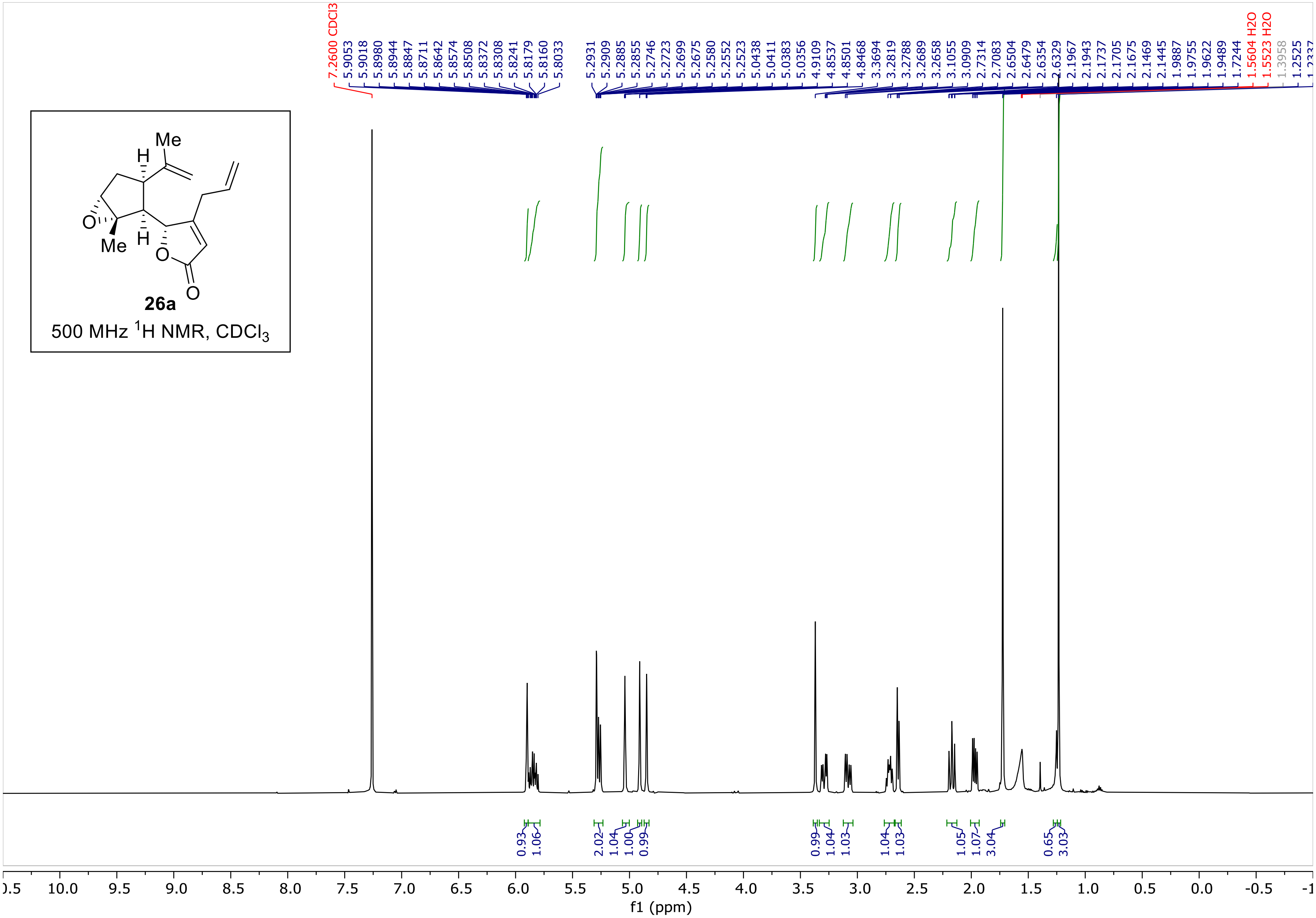

26a
500 MHz ^{1}H NMR, $CDCl_3$
f1 (ppm)

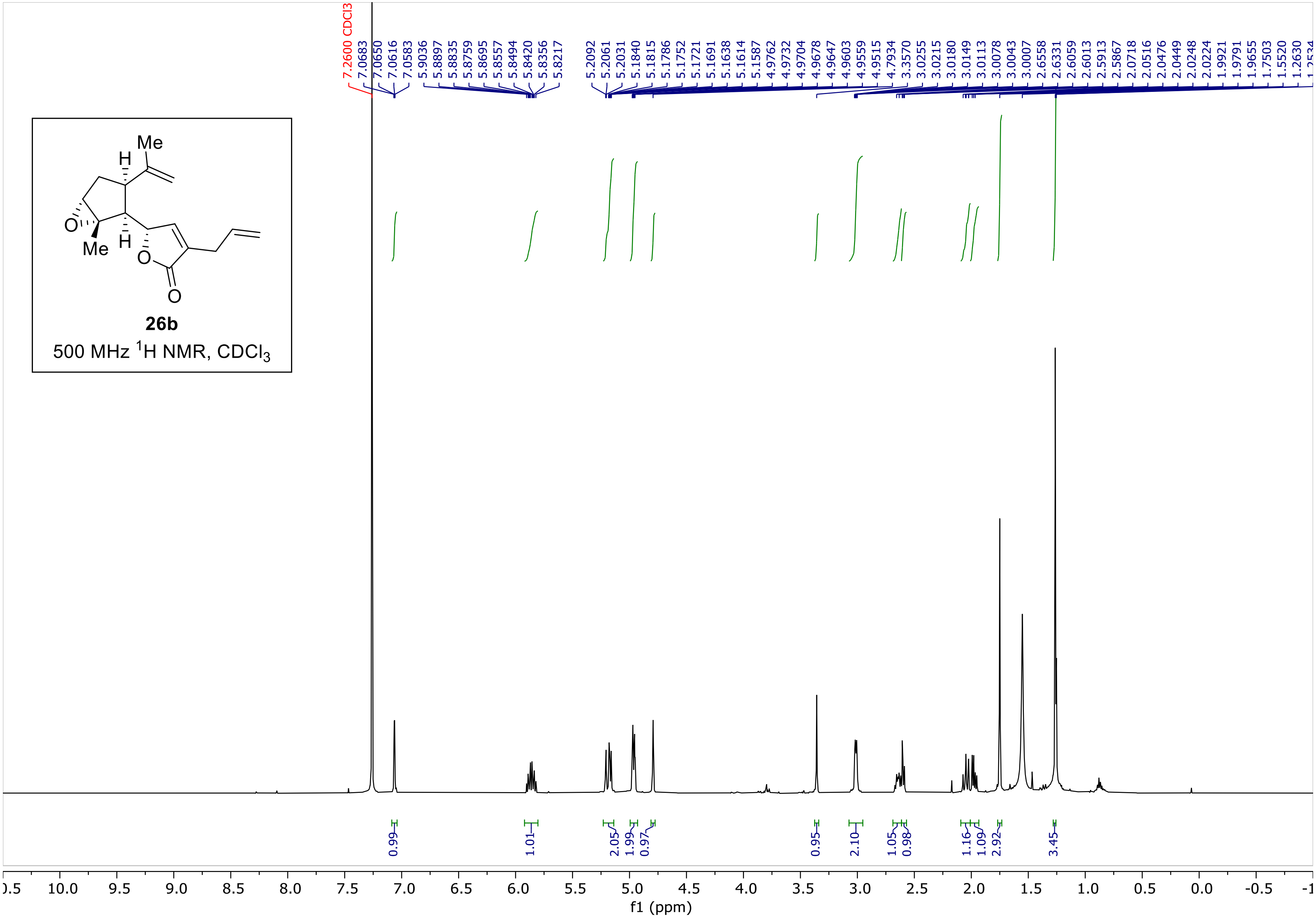
Me
H
O
Me
H
O
O
26b
500 MHz ¹H NMR, CDCl₃
f1 (ppm)

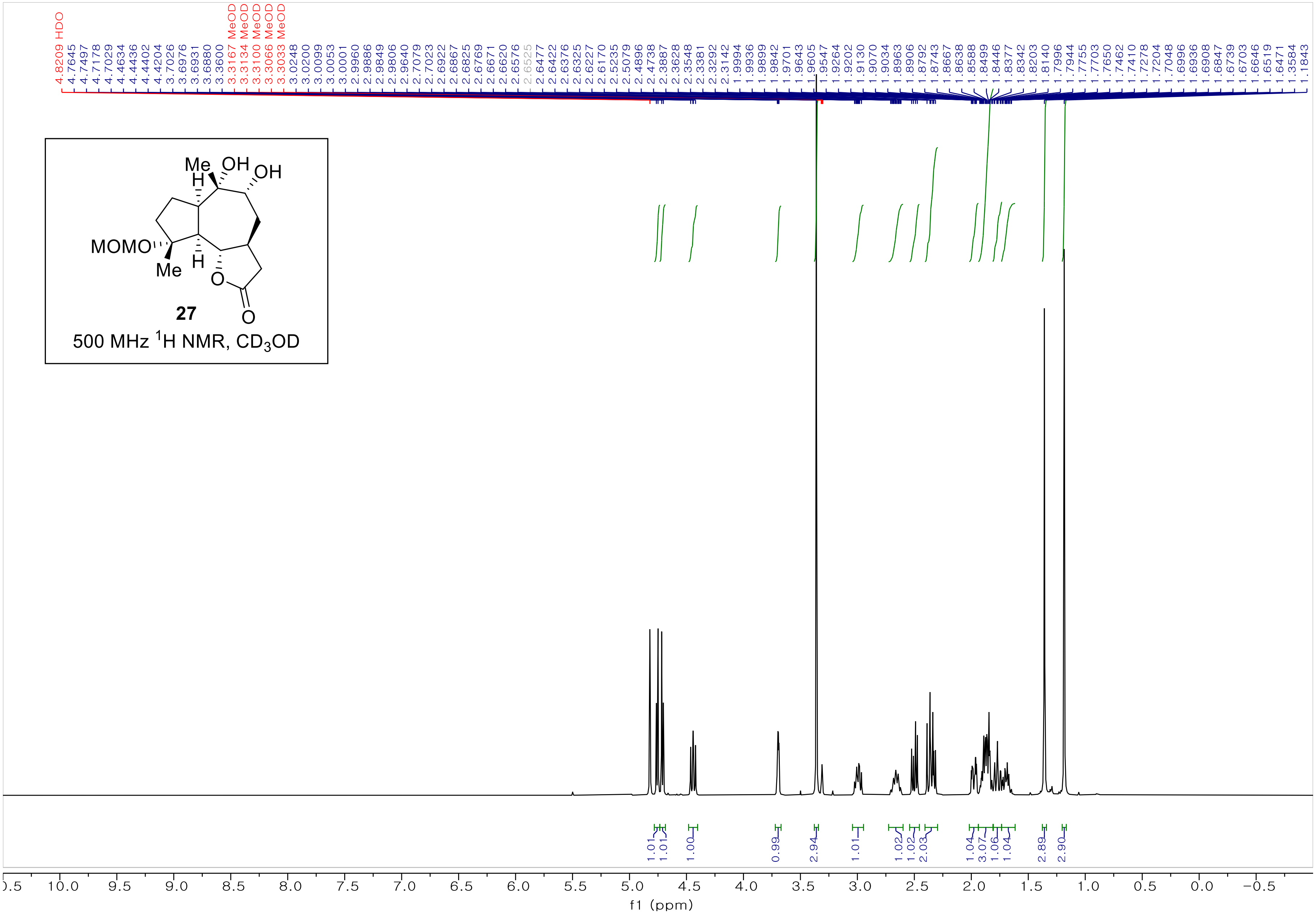

MOMO
Me
OH
OH
H
H
O
O
27
500 MHz ^{1}H NMR, CD_3OD
f1 (ppm)

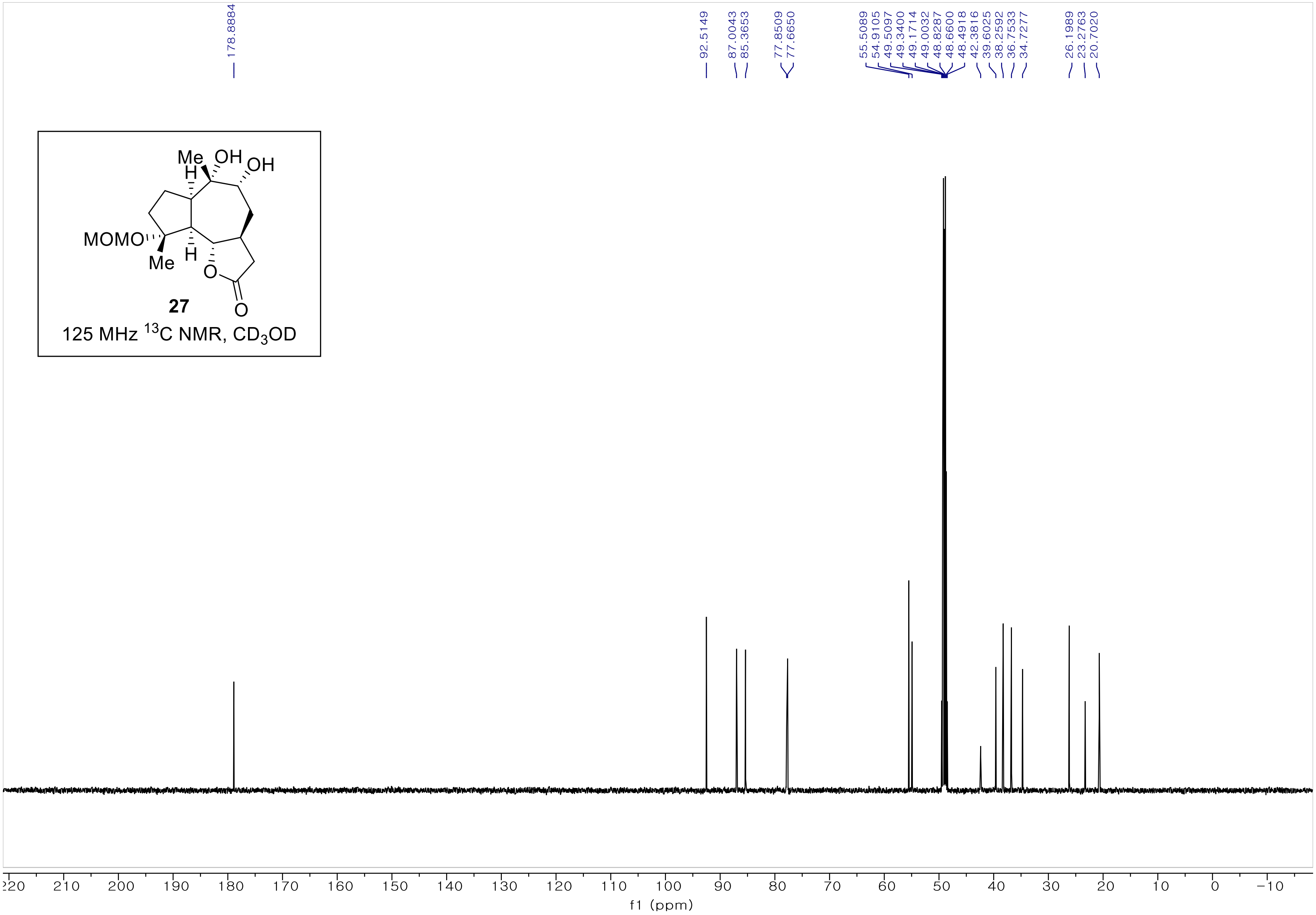
178.8884
92.5149
87.0043
85.3653
77.8509
77.6650
55.5089
54.9105
49.5097
49.3400
49.1714
49.0032
48.8287
48.6600
48.4918
42.3816
39.6025
38.2592
36.7533
34.7277
26.1989
23.2763
20.7020
Me
OH
OH
H
MOMO
Me
H
O
O
27
125 MHz 13C NMR, CD3OD
220
210
200
190
180
170
160
150
140
130
120
110
100
90
80
70
60
50
40
30
20
10
0
−10
f1 (ppm)

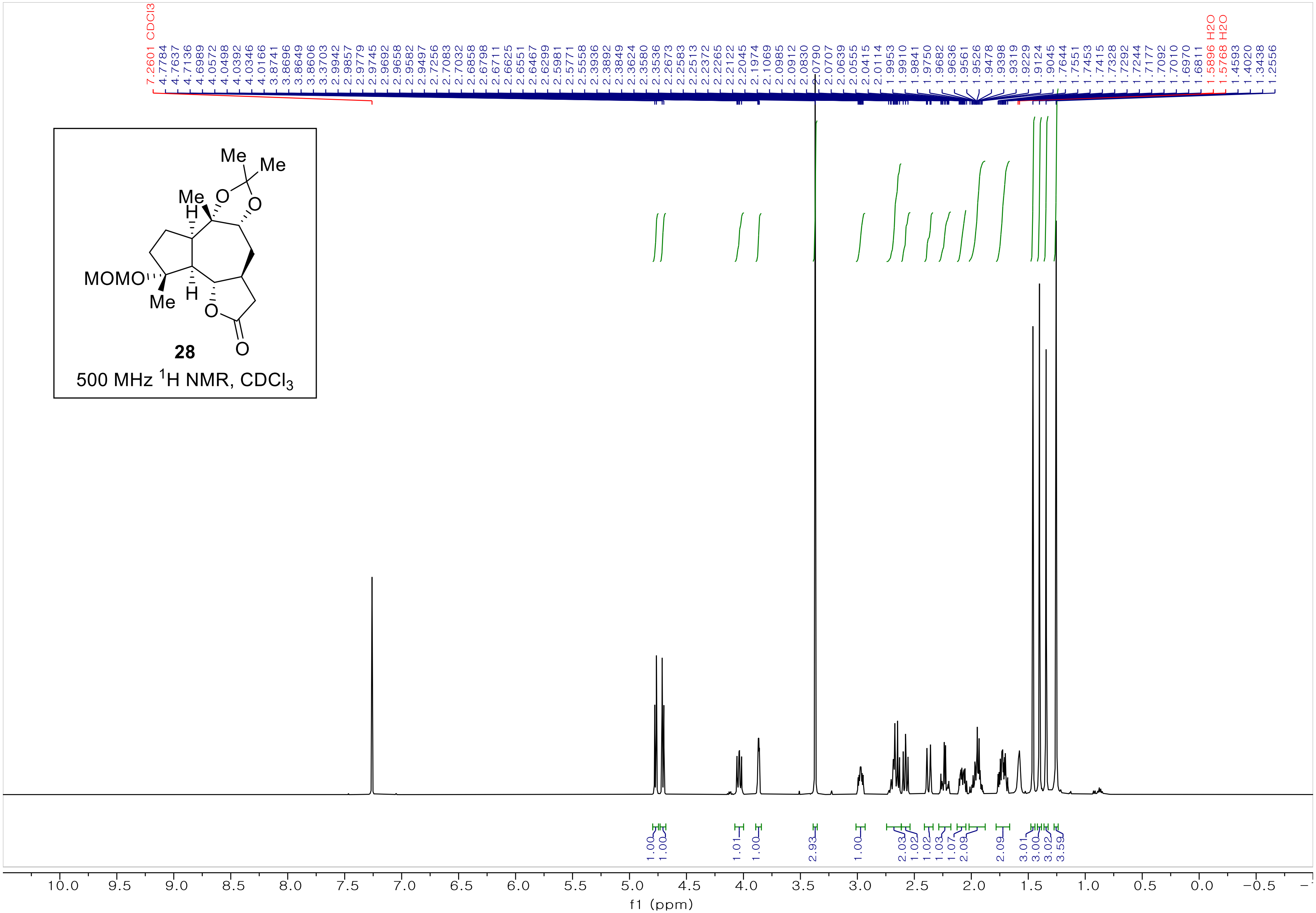
Me
Me
Me
O
O
H
MOMO
Me
H
O
O
28
500 MHz ^{1}H NMR, CDCl$_3$
f1 (ppm)

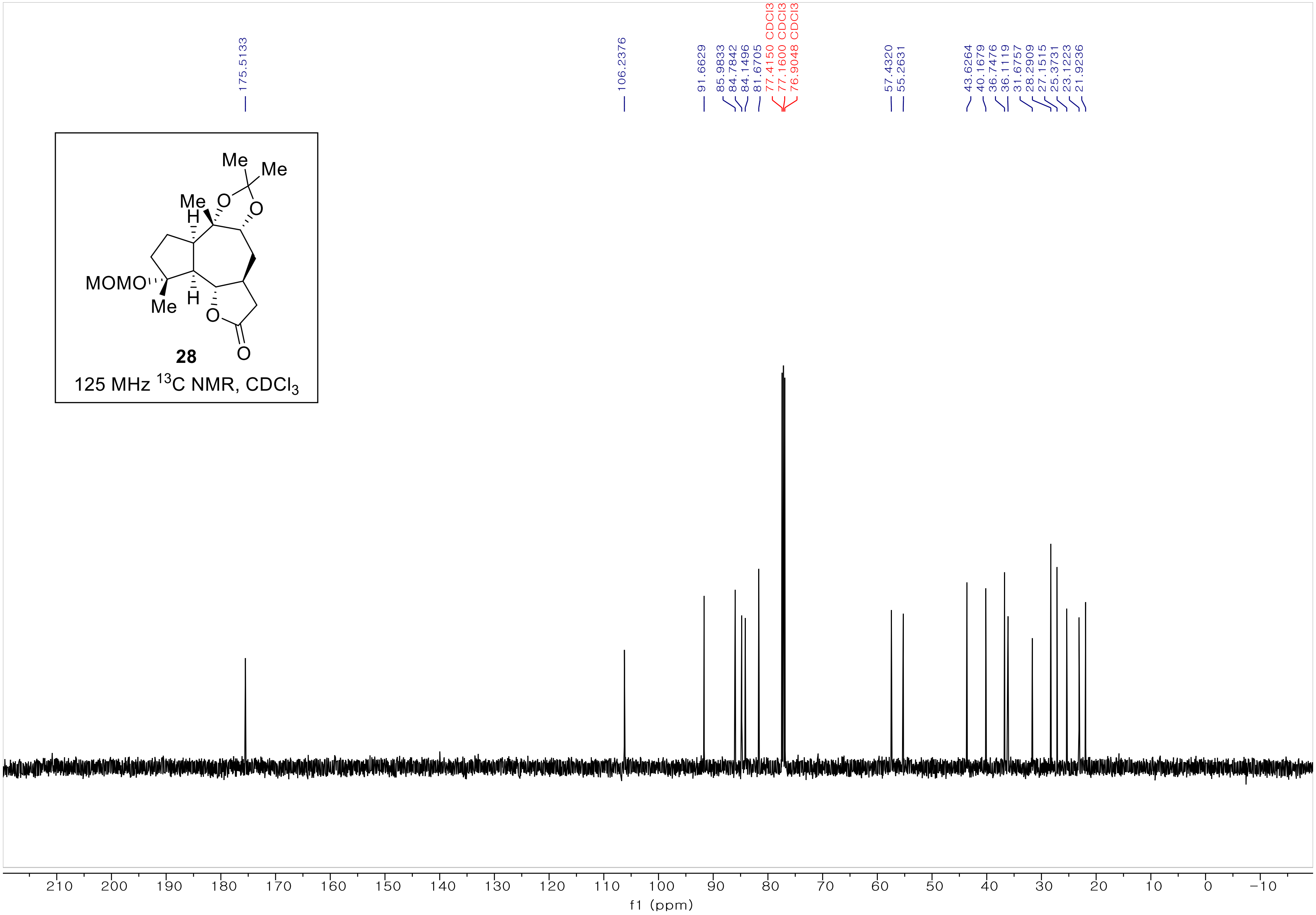

175.5133
106.2376
91.6629
85.9833
84.7842
84.1496
81.6705
77.4150 CDCl3
77.1600 CDCl3
76.9048 CDCl3
57.4320
55.2631
43.6264
40.1679
36.7476
36.1119
31.6757
28.2909
27.1515
25.3731
23.1223
21.9236
Me
Me
Me
O
O
H
MOMO
H
Me
O
O
28
125 MHz ^{13}C NMR, $CDCl_3$
210
200
190
180
170
160
150
140
130
120
110
100
90
80
70
60
50
40
30
20
10
0
−10
f1 (ppm)

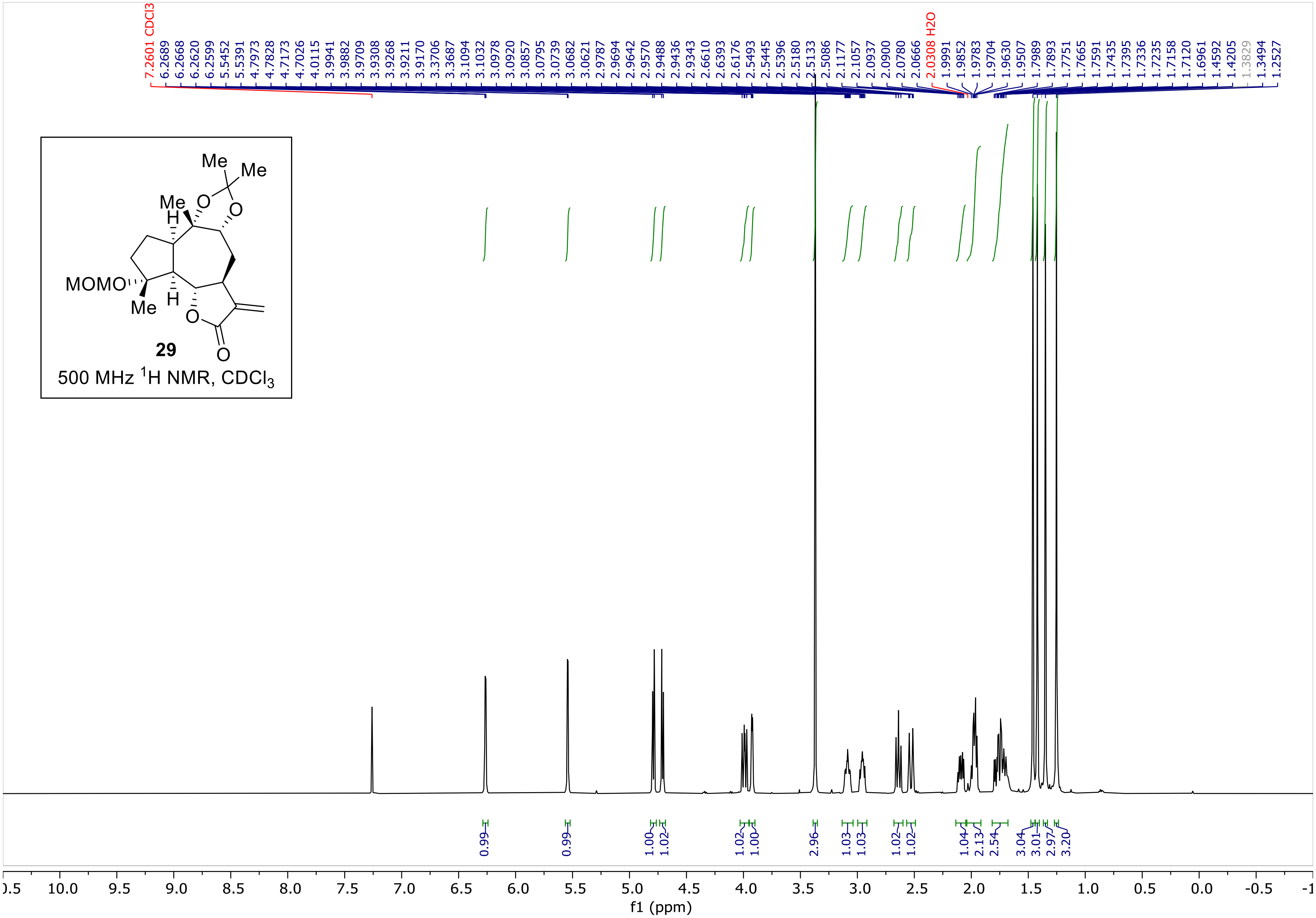

29
500 MHz ^{1}H NMR, $CDCl_3$
f1 (ppm)

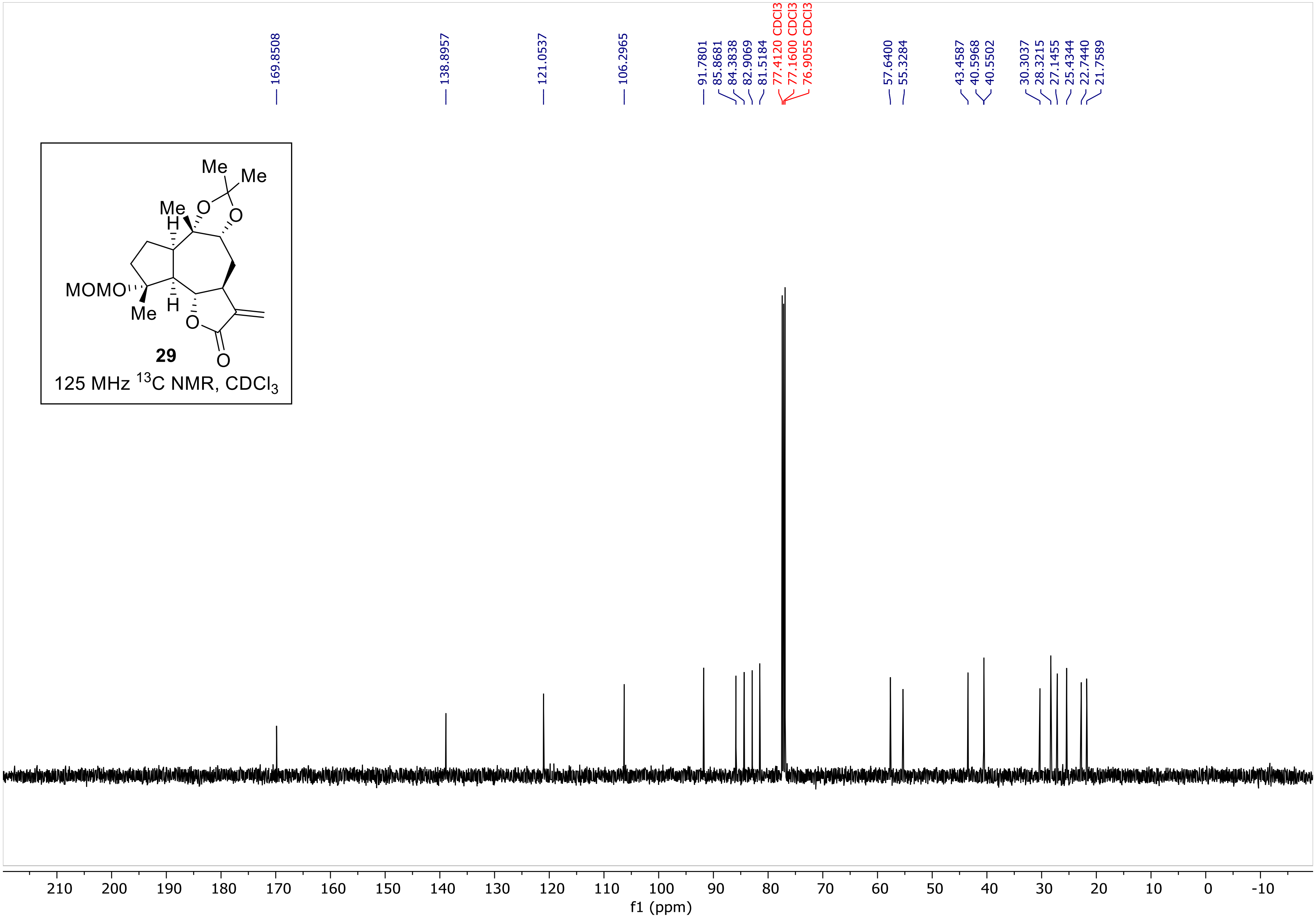
169.8508
138.8957
121.0537
106.2965
91.7801
85.8681
84.3838
82.9069
81.5184
77.4120 CDCl3
77.1600 CDCl3
76.9055 CDCl3
57.6400
55.3284
43.4587
40.5968
40.5502
30.3037
28.3215
27.1455
25.4344
22.7440
21.7589
Me
Me
Me
O
O
H
MOMO
Me
H
O
O
29
125 MHz 13C NMR, CDCl3
f1 (ppm)

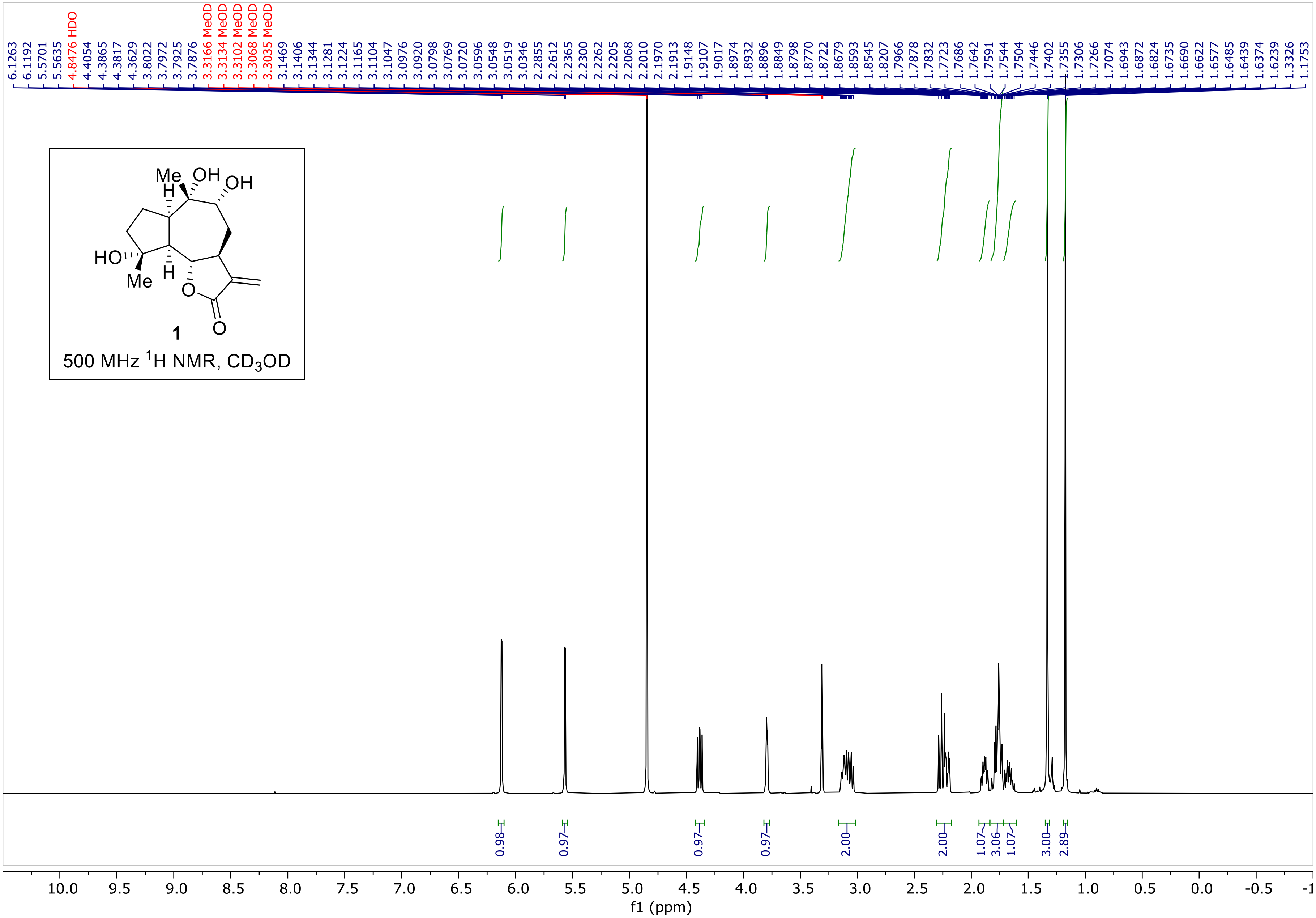
Me
OH
OH
H
HO
Me
H
O
O
1
500 MHz ¹H NMR, CD3OD
f1 (ppm)

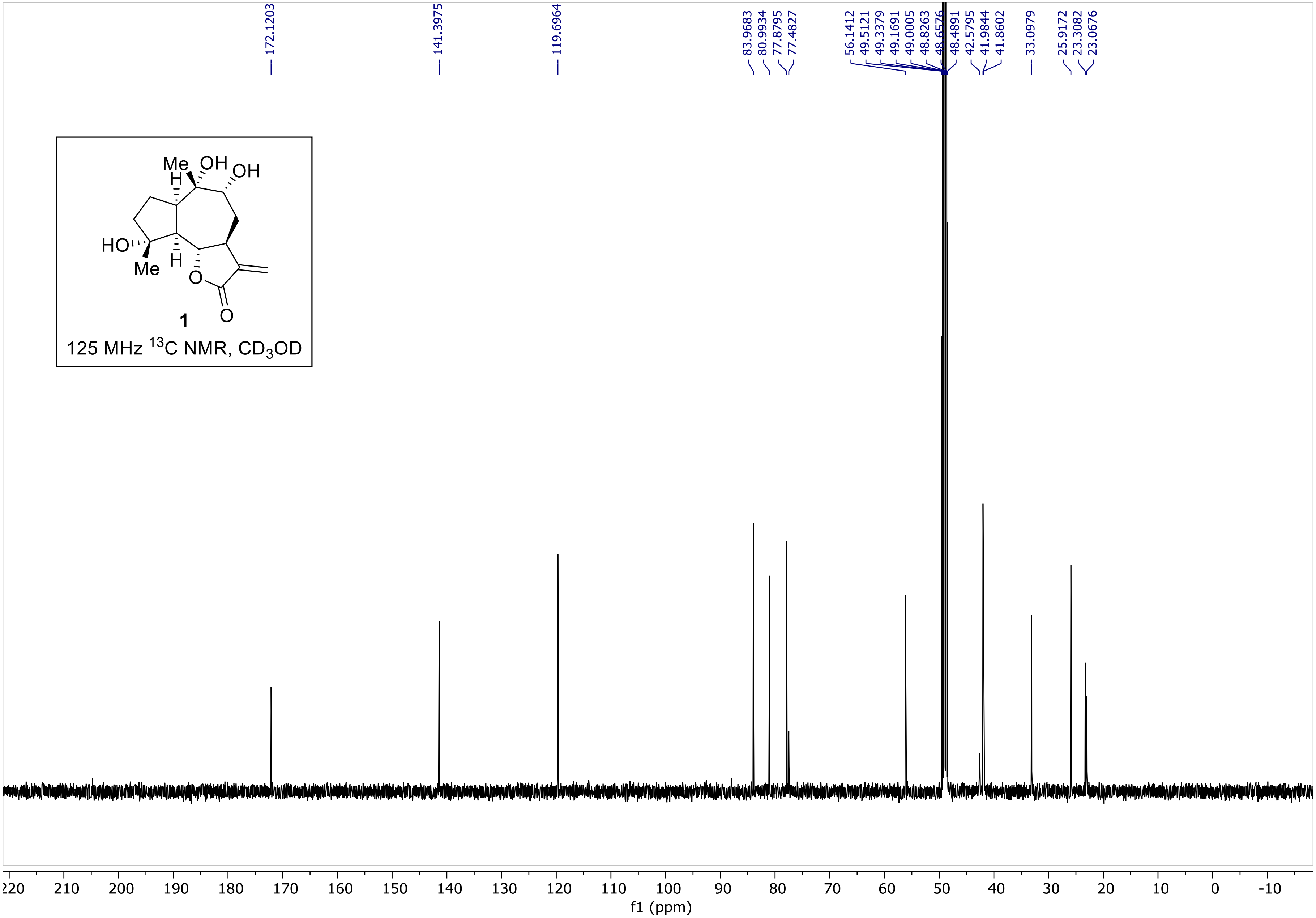
172.1203
141.3975
119.6964
83.9683
80.9934
77.8795
77.4827
56.1412
49.5121
49.3379
49.1691
49.0005
48.8263
48.6576
48.4891
42.5795
41.9844
41.8602
33.0979
25.9172
23.3082
23.0676
1
125 MHz ¹³C NMR, CD₃OD
f1 (ppm)

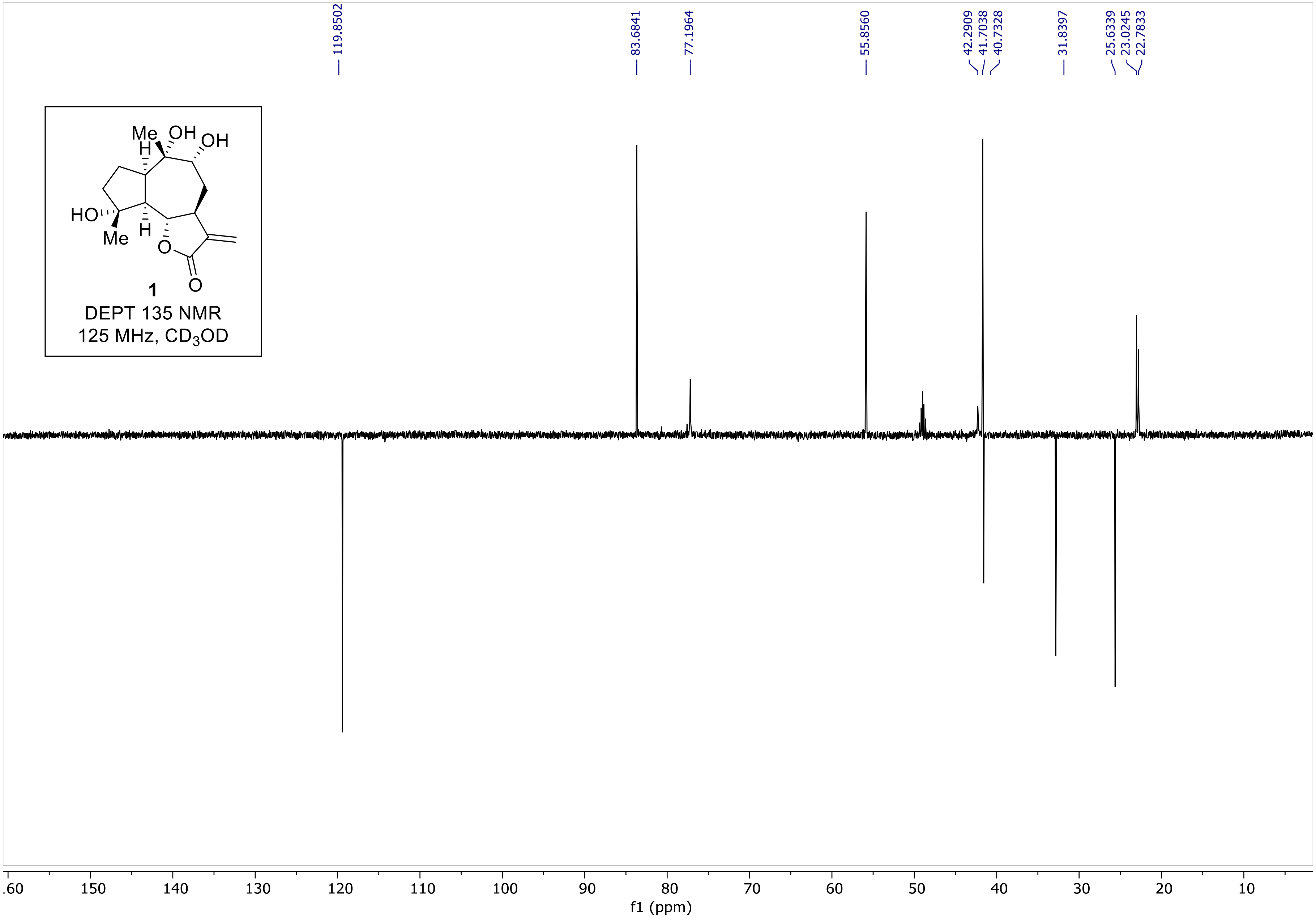

119.8502
83.6841
77.1964
55.8560
42.2909
41.7038
40.7328
31.8397
25.6339
23.0245
22.7833
1
DEPT 135 NMR
125 MHz, CD_3OD
f1 (ppm)

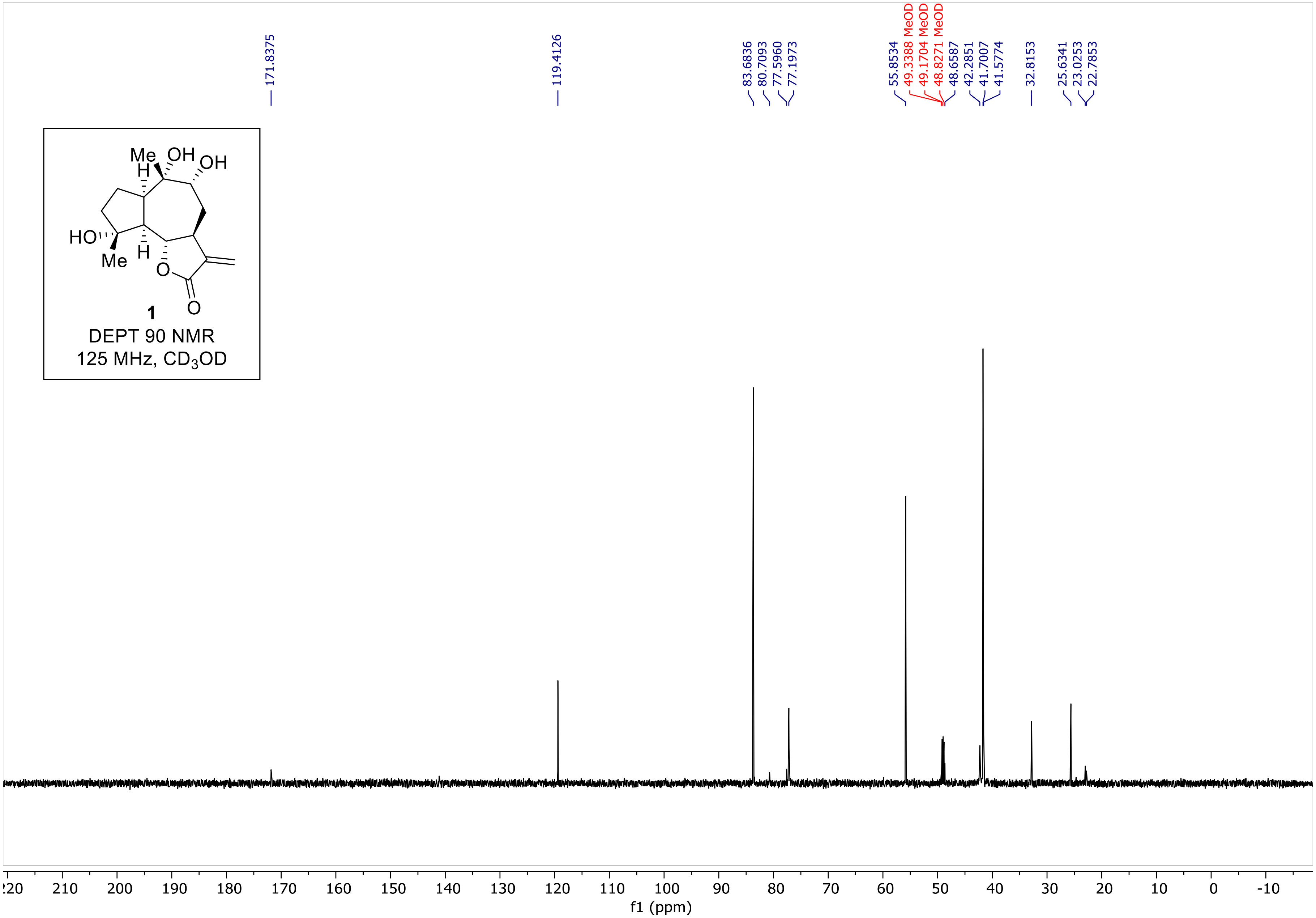

171.8375
119.4126
83.6836
80.7093
77.5960
77.1973
55.8534
49.3388 MeOD
49.1704 MeOD
48.8271 MeOD
48.6587
42.2851
41.7007
41.5774
32.8153
25.6341
23.0253
22.7853
Me
OH
OH
H
HO
Me
H
O
O
1
DEPT 90 NMR
125 MHz, CD_3OD
220
210
200
190
180
170
160
150
140
130
120
110
100
90
80
70
60
50
40
30
20
10
0
-10
f1 (ppm)

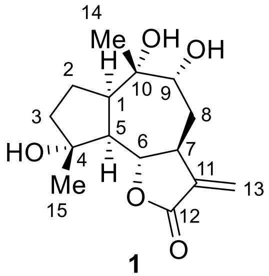

1

500 MHz ^{1}H-^{1}H COSY NMR

CD_3OD

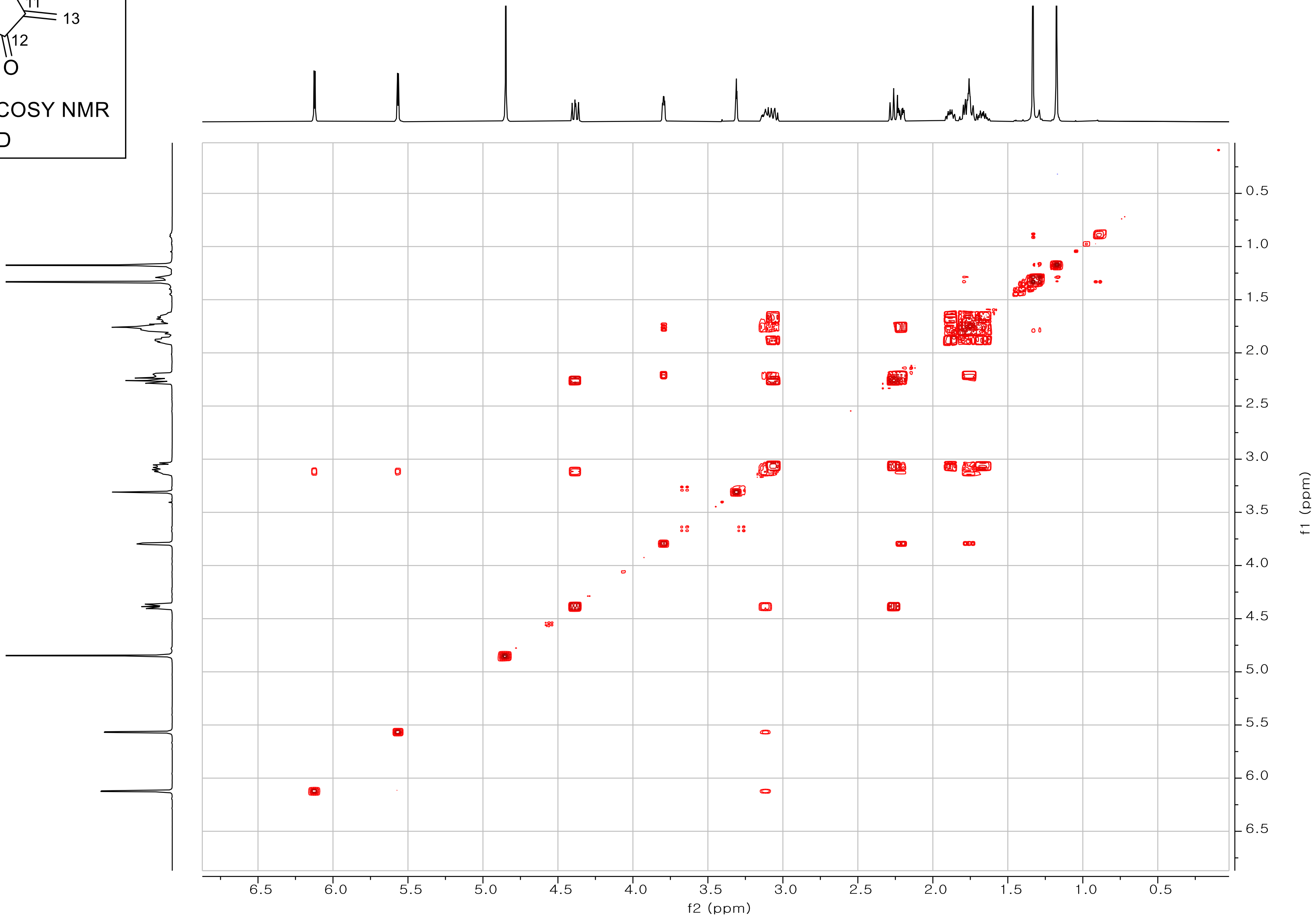

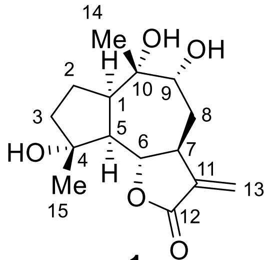

1

500 MHz ^{1}H-^{1}H NOESY NMR

CD_3OD

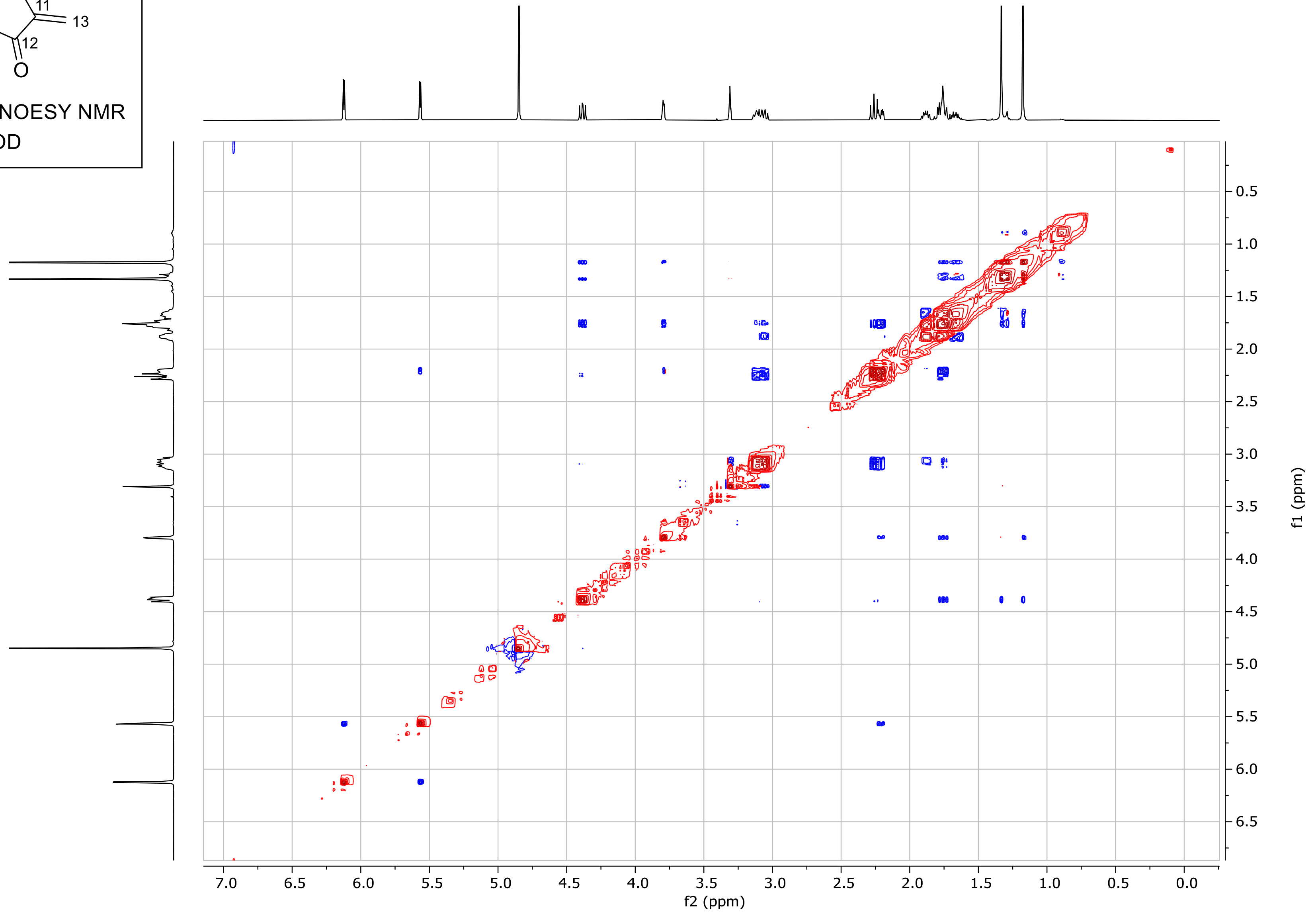

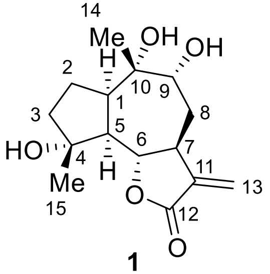

1

500 MHz ^{1}H-^{13}C HSQC NMR

CD_3OD

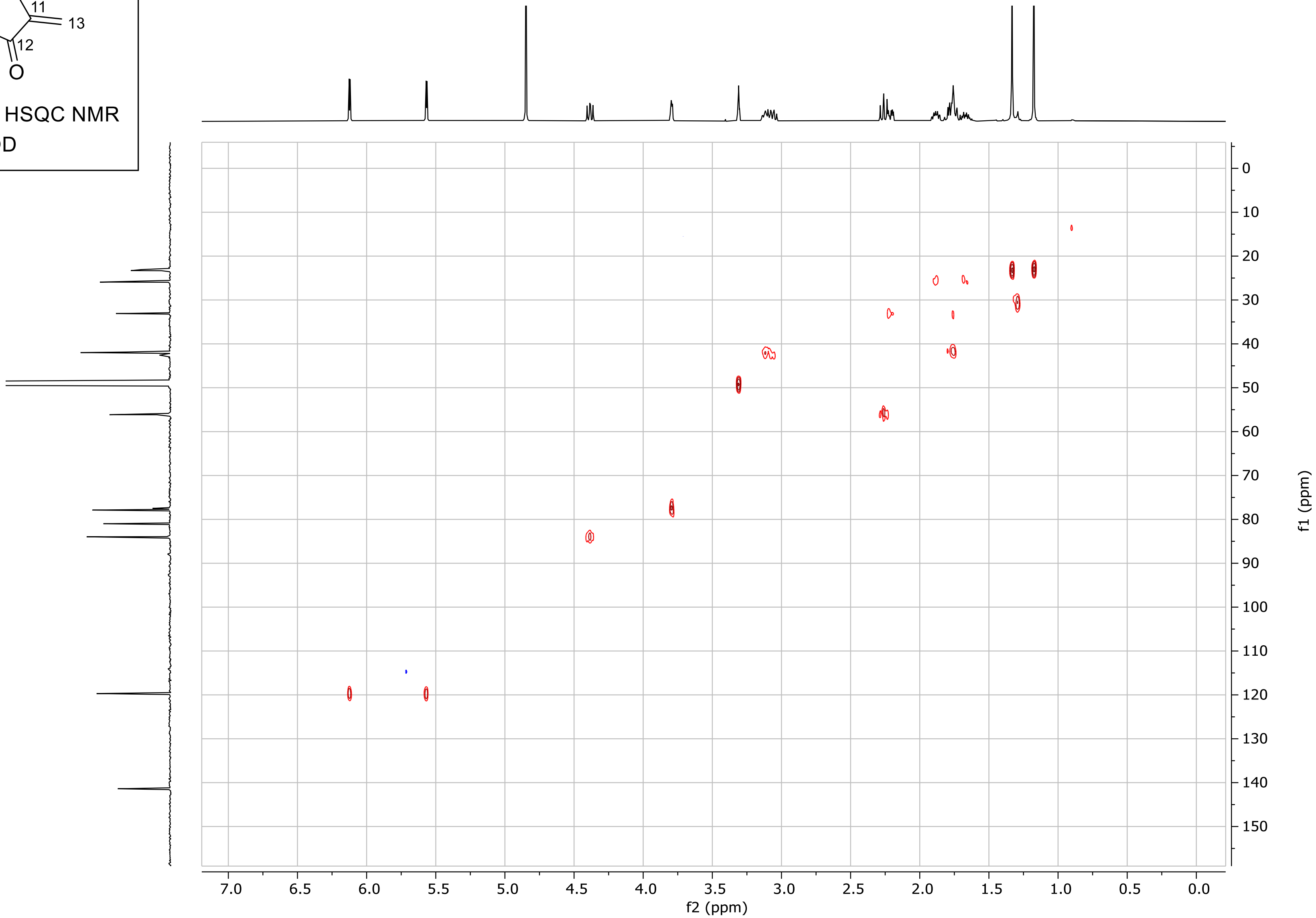

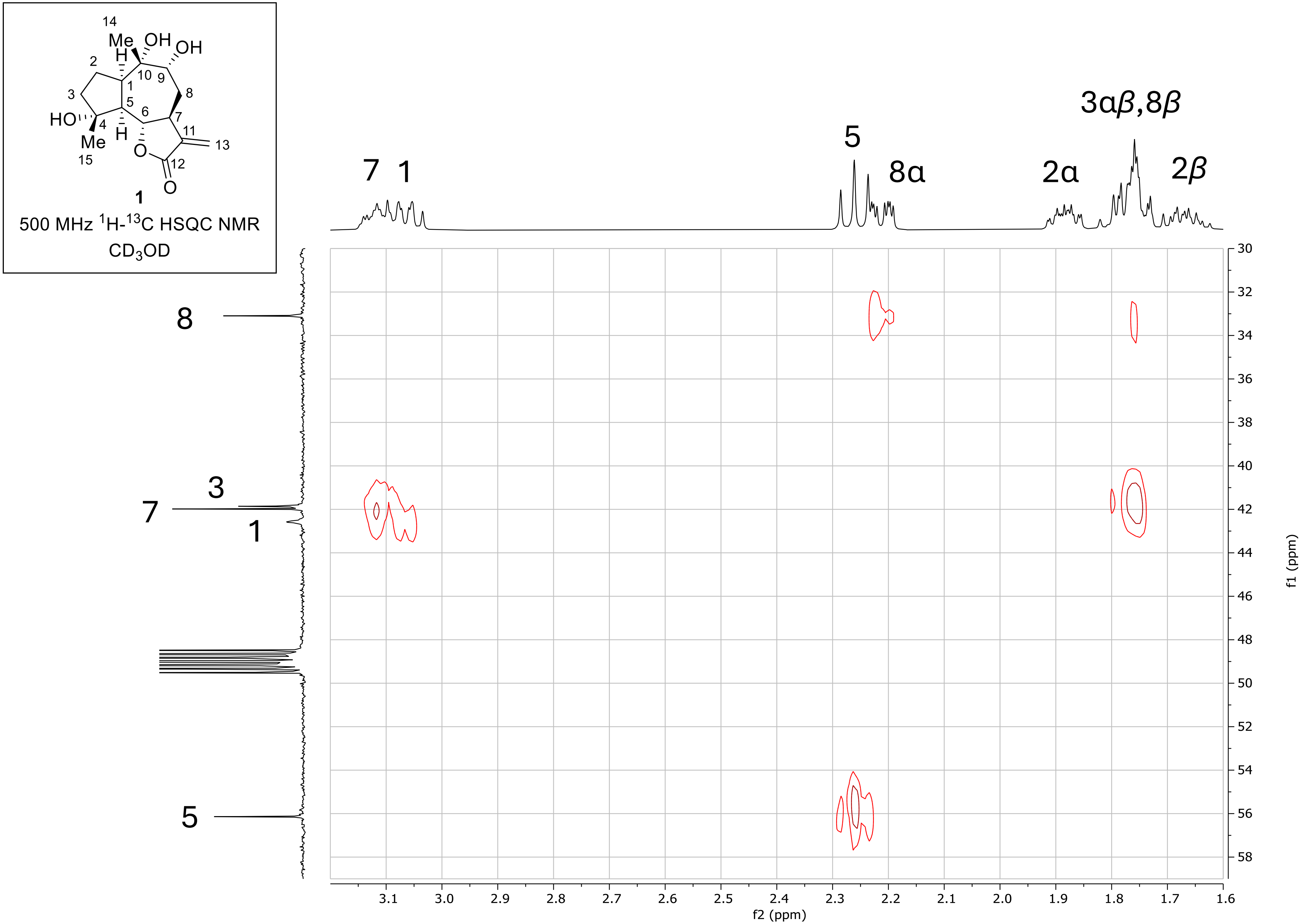
14
Me
OH
OH
H
2
10
9
1
3
8
5
6
7
HO
4
11
13
H
Me
O
15
12
O
1
500 MHz ¹H-¹³C HSQC NMR
CD₃OD
7
1
5
8α
2α
3αβ,8β
2β
8
3
7
1
5
f2 (ppm)
f1 (ppm)

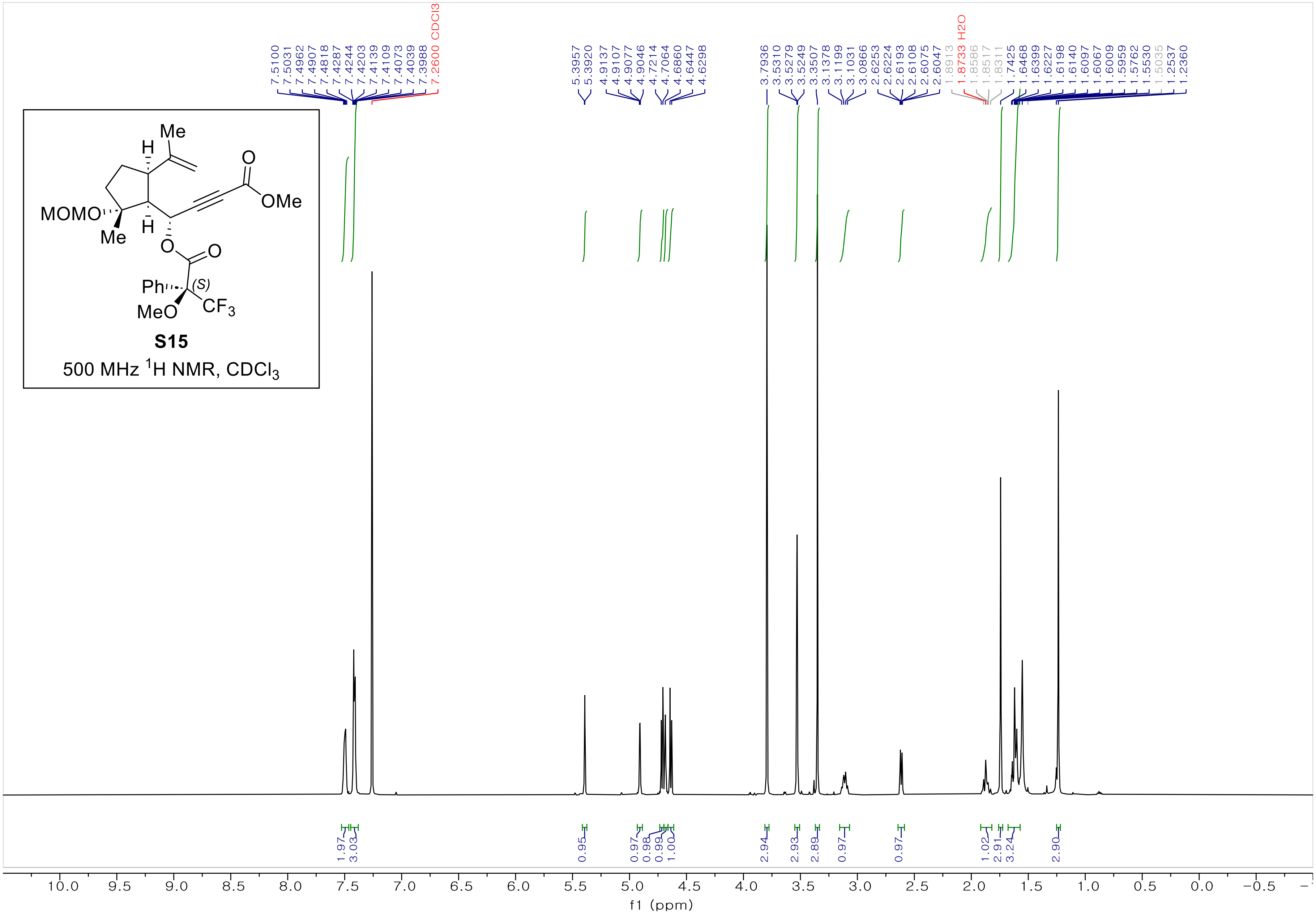
S15
500 MHz ¹H NMR, CDCl₃
f1 (ppm)

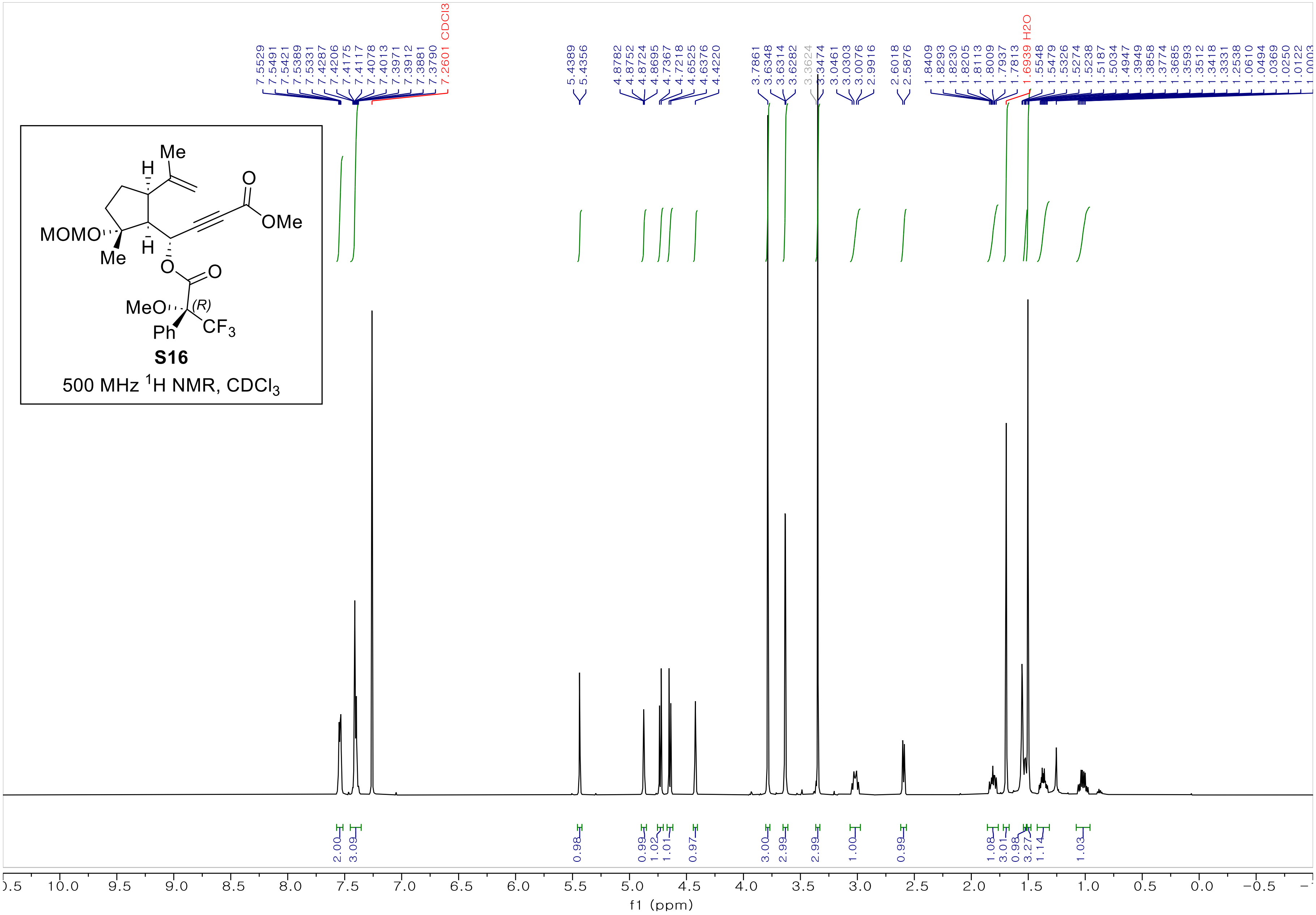

MOMO
Me
H
Me
O
OMe
O
O
MeO
(R)
Ph
CF3
S16
500 MHz 1H NMR, CDCl3
f1 (ppm)
