# Supplementary material for: Asymmetric Total Synthesis of 4,9,10-Trihydroxyguaia-11(13)en-12,6-olide and Discovery of Its Anticancer Activity against Atypical Teratoid Rhabdoid Tumor
Source: ACS Cent Sci. 2025 Jun 3;11(7):1103–10. doi: 10.1021/acscentsci.5c00332 (PMC12291133; doi:10.1021/acscentsci.5c00332)
Supplement: Supplementary file 3 [file oc5c00332_si_003.pdf]

–Supporting Information: Part C–

Asymmetric Total Synthesis of 4,9,10-Trihydroxyguaia-11(13)en-12,6-olide and Discovery of Its Anticancer Activity Against Atypical Teratoid Rhabdoid Tumor

Hyejin Lee[1], Hongjun Jang[1,2], Hwan Myung[3,4], Angela Rivera[5], Anna F. Averette[5], Joseph Heitman[5,6], Jiyong Park[4,3], Deukjoon Kim[7], Hyoungsu Kim[2], and Jiyong Hong[1,6,*]

[1] Department of Chemistry, Duke University, Durham, North Carolina 27708, United States

[2] College of Pharmacy and Research Institute of Pharmaceutical Science and Technology (RIPST), Ajou University, Suwon 16499, Republic of Korea

[3] Department of Chemistry, Korea Advanced Institute of Science and Technology (KAIST), Daejeon 34141, Republic of Korea

[4] Center for Catalytic Hydrocarbon Functionalizations, Institute for Basic Science (IBS), Daejeon 34141, Republic of Korea

[5] Department of Molecular Genetics and Microbiology, Duke University School of Medicine, Durham, North Carolina 27710, United States

[6] Department of Pharmacology and Cancer Biology, Duke University School of Medicine, Durham, North Carolina 27710, United States

[7] College of Pharmacy, Seoul National University, Seoul 08826, Republic of Korea

Computational Details

Important intermediates and transition state (TS) structures were optimized based on density functional theory (DFT) calculations using the ORCA v5.0.3 quantum chemistry software package.[1] Specifically, we utilized Becke's three-parameter hybrid functional (B3LYP)[2] augmented with Grimme's dispersion correction and Becke-Johnson damping function (D3BJ).[3,4] A double zeta quality atomic basis set developed by Alrich's group (def2-SVP) was used for the geometry optimizations.[4,5] With the optimized intermediates and TS structures, we computed vibrational frequencies and thermodynamic values at room temperature (RT) using the same level of the theory for the geometry optimizations. Here, we verified that the intermediates have positive-definite frequencies. Also, we confirmed TS structures bear a single imaginary frequency whose vibrational motion corresponds to the creation and breaking of the chemical bond of interest. Lastly, using the optimized geometries, accurate electronic energies were reevaluated using a hybrid density functional (M06) in combination with a triple-zeta quality basis-set (def2-TZVP),[6,7] while incorporating the solvation energy correction using the conductor-like polarizable continuum solvation model (CPCM) implemented in ORCA.[8]

Gibbs free energy of the identified intermediates and TS structures were computed as follows.

$$G = E_{SCF} + ZPE + U + RT - TS,$$

Here E_{SCF} is the self-consistent electronic energy computed at the M06/def2-TZVP/CPCM(MeOH) level of the theory, ZPE is the zero-point energy, U is the internal energy correction, RT is the thermal energy at the temperature of interest (298 K), and TS is the entropic contribution.

The internal energy correction (U) comprises the thermal vibrational correction (U_{vib}), the thermal rotational correction (U_{rot}), and the thermal translational correction (U_{trans}):

$$U = U_{vib} + U_{rot} + U_{trans}.$$

The entropy (S) is the summation of the vibrational (S_{vib}), the rotational (S_{rot}), and the translational contributions (S_{trans}):

$$S = S_{vib} + S_{rot} + S_{trans}.$$

We utilized CREST software that was developed by Grimme and his coworkers to identify the lowest-energy conformers of intermediate and TS structures.[9] We identified conformers within 8.0 kcal/mol using the software relative to the lowest-energy one. The initially identified conformers were subjected to geometry optimizations, followed by evaluations of Gibbs free energies following the aforementioned computational protocol. For the conformer search of the TS structures, the bond lengths of breaking and emerging bonds were fixed to the value of the pre-identified TS structures.

When considering Gibbs free energy of a molecular complex bearing solvent molecule, the thermodynamic driving force that promotes the solute-solvent complexation (Le Chatelier's principle) was considered.[10] In the tandem hydroallylation/cyclization reaction, the substrate 0.027 mmol of **3** was dissolved in 0.3 mL of MeOH, where the solvent molecule is 275 times higher in molar concentration compared to the solute. This excess corresponds to 3.32 kcal/mol in favor of the formation of solute-solvent complexes at 25 °C.

Description of Computational Results

As summarized in Schemes S1 and S2, we identified important intermediate and transition state structures using density functional theory (DFT) calculations. Previously, the Yamamoto group delineated the mechanism of Cu-catalyzed hydroarylation of alkynes using phenylboronic acid.[11,12] The authors demonstrated that *in-situ* prepared Cu(I)OAc species is the reactive catalyst that can engage alkyne and allyl/phenyl substrates. Moreover, the authors showed η^2-alkyne complexes can undergo carbocupration with the aryl substrates which is the regio-determining step in the hydroarylation of alkynes. Inspired by the previous mechanistic study, we hypothesized that the carbocuprations initiated from η^2-alkyne complexes (**C** and **D**) are the regio-determining steps in the tandem hydroallylation/cyclization reaction of **3** and **25**. We note that the η^2-alkyne complexes bear a solvent molecule (MeOH) that coordinates the metal center.

For the MOM-protected alcohol substrate **3**, we identified two regio-divergent TS structures (Scheme S1). **TS-3-β** leads to the β-allylated product **24'a,** which has an activation energy of 13.7 kcal/mol. **TS-3-α** engenders the α-allylated product **24'b,** which has an activation energy of 13.6 kcal/mol. Here, the activation energies were calculated relative to the preceding intermediate **C**.

The energy difference of the two TSs correlates to the product ratio **24'a**:**24'b** = 1:1.1, which was in good agreement with the experimental ratio of **24a**:**24b** = 1.3:1. We note that in **TS-3-α** the MOM-protected oxygen atom coordinates the metal center, whereas in **TS-3-β** a solvent molecule coordinates the copper-center. For the epoxide substrate **25**, we found the TS of β-allylation **TS-25-β** is lower in energy by 1.6 kcal/mol than that of the α-allylation **TS-25-α** (Scheme S2). The computational finding was in excellent agreement with the experimental observation: **26a**:**26b** = 9.4:1 in the experiment whereas **26'a**:**26'b** = 15:1 in computation. The geometrical constriction imposed on epoxide substrate **25** explained the higher regioselectivity relative to MOM-protected substrate **3**. At the TS, the MOM-protected oxygen atom coordinates the copper center resulting in a six-membered metallacycle (**TS-3-α**). On the contrary, in **TS-25-α** the epoxide oxygen cannot coordinate the metal center but the oxygen of the unprotected hydroxyl group can. Here, the resultant four-membered metallacycle elevates intramolecular strain causing the α-allylation more challenging than the β-allylation.

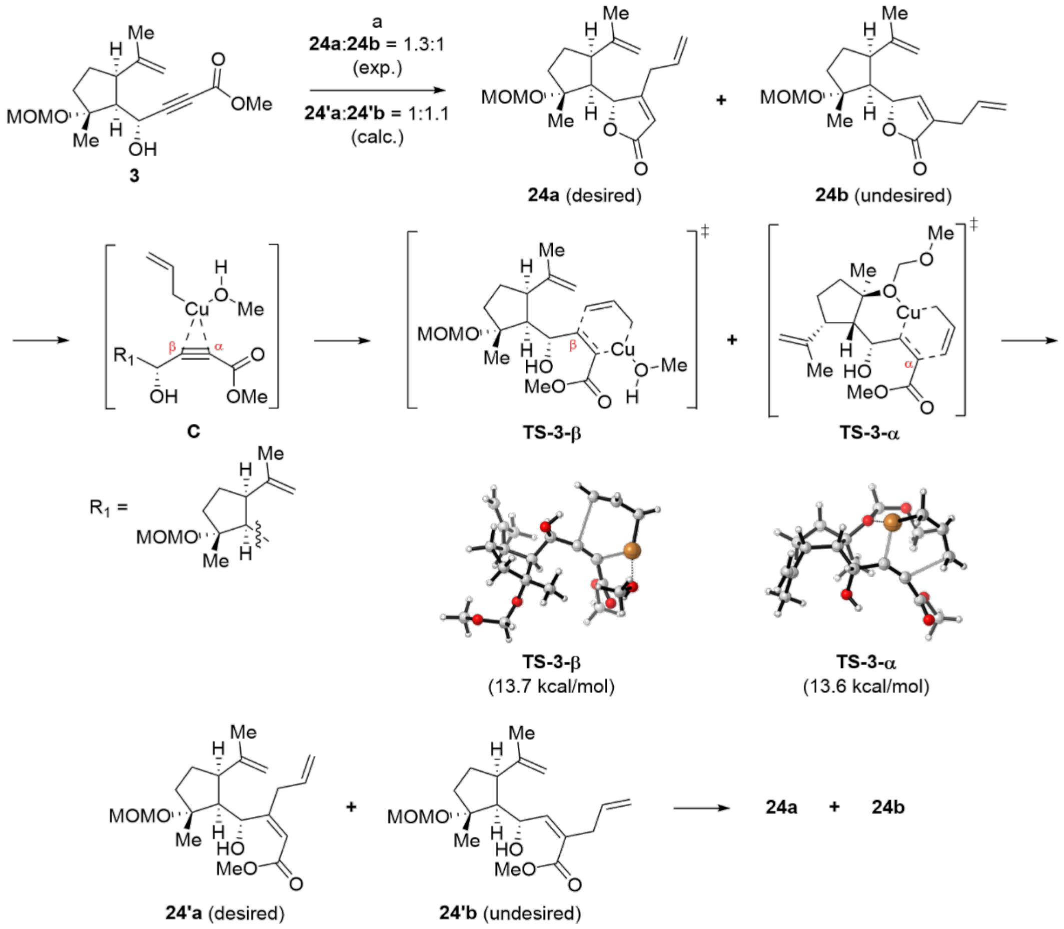

Scheme S1. Allylation of MOM-protected substrate **3**.

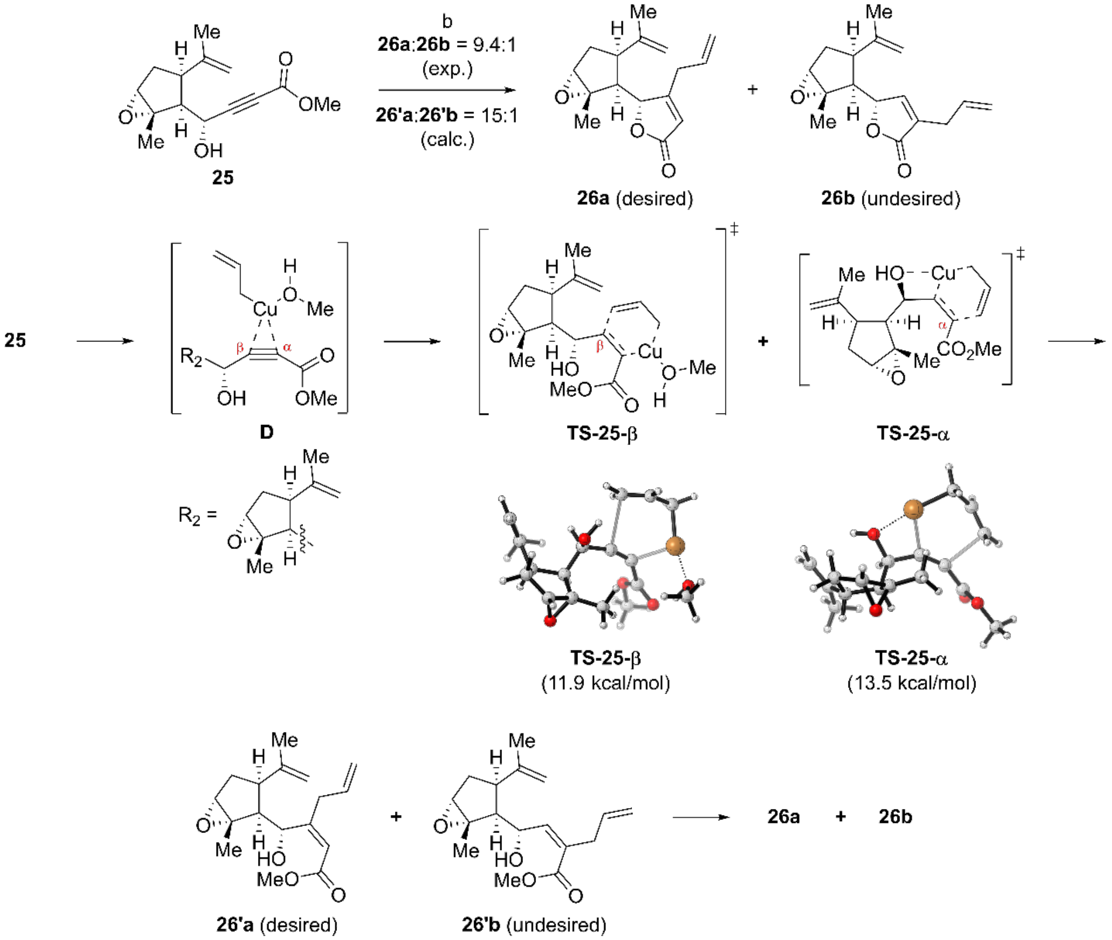

Scheme S2. Allylation of epoxide substrate **25**.

Table S5. Computed electronic energies, internal energies and entropic contributions.

Molecule	E(SCF) [eV]	ZPE [kcal/mol]	U [kcal/mol]	-TS [kcal/mol]	Conc. Correction [kcal/mol]
**MeOH**	-3148.533	31.98	2.08	-16.94	0.0
**C**	-78189.156	317.56	20.65	-58.35	-3.32
**TS-3-β**	-78188.524	316.76	20.60	-58.38	-3.32
**TS-3-α**	-75039.556	282.95	17.91	-52.41	0.0
**D**	-73970.679	263.50	18.12	-53.55	-3.32
**TS-25-β**	-73970.193	263.64	17.71	-52.56	-3.32
**TS-25-α**	-70821.133	229.78	15.05	-47.04	0.0

Coordinates of DFT optimized geometries

================================
MeOH
================================

H	0.7585	-1.0976	0.2785
C	0.3395	-0.1027	0.0603
H	0.6233	0.5671	0.8979
H	0.8382	0.2725	-0.8570
O	-1.0524	-0.2503	-0.0853
H	-1.4276	0.6163	-0.2857

================================
C
================================

C	-1.2265	-1.3258	1.3451
C	-2.4986	-1.4433	2.2195
C	-3.5774	-1.8646	1.2206
H	-2.7414	-0.4545	2.6421
H	-2.3818	-2.1478	3.0567
C	-3.3007	-1.0554	-0.0671
C	-1.7921	-0.6241	0.0824
C	-3.6564	-1.8443	-1.3284
H	-1.8317	0.4346	0.3687
C	0.4995	-0.4133	-0.8583
C	-0.5169	-2.6642	1.2179
H	-0.5077	-0.6399	1.8194
C	1.6931	-0.7377	-0.6448
C	2.9232	-1.4727	-0.4388
O	3.3384	-1.8248	0.6481
O	3.5798	-1.6863	-1.5886
C	4.8342	-2.3600	-1.4770
H	5.5236	-1.7952	-0.8310
H	5.2326	-2.4273	-2.4966
H	4.7007	-3.3656	-1.0499
C	0.5254	-2.8971	2.2812
H	1.3901	-2.2324	2.1164
H	0.8938	-3.9333	2.2758
H	0.1237	-2.6752	3.2851
C	-0.7791	-3.5969	0.2919
H	-1.5146	-3.4543	-0.4989
H	-0.2477	-4.5530	0.2942
Cu	1.4002	1.2066	-0.2064
C	1.9317	4.5037	0.8594
C	1.1890	3.9453	-0.1374
H	2.6292	5.3208	0.6583
H	1.7488	4.2502	1.9110
C	0.2379	2.8493	-0.0379
H	-0.2436	2.7575	0.9492
H	-0.5041	2.8330	-0.8447
H	1.3946	4.3009	-1.1583
H	-4.7393	-2.0430	-1.3352
H	-3.1400	-2.8131	-1.3412
H	-3.3782	-1.3126	-2.2473
O	-4.1706	0.0998	0.0449
H	-3.4794	-2.9390	1.0058
H	2.9169	1.6469	2.6147
C	3.6230	1.3750	1.8101
H	4.6201	1.7749	2.0610
H	3.6824	0.2805	1.7400
O	3.1905	1.8712	0.5456
H	3.0136	2.8475	0.6087
C	-0.9361	-0.6562	-1.1919
H	-0.9544	-1.6599	-1.6446
O	-1.4210	0.2296	-2.1715
H	-1.7276	1.0455	-1.7325
H	-4.6034	-1.6774	1.5668
C	-4.2105	1.0409	-0.9774
O	-3.1821	2.0068	-0.9108
C	-3.2973	2.8956	0.1861
H	-4.1176	0.5888	-1.9750
H	-5.1894	1.5460	-0.8836
H	-3.2214	2.3611	1.1489
H	-4.2670	3.4270	0.1557
H	-2.4794	3.6225	0.1119

================================
TS-3-β
================================

C	-3.2255	-0.4243	-0.3813
C	-3.6165	-0.9765	1.0005
C	-2.4201	-1.8463	1.4524
H	-4.5603	-1.5403	0.9513
H	-3.7543	-0.1510	1.7109
C	-1.3140	-1.6825	0.3794
C	-1.6719	-0.3527	-0.3311
C	0.0955	-1.7820	0.9544
H	-1.2467	-0.3805	-1.3441
C	0.2442	1.1935	-0.0194
C	-3.9044	0.8290	-0.8805
H	-3.4331	-1.2107	-1.1269
C	1.3774	1.0838	-0.5265
C	2.0249	0.2634	-1.5298
O	2.9002	-0.5744	-1.3192
O	1.5388	0.4726	-2.7549
C	2.0774	-0.3386	-3.8040
H	1.8495	-1.4001	-3.6257
H	3.1686	-0.2168	-3.8698
H	1.5947	0.0066	-4.7261
C	-3.5788	1.2003	-2.3068
H	-2.5029	1.4133	-2.4333
H	-4.1410	2.0852	-2.6391
H	-3.8101	0.3636	-2.9892
C	-4.7429	1.5670	-0.1398
H	-4.9902	1.3072	0.8912
H	-5.2173	2.4630	-0.5511
Cu	3.0527	1.8273	0.8116
C	0.0807	3.5883	1.1827
C	1.3521	3.6608	1.6860
H	-0.7886	3.6125	1.8471
H	-0.1122	3.7764	0.1239
C	2.5691	3.7334	0.9240
H	2.4660	4.1209	-0.0989
H	3.4238	4.1766	1.4573
H	1.4629	3.5861	2.7779
H	0.2243	-2.7340	1.4940
H	0.2681	-0.9670	1.6683
H	0.8520	-1.7314	0.1588
O	-1.4492	-2.6431	-0.7044
H	-2.0299	-1.4838	2.4120
H	2.5123	-1.0316	2.1847
C	3.5908	-0.8136	2.1141

H 3.9082 -0.2931 3.0289
H 4.1452 -1.7648 2.0442
O 3.8925 0.0209 1.0038
H 3.6084 -0.4102 0.1508
C -1.1605 0.9469 0.3327
H -1.7470 1.7748 -0.1091
O -1.3665 0.9096 1.7339
H -0.9074 1.6952 2.0745
H -2.6914 -2.9021 1.5686
C -1.4896 -4.0050 -0.4126
O -2.7690 -4.4969 -0.1257
C -3.6788 -4.4214 -1.1986
H -0.8724 -4.2640 0.4636
H -1.0834 -4.5128 -1.3101
H -3.2849 -4.9323 -2.1004
H -3.9162 -3.3780 -1.4713
H -4.6036 -4.9255 -0.8832

===============================
TS-3-α
===============================
C -3.4378 0.2451 -0.2486
C -3.8504 -0.7111 0.8921
C -2.7926 -1.8162 0.8526
H -4.8757 -1.0910 0.7681
H -3.8119 -0.1984 1.8644
C -1.4798 -1.0407 0.6536
C -1.8774 0.0415 -0.3878
C -0.9227 -0.5049 1.9712
H -1.7288 -0.4545 -1.3600
C 0.4666 0.9398 -0.6710
C -3.9517 1.6732 -0.1595
H -3.8606 -0.1659 -1.1779
C 1.5742 1.3001 -0.1939
C 2.4583 1.9414 0.7382
O 2.9367 3.0515 0.6414
O 2.7047 1.1270 1.8073
C 3.5892 1.6550 2.7901
H 3.6717 0.8885 3.5718
H 3.1968 2.5914 3.2164
H 4.5799 1.8645 2.3568
C -4.1767 2.2869 1.1966
H -3.2740 2.1993 1.8179
H -5.0055 1.7861 1.7267
H -4.4311 3.3532 1.1080
C -4.2080 2.3521 -1.2874
H -4.0756 1.8953 -2.2734
H -4.5550 3.3892 -1.2654
Cu 0.6791 -0.9802 -1.4789
C 3.5330 0.6483 -1.5092
C 3.0977 -0.5432 -2.0457
H 4.3534 0.6675 -0.7881
H 3.2762 1.6012 -1.9754
C 1.9988 -0.6782 -2.9474
H 1.7154 0.2055 -3.5305
H 1.9288 -1.6217 -3.5080
H 3.5324 -1.4718 -1.6508
H 0.0019 0.0620 1.8163
H -0.7297 -1.3287 2.6756
H -1.6454 0.1743 2.4391
O -0.4744 -1.8563 -0.0227
H -2.7854 -2.4526 1.7514
C -0.9865 1.2835 -0.4701
H -1.3152 1.8393 -1.3707
O -1.1809 2.0872 0.6818
H -0.5442 2.8135 0.6374
H -2.9514 -2.4640 -0.0256
C 0.0408 -3.0473 0.5764
O 1.4033 -3.1049 0.3966
C 2.1667 -2.2390 1.2305
H -0.2443 -3.0681 1.6441
H -0.3897 -3.9160 0.0541
H 1.9602 -2.4415 2.2978
H 1.9701 -1.1772 1.0213
H 3.2241 -2.4468 1.0220

===============================
D
===============================
C 3.3917 -0.1718 1.3534
C 3.9887 1.0923 0.7028
C 3.5966 0.9441 -0.7499
H 5.0777 1.1600 0.8545
H 3.5206 2.0086 1.0926
C 2.4490 0.0296 -0.8700
C 2.0887 -0.4614 0.5351
C 1.4413 0.0688 -1.9878
H 1.8580 -1.5385 0.5320
C -0.3850 -0.3180 0.5747
C 3.2093 -0.1930 2.8515
H 4.0673 -1.0041 1.1001
C -1.1804 -1.2474 0.2696
C -1.5328 -2.6683 0.2917
O -0.7851 -3.5176 0.7192
O -2.7483 -2.9120 -0.2106
C -3.1537 -4.2815 -0.2246
H -2.4606 -4.8858 -0.8297
H -4.1583 -4.2957 -0.6636
H -3.1733 -4.6922 0.7964
C 2.8614 -1.5449 3.4251
H 1.9228 -1.9402 2.9981
H 2.7493 -1.5094 4.5186
H 3.6440 -2.2836 3.1790
C 3.3466 0.8884 3.6307
H 3.5976 1.8698 3.2237
H 3.2126 0.8220 4.7145
Cu -1.8578 0.3821 -0.5717
C -3.5216 0.3079 -1.7109
C -3.1892 1.2265 -2.7815
H -4.3416 0.6447 -1.0556
H -3.6597 -0.7347 -2.0213
C -3.3585 2.5829 -2.8113
H -3.9760 3.0890 -2.0589
H -3.0517 3.1724 -3.6789
H -2.6402 0.7874 -3.6288
H 1.0173 -0.9322 -2.1650
H 1.9309 0.4066 -2.9125
H 0.6050 0.7501 -1.7695
O 3.7668 -0.3848 -1.2592
H 3.7764 1.7521 -1.4694
H -1.6566 4.3139 0.0226
C -1.8171 3.2581 0.2953
H -1.2289 3.0256 1.1925
H -2.8890 3.0971 0.4996

O	-1.3517	2.3988	-0.7496
H	-1.8753	2.5925	-1.5863
C	0.8538	0.2812	1.1121
H	0.8439	0.1060	2.2012
O	0.9262	1.6859	0.9507
H	0.5115	1.9216	0.1055

================================
TS-25-β
================================

C	-3.6644	0.4785	0.2735
C	-3.8682	1.0428	1.6928
C	-3.0197	0.1314	2.5487
H	-4.9307	1.0573	1.9835
H	-3.4633	2.0620	1.7846
C	-1.9933	-0.5191	1.7245
C	-2.1960	-0.0733	0.2731
C	-0.6503	-0.9528	2.2424
H	-2.1055	-0.9295	-0.4141
C	0.1112	0.3652	-0.5564
C	-4.0080	1.3593	-0.9032
H	-4.2979	-0.4199	0.1992
C	0.9866	-0.4910	-0.7905
C	1.1073	-1.9258	-0.9408
O	1.6967	-2.6841	-0.1724
O	0.4695	-2.3827	-2.0205
C	0.5179	-3.7962	-2.2427
H	1.5589	-4.1402	-2.3316
H	-0.0259	-3.9700	-3.1789
H	0.0374	-4.3362	-1.4133
C	-4.0389	0.6513	-2.2355
H	-3.0603	0.1988	-2.4739
H	-4.3062	1.3325	-3.0564
H	-4.7681	-0.1776	-2.2189
C	-4.2721	2.6686	-0.7974
H	-4.2511	3.1896	0.1616
H	-4.5230	3.2657	-1.6791
Cu	3.0996	0.1869	-0.3102
C	0.9810	2.7183	-1.3880
C	2.3003	2.6080	-1.0352
H	0.3501	3.5052	-0.9633
H	0.6006	2.2231	-2.2845
C	3.2533	1.6837	-1.5836
H	3.0226	1.3020	-2.5877
H	4.3067	1.9852	-1.4795
H	2.6497	3.2336	-0.2009
H	-0.7436	-1.2647	3.2930
H	0.0626	-0.1192	2.1732
H	-0.2472	-1.7993	1.6646
O	-3.1265	-1.2594	2.2104
H	-2.8588	0.3479	3.6114
H	2.1799	-0.5895	2.7108
C	3.2110	-0.9103	2.4871
H	3.9017	-0.1005	2.7625
H	3.4515	-1.7962	3.0989
O	3.3924	-1.1945	1.1068
H	2.7736	-1.9147	0.8041
C	-1.1632	0.9887	-0.1721
H	-1.5660	1.4799	-1.0770
O	-1.0094	1.9395	0.8670
H	-0.2686	2.5025	0.5902

================================
TS-25-α
================================

C	-3.5653	1.0004	0.6059
C	-3.7563	0.0534	1.8097
C	-3.1429	-1.2417	1.3350
H	-4.8173	-0.0413	2.0904
H	-3.1989	0.3881	2.6977
C	-2.2085	-0.9845	0.2284
C	-2.2441	0.5135	-0.0990
C	-1.0279	-1.8601	-0.0923
H	-2.3140	0.6510	-1.1904
C	0.2748	0.8970	-0.2468
C	-3.6222	2.4881	0.8653
H	-4.3709	0.7675	-0.1071
C	0.8912	0.7021	-1.3236
C	0.9277	0.6852	-2.7724
O	1.0930	1.6445	-3.4915
O	0.7567	-0.5746	-3.2472
C	0.7985	-0.7116	-4.6654
H	0.6643	-1.7805	-4.8737
H	1.7632	-0.3620	-5.0650
H	-0.0033	-0.1268	-5.1428
C	-3.7409	3.3450	-0.3701
H	-4.6987	3.1394	-0.8790
H	-2.9489	3.1193	-1.1035
H	-3.6991	4.4180	-0.1343
C	-3.5891	3.0334	2.0932
H	-3.5702	2.4262	3.0018
H	-3.6349	4.1176	2.2294
Cu	1.1878	0.7026	1.6649
C	3.0169	-0.1216	-1.1118
C	3.4240	0.4477	0.0752
H	3.4192	0.2474	-2.0575
H	2.5908	-1.1284	-1.1280
C	3.0247	0.0078	1.3699
H	2.8166	-1.0695	1.4697
H	3.5944	0.4124	2.2198
H	3.9616	1.4030	0.0166
H	-1.2874	-2.9060	0.1277
H	-0.1482	-1.5713	0.5002
H	-0.7459	-1.7751	-1.1524
O	-3.4985	-1.5813	-0.0073
H	-3.0129	-2.0861	2.0225
C	-1.0105	1.3055	0.3581
H	-1.1687	2.3696	0.1192
O	-0.8490	1.2119	1.8129
H	-1.3886	1.9045	2.2301

Computed frequencies of DFT optimized geometries

==============================
MeOH
==============================
330.75 1069.87 1107.04 1170.85 1363.73 1467.50
1473.64 1492.95 2959.82 3010.25 3103.79 3819.06

==============================
C
==============================
11.31 18.20 36.82 43.48 52.36 56.60
64.76 72.22 73.18 88.82 98.66 105.06
110.20 122.55 130.50 134.46 141.63 152.81
156.90 160.08 165.04 175.14 181.45 184.46
186.76 209.30 217.23 233.51 244.24 254.71
260.18 275.40 292.30 303.10 307.29 313.81
348.07 349.93 364.26 388.61 416.37 424.44
434.68 439.34 462.90 469.47 477.07 518.96
522.55 569.22 600.80 609.89 630.69 640.19
667.60 672.05 713.05 718.95 719.67 756.30
766.11 796.55 822.58 841.49 853.74 867.09
886.03 906.47 921.88 934.07 937.81 943.30
955.55 972.34 976.88 1008.09 1012.43 1020.89
1027.71 1047.95 1051.40 1060.30 1065.57 1067.11
1077.41 1109.24 1118.20 1136.88 1142.15 1148.02
1168.66 1169.38 1175.97 1181.65 1181.87 1192.86
1204.81 1209.84 1211.00 1222.96 1236.34 1251.71
1256.12 1280.08 1287.84 1297.41 1310.48 1324.84
1337.96 1342.18 1357.41 1378.39 1392.57 1398.80
1407.73 1408.12 1413.32 1424.23 1430.75 1433.71
1449.97 1452.97 1454.84 1456.97 1457.84 1458.90
1464.16 1466.48 1468.11 1471.20 1472.28 1473.35
1475.55 1483.79 1487.14 1490.12 1497.99 1626.20
1723.54 1762.72 2090.01 2996.66 3007.55 3017.86
3019.99 3041.74 3042.00 3050.54 3052.74 3057.76
3068.09 3068.69 3071.69 3086.03 3090.93 3101.47
3101.68 3103.26 3110.25 3120.18 3124.35 3126.81
3128.53 3135.87 3149.88 3151.47 3153.88 3155.90
3162.12 3170.20 3216.02 3252.89 3261.89 3641.15

==============================
TS-3-β
==============================
-75.43 20.54 25.67 34.13 37.02 40.82
56.08 58.54 65.25 73.84 79.73 83.44
91.03 99.67 114.10 120.89 131.49 134.31
137.70 143.55 147.64 153.60 158.65 170.26
178.03 185.09 197.29 210.10 217.96 246.47
273.14 280.93 284.17 301.40 310.40 326.64
342.98 352.94 372.21 386.41 402.24 412.91
414.77 427.58 457.73 469.35 491.54 496.83
519.04 547.85 575.82 595.58 603.17 641.08
667.65 698.38 708.70 733.47 743.12 766.79
778.69 816.98 833.92 854.88 867.54 888.28
907.51 922.37 923.18 930.30 940.78 955.18
956.26 978.24 989.53 998.37 1002.98 1011.19
1028.03 1035.96 1043.54 1058.73 1077.90 1081.00
1086.63 1096.26 1110.15 1128.19 1137.16 1156.67
1168.15 1172.10 1179.09 1181.63 1190.07 1202.31
1206.82 1222.67 1225.57 1232.98 1243.79 1257.56
1280.69 1289.03 1289.32 1294.43 1312.84 1320.07
1328.66 1337.23 1354.17 1374.80 1389.19 1394.61
1397.61 1401.46 1405.40 1424.50 1430.89 1443.45
1445.69 1452.43 1454.78 1455.57 1460.93 1463.28
1463.31 1464.84 1468.74 1469.83 1472.19 1472.73
1474.85 1477.97 1485.93 1490.41 1495.56 1610.14
1707.98 1722.35 2084.48 2976.27 2987.51 2998.05
3013.39 3019.90 3030.78 3041.08 3046.72 3057.75
3060.82 3064.49 3074.42 3077.96 3092.82 3095.85
3100.55 3102.27 3121.69 3121.76 3122.55 3127.62
3128.72 3134.63 3137.55 3140.67 3143.71 3152.95
3160.09 3174.18 3221.61 3236.86 3239.17 3712.92

==============================
TS-3-α
==============================
-109.78 31.91 35.00 49.88 57.32 61.15
66.45 78.76 82.00 90.72 110.05 114.03
128.74 131.61 135.27 145.50 158.25 168.53
170.46 184.62 197.04 203.32 217.10 236.49
240.04 252.65 280.14 285.69 299.95 303.49
326.31 335.12 353.91 360.06 374.64 392.36
399.74 425.19 440.55 453.49 466.39 488.30
524.86 548.17 573.45 593.42 605.79 636.41
674.15 702.49 723.53 725.60 734.90 759.35
773.02 810.26 832.24 850.42 864.25 898.59
914.24 915.21 934.02 947.46 949.09 952.30
957.07 983.03 987.58 992.57 1011.08 1015.98
1033.83 1037.34 1043.70 1059.66 1073.90 1087.53
1096.53 1123.35 1142.12 1155.91 1161.72 1170.14
1191.50 1199.85 1201.85 1213.49 1227.16 1232.09
1238.39 1249.52 1269.73 1274.29 1297.68 1305.50
1308.13 1314.90 1319.03 1321.46 1345.26 1358.76
1375.34 1388.85 1399.03 1406.31 1410.89 1427.29
1428.91 1452.04 1452.20 1453.54 1455.98 1457.41
1460.54 1469.93 1474.05 1475.69 1476.92 1477.46
1482.88 1488.52 1501.03 1585.28 1714.61 1781.59
2021.23 2981.43 3004.81 3016.03 3023.88 3033.05
3034.50 3038.14 3054.05 3056.60 3063.96 3075.57
3094.48 3099.31 3102.65 3112.33 3114.43 3115.32
3118.44 3126.69 3137.38 3144.78 3146.83 3154.79
3158.93 3172.01 3180.43 3219.12 3243.93 3804.99

==============================
D
==============================
15.96 18.65 27.02 34.48 40.40 51.71
61.22 70.28 87.05 92.24 107.12 113.48
131.03 132.72 145.20 157.74 163.33 169.67
176.79 180.79 191.43 207.21 227.52 241.08
247.68 255.30 270.90 285.27 305.55 315.65
329.91 343.96 365.30 375.33 392.62 414.82
418.55 452.77 459.29 472.36 492.26 504.88
549.66 604.43 637.10 643.74 657.98 668.98
691.09 724.19 748.39 757.72 778.89 801.19
827.74 853.87 865.59 887.09 921.72 922.34
928.75 942.29 950.15 955.34 963.82 977.90
1000.17 1004.35 1013.07 1031.18 1032.32 1045.75
1053.11 1059.53 1078.92 1088.20 1091.49 1119.75
1136.59 1143.51 1171.20 1181.39 1191.77 1207.32
1212.32 1225.07 1228.76 1253.61 1265.25 1289.21

1292.60 1296.69 1306.80 1309.43 1331.14 1351.78
1390.81 1399.94 1404.64 1406.25 1411.70 1425.73
1427.00 1445.14 1449.85 1453.12 1456.89 1457.48
1458.78 1463.29 1466.28 1466.39 1473.58 1481.38
1487.26 1491.03 1615.66 1724.52 1801.75 2039.36
3012.65 3014.85 3037.23 3042.90 3047.44 3052.58
3054.19 3054.43 3057.08 3071.19 3076.70 3095.63
3104.32 3110.99 3115.91 3116.26 3123.35 3128.88
3133.71 3135.73 3141.75 3154.66 3157.98 3173.89
3215.69 3237.23 3739.79

================================
TS-25-β
================================
-89.89 21.52 25.11 37.57 55.90 59.53
66.36 70.45 80.34 84.89 99.36 111.80
118.80 126.59 133.99 139.96 144.27 156.93
158.40 167.98 183.35 190.02 209.26 212.37
239.24 244.90 270.83 279.33 306.47 323.92
326.25 348.31 367.70 389.37 394.17 408.88
427.87 453.43 463.63 492.00 493.25 516.22
548.35 575.42 587.02 643.55 652.38 687.19
709.93 733.21 747.71 766.28 797.42 814.29
837.85 860.90 863.94 889.80 919.27 921.04
927.76 932.88 951.38 956.94 968.73 987.56
997.88 1000.21 1012.10 1030.62 1036.44 1043.52
1058.57 1073.66 1083.12 1088.72 1099.73 1114.74
1138.56 1156.82 1167.90 1179.18 1192.98 1206.41
1223.86 1224.57 1234.83 1244.32 1278.29 1288.38
1294.53 1305.85 1314.42 1320.10 1330.00 1366.03
1386.21 1394.07 1396.53 1401.74 1403.57 1425.03
1437.38 1449.98 1452.45 1454.15 1456.31 1460.51
1463.31 1465.17 1466.10 1470.89 1473.41 1475.14
1493.96 1498.08 1608.55 1708.27 1724.82 2084.80
3010.62 3013.88 3018.89 3040.69 3046.89 3051.12
3055.35 3060.78 3067.61 3075.40 3100.76 3101.51
3113.87 3122.99 3123.31 3128.87 3133.97 3137.19
3138.49 3141.60 3142.50 3143.84 3174.58 3221.69
3237.42 3249.41 3730.12

================================
TS-25-α
================================
-186.34 26.35 32.81 42.27 55.61 65.42
86.26 91.80 95.48 112.96 116.97 133.93
148.95 164.45 175.74 179.74 187.37 199.77
216.02 250.65 267.79 276.68 295.57 316.68
328.95 334.91 357.58 370.71 394.56 405.45
426.96 450.79 461.41 469.85 497.14 530.39
547.97 589.32 611.40 645.80 669.05 674.28
714.19 747.57 754.50 785.40 804.14 809.86
834.98 859.48 870.74 883.68 915.55 925.36
935.85 940.75 956.42 970.02 970.92 996.66
1005.43 1005.60 1019.13 1039.94 1045.02 1053.88
1068.42 1087.82 1093.42 1109.86 1125.92 1170.34
1189.80 1201.41 1204.90 1226.05 1233.22 1239.00
1255.44 1265.01 1277.91 1284.52 1292.86 1311.15
1331.44 1342.12 1369.45 1390.37 1398.80 1401.76
1405.39 1422.12 1449.24 1451.46 1452.86 1453.53
1455.83 1463.33 1464.70 1475.82 1478.99 1493.53
1587.88 1707.60 1797.15 2048.47 3022.11 3036.46
3041.73 3048.09 3054.19 3055.66 3061.08 3069.79
3087.08 3107.87 3119.91 3121.01 3129.83 3129.87
3134.98 3135.44 3136.95 3137.76 3139.98 3161.75
3227.63 3234.30 3712.91

References

(1) Neese, F.; Wennmohs, F.; Becker, U.; Riplinger, C. The ORCA Quantum Chemistry Program Package. *J. Chem. Phys.* **2020**, *152* (22), 224108–224125. DOI: 10.1063/5.0004608

(2) Becke, A. D. Density-Functional Thermochemistry. III. The Role of Exact Exchange. *J. Chem. Phys.* **1993**, *98* (7), 5648–5652. DOI: 10.1063/1.464913

(3) Becke, A. D.; Johnson, E. R. A Density-Functional Model of the Dispersion Interaction. *J. Chem. Phys.* **2005**, *123* (15), 154101–154109. DOI: 10.1063/1.2065267

(4) Weigend, F.; Ahlrichs, R. Balanced Basis Sets of Split Valence, Triple Zeta Valence and Quadruple Zeta Valence Quality for H to Rn: Design and Assessment of Accuracy. *Phys. Chem. Chem. Phys.* **2005**, *7* (18), 3297–3305. DOI: 10.1039/b508541a

(5) Weigend, F. Accurate Coulomb-Fitting Basis Sets for H to Rn. *Phys. Chem. Chem. Phys.* **2006**, *8* (9), 1057–1065. DOI: 10.1039/b515623h

(6) Zhao, Y.; Truhlar, D. G. Comparative DFT Study of van der Waals Complexes: Rare-Gas Dimers, Alkaline-Earth Dimers, Zinc Dimer, and Zinc-Rare-Gas Dimers. *J. Phys. Chem. A* **2006**, *110* (15), 5121–5129. DOI: 10.1021/jp060231d

(7) Zhao, Y.; Truhlar, D. G. Density Functional for Spectroscopy: No Long-Range Self-Interaction Error, Good Performance for Rydberg and Charge-Transfer States, and Better Performance on Average than B3LYP for Ground States. *J. Phys. Chem. A* **2006**, *110* (49), 13126–13130. DOI: 10.1021/jp066479k

(8) Barone, V.; Cossi, M. Quantum Calculation of Molecular Energies and Energy Gradients in Solution by a Conductor Solvent Model. *J. Phys. Chem. A* **1998**, *102* (11), 1995–2001. DOI: 10.1021/jp9716997

(9) Pracht, P.; Bohle, F.; Grimme, S. Automated Exploration of the Low-Energy Chemical Space With Fast Quantum Chemical Methods. *Phys. Chem. Chem. Phys.* **2020**, *22* (14), 7169–7192. DOI: 10.1039/c9cp06869d

(10) Ryu, H.; Park, J.; Kim, H. K.; Park, J. Y.; Kim, S. T.; Baik, M. H. Pitfalls in Computational Modeling of Chemical Reactions and How To Avoid Them. *Organometallics* **2018**, *37* (19), 3228–3239. DOI: 10.1021/acs.organomet.8b00456

(11) Yamamoto, Y.; Shibano, S.; Kurohara, T.; Shibuya, M. Synthesis of β-Allylbutenolides via One-Pot Copper-Catalyzed Hydroallylation/Cyclization of γ-Hydroxybutynoate Derivatives. *J. Org. Chem.* **2014**, *79* (10), 4503–4511. DOI: 10.1021/jo500536b

(12) Yamamoto, Y. Theoretical Study of the Copper-Catalyzed Hydroarylation of (Trifluoromethyl)alkyne with Phenylboronic Acid. *J. Org. Chem.* **2018**, *83* (20), 12775–12783. DOI: 10.1021/acs.joc.8b02215
