# Supplementary material for: Asymmetric Total Synthesis of 4,9,10-Trihydroxyguaia-11(13)en-12,6-olide and Discovery of Its Anticancer Activity against Atypical Teratoid Rhabdoid Tumor
Source: ACS Cent Sci. 2025 Jun 3;11(7):1103–10. doi: 10.1021/acscentsci.5c00332 (PMC12291133; doi:10.1021/acscentsci.5c00332)
Supplement: Supplementary file 4 [file oc5c00332_si_004.pdf]

–Supporting Information: Part D–

Asymmetric Total Synthesis of 4,9,10-Trihydroxyguaia-11(13)en-12,6-olide and Discovery of Its Anticancer Activity Against Atypical Teratoid Rhabdoid Tumor

Hyejin Lee[1], Hongjun Jang[1,2], Hwan Myung[3,4], Angela Rivera[5], Anna F. Averette[5], Joseph Heitman[5,6], Jiyong Park[4,3], Deukjoon Kim[7], Hyoungsu Kim[2], and Jiyong Hong[1,6,*]

[1] Department of Chemistry, Duke University, Durham, North Carolina 27708, United States

[2] College of Pharmacy and Research Institute of Pharmaceutical Science and Technology (RIPST), Ajou University, Suwon 16499, Republic of Korea

[3] Department of Chemistry, Korea Advanced Institute of Science and Technology (KAIST), Daejeon 34141, Republic of Korea

[4] Center for Catalytic Hydrocarbon Functionalizations, Institute for Basic Science (IBS), Daejeon 34141, Republic of Korea

[5] Department of Molecular Genetics and Microbiology, Duke University School of Medicine, Durham, North Carolina 27710, United States

[6] Department of Pharmacology and Cancer Biology, Duke University School of Medicine, Durham, North Carolina 27710, United States

[7] College of Pharmacy, Seoul National University, Seoul 08826, Republic of Korea

X-Ray Crystallography Data for Diol 27

A colorless crystal (approximate dimensions 0.180 × 0.080 × 0.050 mm^3) was placed onto the tip of MiTeGen and mounted on a Bruker D8 VENTURE diffractometer and measured at 150 K.

Data collection

A preliminary set of cell constants was calculated from reflections harvested from a set of 180 frames. These initial sets of frames were oriented such half a sphere in the reciprocal space was surveyed. This produced initial orientation matrices determined from 561 reflections. The data collection was carried out using Cu Kα radiation (graphite monochromator) with a frame time of 0.4 seconds with attenuator and a detector distance of 3.7 cm. A randomly oriented region of reciprocal space was surveyed to achieve complete data with a redundancy of 6.2. Sections of frames were collected with 0.50º steps in ω and ϕ scans. Data to a resolution of 0.82 Å were considered in the reduction. Final cell constants were calculated from the xyz centroids of 6801 strong reflections from the actual data collection after integration (SAINT).[1] The intensity data were corrected for absorption (SADABS).[2] Please refer to Table S5 for additional crystal and refinement information.

Structure solution and refinement

The space group $P2_1$ was determined based on intensity statistics and systematic absences. The structure was solved using SHELXT[3] and refined (full-matrix-least squares) using the Oxford University Crystals for Windows system.[4] The intrinsic-phasing solution provided most non-hydrogen atoms from the E-map. Full-matrix least squares / difference Fourier cycles were performed, which located the remaining non-hydrogen atoms. All non-hydrogen atoms were refined with anisotropic displacement parameters. The hydrogen atoms were placed in ideal positions and refined with individual relative isotropic displacement parameters. The final full matrix least squares refinement converged to R1 = 0.0287 and wR2 = 0.0685 (F^2, all data).

Structure description

The structure was found as proposed. The absolute structure is determined with Flack parameter of 0.03(13).

Figure S4. X-ray crystallographic structure of **27**

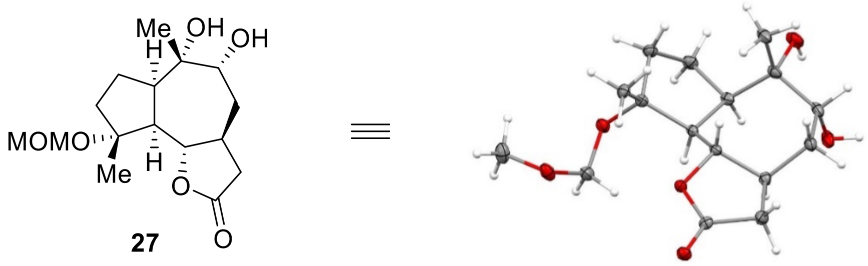

Figure S5. Molecular structure with labels on the asymmetric unit

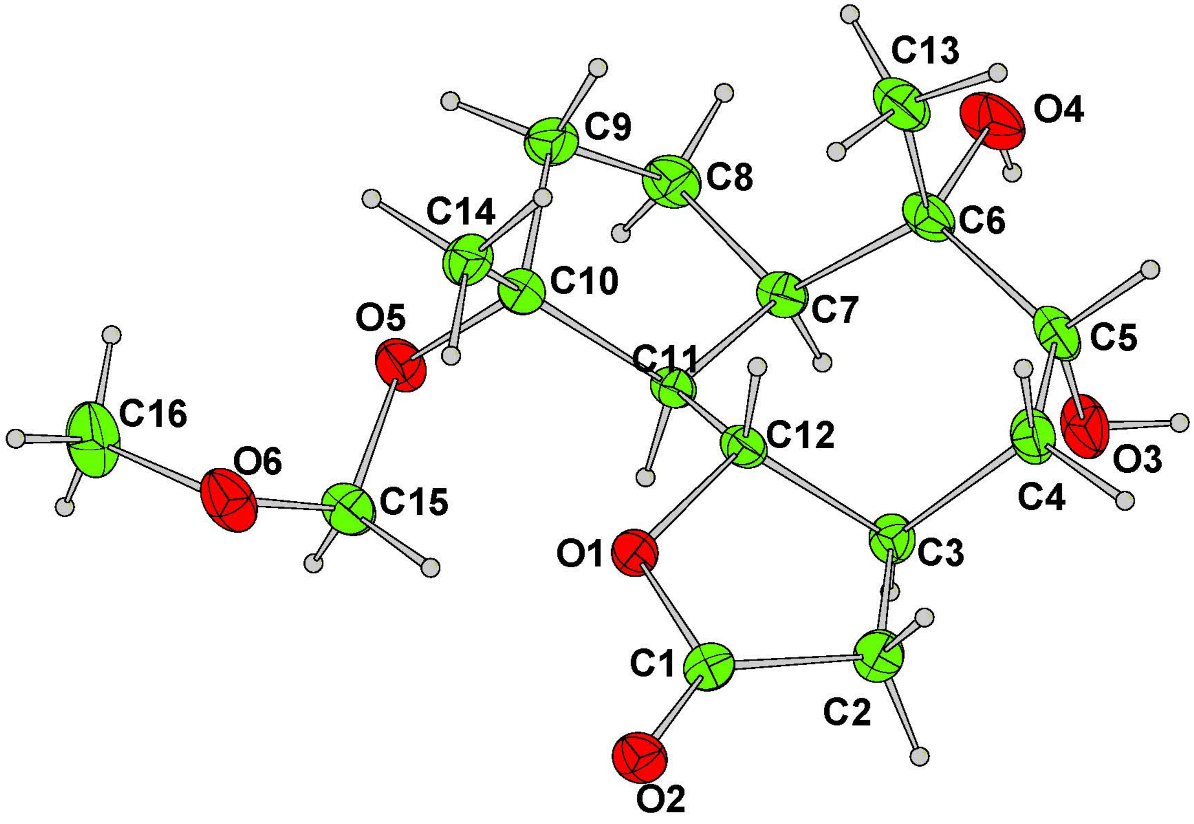

Figure S6. Cell plot, viewed along a-axis

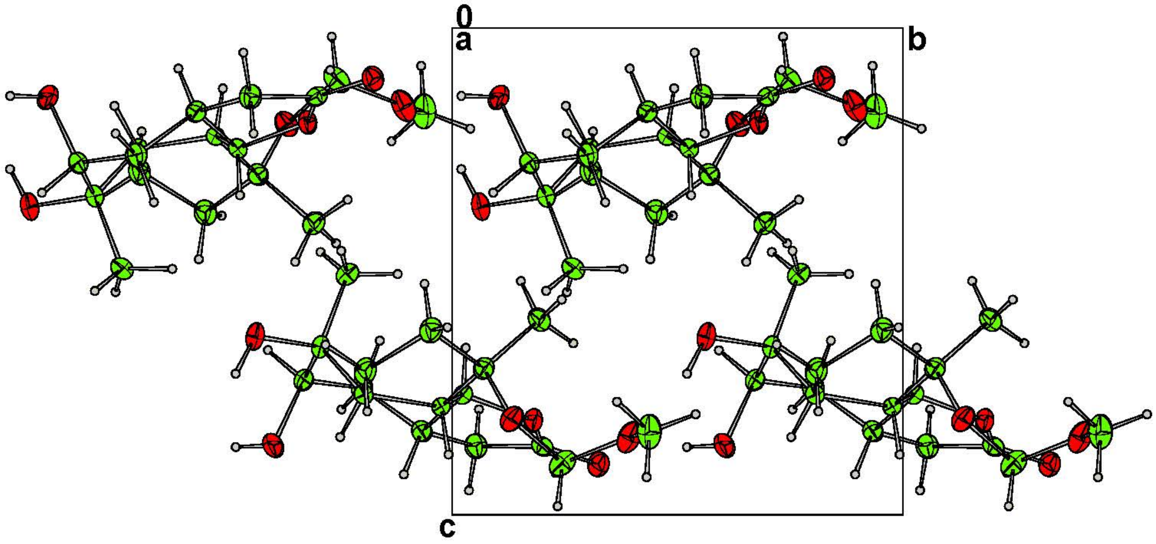

Figure S7. Cell plot, viewed along b-axis

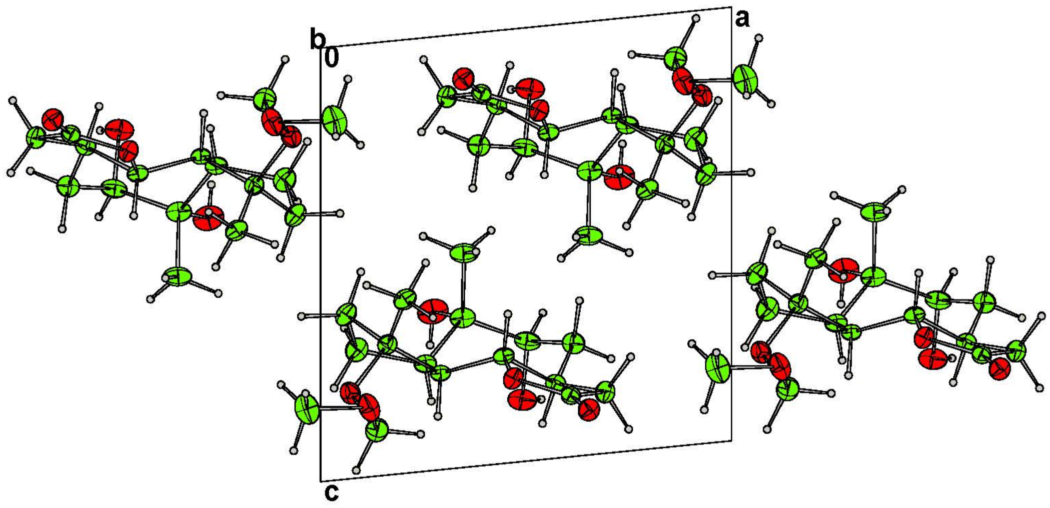

Figure S8. Cell plot, viewed along c-axis

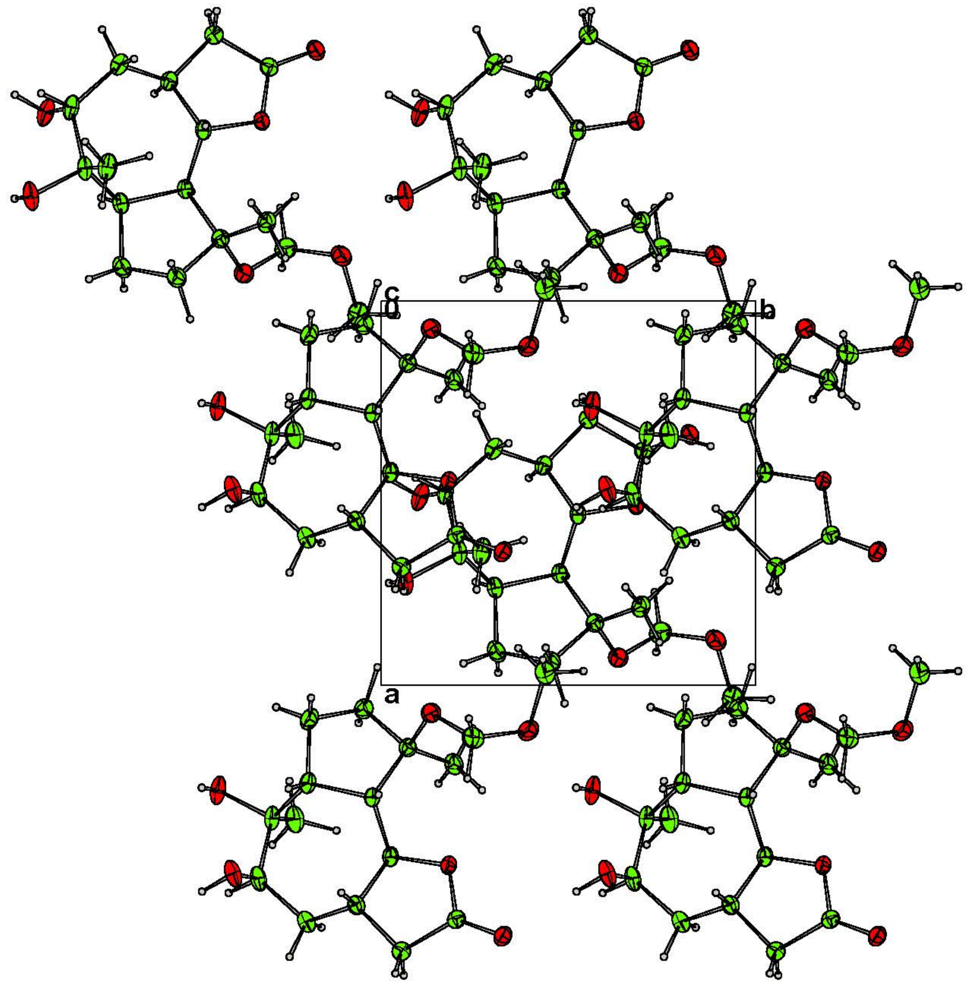

Table S6. Crystal data and structure refinement for **27**

Empirical formula	C16 H26 O6	
Formula weight	314.38	
Crystal color, shape, size	colorless block, 0.180 × 0.080 × 0.050 mm^3	
Temperature	150 K	
Wavelength	1.54178 Å	
Crystal system, space group	Monoclinic, P 1 21 1	
Unit cell dimensions	a = 9.1089(2) Å	α = 90°.
	b = 8.8281(2) Å	β = 95.6900(10)°.
	c = 9.5682(2) Å	γ = 90°.
Volume	765.63(3) $Å^3$	
Z	2	
Density (calculated)	1.364 mg/m^3	
Absorption coefficient	0.857 mm^{-1}	
F(000)	340	
Data collection		
Diffractometer	Bruker D8 VENTURE, Bruker	
Theta range for data collection	4.644 to 70.181°.	
Index ranges	-11<=h<=11, -10<=k<=10, -11<=l<=11	
Reflections collected	16152	
Independent reflections	2891 [R(int) = 0.046]	
Observed Reflections	2783	
Completeness to theta = 65.268°	99.5%	
Solution and Refinement		
Absorption correction	Semi-empirical from equivalents	
Max. and min. transmission	0.96 and 0.93	
Solution	Intrinsic phasing methods	
Refinement method	Full-matrix least-squares on F^2	
Weighting scheme	$w = [\sigma^2Fo^2 + AP^2 + BP]^{-1}$, with	
	$P = (Fo^2 + 2\ Fc^2)/3$, A = 0.035, B = 0.141	
Data / restraints / parameters	2882 / 5 / 280	
Goodness-of-fit on F^2	1.0086	
Final R indices [I>2sigma(I)]	R1 = 0.0287, wR2 = 0.0676	
R indices (all data)	R1 = 0.0297, wR2 = 0.0685	
Absolute structure parameter	0.03(13)	
Largest diff. peak and hole	0.17 and -0.14 e.$Å^{-3}$	

Table S7. Atomic coordinates (× 10^4) and equivalent isotropic displacement parameters (Å^2 × 10^3) for **27**. U(eq) is defined as one third of the trace of the orthogonalized U^{ij} tensor.

	x	y	z	U(eq)
O1	5316(1)	6802(1)	1940(1)	19
O2	3473(1)	8247(2)	1040(1)	22
O3	5096(1)	1040(2)	1424(1)	27
O4	7269(1)	640(2)	3665(1)	31
O5	9281(1)	6330(2)	1972(1)	22
O6	8824(1)	8929(2)	1597(1)	28
C1	3912(2)	7004(2)	1409(2)	18
C2	3077(2)	5533(2)	1412(2)	21
C3	4279(2)	4331(2)	1721(2)	18
C4	3847(2)	3004(2)	2611(2)	22
C5	4968(2)	1716(2)	2768(2)	22
C6	6545(2)	2084(2)	3452(2)	21
C7	7457(2)	3042(2)	2475(2)	18
C8	9122(2)	3077(2)	2957(2)	24
C9	9383(2)	4536(2)	3794(2)	23
C10	8380(2)	5705(2)	3012(2)	18
C11	7086(2)	4760(2)	2224(2)	16
C12	5545(2)	5251(2)	2474(2)	17
C13	6511(2)	2688(2)	4940(2)	27
C14	7949(2)	6937(2)	4009(2)	22
C15	8635(2)	7452(2)	1061(2)	27
C16	10332(2)	9377(2)	1710(2)	34

Table S8. Bond lengths [Å] and angles [°] for **27**

O1-C1	1.3401(17)	O1-C12	1.4688(17)
O2-C1	1.2087(18)	O3-C5	1.4331(19)
O3-H1	0.845(15)	O4-C6	1.4400(18)
O4-H2	0.834(16)	O5-C10	1.4599(18)
O5-C15	1.409(2)	O6-C15	1.405(2)
O6-C16	1.424(2)	C1-C2	1.505(2)
C2-C3	1.532(2)	C2-H21	0.963(19)
C2-H22	0.979(19)	C3-C4	1.523(2)
C3-C12	1.531(2)	C3-H31	0.988(18)
C4-C5	1.526(2)	C4-H41	1.01(2)
C4-H42	0.98(2)	C5-C6	1.553(2)
C5-H51	1.00(2)	C6-C7	1.559(2)
C6-C13	1.524(2)	C7-C8	1.541(2)
C7-C11	1.5681(19)	C7-H71	0.952(19)
C8-C9	1.523(2)	C8-H81	0.98(2)
C8-H82	0.96(2)	C9-C10	1.525(2)
C9-H91	1.036(19)	C9-H92	1.01(2)
C10-C11	1.5738(19)	C10-C14	1.524(2)
C11-C12	1.511(2)	C11-H111	0.959(18)
C12-H121	0.934(18)	C13-H131	0.98(2)
C13-H132	1.02(2)	C13-H133	0.94(2)
C14-H141	0.99(2)	C14-H142	1.00(2)
C14-H143	0.99(2)	C15-H151	1.00(2)
C15-H152	0.98(2)	C16-H161	0.97(3)
C16-H162	0.97(2)	C16-H163	1.01(3)

C1-O1-C12	110.69(11)	C5-O3-H1	107.8(15)
C6-O4-H2	110.8(17)	C10-O5-C15	117.33(12)
C15-O6-C16	111.53(13)	O1-C1-O2	120.50(13)
O1-C1-C2	110.35(12)	O2-C1-C2	129.13(13)
C1-C2-C3	104.28(12)	C1-C2-H21	106.5(11)
C3-C2-H21	111.1(11)	C1-C2-H22	110.2(11)
C3-C2-H22	112.5(11)	H21-C2-H22	111.8(15)
C2-C3-C4	114.93(13)	C2-C3-C12	102.33(12)
C4-C3-C12	111.67(12)	C2-C3-H31	110.3(11)
C4-C3-H31	109.0(11)	C12-C3-H31	108.4(10)
C3-C4-C5	114.90(13)	C3-C4-H41	108.2(11)
C5-C4-H41	108.2(11)	C3-C4-H42	109.1(11)
C5-C4-H42	105.5(11)	H41-C4-H42	110.9(15)

C4-C5-O3	109.57(12)	C4-C5-C6	117.92(12)
O3-C5-C6	108.12(13)	C4-C5-H51	108.8(10)
O3-C5-H51	107.3(11)	C6-C5-H51	104.6(10)
C5-C6-O4	105.43(12)	C5-C6-C7	112.74(12)
O4-C6-C7	107.54(13)	C5-C6-C13	111.28(14)
O4-C6-C13	103.22(12)	C7-C6-C13	115.57(13)
C6-C7-C8	113.50(13)	C6-C7-C11	119.83(13)
C8-C7-C11	102.55(12)	C6-C7-H71	103.1(11)
C8-C7-H71	108.8(11)	C11-C7-H71	108.8(11)
C7-C8-C9	105.78(13)	C7-C8-H81	109.1(11)
C9-C8-H81	108.9(12)	C7-C8-H82	111.7(12)
C9-C8-H82	112.5(12)	H81-C8-H82	108.8(17)
C8-C9-C10	105.07(12)	C8-C9-H91	112.9(11)
C10-C9-H91	110.1(11)	C8-C9-H92	108.7(11)
C10-C9-H92	107.3(11)	H91-C9-H92	112.4(15)
C9-C10-O5	103.91(11)	C9-C10-C11	105.08(12)
O5-C10-C11	108.56(11)	C9-C10-C14	110.80(13)
O5-C10-C14	110.73(12)	C11-C10-C14	116.85(12)
C7-C11-C10	107.42(11)	C7-C11-C12	116.27(12)
C10-C11-C12	115.81(11)	C7-C11-H111	105.2(11)
C10-C11-H111	105.9(10)	C12-C11-H111	105.2(10)
C3-C12-C11	116.21(12)	C3-C12-O1	104.86(11)
C11-C12-O1	108.32(11)	C3-C12-H121	110.3(10)
C11-C12-H121	110.6(10)	O1-C12-H121	105.9(11)
C6-C13-H131	107.1(13)	C6-C13-H132	111.9(12)
H131-C13-H132	110.2(17)	C6-C13-H133	108.3(13)
H131-C13-H133	107.9(18)	H132-C13-H133	111.3(17)
C10-C14-H141	111.9(12)	C10-C14-H142	107.4(11)
H141-C14-H142	110.9(16)	C10-C14-H143	112.7(12)
H141-C14-H143	106.9(16)	H142-C14-H143	107.0(16)
O5-C15-O6	113.47(14)	O5-C15-H151	104.8(11)
O6-C15-H151	110.5(12)	O5-C15-H152	111.6(12)
O6-C15-H152	106.2(12)	H151-C15-H152	110.2(16)
O6-C16-H161	106.6(14)	O6-C16-H162	107.4(13)
H161-C16-H162	112(2)	O6-C16-H163	111.3(13)
H161-C16-H163	112.6(20)	H162-C16-H163	107.1(19)

Symmetry transformations used to generate equivalent atoms:

Table S9. Anisotropic displacement parameters ($Å^2 \times 10^3$) for **27**. The anisotropic displacement factor exponent takes the form: $-2\pi^2[h^2 a^{*2}U^{11} + ... + 2 h k a^* b^* U^{12}]$

	U^{11}	U^{22}	U^{33}	U^{23}	U^{13}	U^{12}
O1	19(1)	14(1)	23(1)	2(1)	-1(1)	1(1)
O2	23(1)	18(1)	24(1)	1(1)	1(1)	4(1)
O3	42(1)	17(1)	23(1)	-5(1)	6(1)	-10(1)
O4	47(1)	14(1)	31(1)	4(1)	0(1)	6(1)
O5	21(1)	22(1)	25(1)	6(1)	5(1)	0(1)
O6	23(1)	23(1)	39(1)	10(1)	10(1)	4(1)
C1	19(1)	18(1)	16(1)	-3(1)	2(1)	2(1)
C2	20(1)	20(1)	24(1)	-1(1)	1(1)	0(1)
C3	21(1)	17(1)	18(1)	-2(1)	3(1)	-2(1)
C4	26(1)	18(1)	23(1)	0(1)	4(1)	-5(1)
C5	35(1)	15(1)	18(1)	2(1)	7(1)	-5(1)
C6	32(1)	12(1)	20(1)	2(1)	2(1)	1(1)
C7	25(1)	15(1)	16(1)	-1(1)	1(1)	2(1)
C8	26(1)	19(1)	28(1)	2(1)	0(1)	6(1)
C9	23(1)	20(1)	25(1)	2(1)	-4(1)	1(1)
C10	19(1)	17(1)	19(1)	2(1)	1(1)	0(1)
C11	20(1)	14(1)	13(1)	1(1)	1(1)	1(1)
C12	22(1)	14(1)	15(1)	2(1)	1(1)	0(1)
C13	41(1)	20(1)	19(1)	4(1)	4(1)	-3(1)
C14	25(1)	19(1)	21(1)	-3(1)	-2(1)	-3(1)
C15	25(1)	28(1)	27(1)	9(1)	1(1)	-2(1)
C16	31(1)	23(1)	48(1)	0(1)	9(1)	-2(1)

Table S10. Hydrogen coordinates (× 10^4) and isotropic displacement parameters ($Å^2$ × 10^3) for **27**

	x	y	z	U(eq)
H21	2450(20)	5600(20)	2170(20)	25
H22	2500(20)	5380(20)	500(20)	26
H31	4603(18)	3940(20)	835(19)	21
H41	3700(20)	3400(20)	3580(20)	27
H42	2930(20)	2550(20)	2170(20)	26
H51	4596(19)	920(20)	3380(20)	28
H71	7309(19)	2520(20)	1600(20)	20
H81	9680(20)	3120(20)	2120(20)	30
H82	9430(20)	2200(20)	3490(20)	30
H91	10470(20)	4900(20)	3860(20)	28
H92	9020(20)	4390(20)	4750(20)	28
H111	7151(17)	4920(20)	1240(19)	17
H121	5447(18)	5300(20)	3435(19)	20
H131	7510(20)	2550(20)	5420(20)	39
H132	6220(20)	3800(30)	4950(20)	38
H133	5850(20)	2090(30)	5400(20)	40
H141	7270(20)	7690(20)	3530(20)	31
H142	8880(20)	7430(20)	4420(20)	33
H143	7450(20)	6530(20)	4810(20)	34
H151	9130(20)	7340(20)	180(20)	30
H152	7570(20)	7300(20)	860(20)	31
H161	10370(20)	10400(30)	2070(30)	51
H162	10660(20)	9320(30)	780(30)	50
H163	10970(20)	8660(30)	2330(30)	51
H1	4630(20)	210(20)	1400(20)	41(2)
H2	7340(30)	210(30)	2900(19)	46(2)

Table S11. Torsion angles [°] for **27**

C12-O1-C1-O2	172.92(13)	C12-O1-C1-C2	-5.38(16)
C1-O1-C12-C3	20.98(15)	C1-O1-C12-C11	145.68(12)
C15-O5-C10-C9	179.36(13)	C15-O5-C10-C11	67.90(17)
C15-O5-C10-C14	-61.64(17)	C10-O5-C15-O6	88.30(17)
C16-O6-C15-O5	66.61(18)	O1-C1-C2-C3	-12.40(16)
O2-C1-C2-C3	169.50(15)	C1-C2-C3-C4	144.96(13)
C1-C2-C3-C12	23.74(15)	C2-C3-C4-C5	172.86(13)
C12-C3-C4-C5	-71.15(17)	C2-C3-C12-O1	-26.96(14)
C2-C3-C12-C11	-146.51(13)	C4-C3-C12-O1	-150.41(12)
C4-C3-C12-C11	90.04(16)	C3-C4-C5-O3	-64.30(18)
C3-C4-C5-C6	59.90(18)	O3-C5-C6-O4	-64.02(15)
O3-C5-C6-C7	53.02(17)	O3-C5-C6-C13	-175.27(13)
C4-C5-C6-O4	171.06(13)	C4-C5-C6-C7	-71.91(18)
C4-C5-C6-C13	59.81(18)	O4-C6-C7-C8	-49.53(17)
O4-C6-C7-C11	-171.00(12)	C5-C6-C7-C8	-165.31(14)
C5-C6-C7-C11	73.21(18)	C13-C6-C7-C8	65.13(19)
C13-C6-C7-C11	-56.34(19)	C6-C7-C8-C9	-96.48(16)
C11-C7-C8-C9	34.23(15)	C6-C7-C11-C10	108.98(15)
C6-C7-C11-C12	-22.60(19)	C8-C7-C11-C10	-17.78(15)
C8-C7-C11-C12	-149.36(13)	C7-C8-C9-C10	-38.44(16)
C8-C9-C10-O5	-88.03(14)	C8-C9-C10-C11	25.95(15)
C8-C9-C10-C14	153.01(13)	O5-C10-C11-C7	105.93(13)
O5-C10-C11-C12	-122.24(14)	C9-C10-C11-C7	-4.74(15)
C9-C10-C11-C12	127.09(14)	C14-C10-C11-C7	-128.01(14)
C14-C10-C11-C12	3.8(2)	C7-C11-C12-O1	-169.62(12)
C7-C11-C12-C3	-51.97(18)	C10-C11-C12-O1	62.83(16)
C10-C11-C12-C3	-179.52(13)		

Symmetry transformations used to generate equivalent atoms:

References

(1) SAINT, Bruker Analytical X-Ray Systems, Madison, WI.

(2) Blessing, R. H. An Empirical Correction for Absorption Anisotropy. *Acta Crystallogr. Sect. A* **1995**, *51* (1), 33–38. DOI: 10.1107/s0108767394005726

(3) Sheldrick, G. M. *SHELXT* – Integrated Space-Group and Crystal-Structure Determination. *Acta Crystallogr. Sect. A* **2015**, *71* (1), 3–8. DOI: 10.1107/S2053273314026370

(4) Betteridge, P. W.; Carruthers, J. R.; Cooper, R. I.; Prout, K.; Watkin, D. J. *CRYSTALS* Version 12: Software for Guided Crystal Structure Analysis. *J. Appl. Crystallogr.* **2003**, *36* (6), 1487–1487. DOI: 10.1107/S0021889803021800
